# Diabetes is causally associated with increased breast cancer mortality by inducing FIBCD1 to activate MCM5-mediated cell cycle arrest via modulating H3K27ac

**DOI:** 10.1038/s41419-025-07849-w

**Published:** 2025-07-22

**Authors:** Binbin Tan, Yang Liu, Qianqian Chen, Weijie Yang, Wenhan Yang, Kaiping Gao, Li Fu, Tiantian Zhang, Penglong Chen, Yongyi Huang, Yuting Wang, Guoqiang Zhang, Juan Xiong, Rihong Zhai

**Affiliations:** 1 Department of Pharmacology and International Cancer Center, Guangdong Provincial Key Laboratory of Regional Immunity and Diseases, Shenzhen University Medical School, Shenzhen, China; 2 Department of Surgery, Harbin Medical University Cancer Hospital, Harbin, China; 3 Cancer Centre, Faculty of Health Sciences, University of Macau, Macau, SAR China; 4 National Clinical Research Center for Infectious Disease, Shenzhen Third People’s Hospital, Southern University of Science and Technology, Shenzhen, China; 5 School of Public Health, Guangdong Key Laboratory for Genome Stability & Disease Prevention, Shenzhen University Medical School, Shenzhen, China

**Keywords:** Cancer epigenetics, Oncogenesis

## Abstract

Breast cancer (BC) is the most common tumor worldwide and it has been recognized that up to one third of BC patients have co-existing diabetes mellitus (DM) (BC-DM). Although many observational studies have indicated an association between DM and BC, the causal relationship of DM and BC prognosis remained uncertain and the molecular mechanisms underlying BC-DM are largely unclear. In this study, we used causal inference methods, including g-computation (GC), inverse probability of treatment weighting (IPTW), targeted maximum likelihood estimation (TMLE), and TMLE-super learner (TMLE-SL), to comprehensively analyze the association of DM with BC mortality in a cohort of 3386 BC patients. We found that the adjusted odds ratios (OR) and 95% confidence intervals (95% CI) for 5-year mortality in BC-DM patients were 1.926 (1.082, 2.943), 2.268 (1.063, 3.974), 1.917 (1.091, 2.953), and 2.113 (1.365, 3.270), respectively. Further transcriptomic and qPCR analyses identified that FIBCD1 was highly expressed in BC-DM tumor tissues and in BC cells under hyperglycemia conditions. Functionally, upregulation of FIBCD1 promoted proliferation, migration, and invasion capacities of BC cells in a glucose level-dependent manner. While knockdown of FIBCD1 suppressed BC tumor growth in diabetic mice. Integrated RNA-seq and Ribo-seq analysis revealed that MCM5 was a target of FIBCD1. Mechanistically, hyperglycemia-activated FIBCD1 promoted MCM5 expression to induce S-phase cell cycle arrest by upregulating histone H3K27ac levels in MCM5 promoter via the PDH-acetyl-CoA axis. Our findings provide new evidence that co-existing DM has a causal effect on overall mortality in BC-DM patients. Targeting FIBCD1 may be a promising therapy for BC-DM.

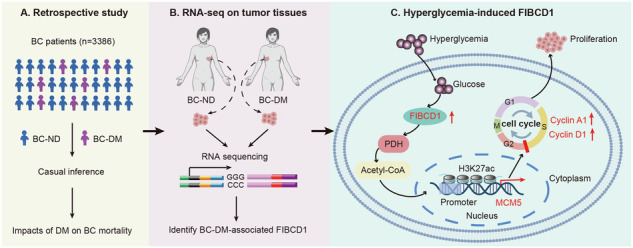

## Introduction

Breast Cancer (BC) has surpassed lung cancer as the most common malignant neoplasm in the world, with an estimated 2.3 million cases and 685,000 deaths in 2020 [1]. Among patients with BC, up to one third cases had diagnosed diabetes mellitus (DM) as comorbidity [2–6], resulting in a mixed-phenotype of BC who have co-existing BC and DM (BC-DM). Indeed, several meta-analyses have found a pooled ~20% increased risk of developing BC among women with preexisting DM [7–9]. Multiple studies have also examined the impact of preexisting DM on BC prognosis, but the findings are inconsistent. Some studies showed that DM was associated with elevated hazard of all-cause mortality in BC patients [10–13]. However, contrasting studies have reported no discernible link between DM and either all-cause mortality or breast cancer-specific survival [14–17]. Thus, it remains uncertain whether DM has a causal effect on BC prognosis [18].

Most existing observational studies on the association between DM and BC mortality have used Cox proportional hazard regression models, which have the problem of covariate imbalance due to the non-random allocation of exposure [19]. Second, these approaches tend to be biased because the causal relationship is based on an assumption of linear association. However, it is likely that nonlinear relationships between many clinicopathologic features are present in clinical practice [20, 21]. Moreover, current reports on the association of DM with BC survival are basically hypothesis generating, the underlying molecular mechanisms linking DM to BC prognosis remain largely unknown. In recent years, several causal inference approaches, including inverse probability of treatment weighting (IPTW), G-computation (GC), and targeted maximum likelihood estimation (TMLE), have been developed to estimate marginal causal effects in observational studies. These approaches mimic a randomized controlled trial where the measured confounders are well balanced by certain weighting procedures, thus allowing for more valid statistical comparisons [22]. Notably, causal inference methods combined with machine learning have greater flexibility to capture non-linear effects of causal variables [23]. In fact, a growing body of reports has demonstrated that causal inference methods have better performance than traditional models across several disease processes [24–26]. However, there are no reports in using causal inference methods to estimate the causal effect of DM on BC outcomes.

The primary aim of the present study was to use multiple causal inference approaches to estimate the causal relationship between co-existing DM and the prognosis of BC in a large cohort of 3386 patients with BC. Secondarily, we aimed to identify functional genes in mediating BC-DM and to elucidate the molecular mechanisms underlying the pathogenesis of BC-DM. To our knowledge, this research constitutes the first study to investigate the causal effect of DM on BC outcome using integrated observational and molecular approaches.

## Results

### Clinical characteristics of patients with BC-DM

A total of 3386 female patients with primary non-metastatic invasive BC were included in this study (Table S1). All patients underwent radical resection of primary tumor. Of these patients, 300 (8.86%) were diagnosed as having DM, which was comparable to previous report in which BC-DM rate in Asian BC patients was 7.7% [27]. Within the median follow-up period of 53 months, the 5-year mortality rate for BC-DM was significantly higher than that for BC without DM (BC-ND) (11.33% versus 5.87%; *P* < 0.001). Clinical characteristics between patients with and without DM are shown in Table 1. Compared with BC-ND patients, BC-DM cases tended to be older (mean age, 58.5 versus 50.0; *P* = 0.001) with a higher BMI (mean value, 25.4 versus 23.6; *P* < 0.001), and were more likely to have advanced stages of disease. There were no statistically significant differences between the two groups with regards to the positive rate of PR (*P* = 0.263), ER+ (*P* = 0.599), or HER-2+ (*P* = 0.987).

**Table 1:** Comparison of clinical characteristics for BC-DM and BC-NO-DM patients.

Variable	Overall *N* = 3386	BC-ND *N* = 3086	BC-DM *N* = 300	*P*-value
5-year mortality	215 (6.35%)	181 (5.87%)	34 (11.33%)	**<0.001**
Age	50.50 (44.00, 58.00)	50.00 (44.00, 58.00)	58.50 (52.00, 64.00)	**<0.001**
Lymphatic_metastasis	2329 (68.78%)	2117 (68.60%)	212 (70.67%)	0.461
Ki67	1391 (41.08%)	1260 (40.83%)	131 (43.67%)	0.340
BMI	23.83 (21.64, 26.02)	23.62 (21.48, 25.78)	25.39 (23.49, 27.84)	**<0.001**
Stage I	1535 (45.33%)	1411 (45.72%)	124 (41.33%)	0.145
Stage II	1376 (40.64%)	1258 (40.76%)	118 (39.33%)	0.630
Stage III	475 (14.03%)	417 (13.51%)	58 (19.33%)	**0.006**
HER2	2507 (74.04%)	2285 (74.04%)	222 (74.00%)	0.987
ER	2459 (72.62%)	2247 (72.81%)	212 (70.67%)	0.426
PR	2156 (63.67%)	1978 (64.10%)	178 (59.33%)	0.102
P53_type	1307 (38.60%)	1199 (38.85%)	108 (36.00%)	0.333
Post-Menopause	1568 (46.31%)	1428 (46.27%)	140 (46.67%)	0.896

*Ki-67* Ki-67 protein positive, *BMI* Body Mass Index, *HER* human epidermal growth factor receptor, *ER* estrogen receptor, *PR* progesterone receptor. A Unpaired t-test or Mann–Whitney U test for continuous variables and Chi-squared test for categorical variables were used for statistical comparison.

Bold values to emphasize that they are statistically significant.

### Causal association between DM and overall BC mortality

**Fig. 1:**
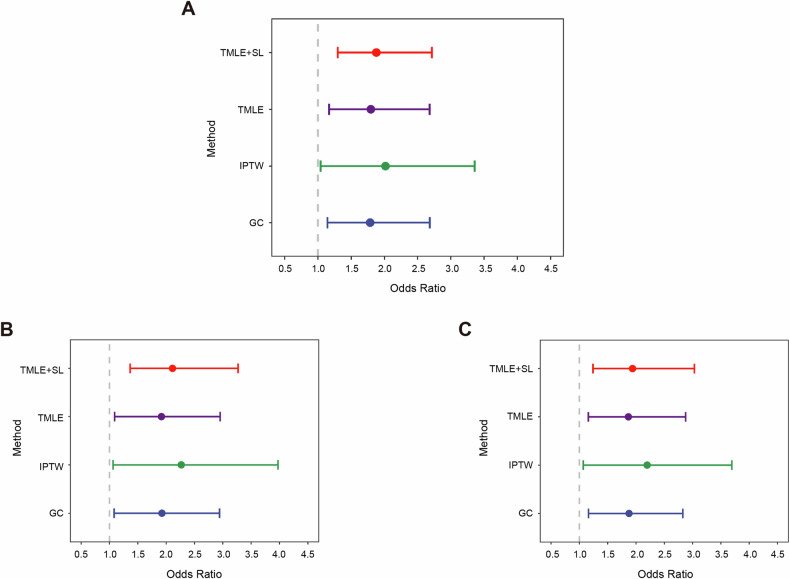
Estimated 5-year mortality odds ratio (OR) by co-existing DM in patients with BC. Points represent adjusted ORs and lines indicate 95% confidence interval (CI). **A** ORs are assessed by G-computation (GC), inverse probability of treatment weighting (IPTW), targeted maximum likelihood estimation (TMLE) and TMLE with super-learner (TMLE-SL), respectively. **B** Sensitivity analysis was conducted by excluding subjects with stage III. **C** Sensitivity analysis was performed by excluding subjects with age > =70. All ORs are adjusted for baseline age, BMI, Ki67, stage, HER2, ER, PR, lymphatic metastasis, hypertension, and menopause status.

We implemented four causal inference methods on a real-life clinical BC dataset to assess the marginal causal effect of co-existing DM on the 5-year BC mortality. All estimated odds ratio (OR) and 95% confidence interval (CI) were adjusted by the baseline age, BMI, Ki67, P53, stage, HER2, ER, PR, lymphatic metastasis, hypertension and menopause status. The 95% CIs of GC, IPTW, and TMLE were calculated by bootstrap sampling, while the 95% CI associated with TMLE-SL was based on the efficient influence curve. Figure 1A contrasts the estimating results of causal inference by four different approaches. In general, ORs of 5-year death for BC-DM estimated by TMLE, TMLE-SL, IPTW, and GC were all >1.7, indicating that co-existing DM had increased causal effect on BC mortality. But IPTW generated a wider 95% CI than the other three estimators. For example, the OR of 5-year death for BC-DM patients was estimated to be 1.784 (95% CI: 1.143–2.684) by GC and 2.014 (95% CI: 1.040–3.359) by IPTW. The ORs calculated by TMLE (OR = 1.794, 95%CI 1.168–2.682) and TMLE-SL (OR = 1.877, 95%CI 1.297, 2.716) were very similar, but TMLE-SL tended to yield higher OR and narrower 95% CI than that of TMLE, suggesting that combined use of non-parametric super learner may improve the estimating efficiency of TMLE.

Since late TNM stage and older age are known risk factors for worse BC prognosis [27, 28], we performed two sets of sensitivity analysis to assess the robustness of causal inference models. In the first set, after subjects with stage III were excluded from the models, the causal association between DM and BC mortality was still statistically significant (Table S2, Fig. 1B). In the second set, ORs by analyses in patients excluding subjects with age ≥ 70 were consistent with that of the total sample (Table S3, Fig. 1C). These results suggest that late stage and elderly age had limited influence on the causal association between DM and BC mortality.

### Transcriptional profiles in BC-DM tumor tissues were distinct from that in BC-ND tumor tissues

**Fig. 2:**
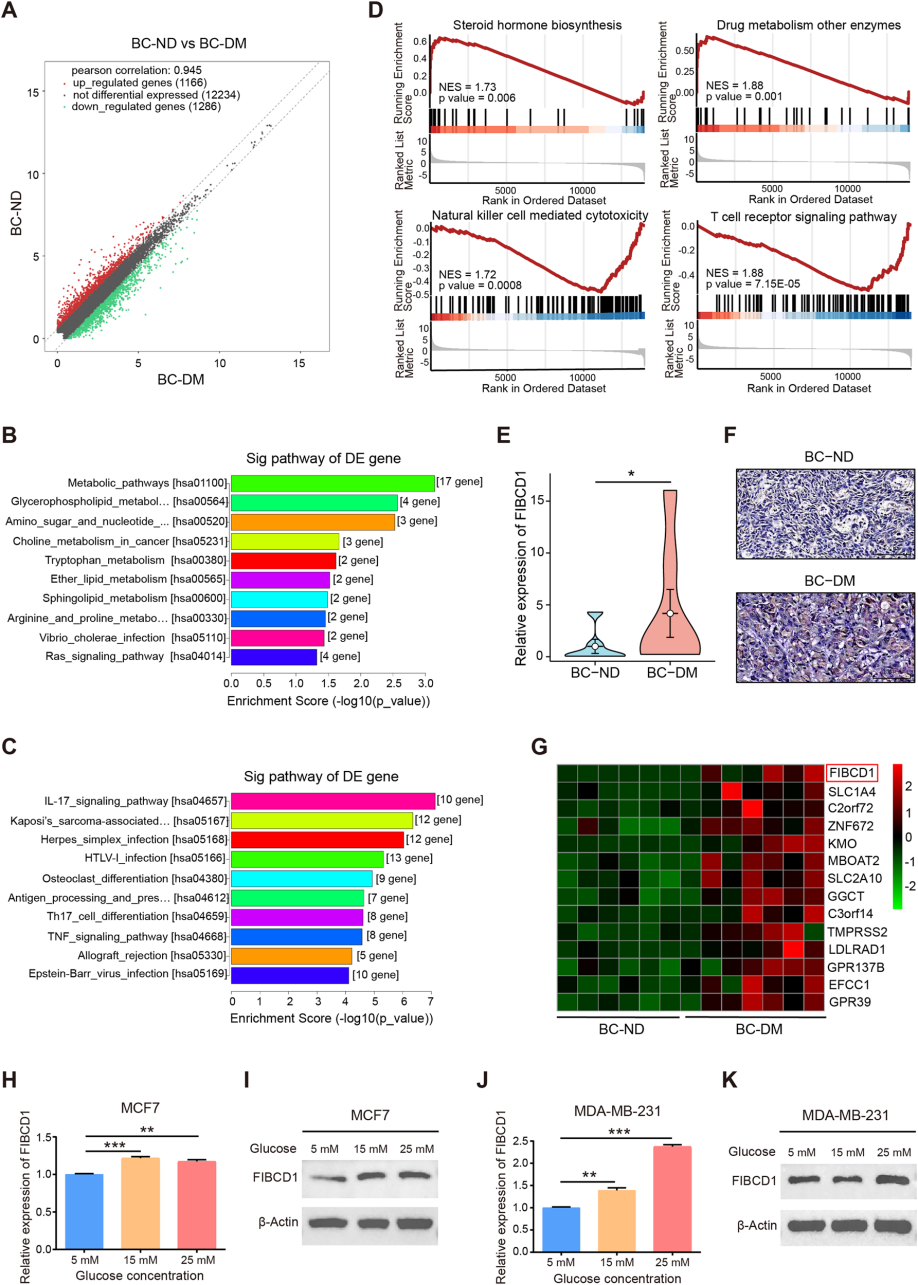
FIBCD1 is upregulated in BC-DM and under hyperglycemia conditions. **A** Scatter plotting shows differentially expressed genes between BC-DM tumor tissues and BC-ND tumor tissues. The red dots and green dots represent upregulated and downregulated mRNAs with statistical significance, respectively. **B** KEGG pathway analysis of significantly up-regulated mRNAs suggests that upregulated genes in BC-DM are enriched in metabolism-related pathways. **C** KEGG pathway analysis of significantly down-regulated mRNAs. **D** Gene set enrichment analysis (GSEA) revealed functional enrichments of differentially expressed genes in BC-DM tumor tissues. Normalized enrichment score (NES) and *p*-values are indicated. **E** FIBCD1 gene expression levels (detected by qRT-PCR assay) in BC-DM tissues are higher than that in BC-ND tissues. **F** Representative IHC images showing that FIBCD1 protein expression level in BC-DM tissue is higher than that in BC-ND tissue. **G** Top upregulated genes in BC-DM tumor tissues with FIBCD1 ranks as the top one DEG. **H** FIBCD1 gene expression levels in MCF7 cells are glucose dependent. **I** FIBCD1 protein expression is related to glucose concentration in MCF7 cells. **J** FIBCD1 gene expression levels in MDA-MB-231 cells under hyperglycemic conditions are higher than in euglycemic conditions. **K** FIBCD1 protein expression is related to glucose concentration in MDA-MB-231 cells. Data are presented as mean ± SD, Student’s t test. *P < 0.05; **P < 0.01; ***P < 0.001.

Given that DM was causatively associated with BC mortality, we sought to investigate the underlying molecular mechanisms of BC-DM. RNA sequencing showed distinct transcriptome profiles between tumor tissues of BC-DM and BC-ND (Fig. 2A). Among the differentially expressed genes (DEGs), 148 were upregulated and 142 were downregulated in BC-DM tissues versus BC-ND tissues (|fold change | ≥1.5, *P* < 0.05) (Table S4). Functional enrichment analysis with Kyoto Encyclopedia of Genes and Genomes (KEGG) showed that up-regulated DEGs were significantly correlated to metabolic and cancer-related pathways (Fig. 2B). Whereas down-regulated DEGs were more enriched in immune-related pathways, including IL-17 signaling, Th17 cell differentiation, and Th1 and Th2 cell differentiation pathways (Fig. 2C). Moreover, gene set enrichment analysis (GSEA) suggested that gene sets associated with steroid biosynthesis and protein metabolisms were enriched in BC-DM tumor tissues (Fig. 2D). Collectively, RNA-sequencing results from tumor tissues suggest that BC-DM and BC-ND are two distinct subtypes of BC at transcriptional levels.

### FIBCD1 was up-regulated in BC-DM and in BC cells under hyperglycemia conditions

Among the top-scoring DEGs in BC-DM tumors, FIBCD1 was the most significantly dysregulated gene (Fig. 2G), and the higher expressions of FIBCD1 gene and protein in BC-DM tumor tissues were further verified by qRT-PCR and IHC assays (Fig. 2E, F). Thus, we focused on elucidating the biological functions of FIBCD1 in subsequent experiments. To evaluate the potential influence of glucose concentration on FIBCD1 expression, BC cells were incubated under different concentrations of glucose ranging from 5, 15 to 25 mM, respectively. The results clearly showed that the expression levels of FIBCD1 in BC cell lines (MCF7, MDA-MB-231) were in glucose level-dependent manner: both FIBCD1 gene and protein expression levels under hyperglycemia conditions were higher than that in physiological glucose levels (Fig. 2H–K). These findings indicate that FIBCD1 is a glucose level-sensitive gene in BC cells.

### Hyperglycemia increased the proliferation, invasion, and migration capacities of BC cells

**Fig. 3:**
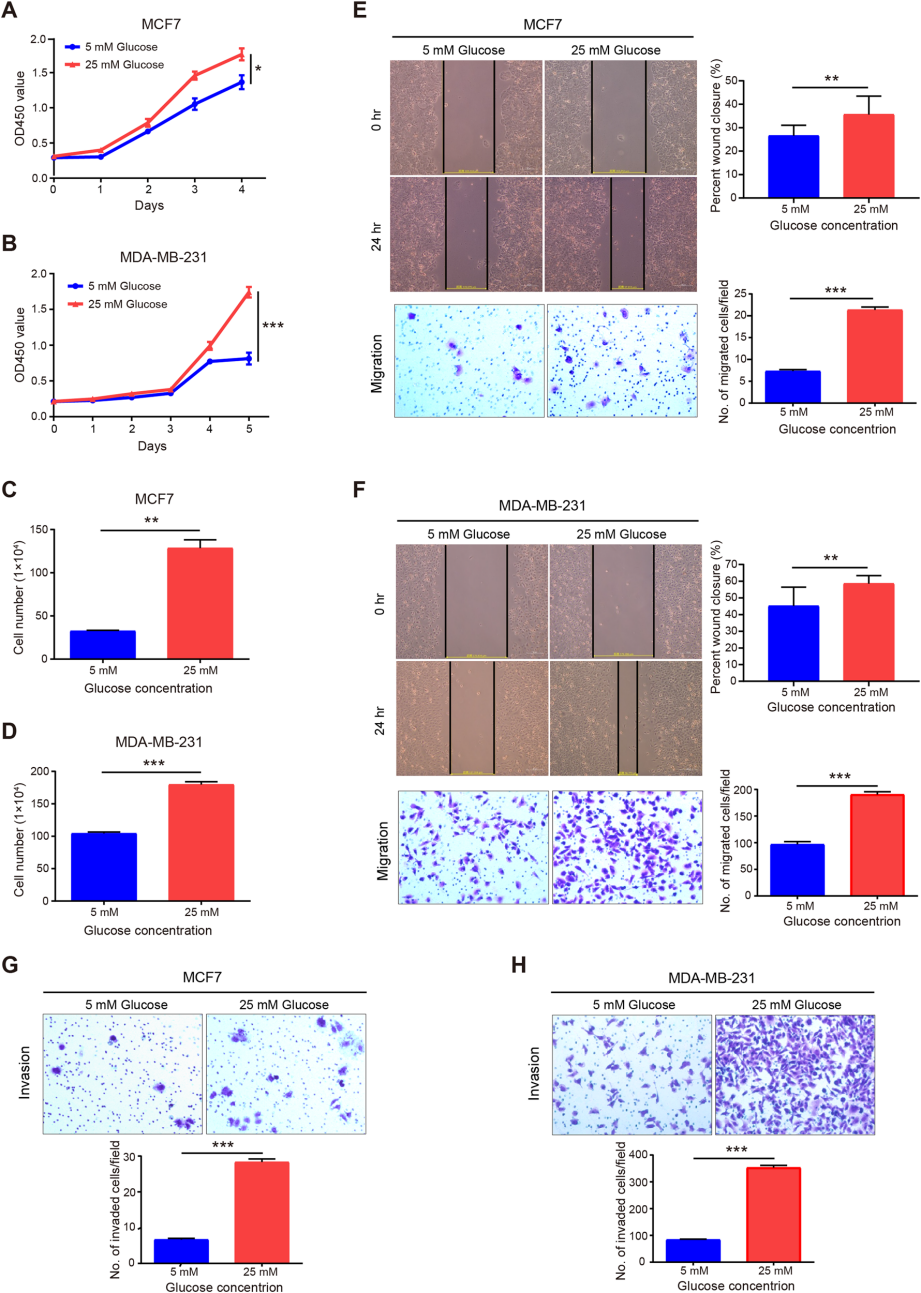
High glucose promotes cell proliferation, migration, invasion, and expression of FIBCD1 in BC cells. **A** Proliferation rate of MCF7 cells is higher under hyperglycemic (25 mM glucose) culture conditions than that under euglycemic (5 mM glucose) culture conditions determined by CCK8 assay. **B** Proliferation capacity of MDA-MB-231 cells is higher under hyperglycemia conditions than that of euglycemic conditions. **C** Proliferation of MCF7 cells is determined by cell counting assay. **D** Proliferation of MDA-MB-231 cells is examined by cell counting assay. **E** Hyperglycemia promotes migration ability of MCF7 cells. **F**. Hyperglycemia enhances migration capacity of MDA-MB-231 cells. **G** Hyperglycemia augments invasion potential of MCF7 cells. **H** Hyperglycemia increases invasion capability of MDA-MB-231 cells. Data are shown as mean ± SD, Student’s t test. *P < 0.05; **P < 0.01; ***P < 0.001.

To assess the influence of glucose level on malignant behaviors of BC cells, BC cells were incubated under euglycemic (5 mM) and hyperglycemic (25 mM) levels of glucose, respectively. CCK8 assay showed that the proliferation rates of both MCF7 and MDA-MB-231 cells under hyperglycemia conditions were significantly higher than that under physiological glucose levels (Fig. 3A, B). Similar results were confirmed by cell counting assays (Fig. 3C, D). Furthermore, transwell chambers and wound-healing assay were used to evaluate cell migration and invasion capacities. The results revealed that higher glucose enhanced the migration rates of both MCF7 and MDA-MB-231 cells (Fig. 3E, F). Moreover, the invasive capacities of both cell lines were significantly increased by supplementation with high concentration of glucose (Fig. 3G, H). Collectively, these data suggest that high glucose levels promote the malignancy of BC cells. However, the molecular mechanisms underlying the effect of hyperglycemia on BC cells are unclear.

### FIBCD1 promoted the malignancy of BC cells in a glucose level-dependent manner

**Fig. 4:**
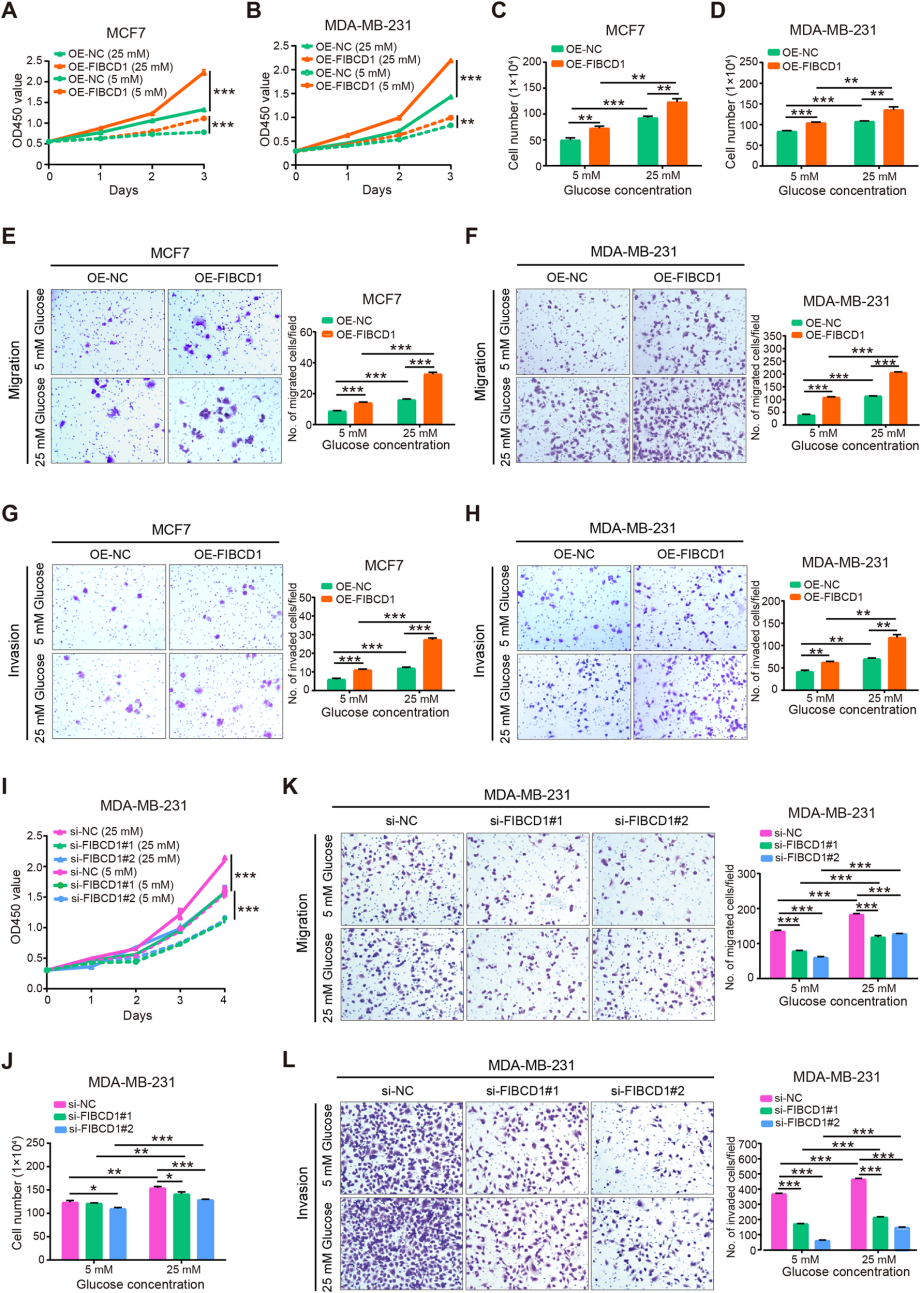
FIBCD1 enhances the malignancy of BC cells in a glucose level-dependent manner. BC cells were incubated in hyperglycemic (25 mM glucose) or euglycemic (5 mM glucose) culture conditions, respectively. **A** High glucose enhances the promoting effect of FIBCD1 on cell proliferation in MCF7 cells. **B** Cell counting assay indicates that the effects of FIBCD1 on cell proliferation are stronger under high glucose conditions. **C** Proliferation of MCF7 cells by FIBCD1 was determined by cell counting assay. **D** Proliferation of MDA-MB-231 cells by FIBCD1 was measured by cell counting assay. **E** Hyperglycemia enhances the effects of FIBCD1 on cell migration ability of MCF7 cells. **F** Hyperglycemia increases the impacts of FIBCD1 on cell migration ability in MDA-MB-231 cells. **G** High glucose augments the promotive effects of FIBCD1 on cell invasion capacity of MCF7 cells. **H** High glucose promotes the influence of FIBCD1 on cell invasion ability of MDA-MB-231 cells. **I** Knockdown of FIBCD1 suppresses the proliferation rate of MAD-MB-231 cells. **J** Silencing of FIBCD1 inhibits proliferation capacity of MDA-MB-231 cells. **K** Knockdown of FIBCD1 depresses migration ability of MCF7 cells. **L** Silencing of FIBCD1 suppresses invasion capacity of MDA-MB-231 cells. Data are presented as mean ± SD, Student’s t test. *P < 0.05; **P < 0.01; ***P < 0.001.

To investigate the biological functions of hyperglycemia-induced FIBCD1, we cultured BC cells under euglycemic and hyperglycemia conditions, respectively. The CCK8 and cell counting assays consistently showed that overexpression of FIBCD1 increased cell proliferation in both MCF7 and MDA-MB-231 cells (Fig. 4A–D). Notably, these effects were stronger under hyperglycemia conditions. In agreement with results from cell proliferation assays, migration and invasion analyses revealed that hyperglycemic conditions increased the promotive effects of FIBCD1 on cell migration and invasion capacities in comparison with that under euglycemic conditions (Fig. 4E–H). In contrast, when FIBCD1 was silenced, the effects of hyperglycemia on cell proliferation, invasion, and migration of BC cells were partially reversed (Fig. 4I–L). Collectively, these findings indicate that the biological functions of FIBCD1 on BC cells were dependent on glucose levels.

### Knockdown of FIBCD1 inhibited BC tumor growth in diabetic mice

**Fig. 5:**
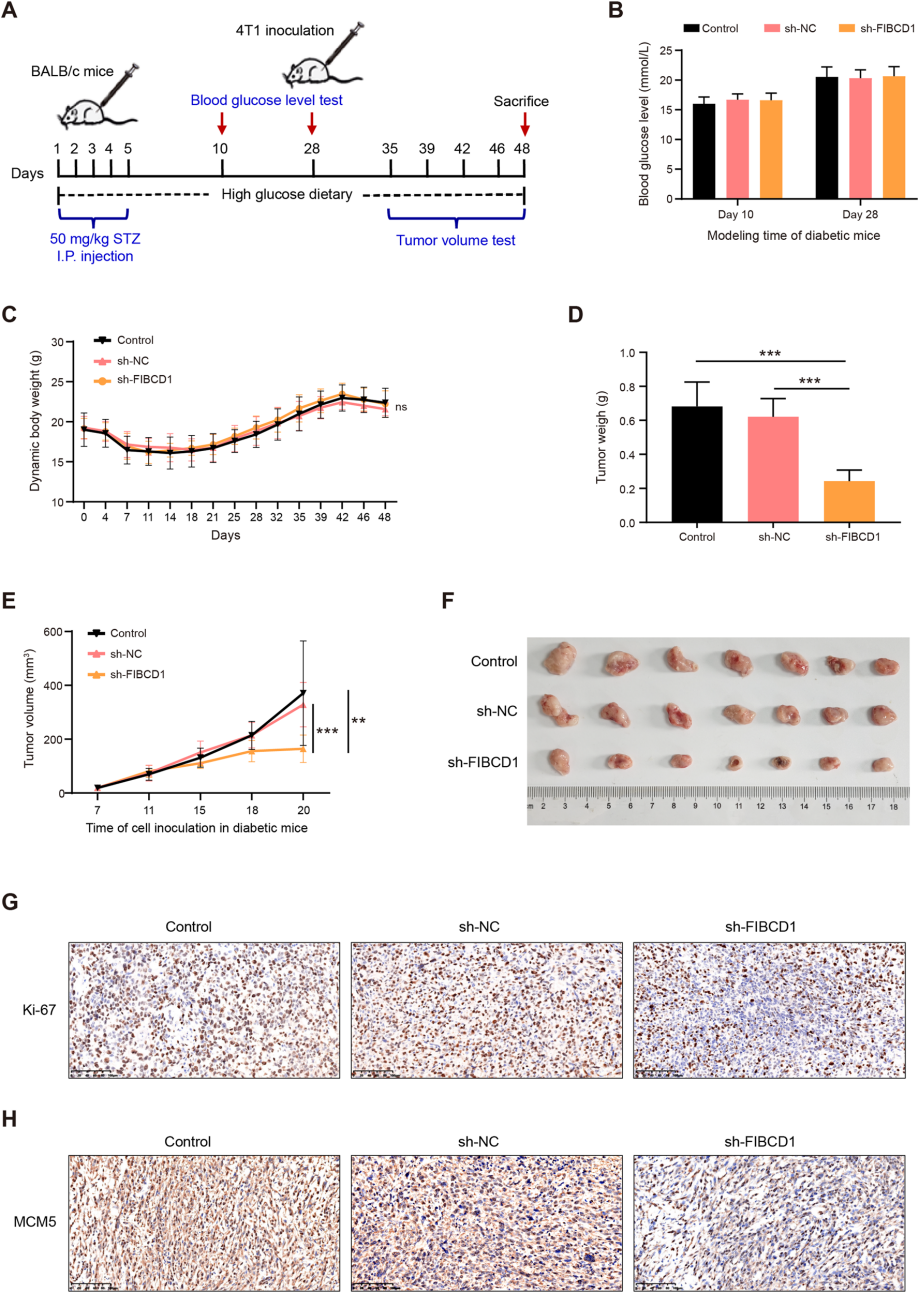
Knockdown of FIBCD1 inhibits BC tumor growth in diabetic mice. **A** Schematic overview of the in vivo experiment. **B** Blood glucose levels in sh-FIBCD1 treatment mice and control animals. **C** Comparison of body weight changes of mice during the experiment. **D**. The average xenograft tumor weight in si-FIBCD1-treated diabetic mice is significantly lower than that of control animals. **E** Tumor growth curves indicate that sh-FIBCD1 treatment significantly inhibits tumor growth in diabetic mice. **F** Representative images of xenograft tumors in diabetic mice with or without si-FIBCD1 treatment. **G** Representative images of IHC staining, indicating that sh-FIBCD1 suppresses the expression levels of Ki67 protein expression in xenograft tumor tissues. **H** sh-FIBCD1 inhibits MCM5 protein expression in xenograft tumor tissues. Data are presented as mean ± SD, Student’s t test. *P < 0.05; **P < 0.01; ***P < 0.001.

To examine the effect of FIBCD1 on BC cell growth in vivo under diabetic conditions, we constructed BC xenograft models in STZ-induced diabetic mice (Fig. 5A–C). The results showed that, compared with control cells, silencing of FIBCD1 decreased BC tumor weight and tumor volume growth under diabetic conditions (Fig. 5D–F). Moreover, IHC staining assay indicated that xenograft tumors tissues from FIBCD1-silencing group had lower level of Ki67 expression than that of NC group, indicating a lower proliferation index (Fig. 5G). In addition, the expression signals of MCM5 in FIBCD1-silencing tumors were also lower than that in NC groups (Fig. 5H), suggesting that inhibition of FIBCD1 suppressed MCM5 expression in vivo. These findings demonstrate that, under diabetic conditions, downregulation of FIBCD1 suppresses BC tumor growth and MCM5 expression in vivo.

### Integrative transcriptome and translatome analyses identified that cell cycle pathway gene MCM5 was a direct target of FIBCD1

**Fig. 6:**
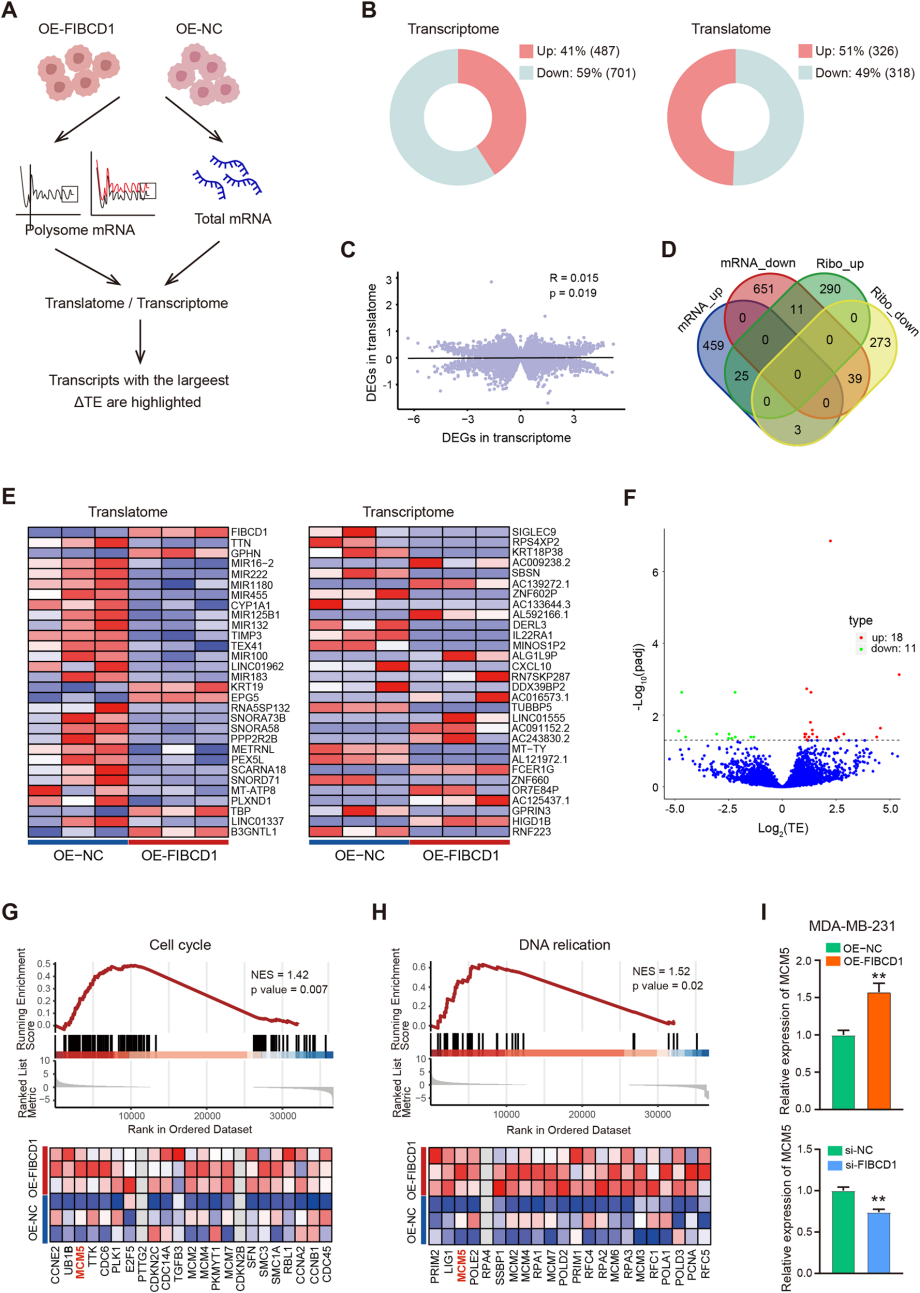
Integrated analysis of transcriptome and translatome identifies that cell cycle pathway gene MCM5 is a target of FIBCD1. **A** Schematic workflow showing RNA extraction from ribosome-protected mRNA fragments (RPFs) or from whole cell lysates in FIBCD1-overexpressing and control cells to be used for Ribo-seq or RNA-seq analyses. **B** Numbers of significantly up- or down-regulated transcripts shown as doughnuts. **C** Genome-wide transcriptional and translational regulations show very little correlation. **E** Heatmaps representing the top 30 up- or downregulated transcripts in the translatome and transcriptome with |fold change>2.0| and *P*-value < 0.05. **D** The four-way Venn diagram showing the overlap between the significantly upregulated or down-regulated transcripts in the translatome and transcriptome. **F** Volcano map of differentially expressed TE genes (DTEG). The blue dot represents the non-differentially expressed translation efficiency genes. **G** Gene set enrichment analysis (GSEA) of DTEG showing the enrichment of Cell cycle pathway in FIBCD1-overexpressing cells, with MCM5 as one of the top up-regulated genes. **H** GSEA analysis showing the enrichment of DNA replication pathway in FIBCD1-overexpressing cells, with MCM5 as one of the top up-regulated genes. **I** Overexpression/Knockdown of FIBCD1 promotes/inhibits MCM5 expression in MDA-MB-231 cells. Data are presented as mean ± SD, Student’s t test. *P < 0.05; **P < 0.01; ***P < 0.001.

To gain a deeper understanding of how FIBCD1 regulates the malignancy of BC cells, we performed paralleled RNA-seq and Ribo-seq analyses to search for the DEGs dysregulated by FIBCD1 at both transcription and translation levels (Fig. 6A). Compared with control cells, 1188 transcriptionally DE genes and 644 translationally DE genes (|fold change | ≧1.5 and *P* < 0.05) were identified in FIBCD1 overexpressing cells (Fig. 6B, Table S5-6). In agreement with previous reports [29, 30], there was a weak correlation between the DE transcripts in transcriptome and translatome (Spearman test, R = 0.015; Fig. 6C). Venn diagram and heatmap analyses also showed that there were little overlaps between the DE-genes of transcriptome and that of translatome (Fig. 6D, E). Further calculation of translation efficiency (TE = (RPKM from translatome) / (FPKM from transcriptome) [31] revealed that there were multiple differentially expressed TE genes in FIBCD1 overexpressing cells compared to that of control cells (Fig. 6F, Table S7). GSEA on differentially expressed TE genes showed that translationally dysregulated genes were related to cell cycle and DNA replication pathways. Among these dysregulated pathways, MCM5 gene was the key translationally dysregulated gene in both the cell cycle and DNA replication pathways (Fig. 6G, H). qRT-PCR assay confirmed that upregulation of FIBCD1 promoted MCM5 expression while knockdown of FIBCD1 inhibited MCM5 expression in BC cells (Fig. 6I). These results suggest that MCM5 is a potential target gene of FIBCD1.

**Fig. 7:**
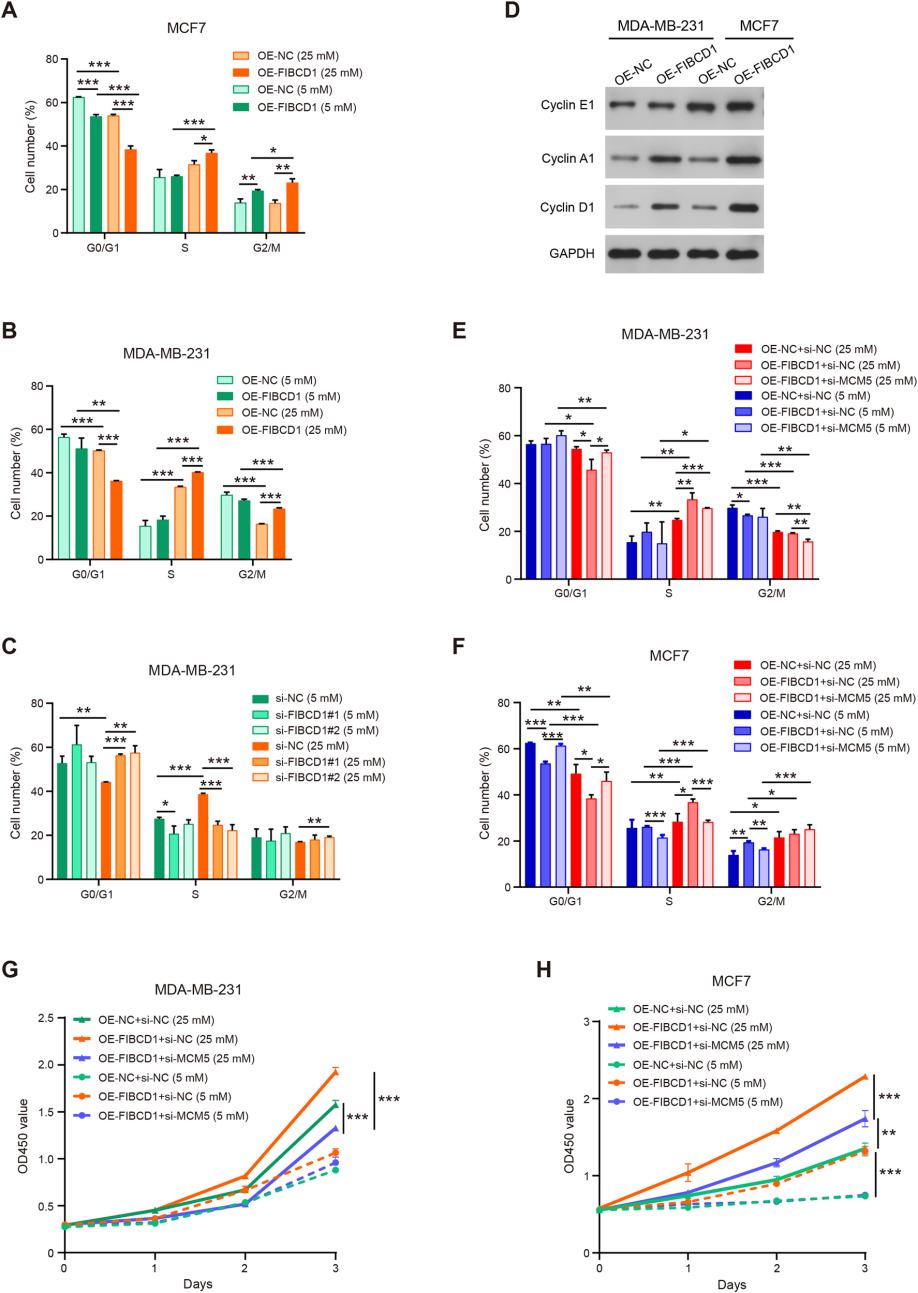
FIBCD1 affects BC cell cycle and proliferation by interacting with MCM5 in a glucose level-dependent manner. **A** Overexpression of FIBCD1 arrests MCF7 cells in the S and G2/M phases. **B** FIBCD1 induces S and G2/M phases arrest in MDA-MB-231 cells. **C** Knockdown of FIBCD1 reduces the percentage of S phase cells. **D** FIBCD1 promotes Cyclin A1 and Cyclin D1 protein expression in both MCF7 and MDA-MB-231 cells. **E** Co-transfection of FIBCD1 and si-MCM5 partly reverses the impact of FIBCD1 on S phase arrest of cell cycle in hyperglycemic compared with euglycemic conditions in MDA-MB-231 cells. **F** si-MCM5 partially abolishes the effect of FIBCD1 on the percentage of S phase cells in hyperglycemic compared with euglycemic conditions in MCF7 cells. **G** Co-transfection of FIBCD1 and si-MCM5 suppresses the influence of FIBCD1 on cell proliferation rate in hyperglycemic compared with euglycemic conditions in MDA-MB-231 cells. **H** si-MCM5 inhibits the promotive effects of FIBCD1 on cell proliferation in hyperglycemic compared with euglycemic conditions in MCF7 cells. Data are presented as mean ± SD, Student’s t test. *P < 0.05; **P < 0.01; ***P < 0.001.

Since FIBCD1 induced an enrichment of cell cycle pathway genes in BC cells, we further conducted cell cycle assay to examine the impact of FIBCD1 expression on cell cycle. The results indicated that overexpression of FIBCD1 decreased the number of G0/G1 phase cells but increased the percentage of the S phase cells. Notably, hyperglycemia significantly promoted cell cycle arrest in the S phase (Fig. 7A, B). On the contrary, knockdown of FIBCD1 led to a decrease of S phase cells in BC cells (Fig. 7C). Further, Western blot analysis demonstrated that upregulation of FIBCD1 increased the expression levels of cell cycle‑related protein Cyclin A1 and Cyclin D1 in both MDA-MB-231 and MAF7 cells (Fig. 7D). These findings indicate that FIBCD1 may promote BC cell proliferation by arresting cell cycle progression at the S phase.

### MCM5 is required for FIBCD1-induced malignancy in a glucose level-dependent fashion

To determine whether MCM5 may participate in the biological function of FIBCD1 in BC cells, we performed rescue experiments by co-transfecting FIBCD1 overexpression plasmids, si-MCM5, and their corresponding NCs into BC cells. Cell cycle assays showed that silencing of MCM5 partially rescued the effect FIBCD1-induced S phase arrest in MDA-MB-231 cells (Fig. 7E). Likewise, the impact of FIBCD1 on the rate of S phase of MCF7 cells decreased upon MCM5 downregulation (Fig. 7F). Importantly, the rescue effects of si-MCM5 on FIBCD1-mediated cell cycle dysregulation were stronger under hyperglycemia conditions than that of physiological culture conditions. In addition, CCK8 assay indicated that si-MCM5 significantly reversed the promotive effect of FIBCD1 on cell proliferation in both MCF7 and MDA-MB-231 cells (Fig. 7G, H). In summary, these results demonstrate that FIBCD1 promotes BC cell malignancy via interacting with MCM5.

### FIBCD1 increased H3K27Ac level in the promoter of MCM5 via the PDH/acetyl-CoA axis

**Fig. 8:**
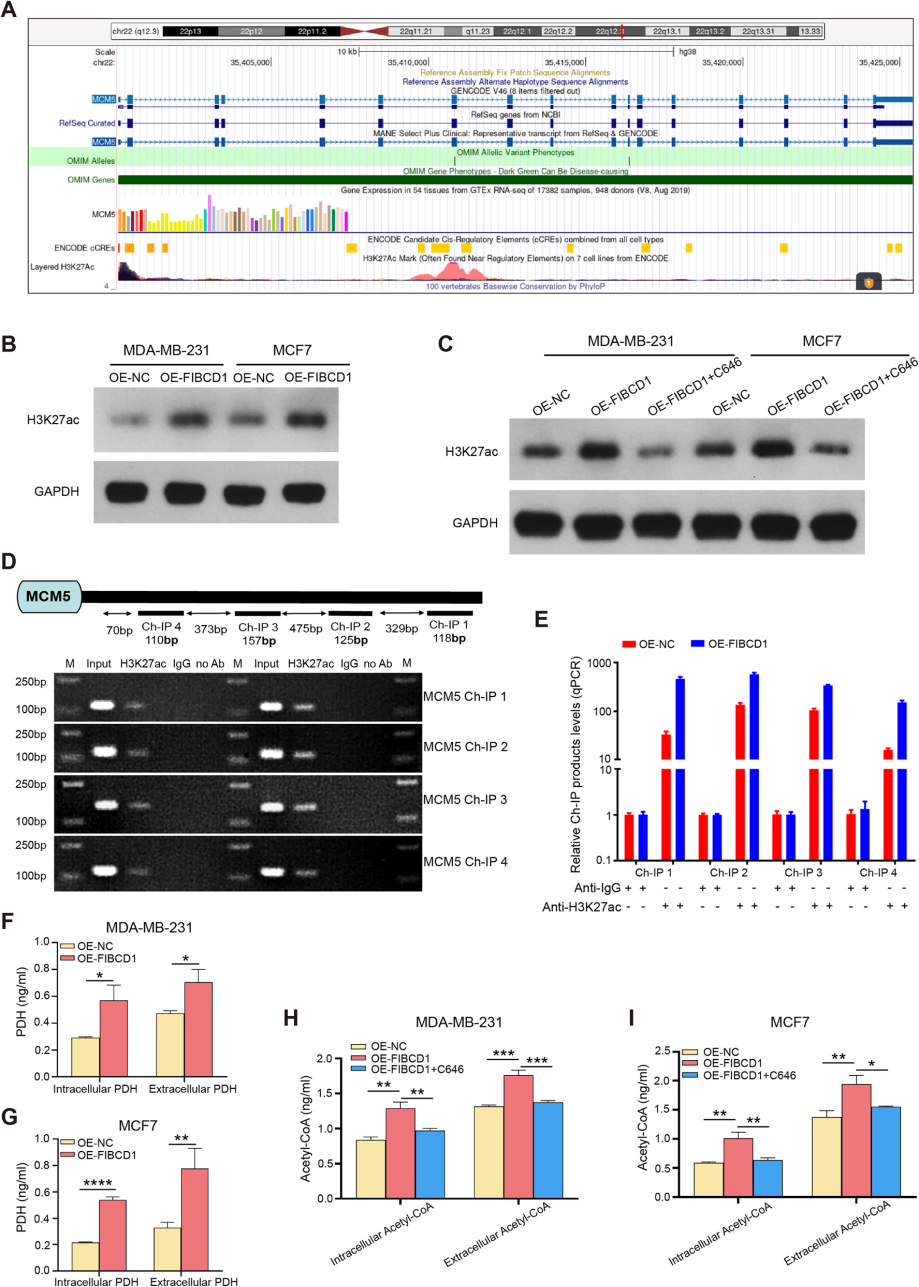
FIBCD1 promotes the enrichment of H3K27ac in the MCM5 promoter region through the PDH-acetyl-CoA axis. **A** UCSC genome browser views for the MCM5 gene, indicating that H3K27ac mark is enriched in the promoter of MCM5. **B** Upregulation of FIBCD1 increases H3K27Ac level in both MDA-MB-231 and MCF7 cells. **C** Histone acetylase inhibitor C646 inhibits the effect of FIBCD1 on H3K27ac in BC cells. **D** Schematic illustration of four potential binding sites (Ch-IP1, Ch-IP2, Ch-IP3, Ch-IP4) in the MCM5 promoter for H3K27ac (top). Agarose gel electrophoresis of PCR products indicates that FIBCD1 increases the binding of H3K27ac with MCM5 promoter (bottom). **E** ChIP assay shows that H3K27Ac is enriched in the promoter region of MCM5 in MDA-MB-231 cells. **F** Upregulation of FIBCD1 promotes PDH production from MDA-MB-231 cells. **G** Overexpression of FIBCD1 augments PDH production from MCF7 cells. **H** Upregulation of FIBCD1 facilitates acetyl-CoA generation and C646 inhibits acetyl-CoA production from MDA-MB-231 cells. **I** Overexpression of FIBCD1 increases the release of acetyl CoA but C646 suppresses acetyl CoA production from MCF7 cells. Data are presented as mean ± SD, Student’s t test. *P < 0.05; **P < 0.01; ***P < 0.001.

Previous studies have reported that the modification of histones leads to increased chromatin openness and upregulation of gene expression [32]. One of these modifications, the histone acetylation, requires the generation of acetyl-CoA [33]. Notably, pyruvate dehydrogenase (PDH) is a key enzyme that catalyzes pyruvate to produce acetyl-CoA [34]. To investigate whether FIBCD1 may affect MCM5 expression through regulating the histone acetylation pathway, we used UCSC (http://genome.ucsc.edu/) to predict the enrichment of histone modifications in the MCM5 promoter. As can be seen from Fig. 8A, the MCM5 promoter region has a higher abundance of H3K27Ac acetylation sites. Western blot assay showed that upregulation of FIBCD1 increased the levels of H3K27ac in both MDA-MB-231 and MCF7 cells (Fig. 8B). In contrast, treatment of BC cells with acetylation inhibitor C646 reversed the influence of FIBCD1 on H3K27ac levels (Fig. 8C). ChIP assay confirmed that FIBCD1-induced H3K27ac enrichment in the promoter region of MCM5 (Fig. 8D, E). Furthermore, upregulation of FIBCD1 enhanced PDH production in BC cells as well as in the cell culture medium (Fig. 8F, G). In addition, overexpression of FIBCD1 promoted intracellular and extracellular acetyl-CoA generation (Fig. 8H, I). Interestingly, the acetylation inhibitor C646 significantly inhibited FIBCD1-mediated acetyl-CoA production (Fig. 8H, I). Collectively, these results suggest that hyperglycemia-induced FIBCD1 activates production of PDH, which catalyzes the generation of acetyl-CoA, thus increasing H3K27ac level and promoting MCM5 expression.

## Discussion

The presence of DM among patients with BC is well recognized, and previous epidemiological studies have explored the associations of DM with BC prognosis. However, no studies have investigated the causal impact of DM on BC prognosis in BC-DM, and none have investigated the genetic mediators of this syndrome [10–13, 18]. In this study, we sought to clarify the causal impact of DM on overall mortality of BC-DM patients and attempted to elucidate the underlying mechanism. Our integrated causal inference analyses clearly demonstrated that BC was causatively associated with increased BC mortality, even after adjusting for linear- and non-linear contributions. We revealed that transcriptome profiles in BC-DM were distinct from that of BC-ND. In addition, we identified that FIBCD1 was a BC-DM associated oncogene, which enhanced the malignancy of BC cells in a glucose-dependent manner. Furthermore, we demonstrated that FIBCD1 facilitated BC cell malignancy by enhancing MCM5 expression to modulate cell cycle through the PDH-Acetyl-CoA-H3K27ac axis. To the best of our knowledge, this is the first study to reveal the causal relationship between DM and BC prognosis and to elucidate the molecular mechanism underlying BC-DM. Our findings not only define the causal effect of DM on BC progression but also demonstrate that FIBCD1 is a key regulator of BC-DM and a potential therapeutic target of BC-DM.

Since BC and DM are two of the most prevalent diseases in the world and up to one-third of BC patients are BC-DM, if considered as a separate category, BC-DM would rank as one of the most common cancers in the world. Clarifying the causal impact of DM on BC outcome thus has great public health significance. Nevertheless, most previous studies used conventional analyzing approaches that suffered from inherent bias and limitations in dealing with non-linear associations. Therefore, literature on the association between DM and BC prognosis is contradictory. The present study comprehensively evaluated the causal relationship between DM and BC outcome, utilizing multiple causal inference methods, including IPTW, TMLE, TMLE-SL, GC, and sensitivity analyses to ensure robust results. Compared with Cox regression model, the IPTW uses the propensity score to balance baseline patient characteristics in the exposed and unexposed groups via weighting everyone in the analysis by the inverse probability of receiving his/her actual exposure [35]. TMLE is a semiparametric, doubly robust approach for estimating marginal causal effects. TMLE coupled with Super Learner (SL) algorithm can reduce bias and improve statistical efficiency [23]. GC is a simulation-based algorithm that provides flexibility to choose models and variables without considering an analytic form [36]. Therefore, GC is particularly useful for investigating nonlinear associations between exposure and outcomes [37]. Moreover, the IPT-weighted analysis used in IPTW and TMLE reduced the selection bias and eliminated the potential confounding factors. In this study, we combined the strengths of these four causal inference algorithms to estimate the causal effect of DM on BC outcome. These cohesive approaches improve the statistical power of our analysis and allow us to have less bias to estimate both linear and nonlinear risk factors for BC mortality. Clarifying this relationship will help to develop more accurate treatment plans and guidelines, as well as better understand the role of DM in BC clinical management.

To obtain valid causal interpretations for estimated exposure effects, three foundational assumptions must be satisfied: (a). stable unit treatment value assumption (SUTVA); (b). positivity; (c). no unmeasured confounding. In this study, a subject’s survival outcome is not affected by another subject’s diabetes status, and the definition of diabetes is consistent across all subjects, with no variation in how diabetes influences the survival outcome. Hence, the SUTVA assumption is satisfied. Regarding the positivity assumption, we used the effect sample size (ESS) associated with each treatment level to assess common support. A high ratio of ESS to the sample size suggests similarity in the estimated propensity scores and sufficient overlap among treatment groups. ESS analysis showed that both IPTW and TMLE + SL methods gave high ratios of ESS to the sample size, suggesting adequate overlap before weighting (Table S9). Further adjustment for multiple covariates significantly improved covariate balance (Figure S1). To examine the sensitivity of our effect estimates (OR) to unmeasured confounding, we implemented the confounding function framework. The confounding function assesses potential violations of the no unmeasured confounding assumption, including the presence, direction and magnitude. Contour plot analysis showed that the estimates of OR were insensitive to various magnitudes of departure from the ignobility assumption (Figure S2).

Little research has been conducted to elucidate the molecular mechanisms underlying the pathogenesis of BC-DM. Here, we found a large number of DEGs between BC-DM and BC-ND tumor tissues, suggesting that BC-DM is a genetically subtype distinct from that of BC-ND. In particular, we identified and validated that FIBCD1 was a key BM-CD-associated gene. The FIBCD1 is a protein belonging to the fibrinogen‑related domain (FReD) superfamily [38]. The FIBCD1 protein is an acetyl receptor that combines with the acetyl sites of chitin at the FReD [39]. FIBCD1 protein also contains a binding domain for carbohydrate [32], suggesting that FIBCD1 may be involved in glucose-associated biological functions. FIBCD1 has been found to be upregulated in gastric cancer and hepatocellular carcinoma and is associated with poor prognosis in patients with these cancers [38, 40]. Nevertheless, whether FIBCD1 may play a role in BC is unknown. Here, we showed that FIBCD1 was upregulated in BC-DM and under hyperglycemia conditions. Moreover, we found that FIBCD1 promoted the malignancy of BC cells in a glucose level-dependent manner. Importantly, we demonstrated that inhibition of FIBCD1 could suppress BC tumor growth in diabetic mice. To our knowledge, this is the first report on the biological functions and regulatory mechanisms of FIBCD1 in BC cells. Our research suggests that FIBCD1 is an oncogene in BC-DM and targeting FIBCD1 could be a potential therapeutic approach for BC-DM treatment.

Integrative RNA-seq and Ribo-seq analysis identified that MCM5 was a target gene of FIBCD1 in BC cells. The MCM5 gene encodes a DNA replication regulatory protein essential for DNA replication initiation and elongation in eukaryotic cells [41]. MCM5 is implicated in cell cycle regulation, cell proliferation, and is upregulated during the transition from G0 to G1/S phase [42, 43]. Previous studies showed that MCM5 was upregulated in cervical and ovarian cancers and was associated with their prognosis [44, 45]. In lung cancer, MCM5 interacted with histone deacetylase 1 (HDAC1) to aggravate cancer progression [46]. But the effect of MCM5 on BC cells has not been reported. Histone acetylation, particularly H3K27Ac modification, is essential for increased chromatin openness and upregulation of gene expression [47]. It has been shown that histone acetylation requires acetyl-CoA [33, 48], which is produced from pyruvate via the catalyzation of enzyme PDH [34]. In this study, we found that hyperglycemia upregulated the expression of FIBCD1, which activated MCM5 expression to affect cell cycle and proliferation via the PDH-acetyl-CoA axis. Importantly, histone acetylation inhibitor C646 could reverse the effect of FIBCD1 on the H3K27Ac enrichment in MCM5 promoter. These results indicate that hyperglycemia-induced FIBCD1-PDK-acetyl-CoA-H3K27ac signaling pathway plays a critical role in BC-DM. Targeting this pathway is expected to become a promising therapeutic approach for BC-DM.

Our study had several strengths. First, we used causal inference methods to assess the relationship between DM and BC progression. These approaches reduced the covariate imbalance by simulating randomized trials. Second, diabetes was defined by fasting plasma glucose (FPG), rather than by self-reported diabetes. Indeed, most previous observational studies defined diabetes by self-reports, which could be subject to recall bias or physician misdiagnosis. It has been reported that the sensitivity of self-reported diabetes was only 66% compared with medical records [49], and up to 60% of BC patients who had diabetes did not know about having diabetes [50]. Identification of diabetes based on FPG level minimized classification bias. Third, we employed causal inference and molecular analyses to identify and validate BC-DM-associated FIBCD1, this integrated approach allowed us to link molecular mechanisms to epidemiological findings. Our study also had several limitations. First, the patients in this study were from a single center. However, all BC patients in this study are the Han nationality, which accounts for ~92% of Chinese population. Moreover, patients in this study were from four provinces with >60 million peoples, which was good representative for Chinese population. Further, the clinical characteristics of our cohort are comparable to other Chinese breast cancer cohorts (Table S9). In addition, the prevalence of DM in our BC cohort was 8.86%, which was comparable to previous report in which BC-DM rate in Asian BC patients was 7.7% [27]. Thus, BC cohort in this study was most likely a good representativeness of Chinese BC patients. However, the results of this study may not be generalized to other ethnic populations since gene expression alterations could vary between ethnic groups. Additional studies are required to clarify the association between the FIBCD1 and BC-DM outcome in diverse ethnic populations. Second, adjuvant chemotherapy was not selected as candidate factors because adjuvant therapies were recommended for only a small proportion of patients. Third, although we demonstrated that both si-FIBCD1 and sh-FIBCD1 were strong inhibitors of BC cell malignancy, we could not exclude the possibility of off-target effects, where si/shRNA might unintentionally silence non-targeted transcripts with partial sequence complementarity. Future studies incorporating chemical modifications to prolong the siRNA half-life, together with specific nano- delivery techniques, may help to mitigate potential off-target effects [51]. Fourth, tissue samples for FIBCD1 analysis were relatively small and may be affected by potential batch effect. However, the clinicopathologic features of molecular samples and epidemiological samples were comparable (Table S11). Future molecular epidemiological studies in larger BC populations are necessary to better determine the clinical significance of FIBCD1 in BC-DM.

In conclusion, our study provided important evidence that DM was a causal risk for all-cause mortality in China BC patients. Furthermore, we identified a novel BC-DM-associated oncogene FIBCD1, which enhanced the malignancy of BC cells in a glucose level-dependent manner by promoting MCM5 expression through regulating histone acetylation modification in the MCM5 promoter. Future therapeutic studies are needed to develop optimal treatment approaches for patients with concurrent BC and DM.

## Materials and Methods

### Cohort of BC patients

Patients with breast cancer (BC) included in this study were recruited from consecutive patients who received surgical resection as their initial treatment at Cancer Hospital of Habin Medical University between 2007 and 2017. The inclusion criteria for this study were as followings: (a) female of the Han nationality; (b) age ≥18 years; (c) pathologically confirmed as BC; (d) received surgical treatment; (e) preoperative biological test data can be obtained; (f) availability of follow-up information. Patients with the following characteristics were excluded: (a) with previous or co-existing cancers other than BC; (b) confirmed metastasis at the first visit; (c) having received any other treatment prior to surgery; (d) not enough follow-up data can be obtained. None of the patients had received radiotherapy or chemotherapy before surgery. Written informed consent was obtained from each study patient. This study was approved by the Ethics Committee of Shenzhen University School of Medicine (approved #2019015).

### Clinical variables

Surgical pathologists specializing in breast pathology examined the pathological specimens of all patients. The histological type, grade, and TNM-stage were classified according to the American Joint Committee on Cancer (AJCC)TNM staging system (7^th^ edition) [52]. Clinicopathologic data on age, gender, menstrual status, menarche age, tumor location, TNM stage, tumor size, estrogen receptor (ER), progesterone receptor (PR), and HER-2 status were retrospectively extracted from electronic medical record system of the hospital. Pre-operation BMI was calculated (BMI = weight (kg)/height (m)^2^). All patients in this study were followed regularly once every 6 months during the first year and then annually thereafter. Survival time was defined as the duration from the date of diagnosis to last follow-up or death due to all causes (overall survival, OS). All patients were follow-up to date of death or December 30 of 2018.

### Assessment of T2DM

Fasting plasma glucose (FPG) level measured before operation was used as the main diagnostic factor of study interest. A venous blood sample was taken from every patient before operation under fasting conditions, defined as 12 h from last meal. FPG level was analyzed within 4 hours of blood collection. DM was defined as having FPG level ≧7.0 mmol/l, according to the criteria of American Diabetes Association (ADA). (2016) [53]. Patients with FPG level <7.0 mmol/L but with medical records of physician-diagnosed T2DM were also classified as having DM.

### Statistical analysis

Continuous variables were presented as mean and standard deviation (SD), or median and interquartile range (IQR), depending on the results of the Shapiro-Wilk test for normality. The homogeneity of variances for t-tests were examined by Levene’s Test. Categorical variables were presented as frequencies and percentages. For the univariate analysis, differences between the comparative groups were assessed with the chi-square test or Fisher’s exact test for categorical variables. Comparison between continuous variables were carried out by the student’s t-test or Mann–Whitney U test, depending on the normality test results. In the case of multiple comparisons, we adjust the *P*-values by the Benjamini and Hochberg method to control the false discovery rate at 5%. All statistical analyses were conducted with R 4.3.3 software (R-Project, Vienna, Austria). Statistical significance was determined by *P* < 0.05 and all *P*-values were two-sided.

### Causal effect modeling

In this study, we aim to estimate the 5-year odds ratio of death for BC patients with DM (BC-DM) versus those without DM (BC-ND). Let Y be the mortality indicator (1 death, 0 alive), and A denote the diabetes indicator (1 diabetes, 0 non-diabetes). We consider the following possible confounders: age, BMI, KI67, P53, stage, HER2, ER, PR, lymphatic_metastasis, hypertension, and menopause status. The causal estimate of interest is the marginal odds ratio (OR):

$${\rm{E}}[{\rm{Y}}(1)]\times \{1-{\rm{E}}[{\rm{Y}}(0)]\}/\{{\rm{E}}(1-{\rm{E}}[{\rm{Y}}(1)])\times {\rm{E}}[{\rm{Y}}(0)]\}$$

We utilized four methods to estimate the OR: G-computation (GC), inverse probability of treatment weighting (IPTW), targeted maximum likelihood estimation (TMLE) and TMLE with super-learner (TMLE-SL). GC and IPTW are well-established techniques for causal inference in observational studies. GC estimates the causal effect of exposure by modeling the outcome as function of exposure assignment mechanism and confounders. The IPTW is based on propensity score to model the exposure assignment mechanism. Both GC and IPTW methods are sensitive to model misspecification [54]. In contrast, the TMLE is a double-robust estimation method. It provides consistent estimator of the causal effect as long as at least one of the exposure models or the outcome model is correctly specified. In addition, TMLE can incorporate machine learning algorithms in generating both the exposure and outcome models. For TMLE-SL, the ensemble machine learning technique, Super learner was used to construct both the exposure and outcome models [55], 15-fold cross validation to determine the optimal linear combination of the following machine learning algorithm: random forest, k-nearest neighbors (kNN), kernel support vector machines (ksvm), elastic net (glmnet), gradient boosting machines (gbm), and extreme gradient boosting (xgboost). The TMLE + SL has been shown to perform well when handling high-dimensional, complex observational data [56].

### RNA sequencing on tumor tissues of BC-DM and BC-ND

Total RNA was isolated from tissue sample using TRIzol reagent (Invitrogen, USA) and then purified using the RNeasy mini kit (Qiagen, USA) according to the manufacturer’s protocol. mRNA was enriched using the NEBNext® Poly(A) mRNA Magnetic Isolation Module (New England Biolabs, USA). RNA-seq libraries were prepared using KAPA Stranded RNA-Seq Library Prep Kit (Illumina, USA) and then sequenced on Illumina X-ten/NovaSeq by the Aksomics Inc. (Shanghai, China). Sequencing reads were mapped to human genome database (GRCh37) using the StringTie software. Fragments per kilobase of transcript per million mapped reads (FPKM) were used to calculate the gene expression level. Kyoto Encyclopedia of Genes and Genomes (KEGG), Gene Ontology (GO), and Gene Set Enrichment Analysis (GSEA) were used for gene functional annotation. The sequencing data has been deposited in CNSA database (accession number: CNP0007369).

### Cell culture

Human breast cancer cell lines (MCF7 and MDA-MB-231) were obtained from the Cell Bank of Type Culture Collection of Chinese Academy of Sciences (Shanghai, China). The identity of all cell lines was verified by the short tandem repeat (STR) assay, and the absence of mycoplasma contamination was examined by ELISA technique. BC cell lines were cultured in Dulbecco’s modified Eagle media (DMEM, Gibco) containing 10% fetal bovine serum (FBS, Gibco, 10091155) and 1% penicillin/streptomycin antibiotic (PS, Gibco, 15140163) in a 5% CO_2_ incubator at 37 °C. Cells were maintained in humidified atmosphere of CO_2_ (5%) at 37 ^o^C in medium containing normal physiological levels of glucose (5 mM) or mimic hyperglycemia (25 mM) [57]. All cell culture experiments were tested in triplicates.

### Plasmid construct, siRNA, and cell transfection

FIBCD1 over-expressed plasmids (pTSB-CMV-FIBCD1) and control plasmids (pTSB-CMV-NC) were constructed by Shuangquan Biotech Co. (Guangzhou, China). Scrambled siRNA and corresponding normal controls (NCs) were ordered from RiboBio Co. (Guangzhou, China). Transfection was performed using the Lipofectamine® 3000 Reagent (L3000015, Thermo-Fisher, USA) according to the manufacturer’s instruction.

### Quantitative Real-time PCR (qRT-PCR)

Total RNA was extracted using Total RNA extraction kit (TaKaRa, cat#9767). Reverse transcription was performed using the PrimeScriptTM RT reagent Kit (TaKaRa, cat#RR047Q). Synthesized cDNA was used for qRT-PCR analysis using Bio-Rad CFX96TM System (Bio-Rad, USA). Relative mRNA levels of targeted genes were analyzed using the 2^-ΔΔCt^ method. The primer sequences are presented in Table S8.

### Western blot

Total cellular proteins were extracted by the RIPA lysis buffer (Solarbio, China, cat#R0010). The protein concentration was measured with BCA reagent (Thermo Fisher, cat#23227). Western blot analysis was performed using standard procedures. The following primary antibodies were used in the experiments: anti-FIBCD1 (Novus, USA), Anti-β-actin (Cell Signaling, USA), anti-Cyclin E1 (Proteintech), anti-Cyclin D1 (Abcam), Cyclin A1 (Abcam), anti-H3K27ac (Abcam). Full and uncropped images of western blots are included in supplementary materials.

### Cell proliferation

Cells growth was quantified using the CCK-8 assay (Dojindo, cat#CK04) according to the manufacturer’s instructions. Briefly, cells were plated in 96-well plates (1 × 10^3^ cells/well) and incubated with 100 μL of medium overnight. Then 10 µL of CCK-8 solution were added to cultured cells in each well, followed by incubation at 37 °C for 1 h. The OD values were measured at 450 nm using a microplate reader.

### Wound-healing assay

Cells were plated in 6-well plates in medium containing 10% FBS. After total confluence (usually 48 h after seeding), 10 μg/mL mitomycin C was added and cells were treated for 3 h. Then, a micropipette tip was used to make a wound on the monolayer. Subsequently, DMEM culture medium with different concentrations of glucose (5 mM or 25 mM) was replaced and incubated for 24 h. The wound was imaged at 0 h and 24 h and the width of the scratch was calculated under a microscope (Zeiss AxioVision AX10).

### Cell migration assay

Cells were cultured in serum-free medium for 24 h before performing migration and invasion assays. Cells in 0.5 mL of serum-free medium were seeded in the upper chamber (Corning, cat#353097) with or without 40 μL of 1 mg/ml Matrigel (BD, cat#356234), and 0.7 mL of medium containing 10% FBS was added to the lower chamber. After 24 h, the cells on the top of the membrane were removed with cotton swabs. Cells that had migrated/invaded to the bottom well were fixed with 4% paraformaldehyde for 10 min and stained with a 0.5% crystal violet (Sigma-Aldrich, cat#548-62-9) solution for 15 ~ 30 min. The number of migrating/invading cells was counted in three randomly selected light microscopy fields (Zeiss AxioVision AX10) by Image-J software.

### Invasion assay

Prior to the experiment, 60 μL diluted matrigel (1:30 in serum-free medium) was applied to the upper surface of transwell inserts (Corning, cat#353097) and allowed to solidify at 37°C for 2 h. Cells in 0.5 mL of serum-free medium were seeded in the upper chamber (Corning, cat# 353097), and 0.7 mL of complete medium containing 10% FBS was added to the lower chamber. After 48 hours of incubation, the chamber was removed, and the cells on the upper surface were carefully wiped off with a cotton swab. The cells were then fixed with methanol and stained with 0.5% crystal violet (Sigma-Aldrich, 548-62-9) solution for 30 min. The number of invading cells was counted in three randomly selected light microscopy fields (Zeiss AxioVision AX10) by Image-J software.

### Cell cycle analysis

Cells were plated in 6-well plates in medium containing 10% FBS. After about 90% confluence, the adherent cells were digested and centrifuged at 500 g for 5 min. Subsequently, the cells were washed in pre-chilled PBS and fixed in 75% ethanol at 4 °C overnight. Then, the cells were centrifuged and then resuspended in PBS. After filtering with a 300-mesh nylon mesh, cell cycle distribution was determined by a flow cytometry (Beckman CytoFLEX, U.S.A.). Results were presented as percentage cells in each phase of cell cycle in relation to total number of cells counted.

### Diabetic breast cancer animal model

BalB/C mice were purchased from the Slac Animal Center (Shanghai, China). Sh-FIBCD1 and its NC were synthesized and cloned into pMKO.1-puro (RRID: Addgene_8452) vector by Yanjiang Bios (Shanghai, China) to construct stably expressed sh-FIBCD1 or NC 4T1 cells (BCRJ Cat#: 0022, RRID: CVCL_0125). To establish diabetic model, mice were fed with high fat diet (Readydietech, D1249, Shenzhen, China) for the entire experimental period (48 days). The sample size of animals was estimated according to previous reports [58]. Glycemic levels were measured on whole blood obtained from the mouse tail using a Accu-Chek glucose meter (Roche, Germany). STZ (streptozotocin, once 50 mg kg−1 for 5 days) was injected intraperitoneally to induce diabetes and diabetes was confirmed by the presence of hyperglycemia (>11.1 mmol). After the establishment of diabetes, mice were divided into 3 groups (n = 8 for each group) by a random number table and the investigator was blinded to the group allocation during the experiments. Then, sh-FIBCD1-4T1, NC-4T1, and control cells were injected into the right flanks of diabetic mice, respectively. Mice were followed with measurements of blood glucose levels and body weight. The equation of volume = (length × width^2^)/2 was used to calculate tumor volumes. All animal study procedures were performed in accordance with the guidelines approved by the Institutional Animal Care and Use Committee of Shenzhen University Medical School (Approved No. IACUC-202300060).

### Immunohistochemistry (IHC) staining

IHC staining was performed as previously described [59]. Briefly, tissue slides were incubated with anti-Ki67 (Proteintech), anti-FIBCD1 (Invitrogen), or anti-MCM5 (CST, USA) antibodies, followed by the IgG-horseradish peroxidase-conjugated secondary antibody. After DAB staining, the slides were analyzed according to the staining intensity and distribution of stained cells.

### Parallel Ribo-seq and RNA-seq on BC cells

Cell samples were treated with a specific lysis buffer containing cycloheximide (50 mg/mL). Lysates were digested with unspecific endoribonuclease RNase ǀ to remove the RNA other than the ribosome-protected fragments (RPFs). RPFs were isolated by size-exclusion chromatography using the MicroSpin S-400 HR columns (GE Healthcare). rRNAs were removed using the rRNA depletion kit. Purified RPFs were phosphorylated and ligated with 5’ and 3’ adapters respectively. Then the fragments were reversely transcribed to the cDNAs and amplified by PCR. Library samples were sequenced (Paired-end 150nt) on Illumina PE150 platform. Reads were mapped to human genome database using TopHat2, and differential expression was determined using HTSeq and DESeq2 R package.

RNA-seq was performed with the same cultures as used for the Ribo-seq analyses. Total RNA was extracted using TRIzol reagent. Library preparation was conducted using the NEBNext® Ultra™ RNA Library Prep Kit for Illumina® in the sequencing facility at the Novogene Biotech (Beijing, China), and cDNA was sequenced on an Illumina Novaseq platform and 150 bp paired-end reads were generated. Index of the reference genome was built using Hisat2 v2.0.5 and paired-end clean reads were aligned to the reference genome using Hisat2 v2.0.5. FeatureCounts v1.5.0-p3 was used to count the reads that mapped to each gene. FPKM (Fragments Per Kilobase of transcript sequence per Millions base pairs) of each gene was calculated using the DESeq2 R package (1.20.0).

### Chromatin immunoprecipitation (ChIP)

ChIP assay was conducted to determine the enrichment of H3K27Ac in the MCM5 promoter of MDA-MB-231 cells, using the Pierce Agarose ChIP Kit (Thermo Fisher, cat#26156) was used. Anti-H3K27Ac (Abcam, Ab4729) was used in the ChIP assay, and goat anti-rabbit IgG (Cell Signaling Technology) was used as a negative control.

### Measurement of PDH and acetyl-CoA production

After transfection for 24 h, the cells were ultrasonicated on ice. Then, the supernatant was separated by centrifugation. Finally, PDH activity and concentrations of acetyl-CoA were measured using the pyruvate dehydrogenase (PDH) activity assay kit (Solarbio, Beijing, China), acetyl coenzyme A assay kit (Nanjing Jiancheng Bioengineering, China). The results were normalized by protein concentration.

## Supplementary information

Supplementary materials

Supplementary materials

Supplementary materials

## Data Availability

All other data and materials supporting our findings can be made available from the corresponding authors upon request.

## References

[1] Sung H, Ferlay J, Siegel RL, Laversanne M, Soerjomataram I, Jemal A, et al. Global Cancer Statistics 2020: GLOBOCAN Estimates of Incidence and Mortality Worldwide for 36 Cancers in 185 Countries. CA Cancer J Clin. 2021;71:209–49. 33538338 10.3322/caac.21660

[2] Yancik R, Wesley MN, Ries LA, Havlik RJ, Edwards BK, Yates JW. Effect of age and comorbidity in postmenopausal breast cancer patients aged 55 years and older. JAMA. 2001;285:885–92. 11180731 10.1001/jama.285.7.885

[3] Srokowski TP, Fang S, Hortobagyi GN, Giordano SH. Impact of diabetes mellitus on complications and outcomes of adjuvant chemotherapy in older patients with breast cancer. J Clin Oncol. 2009;27:2170–6. 19307509 10.1200/JCO.2008.17.5935 PMC2674004

[4] Tammemagi CM, Nerenz D, Neslund-Dudas C, Feldkamp C, Nathanson D. Comorbidity and survival disparities among black and white patients with breast cancer. JAMA. 2005;294:1765–72. 16219879 10.1001/jama.294.14.1765

[5] Shao S, Gill AA, Zahm SH, Jatoi I, Shriver CD, McGlynn KA, et al. Diabetes and Overall Survival among Breast Cancer Patients in the U.S. Military Health System. Cancer Epidemiol Biomark Prev. 2018;27:50–57. 10.1158/1055-9965.EPI-17-0439 PMC5808886 29097445

[6] Maskarinec G, Shvetsov YB, Conroy SM, Haiman CA, Setiawan VW, Le Marchand L. Type 2 diabetes as a predictor of survival among breast cancer patients: the multiethnic cohort. Breast Cancer Res Treat. 2019;173:637–45. 30367331 10.1007/s10549-018-5025-2 PMC6391188

[7] Boyle P, Boniol M, Koechlin A, Robertson C, Valentini F, Coppens K, et al. Diabetes and breast cancer risk: a meta-analysis. Br J Cancer. 2012;107:1608–17. 22996614 10.1038/bjc.2012.414 PMC3493760

[8] Liao S, Li J, Wei W, Wang L, Zhang Y, Li J, et al. Association between diabetes mellitus and breast cancer risk: a meta-analysis of the literature. Asian Pac J Cancer Prev. 2011;12:1061–5. 21790252

[9] Hardefeldt PJ, Edirimanne S, Eslick GD. Diabetes increases the risk of breast cancer: a meta-analysis. Endocr Relat Cance r. 2012;19:793–803. 10.1530/ERC-12-0242 23035011

[10] Barone BB, Yeh HC, Snyder CF, Peairs KS, Stein KB, Derr RL, et al. Long-term all-cause mortality in cancer patients with preexisting diabetes mellitus: a systematic review and meta-analysis. JAMA. 2008;300:2754–64. 19088353 10.1001/jama.2008.824 PMC3093051

[11] Zhao XB, Ren GS. Diabetes mellitus and prognosis in women with breast cancer: A systematic review and meta-analysis. Medicine (Baltim). 2016;95:e5602. 10.1097/MD.0000000000005602 PMC5266055 27930583

[12] Zhou Y, Zhang X, Gu C, Xia J. Influence of diabetes mellitus on mortality in breast cancer patients. ANZ J Surg. 2015;85:972–8. 25312511 10.1111/ans.12877

[13] Fernández-Arce L, Robles-Rodríguez N, Fernández-Feito A, Llaneza-Folgueras A, Encinas-Muñiz AI, Lana A. Type 2 Diabetes and all-cause mortality among Spanish women with breast cancer. Cancer Causes Control. 2022;33:271–8. 34853980 10.1007/s10552-021-01526-x PMC8776668

[14] Du W, Simon MS. Racial disparities in treatment and survival of women with stage I-III breast cancer at a large academic medical center in metropolitan Detroit. Breast Cancer Res Treat. 2005;91:243–8. 15952057 10.1007/s10549-005-0324-9

[15] Jiralerspong S, Kim ES, Dong W, Feng L, Hortobagyi GN, Giordano SH. Obesity, diabetes, and survival outcomes in a large cohort of early-stage breast cancer patients. Ann Oncol. 2013;24:2506–14. 23793035 10.1093/annonc/mdt224 PMC3784334

[16] Luo J, Virnig B, Hendryx M, Wen S, Chelebowski R, Chen C, et al. Diabetes, diabetes treatment and breast cancer prognosis. Breast Cancer Res Treat. 2014;148:153–62. 25261292 10.1007/s10549-014-3146-9 PMC4393950

[17] Calip GS, Yu O, Hoskins KF, Boudreau DM. Associations between diabetes medication use and risk of second breast cancer events and mortality. Cancer Causes Control. 2015;26:1065–77. 25956271 10.1007/s10552-015-0599-z PMC4501774

[18] Lohmann AE, Goodwin PJ. Diabetes, metformin and breast cancer: a tangled web. Ann Oncol. 2021;32:285–6. 33516777 10.1016/j.annonc.2020.12.014

[19] Bind MA. Causal Modeling in Environmental Health. Annu Rev Public Health. 2019;40:23–43. 30633715 10.1146/annurev-publhealth-040218-044048 PMC6445691

[20] Matsuo K, Purushotham S, Jiang B, Mandelbaum RS, Takiuchi T, Liu Y, et al. Survival outcome prediction in cervical cancer: Cox models vs deep-learning model. Am J Obstet Gynecol. 2019;220:381.e381–381.e314. 10.1016/j.ajog.2018.12.030 PMC7526040 30582927

[21] Peng J, Lu Y, Chen L, Qiu K, Chen F, Liu J, et al. The prognostic value of machine learning techniques versus cox regression model for head and neck cancer. Methods. 2022;205:123–32. 35798257 10.1016/j.ymeth.2022.07.001

[22] Wang Y, Kloog I, Coull BA, Kosheleva A, Zanobetti A, Schwartz JD. Estimating Causal Effects of Long-Term PM2.5 Exposure on Mortality in New Jersey. Environ Health Perspect. 2016;124:1182–8. 27082965 10.1289/ehp.1409671 PMC4977041

[23] Schuler MS, Rose S. Targeted Maximum Likelihood Estimation for Causal Inference in Observational Studies. Am J Epidemiol. 2017;185:65–73. 27941068 10.1093/aje/kww165

[24] Piulats JM, Watkins C, Costa-García M, Del Carpio L, Piperno-Neumann S, Rutkowski P, et al. Overall survival from tebentafusp versus nivolumab plus ipilimumab in first-line metastatic uveal melanoma: a propensity score-weighted analysis. Ann Oncol. 2024;35:317–26. 38048850 10.1016/j.annonc.2023.11.013

[25] Rossides M, Kullberg S, Di Giuseppe D, Eklund A, Grunewald J, Askling J, et al. Infection risk in sarcoidosis patients treated with methotrexate compared to azathioprine: A retrospective ‘target trial’ emulated with Swedish real-world data. Respirology. 2021;26:452–60. 33398914 10.1111/resp.14001 PMC8247001

[26] Reyes LF, Garcia E, Ibáñez-Prada ED, Serrano-Mayorga CC, Fuentes YV, Rodríguez A, et al. Impact of macrolide treatment on long-term mortality in patients admitted to the ICU due to CAP: a targeted maximum likelihood estimation and survival analysis. Crit Care. 2023;27:212. 37259125 10.1186/s13054-023-04466-x PMC10230128

[27] Chen WW, Shao YY, Shau WY, Lin ZZ, Lu YS, Chen HM, et al. The impact of diabetes mellitus on prognosis of early breast cancer in Asia. Oncologist. 2012;17:485–91. 22467665 10.1634/theoncologist.2011-0412 PMC3336825

[28] Lao C, Gurney J, Stanley J, Krebs J, Meredith I, Campbell I, et al. Association of diabetes and breast cancer characteristics at diagnosis. Cancer Causes Control. 2023;34:103–11. 36409455 10.1007/s10552-022-01654-y

[29] Boussaid I, Le Goff S, Floquet C, Gautier EF, Raimbault A, Viailly PJ, et al. Integrated analyses of translatome and proteome identify the rules of translation selectivity in RPS14-deficient cells. Haematologica. 2021;106:746–58. 32327500 10.3324/haematol.2019.239970 PMC7927886

[30] Khajuria RK, Munschauer M, Ulirsch JC, Fiorini C, Ludwig LS, McFarland SK, et al. Ribosome Levels Selectively Regulate Translation and Lineage Commitment in Human Hematopoiesis. Cell. 2018;173:90–103.e119. 29551269 10.1016/j.cell.2018.02.036 PMC5866246

[31] Ingolia NT, Ghaemmaghami S, Newman JR, Weissman JS. Genome-wide analysis in vivo of translation with nucleotide resolution using ribosome profiling. Science. 2009;324:218–23. 19213877 10.1126/science.1168978 PMC2746483

[32] Williams HM, Moeller JB, Burns I, Schlosser A, Sorensen GL, Greenhough TJ, et al. Crystal structures of human immune protein FIBCD1 suggest an extended binding site compatible with recognition of pathogen-associated carbohydrate motifs. J Biol Chem. 2024;300:105552. 38072065 10.1016/j.jbc.2023.105552 PMC10825690

[33] Sutendra G, Kinnaird A, Dromparis P, Paulin R, Stenson TH, Haromy A, et al. A nuclear pyruvate dehydrogenase complex is important for the generation of acetyl-CoA and histone acetylation. Cell. 2014;158:84–97. 24995980 10.1016/j.cell.2014.04.046

[34] Jakkamsetti V, Ma Q, Pascual JM. A subset of synaptic transmission events is coupled to acetyl coenzyme A production. J Neurophysiol. 2022;127:623–36. 35080429 10.1152/jn.00200.2021 PMC8897004

[35] Austin PC, Stuart EA. Moving towards best practice when using inverse probability of treatment weighting (IPTW) using the propensity score to estimate causal treatment effects in observational studies. Stat Med. 2015;34:3661–79. 26238958 10.1002/sim.6607 PMC4626409

[36] Snowden JM, Rose S, Mortimer KM. Implementation of G-computation on a simulated data set: demonstration of a causal inference technique. Am J Epidemiol. 2011;173:731–8. 21415029 10.1093/aje/kwq472 PMC3105284

[37] Royston P, Parmar MK. Restricted mean survival time: an alternative to the hazard ratio for the design and analysis of randomized trials with a time-to-event outcome. BMC Med Res Methodol. 2013;13:152. 24314264 10.1186/1471-2288-13-152 PMC3922847

[38] Wang Y, Sun M, Liu J, Liu Y, Jiang C, Zhu H, et al. FIBCD1 overexpression predicts poor prognosis in patients with hepatocellular carcinoma. Oncol Lett. 2020;19:795–804. 31897196 10.3892/ol.2019.11183 PMC6924150

[39] Shrive AK, Moeller JB, Burns I, Paterson JM, Shaw AJ, Schlosser A, et al. Crystal structure of the tetrameric fibrinogen-like recognition domain of fibrinogen C domain containing 1 (FIBCD1) protein. J Biol Chem. 2014;289:2880–7. 24293368 10.1074/jbc.M113.520577 PMC3908420

[40] Jiang C, Zhu J, Zhou P, Zhu H, Wang W, Jin Q, et al. Overexpression of FIBCD1 Is Predictive of Poor Prognosis in Gastric Cancer. Am J Clin Pathol. 2018;149:474–83. 29659669 10.1093/ajcp/aqy013

[41] Simonetti G, Padella A, do Valle IF, Fontana MC, Fonzi E, Bruno S, et al. Aneuploid acute myeloid leukemia exhibits a signature of genomic alterations in the cell cycle and protein degradation machinery. Cancer. 2019;125:712–25. 30480765 10.1002/cncr.31837 PMC6587451

[42] Sharma G, Sharma A, Krishna M, Ahluwalia P, Gautam G. Diagnostic performance of minichromosome maintenance 5 (MCM5) in bladder cancer: A systematic review and meta-analysis. Urol Oncol. 2022;40:235–42. 35414492 10.1016/j.urolonc.2022.03.001

[43] Mao J, Shen J, Lu X, Cai Y, Tao R, Deng Y, et al. MCM5 is an oncogene of colon adenocarcinoma and promotes progression through cell cycle control. Acta Histochem. 2023;125:152072. 37385108 10.1016/j.acthis.2023.152072

[44] Wang D, Li Q, Li Y, Wang H. The role of MCM5 expression in cervical cancer: Correlation with progression and prognosis. Biomed Pharmacother. 2018;98:165–72. 29253764 10.1016/j.biopha.2017.12.006

[45] Stockley J, Akhand R, Kennedy A, Nyberg C, Crosbie EJ, Edmondson RJ. Detection of MCM5 as a novel non-invasive aid for the diagnosis of endometrial and ovarian tumours. BMC Cancer. 2020;20:1000. 33059604 10.1186/s12885-020-07468-y PMC7559715

[46] Zhang LL, Li Q, Zhong DS, Zhang WJ, Sun XJ, Zhu Y. MCM5 Aggravates the HDAC1-Mediated Malignant Progression of Lung Cancer. Front Cell Dev Biol. 2021;9:669132. 34409025 10.3389/fcell.2021.669132 PMC8366603

[47] Guo P, Chen W, Li H, Li M, Li L. The Histone Acetylation Modifications of Breast Cancer and their Therapeutic Implications. Pathol Oncol Res. 2018;24:807–13. 29948617 10.1007/s12253-018-0433-5

[48] Mocholi E, Russo L, Gopal K, Ramstead AG, Hochrein SM, Vos HR, et al. Pyruvate metabolism controls chromatin remodeling during CD4(+) T cell activation. Cell Rep. 2023;42:112583. 37267106 10.1016/j.celrep.2023.112583

[49] Tsilidis KK, Kasimis JC, Lopez DS, Ntzani EE, Ioannidis JP. Type 2 diabetes and cancer: umbrella review of meta-analyses of observational studies. BMJ. 2015;350:g7607. 25555821 10.1136/bmj.g7607

[50] Erickson K, Patterson RE, Flatt SW, Natarajan L, Parker BA, Heath DD, et al. Clinically defined type 2 diabetes mellitus and prognosis in early-stage breast cancer. J Clin Oncol. 2011;29:54–60. 21115861 10.1200/JCO.2010.29.3183 PMC3055860

[51] Shan Y, Zhao J, Wei K, Jiang P, Shi Y, Chang C, et al. Multi-target RNA interference: A disruptive next-generation strategy for precision treatment of rheumatoid arthritis. Int Immunopharmacol. 2025;159:114890. 40394795 10.1016/j.intimp.2025.114890

[52] Edge SB, Compton CC. The American Joint Committee on Cancer: the 7th edition of the AJCC cancer staging manual and the future of TNM. Ann Surg Oncol. 2010;17:1471–4. 20180029 10.1245/s10434-010-0985-4

[53] American Diabetes Association. Sec. 2. Classification and Diagnosis of Diabetes. Diabetes Care. 2016; 39 Suppl 1:S13-22. 10.2337/dc16-S005 26696675

[54] Luque-Fernandez MA, Schomaker M, Rachet B, Schnitzer ME. Targeted maximum likelihood estimation for a binary treatment: A tutorial. Stat Med. 2018;37:2530–46. 29687470 10.1002/sim.7628 PMC6032875

[55] van der Laan MJ, Polley EC, Hubbard AE. Super learner. Stat Appl Genet Mol Biol. 2007;6:Article25. 17910531 10.2202/1544-6115.1309

[56] Ren J, Cislo P, Cappelleri JC, Hlavacek P, DiBonaventura M. Comparing g-computation, propensity score-based weighting, and targeted maximum likelihood estimation for analyzing externally controlled trials with both measured and unmeasured confounders: a simulation study. BMC Med Res Methodol. 2023;23:18. 36647031 10.1186/s12874-023-01835-6 PMC9843888

[57] Nandy SB, Orozco A, Lopez-Valdez R, Roberts R, Subramani R, Arumugam A, et al. Glucose insult elicits hyperactivation of cancer stem cells through miR-424-cdc42-prdm14 signalling axis. Br J Cancer. 2017;117:1665–75. 29024936 10.1038/bjc.2017.335 PMC5729435

[58] Huang Y, Chen C, Liu Y, Tan B, Xiang Q, Chen Q, et al. Downregulation of tRF-Cys-GCA-029 by hyperglycemia promotes tumorigenesis and glycolysis of diabetic breast cancer through upregulating PRKCG translation. Breast Cancer Res. 2024;26:117. 39039568 10.1186/s13058-024-01870-1 PMC11265092

[59] Yang W, Gao K, Qian Y, Huang Y, Xiang Q, Chen C, et al. A novel tRNA-derived fragment AS-tDR-007333 promotes the malignancy of NSCLC via the HSPB1/MED29 and ELK4/MED29 axes. J Hematol Oncol. 2022;15:53. 35526007 10.1186/s13045-022-01270-y PMC9077895

