# Supplementary material for: Diabetes is causally associated with increased breast cancer mortality by inducing FIBCD1 to activate MCM5-mediated cell cycle arrest via modulating H3K27ac
Source: Cell Death Dis. 2025 Jul 22;16(1):546. doi: 10.1038/s41419-025-07849-w (PMC12283923; doi:10.1038/s41419-025-07849-w)
Supplement: Supplementary file 1 — Supplementary materials [file 41419_2025_7849_MOESM1_ESM.docx]

**Supplementary materials**

| **Table S1** Comparison of demographic and clinical characteristics by 5-year mortality status | | | | |
| --- | --- | --- | --- | --- |
| **Variable** | **Overall**  **N = 3386** | **Survival**  **N = 3171** | **Dead**  **N = 215** | **P-value** |
| Age | 50.50 (44.00, 58.00) | 50.00 (44.00, 58.00) | 55.00 (46.00, 62.00) | <0.001 |
| Diabetes | 300 (8.86%) | 266 (8.39%) | 34 (15.81%) | <0.001 |
| Hypertension | 2,158 (63.73%) | 2,024 (63.83%) | 134 (62.33%) | 0.657 |
| Lymphatic_metastasis | 2,329 (68.78%) | 2,171 (68.46%) | 158 (73.49%) | 0.124 |
| Ki67 | 1,391 (41.08%) | 1,326 (41.82%) | 65 (30.23%) | <0.001 |
| BMI | 23.83 (21.64, 26.02) | 23.83 (21.64, 26.03) | 23.63 (21.36, 25.79) | 0.321 |
| Stage I | 1,535 (45.33%) | 1,470 (46.36%) | 65 (30.23%) | <0.001 |
| Stage II | 1,376 (40.64%) | 1,276 (40.24%) | 100 (46.51%) | 0.070 |
| Stage III | 475 (14.03%) | 425 (13.40%) | 50 (23.26%) | <0.001 |
| HER2 | 2,507 (74.04%) | 2,337 (73.70%) | 170 (79.07%) | 0.082 |
| ER | 2,459 (72.62%) | 2,323 (73.26%) | 136 (63.26%) | 0.001 |
| PR | 2,156 (63.67%) | 2,047 (64.55%) | 109 (50.70%) | <0.001 |
| Post-menopause | 1,568 (46.31%) | 1,467 (46.26%) | 101 (46.98%) | 0.839 |

| **Table S2.** The causal effect of co-existing DM on BC mortality after excluding patients with stage III | |
| --- | --- |
| **Causal Inference method** | **OR (95% CI)** |
| GC | 1.926 (1.082, 2.943) |
| IPTW | 2.268 (1.063, 3.974) |
| TMLE | 1.917 (1.091, 2.953) |
| TMLE+SL | 2.113 (1.365, 3.270) |

| **Table S3.** The causal effect of co-existing DM on BC mortality after excluding subjects with age>=70 | |
| --- | --- |
| **Causal Inference method** | **OR (95% CI)** |
| GC | 1.878 (1.160, 2.827) |
| IPTW | 2.197 (1.069, 3.693) |
| TMLE | 1.866 (1.158, 2.877) |
| TMLE+SL | 1.939 (1.240, 3.033) |

| **Table S4. Differentially Expressed Genes between BC-DM and BC-NO-DM tumor tissues** | | | | | | |
| --- | --- | --- | --- | --- | --- | --- |
| **Track_id** | **Gene_Name** | **Locus** | **log2FC** | **Fold_Change** | **p_value** | **q_value** |
| ENSG00000229453.2_2 | SPINK8 | chr3:48348332-48369831 | 2.704016348 | 6.51613434 | 0.031125417 | 0.928591203 |
| ENSG00000163993.6_2 | S100P | chr4:6694796-6698897 | 2.651813192 | 6.284566322 | 0.022129153 | 0.928591203 |
| ENSG00000134240.11_2 | HMGCS2 | chr1:120290619-120311528 | 2.583252963 | 5.992894442 | 0.033856571 | 0.928591203 |
| ENSG00000164756.12_3 | SLC30A8 | chr8:117962512-118188953 | 2.212984312 | 4.63633339 | 0.047096732 | 0.928591203 |
| ENSG00000130720.12_2 | FIBCD1 | chr9:133777825-133814673 | 1.773812287 | 3.419563753 | 0.00898047 | 0.928591203 |
| ENSG00000151117.8_2 | TMEM86A | chr11:18714669-18726332 | 1.721896856 | 3.29869835 | 0.033369653 | 0.928591203 |
| ENSG00000115902.10_2 | SLC1A4 | chr2:65215611-65250999 | 1.692753304 | 3.232730642 | 0.010505377 | 0.928591203 |
| ENSG00000204128.5_2 | C2orf72 | chr2:231902205-231914434 | 1.623309827 | 3.080810248 | 0.004847009 | 0.928591203 |
| ENSG00000064651.13_3 | SLC12A2 | chr5:127419458-127525380 | 1.536637306 | 2.901174968 | 0.034307165 | 0.928591203 |
| ENSG00000171161.12_2 | ZNF672 | chr1:249132409-249143716 | 1.51868109 | 2.865289853 | 0.004136769 | 0.928591203 |
| ENSG00000117009.11_2 | KMO | chr1:241695434-241758944 | 1.511771855 | 2.851600453 | 0.018012751 | 0.928591203 |
| ENSG00000206053.12_4 | JPT2 | chr16:1728257-1752281 | 1.497494436 | 2.823519188 | 0.031698904 | 0.928591203 |
| ENSG00000109586.11_2 | GALNT7 | chr4:174089904-174245118 | 1.43631168 | 2.706281064 | 0.022481897 | 0.928591203 |
| ENSG00000223572.9_2 | CKMT1A | chr15:43985084-43991420 | 1.370209176 | 2.585080444 | 0.020866014 | 0.928591203 |
| ENSG00000145423.4_2 | SFRP2 | chr4:154701744-154710272 | 1.359196004 | 2.56542172 | 0.028251953 | 0.928591203 |
| ENSG00000136193.16_3 | SCRN1 | chr7:29959719-30029905 | 1.331084999 | 2.515918169 | 0.036868458 | 0.928591203 |
| ENSG00000143797.11_3 | MBOAT2 | chr2:8992820-9143942 | 1.303891087 | 2.46893882 | 0.01522798 | 0.928591203 |
| ENSG00000112378.11_2 | PERP | chr6:138409642-138428648 | 1.271445883 | 2.414033812 | 0.034854602 | 0.928591203 |
| ENSG00000141741.11_2 | MIEN1 | chr17:37884749-37887040 | 1.247184671 | 2.373777429 | 0.030422069 | 0.928591203 |
| ENSG00000170231.15_2 | FABP6 | chr5:159614374-159665742 | 1.245641347 | 2.371239437 | 0.03725734 | 0.928591203 |
| ENSG00000284292.1_2 | AC004922.1 | chr7:98923502-98992276 | 1.242369967 | 2.36586863 | 0.035451157 | 0.928591203 |
| ENSG00000197496.5_2 | SLC2A10 | chr20:45338126-45364986 | 1.241262164 | 2.364052646 | 0.005759349 | 0.928591203 |
| ENSG00000006625.17_3 | GGCT | chr7:30536237-30544460 | 1.1857685 | 2.274845396 | 0.018799456 | 0.928591203 |
| ENSG00000114405.10_2 | C3orf14 | chr3:62304648-62321888 | 1.143787111 | 2.209602889 | 0.019870369 | 0.928591203 |
| ENSG00000184012.11_3 | TMPRSS2 | chr21:42836478-42903043 | 1.142397064 | 2.207474946 | 0.016671296 | 0.928591203 |
| ENSG00000143590.13_3 | EFNA3 | chr1:155051348-155060014 | 1.141081686 | 2.205463196 | 0.035131012 | 0.928591203 |
| ENSG00000120149.8_2 | MSX2 | chr5:174151536-174157896 | 1.123458064 | 2.178685666 | 0.026639747 | 0.928591203 |
| ENSG00000203985.10_2 | LDLRAD1 | chr1:54472972-54483859 | 1.083146422 | 2.118651685 | 0.016444903 | 0.928591203 |
| ENSG00000166801.15_2 | FAM111A | chr11:58910221-58922512 | 1.078850942 | 2.112352993 | 0.035878042 | 0.928591203 |
| ENSG00000183077.15_2 | AFMID | chr17:76183398-76203782 | 1.073655793 | 2.104760083 | 0.032472703 | 0.928591203 |
| ENSG00000077585.13_3 | GPR137B | chr1:236305805-236385165 | 1.073177489 | 2.104062397 | 0.019494306 | 0.928591203 |
| ENSG00000114654.7_2 | EFCC1 | chr3:128720472-128759585 | 1.056733258 | 2.080215884 | 0.018942801 | 0.928591203 |
| ENSG00000183840.6_3 | GPR39 | chr2:133174147-133404132 | 1.045524045 | 2.064115992 | 0.005003265 | 0.928591203 |
| ENSG00000184828.9_3 | ZBTB7C | chr18:45553044-45937123 | 1.027336133 | 2.03825723 | 0.035742551 | 0.928591203 |
| ENSG00000172780.16_3 | RAB43 | chr3:128806412-128841644 | 1.024657752 | 2.034476692 | 0.045629748 | 0.928591203 |
| ENSG00000198740.8_3 | ZNF652 | chr17:47366568-47439835 | 0.999294168 | 1.999021749 | 0.029390315 | 0.928591203 |
| ENSG00000187867.8_2 | PALM3 | chr19:14164177-14169971 | 0.994943144 | 1.99300198 | 0.038054592 | 0.928591203 |
| ENSG00000006118.14_4 | TMEM132A | chr11:60691935-60704631 | 0.971460193 | 1.960824197 | 0.033990824 | 0.928591203 |
| ENSG00000141934.9_3 | PLPP2 | chr19:281040-291504 | 0.967658717 | 1.955664262 | 0.046208581 | 0.928591203 |
| ENSG00000162729.13_3 | IGSF8 | chr1:160061130-160068733 | 0.952271323 | 1.934916518 | 0.016235819 | 0.928591203 |
| ENSG00000090661.11_2 | CERS4 | chr19:8271620-8327305 | 0.937396375 | 1.915069002 | 0.036666148 | 0.928591203 |
| ENSG00000120992.17_2 | LYPLA1 | chr8:54958927-55014577 | 0.922305394 | 1.895141268 | 0.002470839 | 0.928591203 |
| ENSG00000117143.13_4 | UAP1 | chr1:162531296-162569632 | 0.921666636 | 1.894302374 | 0.002490592 | 0.928591203 |
| ENSG00000159231.5_2 | CBR3 | chr21:37507210-37518864 | 0.912920708 | 1.882853442 | 0.03150807 | 0.928591203 |
| ENSG00000187678.8_3 | SPRY4 | chr5:141689992-141706020 | 0.908284427 | 1.876812369 | 0.011674927 | 0.928591203 |
| ENSG00000168283.13_3 | BMI1 | chr10:22610140-22620413 | 0.907023979 | 1.87517336 | 0.04517933 | 0.928591203 |
| ENSG00000196421.8_4 | C20orf204 | chr20:62665697-62671315 | 0.903617862 | 1.870751409 | 0.049300102 | 0.928591203 |
| ENSG00000128039.10_2 | SRD5A3 | chr4:56212276-56239263 | 0.888553785 | 1.851319357 | 0.045889606 | 0.928591203 |
| ENSG00000054179.11_3 | ENTPD2 | chr9:139942550-139948497 | 0.885175417 | 1.846989183 | 0.027768407 | 0.928591203 |
| ENSG00000197191.4_2 | CYSRT1 | chr9:140119087-140120763 | 0.883169064 | 1.844422363 | 0.038375451 | 0.928591203 |
| ENSG00000167968.12_3 | DNASE1L2 | chr16:2285817-2288712 | 0.88285934 | 1.844026438 | 0.042981656 | 0.928591203 |
| ENSG00000153982.10_3 | GDPD1 | chr17:57297828-57353328 | 0.876128169 | 1.835442818 | 0.049623825 | 0.928591203 |
| ENSG00000145022.4_2 | TCTA | chr3:49449639-49453908 | 0.868723373 | 1.826046335 | 0.021097449 | 0.928591203 |
| ENSG00000131779.10_3 | PEX11B | chr1:145516252-145523730 | 0.858935726 | 1.813699853 | 0.023563299 | 0.928591203 |
| ENSG00000260001.6_2 | TGFBR3L | chr19:7981030-7983982 | 0.858808311 | 1.813539679 | 0.008623531 | 0.928591203 |
| ENSG00000155729.12_2 | KCTD18 | chr2:201353675-201384507 | 0.856862608 | 1.811095482 | 0.004376238 | 0.928591203 |
| ENSG00000180891.12_3 | CUEDC1 | chr17:55938604-56032684 | 0.847542965 | 1.799433722 | 0.007968996 | 0.928591203 |
| ENSG00000168907.13_2 | PLA2G4F | chr15:42431232-42448834 | 0.834139171 | 1.782792962 | 0.041510682 | 0.928591203 |
| ENSG00000162065.12_3 | TBC1D24 | chr16:2525052-2559561 | 0.831148585 | 1.77910121 | 0.02264425 | 0.928591203 |
| ENSG00000170619.9_3 | COMMD5 | chr8:146066427-146079121 | 0.828956347 | 1.77639984 | 0.046109422 | 0.928591203 |
| ENSG00000256683.6_3 | ZNF350 | chr19:52467596-52490109 | 0.826310156 | 1.773144555 | 0.026806892 | 0.928591203 |
| ENSG00000143061.17_3 | IGSF3 | chr1:117117021-117210377 | 0.819110556 | 1.764317928 | 0.029656221 | 0.928591203 |
| ENSG00000188171.15_3 | ZNF626 | chr19:20802867-20844402 | 0.787225576 | 1.725752502 | 0.015276492 | 0.928591203 |
| ENSG00000171307.18_2 | ZDHHC16 | chr10:99205888-99217127 | 0.785994568 | 1.724280597 | 0.022612867 | 0.928591203 |
| ENSG00000132825.6_2 | PPP1R3D | chr20:58511723-58515352 | 0.77557596 | 1.711873337 | 0.003764374 | 0.928591203 |
| ENSG00000187695.8_4 | AC112484.1 | chr3:128628717-128690173 | 0.775496242 | 1.711778748 | 0.021659787 | 0.928591203 |
| ENSG00000166578.9_2 | IQCD | chr12:113633246-113658899 | 0.773243763 | 1.709108234 | 0.025974124 | 0.928591203 |
| ENSG00000159267.14_3 | HLCS | chr21:38123189-38362536 | 0.77309014 | 1.708926253 | 0.035804258 | 0.928591203 |
| ENSG00000186104.10_3 | CYP2R1 | chr11:14898986-14913798 | 0.770574199 | 1.705948623 | 0.024838774 | 0.928591203 |
| ENSG00000160221.16_3 | C21orf33 | chr21:45553487-45565605 | 0.76458674 | 1.698883284 | 0.00231865 | 0.928591203 |
| ENSG00000205133.11_3 | TRIQK | chr8:93895758-94029901 | 0.753949373 | 1.686403041 | 0.030979851 | 0.928591203 |
| ENSG00000102471.13_2 | NDFIP2 | chr13:80055259-80130210 | 0.750522453 | 1.68240198 | 0.002038115 | 0.928591203 |
| ENSG00000149380.11_2 | P4HA3 | chr11:73946846-74022702 | 0.744933822 | 1.675897392 | 0.011961323 | 0.928591203 |
| ENSG00000204237.4_3 | OXLD1 | chr17:79632066-79633665 | 0.742314923 | 1.672857925 | 0.029067625 | 0.928591203 |
| ENSG00000163701.18_3 | IL17RE | chr3:9944296-9958086 | 0.741470102 | 1.671878611 | 0.023451389 | 0.928591203 |
| ENSG00000197070.13_3 | ARRDC1 | chr9:140500106-140509812 | 0.738656796 | 1.668621565 | 0.015846736 | 0.928591203 |
| ENSG00000129235.10_2 | TXNDC17 | chr17:6544078-6547861 | 0.737031094 | 1.666742337 | 0.005488318 | 0.928591203 |
| ENSG00000083782.7_2 | EPYC | chr12:91357456-91398803 | 0.736366495 | 1.665974704 | 0.046777877 | 0.928591203 |
| ENSG00000188603.18_3 | CLN3 | chr16:28485432-28506896 | 0.735045724 | 1.664450221 | 0.017588643 | 0.928591203 |
| ENSG00000177888.7_2 | ZBTB41 | chr1:197122810-197169672 | 0.734233204 | 1.663513073 | 0.027661013 | 0.928591203 |
| ENSG00000008283.15_3 | CYB561 | chr17:61509665-61523739 | 0.7339833 | 1.663224944 | 0.046504377 | 0.928591203 |
| ENSG00000170113.15_4 | NIPA1 | chr15:23043277-23100005 | 0.730951773 | 1.659733688 | 0.044646503 | 0.928591203 |
| ENSG00000148444.15_3 | COMMD3 | chr10:22604903-22609237 | 0.722900393 | 1.650496859 | 0.019221417 | 0.928591203 |
| ENSG00000234444.9_3 | ZNF736 | chr7:63767837-63815238 | 0.719070962 | 1.646121657 | 0.022107053 | 0.928591203 |
| ENSG00000116747.12_3 | TROVE2 | chr1:193028552-193060907 | 0.718619335 | 1.645606429 | 0.0044266 | 0.928591203 |
| ENSG00000197467.13_3 | COL13A1 | chr10:71561687-71724031 | 0.716569349 | 1.643269778 | 0.025004384 | 0.928591203 |
| ENSG00000143537.13_3 | ADAM15 | chr1:155023042-155035251 | 0.711613791 | 1.637634942 | 0.042187834 | 0.928591203 |
| ENSG00000170412.16_3 | GPRC5C | chr17:72420990-72447792 | 0.706508522 | 1.631850081 | 0.035172777 | 0.928591203 |
| ENSG00000178904.18_2 | DPY19L3 | chr19:32896449-32976801 | 0.705940585 | 1.631207807 | 0.011155834 | 0.928591203 |
| ENSG00000160439.15_3 | RDH13 | chr19:55550476-55582659 | 0.701345194 | 1.626020215 | 0.019586584 | 0.928591203 |
| ENSG00000129946.10_2 | SHC2 | chr19:416583-460996 | 0.696971026 | 1.621097682 | 0.017596031 | 0.928591203 |
| ENSG00000180855.15_3 | ZNF443 | chr19:12540521-12551926 | 0.696967087 | 1.621093257 | 0.027375275 | 0.928591203 |
| ENSG00000114023.15_2 | FAM162A | chr3:122103023-122131181 | 0.693925068 | 1.617678674 | 0.032531436 | 0.928591203 |
| ENSG00000156239.11_2 | N6AMT1 | chr21:30244513-30257693 | 0.692704637 | 1.616310796 | 0.007967467 | 0.928591203 |
| ENSG00000153214.9_3 | TMEM87B | chr2:112812800-112876895 | 0.691888316 | 1.615396497 | 0.035343108 | 0.928591203 |
| ENSG00000127838.13_2 | PNKD | chr2:219135115-219211516 | 0.691232782 | 1.614662657 | 0.011708577 | 0.928591203 |
| ENSG00000205808.5_2 | PLPP6 | chr9:4662315-4665258 | 0.687052393 | 1.609990743 | 0.006374804 | 0.928591203 |
| ENSG00000023572.8_2 | GLRX2 | chr1:193065598-193075244 | 0.68690975 | 1.609831566 | 0.012213603 | 0.928591203 |
| ENSG00000010626.14_4 | LRRC23 | chr12:6982733-7023407 | 0.685485269 | 1.608242843 | 0.049305617 | 0.928591203 |
| ENSG00000008300.16_3 | CELSR3 | chr3:48673902-48700348 | 0.683501253 | 1.606032684 | 0.022391849 | 0.928591203 |
| ENSG00000135245.9_3 | HILPDA | chr7:128095903-128098472 | 0.676348179 | 1.598089469 | 0.029709946 | 0.928591203 |
| ENSG00000152433.14_3 | ZNF547 | chr19:57874845-57890933 | 0.674072964 | 1.595571174 | 0.008496556 | 0.928591203 |
| ENSG00000198105.13_3 | ZNF248 | chr10:38065454-38147034 | 0.665553839 | 1.586177077 | 0.003007816 | 0.928591203 |
| ENSG00000136888.6_2 | ATP6V1G1 | chr9:117350026-117360653 | 0.663741458 | 1.584185698 | 0.008461286 | 0.928591203 |
| ENSG00000159348.12_2 | CYB5R1 | chr1:202930997-202936408 | 0.663737616 | 1.58418148 | 0.048957631 | 0.928591203 |
| ENSG00000198369.9_3 | SPRED2 | chr2:65537985-65659771 | 0.662861618 | 1.583219864 | 0.037278331 | 0.928591203 |
| ENSG00000166557.12_3 | TMED3 | chr15:79603404-79719774 | 0.658507718 | 1.578449078 | 0.04173926 | 0.928591203 |
| ENSG00000163702.18_3 | IL17RC | chr3:9958758-9975314 | 0.658197437 | 1.578109636 | 0.025689721 | 0.928591203 |
| ENSG00000196865.4_2 | NHLRC2 | chr10:115614420-115676953 | 0.657316781 | 1.577146613 | 0.037291187 | 0.928591203 |
| ENSG00000118640.10_2 | VAMP8 | chr2:85788685-85809154 | 0.656421482 | 1.57616818 | 0.036216284 | 0.928591203 |
| ENSG00000203995.9_2 | ZYG11A | chr1:53308183-53360670 | 0.654036558 | 1.573564764 | 0.012285889 | 0.928591203 |
| ENSG00000169962.4_2 | TAS1R3 | chr1:1266694-1270686 | 0.652608384 | 1.572007808 | 0.037550985 | 0.928591203 |
| ENSG00000152683.14_3 | SLC30A6 | chr2:32390910-32449448 | 0.649835812 | 1.568989625 | 0.03155774 | 0.928591203 |
| ENSG00000109089.7_2 | CDR2L | chr17:72983727-73001895 | 0.649706262 | 1.56884874 | 0.035452433 | 0.928591203 |
| ENSG00000213240.8_4 | AC239799.1 | chr1:145209145-145318987 | 0.648256484 | 1.56727298 | 0.031295211 | 0.928591203 |
| ENSG00000180758.11_3 | GPR157 | chr1:9160364-9189229 | 0.634101123 | 1.551970488 | 0.006661369 | 0.928591203 |
| ENSG00000110723.11_4 | EXPH5 | chr11:108376158-108464465 | 0.631564922 | 1.549244581 | 0.026267357 | 0.928591203 |
| ENSG00000145439.11_2 | CBR4 | chr4:169784921-169931426 | 0.630412636 | 1.548007688 | 0.032153095 | 0.928591203 |
| ENSG00000213139.7_3 | CRYGS | chr3:186256230-186264491 | 0.629113571 | 1.546614422 | 0.001483642 | 0.928591203 |
| ENSG00000186130.4_2 | ZBTB6 | chr9:125670335-125675609 | 0.629002789 | 1.546495664 | 0.00660917 | 0.928591203 |
| ENSG00000203666.12_2 | EFCAB2 | chr1:245133007-245290466 | 0.627729017 | 1.545130848 | 0.019007957 | 0.928591203 |
| ENSG00000175137.10_2 | SH3BP5L | chr1:249104645-249120832 | 0.626207174 | 1.543501809 | 0.042548152 | 0.928591203 |
| ENSG00000165724.5_2 | ZMYND19 | chr9:140476531-140484942 | 0.62605497 | 1.543338979 | 0.044305003 | 0.928591203 |
| ENSG00000239305.6_3 | RNF103 | chr2:86830516-86850989 | 0.623789128 | 1.540916971 | 0.040024469 | 0.928591203 |
| ENSG00000167080.8_2 | B4GALNT2 | chr17:47209822-47254202 | 0.613155123 | 1.529600737 | 0.019693952 | 0.928591203 |
| ENSG00000119559.15_3 | C19orf25 | chr19:1461142-1479555 | 0.608817595 | 1.525008829 | 0.00242492 | 0.928591203 |
| ENSG00000144791.9_3 | LIMD1 | chr3:45596886-45727830 | 0.603941646 | 1.51986338 | 0.0359712 | 0.928591203 |
| ENSG00000106086.18_3 | PLEKHA8 | chr7:30067020-30170099 | 0.6035697 | 1.519471589 | 0.029926385 | 0.928591203 |
| ENSG00000128000.15_3 | ZNF780B | chr19:40534167-40562116 | 0.603024761 | 1.518897758 | 0.021132459 | 0.928591203 |
| ENSG00000180787.5_2 | ZFP3 | chr17:4981543-4999669 | 0.602214093 | 1.51804451 | 0.007717504 | 0.928591203 |
| ENSG00000106080.10_2 | FKBP14 | chr7:30050203-30066300 | 0.602099127 | 1.517923546 | 0.004983084 | 0.928591203 |
| ENSG00000099246.16_2 | RAB18 | chr10:27793103-27831166 | 0.601452465 | 1.517243316 | 0.017950393 | 0.928591203 |
| ENSG00000169955.7_3 | ZNF747 | chr16:30541688-30546668 | 0.600476284 | 1.51621704 | 0.026983049 | 0.928591203 |
| ENSG00000228300.13_3 | C19orf24 | chr19:1275437-1279248 | 0.599585903 | 1.515281573 | 0.02663134 | 0.928591203 |
| ENSG00000198858.9_2 | R3HDM4 | chr19:896503-913245 | 0.598165896 | 1.513790854 | 0.035308797 | 0.928591203 |
| ENSG00000215012.8_4 | RTL10 | chr22:19833661-19842419 | 0.597651314 | 1.51325101 | 0.049648186 | 0.928591203 |
| ENSG00000156381.8_3 | ANKRD9 | chr14:102968097-102976136 | 0.596707393 | 1.512261249 | 0.031664899 | 0.928591203 |
| ENSG00000155903.11_3 | RASA2 | chr3:141205889-141334184 | 0.59427094 | 1.509709468 | 0.048317152 | 0.928591203 |
| ENSG00000203811.1_2 | HIST2H3C | chr1:149811110-149812765 | 0.592702983 | 1.508069569 | 0.03149237 | 0.928591203 |
| ENSG00000180185.11_2 | FAHD1 | chr16:1876942-1890208 | 0.592641083 | 1.508004865 | 0.026655926 | 0.928591203 |
| ENSG00000162066.14_3 | AMDHD2 | chr16:2570358-2581423 | 0.592167095 | 1.507509502 | 0.035264286 | 0.928591203 |
| ENSG00000181588.16_3 | MEX3D | chr19:1554668-1568057 | 0.590470057 | 1.505737265 | 0.018738119 | 0.928591203 |
| ENSG00000137460.8_2 | FHDC1 | chr4:153857504-153900848 | 0.589869762 | 1.505110868 | 0.042611838 | 0.928591203 |
| ENSG00000136122.15_2 | BORA | chr13:73301887-73330336 | 0.589789486 | 1.505027122 | 0.049175352 | 0.928591203 |
| ENSG00000187650.3_3 | VMAC | chr19:5904869-5910864 | 0.589213966 | 1.504426857 | 0.049128905 | 0.928591203 |
| ENSG00000115275.11_3 | MOGS | chr2:74688184-74692537 | 0.587679302 | 1.502827376 | 0.014221651 | 0.928591203 |
| ENSG00000130054.4_2 | FAM155B | chrX:68725084-68752351 | 0.586757555 | 1.501867517 | 0.013046085 | 0.928591203 |
| ENSG00000185658.13_3 | BRWD1 | chr21:40556102-40693485 | 0.585595723 | 1.500658519 | 0.032586235 | 0.928591203 |
| ENSG00000125740.13_3 | FOSB | chr19:45971253-45978437 | -3.629387347 | 0.08080636 | 0.001647176 | 0.928591203 |
| ENSG00000184557.4_3 | SOCS3 | chr17:76352859-76356158 | -2.960793462 | 0.128443567 | 0.014685418 | 0.928591203 |
| ENSG00000120738.7_2 | EGR1 | chr5:137801179-137805004 | -2.777391025 | 0.145855225 | 0.010483996 | 0.928591203 |
| ENSG00000189143.9_3 | CLDN4 | chr7:73213872-73247023 | -2.670721516 | 0.15704811 | 0.037532178 | 0.928591203 |
| ENSG00000128016.5_2 | ZFP36 | chr19:39897453-39900052 | -2.652058905 | 0.159092871 | 0.01278167 | 0.928591203 |
| ENSG00000170345.9_3 | FOS | chr14:75745477-75748933 | -2.584527626 | 0.166716913 | 0.019104653 | 0.928591203 |
| ENSG00000120129.5_2 | DUSP1 | chr5:172195093-172198198 | -2.514385421 | 0.175022776 | 0.015861812 | 0.928591203 |
| ENSG00000162772.16_3 | ATF3 | chr1:212738676-212794119 | -2.345316493 | 0.19678382 | 0.003983016 | 0.928591203 |
| ENSG00000177606.6_2 | JUN | chr1:59246460-59249999 | -2.055766087 | 0.240520858 | 0.006363587 | 0.928591203 |
| ENSG00000164764.10_3 | SBSPON | chr8:73976775-74036323 | -1.941272728 | 0.260386629 | 0.033515842 | 0.928591203 |
| ENSG00000152137.6_3 | HSPB8 | chr12:119616447-119658936 | -1.936432766 | 0.261261642 | 0.015388127 | 0.928591203 |
| ENSG00000123358.19_3 | NR4A1 | chr12:52416616-52453291 | -1.914051417 | 0.265346345 | 0.036254047 | 0.928591203 |
| ENSG00000142178.7_2 | SIK1 | chr21:44834395-44847008 | -1.859601728 | 0.275552338 | 0.015791608 | 0.928591203 |
| ENSG00000171223.5_2 | JUNB | chr19:12902310-12904129 | -1.824947455 | 0.282251381 | 0.022928209 | 0.928591203 |
| ENSG00000196136.17_3 | SERPINA3 | chr14:95078714-95090392 | -1.819819329 | 0.283256442 | 0.045134507 | 0.928591203 |
| ENSG00000158050.4_2 | DUSP2 | chr2:96808905-96811179 | -1.815096842 | 0.284185166 | 0.013349647 | 0.928591203 |
| ENSG00000144655.14_2 | CSRNP1 | chr3:39183346-39196053 | -1.767500751 | 0.293717117 | 0.01760312 | 0.928591203 |
| ENSG00000117228.9_2 | GBP1 | chr1:89518002-89531043 | -1.587280689 | 0.332798148 | 0.00863688 | 0.928591203 |
| ENSG00000205364.3_2 | MT1M | chr16:56666145-56667898 | -1.586459039 | 0.332987739 | 0.040190133 | 0.928591203 |
| ENSG00000169429.10_2 | CXCL8 | chr4:74606223-74609433 | -1.548908995 | 0.341768421 | 0.049402242 | 0.928591203 |
| ENSG00000205362.11_3 | MT1A | chr16:56672578-56673999 | -1.538111014 | 0.344336014 | 0.046513915 | 0.928591203 |
| ENSG00000117525.13_2 | F3 | chr1:94994781-95007356 | -1.481664579 | 0.358075427 | 0.042034408 | 0.928591203 |
| ENSG00000087074.7_3 | PPP1R15A | chr19:49375649-49379314 | -1.477543751 | 0.359099674 | 0.02696506 | 0.928591203 |
| ENSG00000140379.7_2 | BCL2A1 | chr15:80253231-80263788 | -1.455822106 | 0.364547293 | 0.03049322 | 0.928591203 |
| ENSG00000162654.8_2 | GBP4 | chr1:89646831-89664615 | -1.441513336 | 0.368180893 | 0.006461005 | 0.928591203 |
| ENSG00000130522.5_3 | JUND | chr19:18390570-18392432 | -1.440679651 | 0.368393714 | 0.015727719 | 0.928591203 |
| ENSG00000165030.3_2 | NFIL3 | chr9:94171327-94186144 | -1.420685672 | 0.373534739 | 0.040327291 | 0.928591203 |
| ENSG00000076555.15_3 | ACACB | chr12:109554400-109706031 | -1.415370198 | 0.374913531 | 0.039194431 | 0.928591203 |
| ENSG00000148053.15_2 | NTRK2 | chr9:87283466-87641985 | -1.370607214 | 0.386728445 | 0.046018919 | 0.928591203 |
| ENSG00000198576.3_2 | ARC | chr8:143692405-143695833 | -1.360886008 | 0.389343108 | 0.015776164 | 0.928591203 |
| ENSG00000110848.8_2 | CD69 | chr12:9905082-9913497 | -1.354971494 | 0.390942546 | 0.03367681 | 0.928591203 |
| ENSG00000135604.9_2 | STX11 | chr6:144471663-144509507 | -1.341914137 | 0.394496898 | 0.027920821 | 0.928591203 |
| ENSG00000111716.12_3 | LDHB | chr12:21788276-21910791 | -1.336682975 | 0.395929927 | 0.017221836 | 0.928591203 |
| ENSG00000118503.14_3 | TNFAIP3 | chr6:138188325-138204449 | -1.324948046 | 0.399163568 | 0.009143295 | 0.928591203 |
| ENSG00000113070.7_2 | HBEGF | chr5:139712428-139726216 | -1.30675895 | 0.404227968 | 0.034945224 | 0.928591203 |
| ENSG00000112149.9_2 | CD83 | chr6:14117487-14137149 | -1.304387237 | 0.404893044 | 0.003196068 | 0.928591203 |
| ENSG00000138642.14_3 | HERC6 | chr4:89299891-89364263 | -1.292802934 | 0.408157272 | 0.011369986 | 0.928591203 |
| ENSG00000147027.3_2 | TMEM47 | chrX:34645181-34675405 | -1.277456845 | 0.412522054 | 0.017552534 | 0.928591203 |
| ENSG00000187193.8_3 | MT1X | chr16:56716336-56718108 | -1.275968279 | 0.412947912 | 0.036429856 | 0.928591203 |
| ENSG00000119917.13_2 | IFIT3 | chr10:91087651-91100728 | -1.251357855 | 0.420052671 | 0.034605596 | 0.928591203 |
| ENSG00000091513.14_3 | TF | chr3:133464800-133497850 | -1.247084128 | 0.421298847 | 0.03424563 | 0.928591203 |
| ENSG00000003989.17_4 | SLC7A2 | chr8:17354597-17428082 | -1.226229156 | 0.427433191 | 0.031854172 | 0.928591203 |
| ENSG00000179094.15_3 | PER1 | chr17:8043790-8059824 | -1.214224431 | 0.431004718 | 0.009015172 | 0.928591203 |
| ENSG00000102760.12_2 | RGCC | chr13:42031695-42045018 | -1.203410537 | 0.434247505 | 0.028384714 | 0.928591203 |
| ENSG00000198300.12_3 | PEG3 | chr19:57321451-57352096 | -1.192106782 | 0.43766327 | 0.03997701 | 0.928591203 |
| ENSG00000164742.14_2 | ADCY1 | chr7:45613739-45762715 | -1.185428841 | 0.43969382 | 0.023106531 | 0.928591203 |
| ENSG00000131203.12_3 | IDO1 | chr8:39759794-39785963 | -1.17749542 | 0.442118368 | 0.012165737 | 0.928591203 |
| ENSG00000138814.16_2 | PPP3CA | chr4:101944566-102269435 | -1.168685313 | 0.444826514 | 0.026528459 | 0.928591203 |
| ENSG00000126561.16_2 | STAT5A | chr17:40439565-40463961 | -1.154999249 | 0.44906642 | 0.030701294 | 0.928591203 |
| ENSG00000102393.9_2 | GLA | chrX:100652791-100662913 | -1.148042262 | 0.451237145 | 0.006019445 | 0.928591203 |
| ENSG00000169439.11_3 | SDC2 | chr8:97505579-97624008 | -1.147079499 | 0.451538373 | 0.018920311 | 0.928591203 |
| ENSG00000221963.5_2 | APOL6 | chr22:36044442-36064456 | -1.123442158 | 0.458997386 | 0.010625771 | 0.928591203 |
| ENSG00000112183.14_2 | RBM24 | chr6:17281577-17294106 | -1.120065232 | 0.460073023 | 0.029773033 | 0.928591203 |
| ENSG00000122971.8_2 | ACADS | chr12:121163538-121177811 | -1.111975853 | 0.462659958 | 0.044262575 | 0.928591203 |
| ENSG00000055070.16_2 | SZRD1 | chr1:16679070-16724640 | -1.103813755 | 0.465284891 | 0.009777189 | 0.928591203 |
| ENSG00000125347.13_3 | IRF1 | chr5:131817301-131826490 | -1.095990513 | 0.467814825 | 0.002326749 | 0.928591203 |
| ENSG00000162645.12_3 | GBP2 | chr1:89571815-89616139 | -1.093189368 | 0.468724019 | 0.024322753 | 0.928591203 |
| ENSG00000167549.18_4 | CORO6 | chr17:27941774-27949925 | -1.091306779 | 0.469336062 | 0.009816819 | 0.928591203 |
| ENSG00000137285.9_2 | TUBB2B | chr6:3224495-3231964 | -1.069848363 | 0.476369066 | 0.041347155 | 0.928591203 |
| ENSG00000116106.11_3 | EPHA4 | chr2:222282747-222438922 | -1.038207147 | 0.486932214 | 0.024481217 | 0.928591203 |
| ENSG00000079335.18_3 | CDC14A | chr1:100810581-100985833 | -1.032173469 | 0.488972939 | 0.017664758 | 0.928591203 |
| ENSG00000125878.6_3 | TCF15 | chr20:584441-591042 | -1.029935339 | 0.489732098 | 0.019715638 | 0.928591203 |
| ENSG00000204592.8_2 | HLA-E | chr6:30457244-30461982 | -1.026756219 | 0.490812461 | 0.04682618 | 0.928591203 |
| ENSG00000144677.14_2 | CTDSPL | chr3:37903451-38025960 | -1.02315468 | 0.492039254 | 0.015489914 | 0.928591203 |
| ENSG00000240065.7_3 | PSMB9 | chr6:32811913-32827362 | -1.02258474 | 0.492233673 | 0.013457371 | 0.928591203 |
| ENSG00000092421.16_2 | SEMA6A | chr5:115779312-115910630 | -1.006404814 | 0.497785181 | 0.010113126 | 0.928591203 |
| ENSG00000104368.17_3 | PLAT | chr8:42032751-42065242 | -0.990967082 | 0.503140392 | 0.031928463 | 0.928591203 |
| ENSG00000234745.10_2 | HLA-B | chr6:31237268-31324965 | -0.988243259 | 0.504091223 | 0.003805821 | 0.928591203 |
| ENSG00000175183.9_2 | CSRP2 | chr12:77252495-77272840 | -0.986760896 | 0.504609441 | 0.047784041 | 0.928591203 |
| ENSG00000168394.10_2 | TAP1 | chr6:32812986-32821755 | -0.975322427 | 0.508626161 | 0.036865225 | 0.928591203 |
| ENSG00000162512.15_3 | SDC3 | chr1:31342314-31381608 | -0.972856071 | 0.509496425 | 0.014715762 | 0.928591203 |
| ENSG00000090975.12_3 | PITPNM2 | chr12:123468027-123634562 | -0.971760811 | 0.50988337 | 0.014866372 | 0.928591203 |
| ENSG00000063322.13_2 | MED29 | chr19:39881943-39891277 | -0.968629544 | 0.510991237 | 0.046592178 | 0.928591203 |
| ENSG00000133687.15_4 | TMTC1 | chr12:29653773-29937692 | -0.960060811 | 0.514035246 | 0.03422212 | 0.928591203 |
| ENSG00000100906.10_2 | NFKBIA | chr14:35870717-35873955 | -0.955261346 | 0.515748153 | 0.01820792 | 0.928591203 |
| ENSG00000148339.12_4 | SLC25A25 | chr9:130830480-130871524 | -0.928204352 | 0.525512013 | 0.025868596 | 0.928591203 |
| ENSG00000102755.11_2 | FLT1 | chr13:28874481-29069282 | -0.920229637 | 0.528424903 | 0.044748722 | 0.928591203 |
| ENSG00000115896.15_2 | PLCL1 | chr2:198669426-199437305 | -0.908271245 | 0.532823181 | 0.009330814 | 0.928591203 |
| ENSG00000221968.8_3 | FADS3 | chr11:61640991-61659523 | -0.892661865 | 0.538619414 | 0.026444951 | 0.928591203 |
| ENSG00000118689.14_2 | FOXO3 | chr6:108881038-109005977 | -0.883065384 | 0.54221413 | 0.038074762 | 0.928591203 |
| ENSG00000000938.12_2 | FGR | chr1:27938575-27961788 | -0.880643901 | 0.543124971 | 0.046064365 | 0.928591203 |
| ENSG00000138646.8_3 | HERC5 | chr4:89378268-89427314 | -0.878410806 | 0.543966305 | 0.022253957 | 0.928591203 |
| ENSG00000166831.8_2 | RBPMS2 | chr15:65032091-65067786 | -0.872718434 | 0.546116846 | 0.034770634 | 0.928591203 |
| ENSG00000119669.4_2 | IRF2BPL | chr14:77490886-77495042 | -0.872462228 | 0.546213839 | 0.01075487 | 0.928591203 |
| ENSG00000111537.4_2 | IFNG | chr12:68548548-68553527 | -0.867409253 | 0.548130283 | 0.018801493 | 0.928591203 |
| ENSG00000204267.13_3 | TAP2 | chr6:32789610-32806557 | -0.867189344 | 0.54821384 | 0.010663972 | 0.928591203 |
| ENSG00000115844.10_2 | DLX2 | chr2:172964167-172967628 | -0.866002105 | 0.548665168 | 0.035245512 | 0.928591203 |
| ENSG00000128284.19_3 | APOL3 | chr22:36536378-36562225 | -0.865959918 | 0.548681212 | 0.008915902 | 0.928591203 |
| ENSG00000151789.10_2 | ZNF385D | chr3:21453714-22414812 | -0.84363999 | 0.557235861 | 0.04613371 | 0.928591203 |
| ENSG00000006652.13_3 | IFRD1 | chr7:112063023-112121072 | -0.835210656 | 0.560501191 | 0.023351867 | 0.928591203 |
| ENSG00000150991.14_3 | UBC | chr12:125396150-125401914 | -0.824263137 | 0.564770588 | 0.017658303 | 0.928591203 |
| ENSG00000116285.12_2 | ERRFI1 | chr1:8064464-8086368 | -0.823095732 | 0.565227776 | 0.008655586 | 0.928591203 |
| ENSG00000124615.17_3 | MOCS1 | chr6:39867354-39902290 | -0.817704261 | 0.567344031 | 0.029611151 | 0.928591203 |
| ENSG00000007314.12_2 | SCN4A | chr17:62015914-62050278 | -0.813280341 | 0.56908642 | 0.041601747 | 0.928591203 |
| ENSG00000169129.14_2 | AFAP1L2 | chr10:116054583-116164515 | -0.804029558 | 0.572747214 | 0.037789683 | 0.928591203 |
| ENSG00000146278.10_2 | PNRC1 | chr6:89790470-89794879 | -0.802763517 | 0.573250051 | 0.02243278 | 0.928591203 |
| ENSG00000111666.10_4 | CHPT1 | chr12:102090725-102137918 | -0.788519904 | 0.578937734 | 0.036955141 | 0.928591203 |
| ENSG00000177432.6_3 | NAP1L5 | chr4:89617066-89619386 | -0.77554672 | 0.584167208 | 0.021665998 | 0.928591203 |
| ENSG00000206503.12_2 | HLA-A | chr6:29909037-29913661 | -0.773339591 | 0.585061589 | 0.027434277 | 0.928591203 |
| ENSG00000053747.15_3 | LAMA3 | chr18:21269407-21535030 | -0.769138153 | 0.586767897 | 0.023658814 | 0.928591203 |
| ENSG00000173621.8_3 | LRFN4 | chr11:66624118-66627946 | -0.76604888 | 0.588025702 | 0.035383205 | 0.928591203 |
| ENSG00000186204.14_3 | CYP4F12 | chr19:15783567-15807984 | -0.763998641 | 0.58886195 | 0.020582285 | 0.928591203 |
| ENSG00000204642.13_3 | HLA-F | chr6:29690552-29706305 | -0.761309891 | 0.589960434 | 0.022220282 | 0.928591203 |
| ENSG00000104998.3_2 | IL27RA | chr19:14142560-14164028 | -0.757037003 | 0.591710334 | 0.041420785 | 0.928591203 |
| ENSG00000119508.17_2 | NR4A3 | chr9:102584137-102629173 | -0.75135679 | 0.594044622 | 0.034099344 | 0.928591203 |
| ENSG00000163600.12_2 | ICOS | chr2:204801471-204826300 | -0.751048867 | 0.594171426 | 0.048130902 | 0.928591203 |
| ENSG00000186470.13_4 | BTN3A2 | chr6:26365387-26378548 | -0.748429697 | 0.595251107 | 0.033000089 | 0.928591203 |
| ENSG00000105429.12_3 | MEGF8 | chr19:42829761-42882921 | -0.746544144 | 0.596029588 | 0.048370898 | 0.928591203 |
| ENSG00000196850.5_2 | PPTC7 | chr12:110971050-111021125 | -0.743078893 | 0.59746293 | 0.014576332 | 0.928591203 |
| ENSG00000037042.8_2 | TUBG2 | chr17:40811323-40819024 | -0.737196422 | 0.599904009 | 0.022700226 | 0.928591203 |
| ENSG00000134326.11_4 | CMPK2 | chr2:6980701-7006766 | -0.734197019 | 0.601152524 | 0.035118883 | 0.928591203 |
| ENSG00000063127.15_3 | SLC6A16 | chr19:49792895-49828482 | -0.72736249 | 0.604007141 | 0.01668178 | 0.928591203 |
| ENSG00000109133.12_2 | TMEM33 | chr4:41937137-41962589 | -0.726757287 | 0.604260572 | 0.006744751 | 0.928591203 |
| ENSG00000151967.18_3 | SCHIP1 | chr3:159557650-159615149 | -0.724260459 | 0.605307252 | 0.014333255 | 0.928591203 |
| ENSG00000073060.15_2 | SCARB1 | chr12:125261402-125367214 | -0.722683442 | 0.605969278 | 0.0314765 | 0.928591203 |
| ENSG00000186827.10_2 | TNFRSF4 | chr1:1146706-1149518 | -0.718060713 | 0.607914058 | 0.028016664 | 0.928591203 |
| ENSG00000115758.12_3 | ODC1 | chr2:10580094-10588630 | -0.705429547 | 0.61325987 | 0.028440382 | 0.928591203 |
| ENSG00000253368.3_3 | TRNP1 | chr1:27320198-27327389 | -0.701319417 | 0.615009493 | 0.025050113 | 0.928591203 |
| ENSG00000154655.15_4 | L3MBTL4 | chr18:5954705-6415236 | -0.694271454 | 0.618021334 | 0.006559171 | 0.928591203 |
| ENSG00000068079.7_2 | IFI35 | chr17:41158742-41166473 | -0.693683932 | 0.618273068 | 0.033747217 | 0.928591203 |
| ENSG00000151929.9_2 | BAG3 | chr10:121410882-121437331 | -0.690748867 | 0.619532183 | 0.020331561 | 0.928591203 |
| ENSG00000172216.5_2 | CEBPB | chr20:48807271-48809226 | -0.68074306 | 0.623843881 | 0.007426536 | 0.928591203 |
| ENSG00000163734.4_2 | CXCL3 | chr4:74902306-74904524 | -0.679162103 | 0.624527886 | 0.046935359 | 0.928591203 |
| ENSG00000143479.15_3 | DYRK3 | chr1:206808881-206857764 | -0.679088605 | 0.624559704 | 0.046442327 | 0.928591203 |
| ENSG00000163864.15_3 | NMNAT3 | chr3:139279022-139396859 | -0.670552761 | 0.628265925 | 0.029924899 | 0.928591203 |
| ENSG00000064489.22_3 | BORCS8-MEF2B | chr19:19256376-19302967 | -0.668202327 | 0.629290327 | 0.019438466 | 0.928591203 |
| ENSG00000112096.16_3 | SOD2 | chr6:160090089-160183561 | -0.667194001 | 0.629730304 | 0.028844038 | 0.928591203 |
| ENSG00000197565.15_3 | COL4A6 | chrX:107398837-107682727 | -0.66432294 | 0.630984757 | 0.04566972 | 0.928591203 |
| ENSG00000147255.18_3 | IGSF1 | chrX:130407480-130712873 | -0.658218329 | 0.63366036 | 0.038975288 | 0.928591203 |
| ENSG00000102572.14_3 | STK24 | chr13:99097439-99230194 | -0.653768817 | 0.635617691 | 0.023454499 | 0.928591203 |
| ENSG00000176597.11_2 | B3GNT5 | chr3:182971032-183016292 | -0.64583531 | 0.639122636 | 0.028734869 | 0.928591203 |
| ENSG00000186417.13_3 | GLDN | chr15:51633826-51700210 | -0.643301981 | 0.640245902 | 0.049093464 | 0.928591203 |
| ENSG00000169136.10_3 | ATF5 | chr19:50431959-50437192 | -0.642604047 | 0.64055571 | 0.01772931 | 0.928591203 |
| ENSG00000036672.15_3 | USP2 | chr11:119225925-119252436 | -0.641928853 | 0.640855565 | 0.001707727 | 0.928591203 |
| ENSG00000185686.17_4 | PRAME | chr22:22890123-22901768 | -0.639494679 | 0.641937756 | 0.027353786 | 0.928591203 |
| ENSG00000113231.13_3 | PDE8B | chr5:76506274-76725632 | -0.627418515 | 0.647333686 | 0.037034975 | 0.928591203 |
| ENSG00000198382.8_3 | UVRAG | chr11:75526212-75854239 | -0.617501085 | 0.651798942 | 0.009749156 | 0.928591203 |
| ENSG00000185404.16_2 | SP140L | chr2:231191899-231268447 | -0.612329272 | 0.654139722 | 0.022726681 | 0.928591203 |
| ENSG00000137177.19_3 | KIF13A | chr6:17759414-17987854 | -0.609814721 | 0.655280851 | 0.02437984 | 0.928591203 |
| ENSG00000168610.14_3 | STAT3 | chr17:40465342-40540586 | -0.608776869 | 0.655752419 | 0.015926601 | 0.928591203 |
| ENSG00000091972.18_3 | CD200 | chr3:112051194-112081659 | -0.60198024 | 0.658849 | 0.027672008 | 0.928591203 |
| ENSG00000124191.17_2 | TOX2 | chr20:42543504-42698256 | -0.586064893 | 0.666157448 | 0.031088441 | 0.928591203 |

| **Table S5. Differentially expressed genes between FIBCD1-overexpressing and control cells** | | | | | | | |
| --- | --- | --- | --- | --- | --- | --- | --- |
| **Gene_id** | **FIBCD1** | **EV** | **log2FoldChange** | **pvalue** | **padj** | **gene_name** | **gene_chr** |
| ENSG00000237973 | 804.479 | 2521.185 | -1.64813 | 2.79E-21 | 4.14E-17 | MTCO1P12 | 1 |
| ENSG00000171723 | 886.4336 | 281.0649 | 1.657447 | 1.16E-15 | 8.63E-12 | GPHN | 14 |
| ENSG00000171345 | 887.9094 | 307.8788 | 1.527465 | 5.92E-15 | 2.93E-11 | KRT19 | 17 |
| ENSG00000111678 | 329.5872 | 1106.379 | -1.74828 | 2.20E-13 | 8.14E-10 | C12orf57 | 12 |
| ENSG00000130720 | 682.6191 | 262.9541 | 1.375493 | 1.49E-12 | 4.43E-09 | FIBCD1 | 9 |
| ENSG00000167996 | 4474.839 | 12509.16 | -1.48315 | 2.81E-12 | 6.94E-09 | FTH1 | 11 |
| ENSG00000224631 | 784.6436 | 1994.405 | -1.34591 | 4.16E-12 | 8.82E-09 | RPS27AP16 | 16 |
| ENSG00000237506 | 1000.435 | 336.2016 | 1.573062 | 6.81E-11 | 1.26E-07 | RPSAP15 | X |
| ENSG00000188856 | 151.0143 | 22.7557 | 2.731169 | 1.51E-10 | 2.48E-07 | RPSAP47 | 8 |
| ENSG00000210144 | 1.951035 | 44.4302 | -4.51765 | 3.25E-09 | 4.82E-06 | MT-TY | MT |
| ENSG00000187653 | 5633.412 | 2433.5 | 1.210942 | 6.63E-09 | 8.93E-06 | TMSB4XP8 | 4 |
| ENSG00000161970 | 533.2718 | 1519.299 | -1.51119 | 7.34E-09 | 9.07E-06 | RPL26 | 17 |
| ENSG00000140264 | 1375.266 | 3331.56 | -1.27681 | 2.88E-08 | 3.28E-05 | SERF2 | 15 |
| ENSG00000188483 | 256.6172 | 696.7528 | -1.44172 | 8.54E-08 | 9.05E-05 | IER5L | 9 |
| ENSG00000137970 | 440.2479 | 903.0671 | -1.03695 | 1.30E-07 | 0.000129 | RPL7P9 | 1 |
| ENSG00000181240 | 3.001809 | 38.82402 | -3.72348 | 1.53E-07 | 0.000142 | SLC25A41 | 19 |
| ENSG00000210082 | 69751.12 | 126748.9 | -0.86169 | 2.39E-07 | 0.000208 | MT-RNR2 | MT |
| ENSG00000213553 | 1115.827 | 2213.379 | -0.98842 | 5.10E-07 | 0.00042 | RPLP0P6 | 2 |
| ENSG00000182117 | 1413.568 | 2812.646 | -0.99273 | 6.23E-07 | 0.000486 | NOP10 | 15 |
| ENSG00000241468 | 462.4896 | 1023.202 | -1.14592 | 1.09E-06 | 0.000801 | ATP5MF | 7 |
| ENSG00000170906 | 561.4936 | 1181.812 | -1.07422 | 1.13E-06 | 0.000801 | NDUFA3 | 19 |
| ENSG00000138621 | 740.6795 | 384.66 | 0.944604 | 1.36E-06 | 0.000919 | PPCDC | 15 |
| ENSG00000249353 | 251.9325 | 510.3587 | -1.01708 | 2.56E-06 | 0.001613 | NPM1P27 | 5 |
| ENSG00000231414 | 33.27484 | 117.808 | -1.81774 | 2.73E-06 | 0.001613 | AC016700.2 | 2 |
| ENSG00000197582 | 310.4072 | 129.5739 | 1.259421 | 2.75E-06 | 0.001613 | GPX1P1 | X |
| ENSG00000221983 | 8106.858 | 14061.76 | -0.7946 | 2.83E-06 | 0.001613 | UBA52 | 19 |
| ENSG00000218175 | 1187.947 | 654.6906 | 0.859537 | 3.79E-06 | 0.00202 | AC016739.1 | 2 |
| ENSG00000127922 | 508.9419 | 879.0887 | -0.78855 | 3.82E-06 | 0.00202 | SEM1 | 7 |
| ENSG00000181649 | 3447.295 | 6276.879 | -0.86465 | 4.45E-06 | 0.002273 | PHLDA2 | 11 |
| ENSG00000232573 | 576.025 | 1280.483 | -1.15328 | 4.90E-06 | 0.002421 | RPL3P4 | 14 |
| ENSG00000213763 | 129.6297 | 51.11758 | 1.343964 | 5.32E-06 | 0.002544 | ACTBP2 | 5 |
| ENSG00000128218 | 2.548465 | 29.55539 | -3.52498 | 6.71E-06 | 0.003107 | VPREB3 | 22 |
| ENSG00000211459 | 8255.371 | 14241.43 | -0.7867 | 1.55E-05 | 0.006959 | MT-RNR1 | MT |
| ENSG00000127589 | 654.8957 | 335.8223 | 0.964111 | 1.93E-05 | 0.008402 | TUBBP1 | 8 |
| ENSG00000213326 | 353.6742 | 637.5588 | -0.85009 | 2.43E-05 | 0.010301 | RPS7P11 | 17 |
| ENSG00000241360 | 82.26499 | 194.0276 | -1.24089 | 3.07E-05 | 0.012523 | PDXP | 22 |
| ENSG00000159335 | 2136.556 | 3714.187 | -0.7979 | 3.20E-05 | 0.012523 | PTMS | 12 |
| ENSG00000170989 | 63.23249 | 15.3544 | 2.036259 | 3.21E-05 | 0.012523 | S1PR1 | 1 |
| ENSG00000088340 | 31.66617 | 99.50453 | -1.651 | 3.63E-05 | 0.01381 | FER1L4 | 20 |
| ENSG00000129450 | 0 | 14.50335 | -6.34574 | 3.94E-05 | 1 | SIGLEC9 | 19 |
| ENSG00000165502 | 757.0973 | 1383.086 | -0.86957 | 4.08E-05 | 0.015133 | RPL36AL | 14 |
| ENSG00000189343 | 1121.368 | 570.1872 | 0.97538 | 4.74E-05 | 0.017144 | RPS2P46 | 17 |
| ENSG00000181588 | 958.1729 | 1570.567 | -0.71319 | 5.12E-05 | 0.018069 | MEX3D | 19 |
| ENSG00000108342 | 258.9589 | 483.3815 | -0.90076 | 5.45E-05 | 0.018806 | CSF3 | 17 |
| ENSG00000119655 | 3015.5 | 4743.771 | -0.65371 | 5.98E-05 | 0.02014 | NPC2 | 14 |
| ENSG00000160213 | 2069.45 | 3431.448 | -0.72954 | 6.44E-05 | 0.021229 | CSTB | 21 |
| ENSG00000144713 | 16820.27 | 27886.07 | -0.72937 | 6.63E-05 | 0.02137 | RPL32 | 3 |
| ENSG00000250697 | 215.1302 | 106.0707 | 1.020036 | 6.78E-05 | 0.021373 | AC010343.3 | 5 |
| ENSG00000005243 | 67.17931 | 150.2478 | -1.16297 | 8.30E-05 | 0.02549 | COPZ2 | 17 |
| ENSG00000100650 | 1422.646 | 2252.25 | -0.66272 | 8.43E-05 | 0.02549 | SRSF5 | 14 |
| ENSG00000198712 | 74742.78 | 122004.5 | -0.70694 | 9.39E-05 | 0.027842 | MT-CO2 | MT |
| ENSG00000156467 | 2031.393 | 3147.711 | -0.63188 | 9.66E-05 | 0.028081 | UQCRB | 8 |
| ENSG00000214182 | 16.07776 | 54.3079 | -1.75295 | 0.000101 | 0.028668 | PTMAP5 | 13 |
| ENSG00000104899 | 45.07465 | 108.1036 | -1.2631 | 0.000105 | 0.029273 | AMH | 19 |
| ENSG00000234287 | 483.1308 | 861.5627 | -0.83531 | 0.000122 | 0.03351 | AC099560.2 | 3 |
| ENSG00000198938 | 85930.25 | 130042.8 | -0.59775 | 0.000137 | 0.037009 | MT-CO3 | MT |
| ENSG00000204388 | 527.2842 | 1016.46 | -0.94704 | 0.000144 | 0.038022 | HSPA1B | 6 |
| ENSG00000109971 | 20080.79 | 37135.73 | -0.88699 | 0.00016 | 0.041554 | HSPA8 | 11 |
| ENSG00000130522 | 2374.293 | 3724.872 | -0.64983 | 0.00017 | 0.042515 | JUND | 19 |
| ENSG00000244398 | 1600.866 | 2697.597 | -0.75313 | 0.000173 | 0.042515 | AC116533.1 | 11 |
| ENSG00000127528 | 319.21 | 547.5497 | -0.77902 | 0.000175 | 0.042515 | KLF2 | 19 |
| ENSG00000130881 | 1215.417 | 1951.844 | -0.68362 | 0.000177 | 0.042515 | LRP3 | 19 |
| ENSG00000198242 | 3133.439 | 5301.419 | -0.75877 | 0.000178 | 0.042515 | RPL23A | 17 |
| ENSG00000010438 | 586.4987 | 326.2512 | 0.845201 | 0.000202 | 0.047611 | PRSS3 | 9 |
| ENSG00000122674 | 172.1314 | 377.9919 | -1.13489 | 0.00022 | 0.050162 | CCZ1 | 7 |
| ENSG00000204344 | 266.0244 | 142.7783 | 0.896406 | 0.000225 | 0.050162 | STK19 | 6 |
| ENSG00000131094 | 69.8509 | 153.2429 | -1.13509 | 0.000227 | 0.050162 | C1QL1 | 17 |
| ENSG00000166073 | 382.271 | 184.6928 | 1.048392 | 0.00023 | 0.050162 | GPR176 | 15 |
| ENSG00000137834 | 26.75576 | 85.1259 | -1.66774 | 0.000232 | 0.050162 | SMAD6 | 15 |
| ENSG00000173621 | 685.4631 | 1241.713 | -0.85762 | 0.000233 | 0.050162 | LRFN4 | 11 |
| ENSG00000166337 | 54.18546 | 136.7145 | -1.33839 | 0.000241 | 0.051018 | TAF10 | 11 |
| ENSG00000163082 | 41.13838 | 11.17798 | 1.880533 | 0.000244 | 0.051018 | SGPP2 | 2 |
| ENSG00000214012 | 0 | 9.865106 | -5.78834 | 0.000256 | 1 | KRT18P38 | 6 |
| ENSG00000159167 | 104.9042 | 268.5428 | -1.3538 | 0.00026 | 0.053492 | STC1 | 8 |
| ENSG00000267279 | 507.1002 | 276.1181 | 0.87643 | 0.000285 | 0.057962 | AC090409.1 | 18 |
| ENSG00000249992 | 697.3891 | 1128.185 | -0.69426 | 0.000295 | 0.05917 | TMEM158 | 3 |
| ENSG00000180096 | 52.38398 | 115.863 | -1.1485 | 0.000302 | 0.059254 | 1-Sep | 16 |
| ENSG00000232472 | 330.7959 | 531.4643 | -0.68466 | 0.000304 | 0.059254 | EEF1B2P3 | X |
| ENSG00000175550 | 1327.786 | 2317.279 | -0.80366 | 0.000323 | 0.062256 | DRAP1 | 11 |
| ENSG00000205426 | 839.7685 | 416.5957 | 1.010635 | 0.000344 | 0.065302 | KRT81 | 12 |
| ENSG00000141522 | 3585.676 | 5993.477 | -0.74125 | 0.00035 | 0.065605 | ARHGDIA | 17 |
| ENSG00000148344 | 2569.833 | 4278.604 | -0.7356 | 0.000364 | 0.067436 | PTGES | 9 |
| ENSG00000233493 | 508.7041 | 880.623 | -0.79241 | 0.000389 | 0.07076 | TMEM238 | 19 |
| ENSG00000106211 | 3006.184 | 5237.506 | -0.80103 | 0.000391 | 0.07076 | HSPB1 | 7 |
| ENSG00000187534 | 18.84174 | 54.50234 | -1.53641 | 0.000425 | 0.075371 | PRR13P5 | 19 |
| ENSG00000051523 | 1376.615 | 2434.214 | -0.82267 | 0.000436 | 0.075371 | CYBA | 16 |
| ENSG00000176406 | 369.6036 | 218.8681 | 0.756161 | 0.000437 | 0.075371 | RIMS2 | 8 |
| ENSG00000229994 | 167.1578 | 87.58076 | 0.931299 | 0.000437 | 0.075371 | RPL5P4 | 1 |
| ENSG00000148082 | 338.5921 | 556.5833 | -0.71655 | 0.000449 | 0.076254 | SHC3 | 9 |
| ENSG00000224861 | 42.46627 | 111.0068 | -1.38925 | 0.000453 | 0.076254 | YBX1P1 | 14 |
| ENSG00000243678 | 39.43501 | 89.99297 | -1.19383 | 0.000467 | 0.077248 | NME2 | 17 |
| ENSG00000101084 | 1264.168 | 2060.201 | -0.70482 | 0.000469 | 0.077248 | RAB5IF | 20 |
| ENSG00000226948 | 0 | 9.975307 | -5.80776 | 0.000487 | 1 | RPS4XP2 | 20 |
| ENSG00000163584 | 3462.6 | 2390.366 | 0.534593 | 0.000497 | 0.080986 | RPL22L1 | 3 |
| ENSG00000138326 | 9871.033 | 15840.17 | -0.68234 | 0.000516 | 0.081408 | RPS24 | 10 |
| ENSG00000168056 | 745.5077 | 1269.418 | -0.76822 | 0.000516 | 0.081408 | LTBP3 | 11 |
| ENSG00000041982 | 613.4538 | 343.5164 | 0.835812 | 0.000516 | 0.081408 | TNC | 9 |
| ENSG00000108107 | 16109.2 | 25670.19 | -0.67224 | 0.000527 | 0.082218 | RPL28 | 19 |
| ENSG00000184058 | 164.417 | 296.5968 | -0.85226 | 0.000536 | 0.08278 | TBX1 | 22 |
| ENSG00000108984 | 9.571804 | 37.79437 | -1.97336 | 0.000549 | 0.08278 | MAP2K6 | 17 |
| ENSG00000139410 | 754.4399 | 472.1532 | 0.675596 | 0.000558 | 0.08278 | SDSL | 12 |
| ENSG00000134590 | 1091.371 | 1664.967 | -0.6095 | 0.000559 | 0.08278 | RTL8C | X |
| ENSG00000126267 | 1905.885 | 3343.484 | -0.81112 | 0.00056 | 0.08278 | COX6B1 | 19 |
| ENSG00000159884 | 111.4795 | 215.2187 | -0.94873 | 0.000567 | 0.08278 | CCDC107 | 9 |
| ENSG00000276672 | 60.29607 | 142.1852 | -1.23497 | 0.000576 | 0.08278 | AL161891.1 | 13 |
| ENSG00000100445 | 77.85358 | 33.82558 | 1.202945 | 0.000577 | 0.08278 | SDR39U1 | 14 |
| ENSG00000261040 | 219.0465 | 102.3761 | 1.095691 | 0.000582 | 0.08278 | WFDC21P | 17 |
| ENSG00000232727 | 22.54745 | 59.40184 | -1.39664 | 0.000586 | 0.08278 | YWHAEP1 | 7 |
| ENSG00000142676 | 2843.422 | 4874.608 | -0.77779 | 0.000627 | 0.087677 | RPL11 | 1 |
| ENSG00000224543 | 179.557 | 301.9994 | -0.75125 | 0.000639 | 0.088461 | SNRPGP15 | 19 |
| ENSG00000135390 | 2470.495 | 3751.415 | -0.6028 | 0.000669 | 0.091865 | ATP5MC2 | 12 |
| ENSG00000156381 | 940.1945 | 1449.984 | -0.62514 | 0.000677 | 0.092067 | ANKRD9 | 14 |
| ENSG00000183337 | 426.1953 | 658.8715 | -0.62836 | 0.000684 | 0.092145 | BCOR | X |
| ENSG00000162576 | 872.9244 | 1396.487 | -0.67841 | 0.00071 | 0.094807 | MXRA8 | 1 |
| ENSG00000136161 | 102.6217 | 44.32055 | 1.2113 | 0.000725 | 0.096023 | RCBTB2 | 13 |
| ENSG00000116106 | 332.3099 | 189.5891 | 0.809428 | 0.000756 | 0.097997 | EPHA4 | 2 |
| ENSG00000074181 | 139.3734 | 241.5361 | -0.79453 | 0.000756 | 0.097997 | NOTCH3 | 19 |
| ENSG00000079739 | 1014.177 | 1714.037 | -0.75711 | 0.000761 | 0.097997 | PGM1 | 1 |
| ENSG00000250182 | 56.33858 | 121.8199 | -1.11063 | 0.000767 | 0.097997 | EEF1A1P13 | 5 |
| ENSG00000137273 | 12.40394 | 43.6645 | -1.80958 | 0.000948 | 0.120074 | FOXF2 | 6 |
| ENSG00000148346 | 36.47602 | 80.06962 | -1.13431 | 0.000995 | 0.124991 | LCN2 | 9 |
| ENSG00000142089 | 6248.096 | 9464.089 | -0.59912 | 0.00102 | 0.127046 | IFITM3 | 11 |
| ENSG00000103260 | 988.7724 | 1579.609 | -0.6762 | 0.001045 | 0.129058 | METRN | 16 |
| ENSG00000173511 | 1395.33 | 2129.512 | -0.61014 | 0.001114 | 0.136483 | VEGFB | 11 |
| ENSG00000232389 | 24.86221 | 61.16431 | -1.29868 | 0.001157 | 0.140046 | AL583856.1 | 6 |
| ENSG00000105516 | 86.20712 | 170.889 | -0.98984 | 0.001174 | 0.140046 | DBP | 19 |
| ENSG00000107984 | 3698.637 | 2534.865 | 0.545154 | 0.00118 | 0.140046 | DKK1 | 10 |
| ENSG00000154133 | 1115.254 | 607.1541 | 0.876786 | 0.001181 | 0.140046 | ROBO4 | 11 |
| ENSG00000179967 | 217.8759 | 401.0388 | -0.88103 | 0.001194 | 0.140243 | PPP1R14BP3 | 4 |
| ENSG00000213189 | 270.0668 | 165.695 | 0.703979 | 0.001208 | 0.140243 | BTF3L4P2 | 2 |
| ENSG00000247095 | 44.55766 | 103.4823 | -1.21461 | 0.001218 | 0.140243 | MIR210HG | 11 |
| ENSG00000175592 | 5376.416 | 8403.035 | -0.64436 | 0.00122 | 0.140243 | FOSL1 | 11 |
| ENSG00000178464 | 1446.583 | 2140.078 | -0.56522 | 0.001252 | 0.141801 | RPL10P16 | 19 |
| ENSG00000103495 | 800.2765 | 1274.019 | -0.67119 | 0.001253 | 0.141801 | MAZ | 16 |
| ENSG00000259781 | 326.0094 | 570.0207 | -0.80662 | 0.001323 | 0.148572 | HMGB1P6 | 15 |
| ENSG00000264577 | 7.748716 | 30.67039 | -1.983 | 0.001346 | 0.150066 | AC010761.1 | 17 |
| ENSG00000170089 | 456.0093 | 744.2827 | -0.70762 | 0.0014 | 0.154877 | AC106795.1 | 5 |
| ENSG00000113070 | 1731.962 | 1107.261 | 0.64527 | 0.001454 | 0.158409 | HBEGF | 5 |
| ENSG00000231528 | 49.92102 | 17.99466 | 1.472095 | 0.001458 | 0.158409 | FAM225A | 9 |
| ENSG00000113578 | 118.3765 | 59.35871 | 0.995374 | 0.001464 | 0.158409 | FGF1 | 5 |
| ENSG00000240036 | 126.4968 | 228.2032 | -0.85071 | 0.001502 | 0.161323 | AC104563.1 | 11 |
| ENSG00000095752 | 694.2874 | 1221.017 | -0.81448 | 0.001521 | 0.162252 | IL11 | 19 |
| ENSG00000122026 | 8509.518 | 12424.22 | -0.54606 | 0.001542 | 0.163256 | RPL21 | 13 |
| ENSG00000143436 | 790.3916 | 1224.683 | -0.63214 | 0.001557 | 0.163723 | MRPL9 | 1 |
| ENSG00000163888 | 313.2328 | 518.4984 | -0.72836 | 0.00158 | 0.164521 | CAMK2N2 | 3 |
| ENSG00000055732 | 176.6613 | 294.3777 | -0.73566 | 0.001593 | 0.164521 | MCOLN3 | 1 |
| ENSG00000178150 | 500.137 | 935.8231 | -0.90353 | 0.001604 | 0.164521 | ZNF114 | 19 |
| ENSG00000133083 | 94.73374 | 43.40742 | 1.1275 | 0.001609 | 0.164521 | DCLK1 | 13 |
| ENSG00000156482 | 4565.713 | 2861.956 | 0.673762 | 0.001625 | 0.164904 | RPL30 | 8 |
| ENSG00000083454 | 401.5097 | 253.2427 | 0.664134 | 0.001635 | 0.164904 | P2RX5 | 17 |
| ENSG00000235363 | 210.645 | 391.1661 | -0.8949 | 0.001675 | 0.167795 | SNRPGP10 | 1 |
| ENSG00000180035 | 985.6456 | 618.3433 | 0.672145 | 0.001747 | 0.173818 | ZNF48 | 16 |
| ENSG00000145040 | 29.25633 | 75.68727 | -1.36927 | 0.001827 | 0.180182 | UCN2 | 3 |
| ENSG00000204435 | 337.5771 | 510.9781 | -0.59855 | 0.001835 | 0.180182 | CSNK2B | 6 |
| ENSG00000100836 | 257.9854 | 404.5705 | -0.64906 | 0.001868 | 0.181897 | PABPN1 | 14 |
| ENSG00000167615 | 974.411 | 1418.782 | -0.54239 | 0.001877 | 0.181897 | LENG8 | 19 |
| ENSG00000087086 | 79943.88 | 122769.3 | -0.61889 | 0.001891 | 0.182009 | FTL | 19 |
| ENSG00000014138 | 136.606 | 76.64686 | 0.832887 | 0.001914 | 0.183063 | POLA2 | 11 |
| ENSG00000128524 | 1694.817 | 2690.559 | -0.66701 | 0.001934 | 0.183811 | ATP6V1F | 7 |
| ENSG00000226221 | 295.3892 | 478.9039 | -0.69827 | 0.001988 | 0.184797 | RPL26P19 | 5 |
| ENSG00000129521 | 29.13918 | 81.12332 | -1.48014 | 0.00199 | 0.184797 | EGLN3 | 14 |
| ENSG00000070371 | 562.1713 | 361.5837 | 0.636043 | 0.001993 | 0.184797 | CLTCL1 | 22 |
| ENSG00000104529 | 1923.088 | 2884.687 | -0.58521 | 0.002011 | 0.184797 | EEF1D | 8 |
| ENSG00000083838 | 272.3436 | 154.977 | 0.811971 | 0.002027 | 0.184797 | ZNF446 | 19 |
| ENSG00000102109 | 30.7283 | 72.3536 | -1.23696 | 0.002027 | 0.184797 | PCSK1N | X |
| ENSG00000198727 | 33542.5 | 49064.31 | -0.5487 | 0.002032 | 0.184797 | MT-CYB | MT |
| ENSG00000160221 | 208.005 | 111.9033 | 0.893947 | 0.002087 | 0.188683 | GATD3A | 21 |
| ENSG00000169750 | 470.3731 | 796.4906 | -0.76063 | 0.00211 | 0.189533 | RAC3 | 17 |
| ENSG00000115255 | 358.496 | 560.1768 | -0.64507 | 0.002146 | 0.191636 | REEP6 | 19 |
| ENSG00000179111 | 75.08837 | 152.6596 | -1.02703 | 0.002161 | 0.191803 | HES7 | 17 |
| ENSG00000069869 | 2482.637 | 1441.859 | 0.783952 | 0.002192 | 0.193386 | NEDD4 | 15 |
| ENSG00000068078 | 42.5467 | 114.212 | -1.4248 | 0.002215 | 0.194329 | FGFR3 | 4 |
| ENSG00000067798 | 376.2876 | 215.7796 | 0.802611 | 0.002257 | 0.196839 | NAV3 | 12 |
| ENSG00000156990 | 1554.872 | 1064.578 | 0.546181 | 0.002277 | 0.197416 | RPUSD3 | 3 |
| ENSG00000087258 | 16.81818 | 2.400576 | 2.819377 | 0.002314 | 0.199427 | GNAO1 | 16 |
| ENSG00000228889 | 69.2509 | 30.50996 | 1.184386 | 0.002342 | 0.200652 | UBAC2-AS1 | 13 |
| ENSG00000229605 | 3.892489 | 19.27621 | -2.31107 | 0.002406 | 0.204991 | RPL21P93 | 10 |
| ENSG00000198695 | 3223.437 | 5091.929 | -0.65965 | 0.002545 | 0.215548 | MT-ND6 | MT |
| ENSG00000166707 | 39.66641 | 14.52052 | 1.449764 | 0.00257 | 0.216465 | ZCCHC18 | X |
| ENSG00000258920 | 137.7738 | 78.14901 | 0.817562 | 0.002593 | 0.216612 | FOXN3-AS1 | 14 |
| ENSG00000169249 | 64.85401 | 118.3382 | -0.86726 | 0.002601 | 0.216612 | ZRSR2 | X |
| ENSG00000253366 | 6.695884 | 0 | 5.168058 | 0.002629 | 1 | AC139272.1 | 5 |
| ENSG00000166595 | 1046.75 | 1509.618 | -0.52854 | 0.002702 | 0.223744 | CIAO2B | 16 |
| ENSG00000198888 | 10600 | 16527.86 | -0.64086 | 0.002739 | 0.225546 | MT-ND1 | MT |
| ENSG00000117984 | 1902.123 | 2927.353 | -0.62226 | 0.002795 | 0.227976 | CTSD | 11 |
| ENSG00000272115 | 2.839657 | 17.63351 | -2.61742 | 0.002799 | 0.227976 | AC233992.3 | 8 |
| ENSG00000104907 | 1191.302 | 843.2011 | 0.498289 | 0.002857 | 0.229272 | TRMT1 | 19 |
| ENSG00000182871 | 162.0022 | 297.5357 | -0.87696 | 0.002861 | 0.229272 | COL18A1 | 21 |
| ENSG00000176973 | 181.7738 | 101.1144 | 0.844281 | 0.002892 | 0.229272 | FAM89B | 11 |
| ENSG00000210140 | 69.3209 | 268.9108 | -1.95763 | 0.002895 | 0.229272 | MT-TC | MT |
| ENSG00000104763 | 1370.35 | 2060.109 | -0.58787 | 0.002903 | 0.229272 | ASAH1 | 8 |
| ENSG00000184857 | 314.6685 | 207.0653 | 0.603878 | 0.002908 | 0.229272 | TMEM186 | 16 |
| ENSG00000099860 | 1141.503 | 777.3135 | 0.553929 | 0.002927 | 0.229555 | GADD45B | 19 |
| ENSG00000189001 | 0 | 6.450173 | -5.17566 | 0.002934 | 1 | SBSN | 19 |
| ENSG00000105607 | 686.2748 | 458.4929 | 0.581411 | 0.002944 | 0.229683 | GCDH | 19 |
| ENSG00000115604 | 11.95702 | 0.994303 | 3.569075 | 0.002968 | 1 | IL18R1 | 2 |
| ENSG00000233275 | 6.982962 | 0 | 5.228976 | 0.002982 | 1 | AC009238.2 | 2 |
| ENSG00000230397 | 90.62393 | 39.35672 | 1.20455 | 0.003 | 0.232802 | SPTLC1P1 | 10 |
| ENSG00000106070 | 3253.767 | 2181.093 | 0.576964 | 0.003018 | 0.233052 | GRB10 | 7 |
| ENSG00000126522 | 639.3156 | 405.0236 | 0.6576 | 0.003042 | 0.233622 | ASL | 7 |
| ENSG00000166741 | 792.3281 | 530.6044 | 0.578082 | 0.003079 | 0.235301 | NNMT | 11 |
| ENSG00000228305 | 768.8047 | 511.5573 | 0.587727 | 0.003107 | 0.235502 | AC016734.1 | 2 |
| ENSG00000264350 | 23.43839 | 55.97908 | -1.26003 | 0.003114 | 0.235502 | SNRPGP2 | 18 |
| ENSG00000172260 | 62.62792 | 27.18709 | 1.202212 | 0.003146 | 0.235603 | NEGR1 | 1 |
| ENSG00000266074 | 366.9805 | 557.323 | -0.6038 | 0.003147 | 0.235603 | BAHCC1 | 17 |
| ENSG00000163689 | 78.92982 | 38.13551 | 1.050257 | 0.003209 | 0.23905 | C3orf67 | 3 |
| ENSG00000183474 | 174.0776 | 293.2029 | -0.75206 | 0.00327 | 0.242385 | GTF2H2C | 5 |
| ENSG00000204580 | 630.6135 | 895.7721 | -0.50661 | 0.00334 | 0.246359 | DDR1 | 6 |
| ENSG00000162772 | 307.3808 | 201.631 | 0.607871 | 0.003387 | 0.248576 | ATF3 | 1 |
| ENSG00000275765 | 34.87777 | 12.17228 | 1.519006 | 0.003441 | 0.249852 | AC091982.3 | 5 |
| ENSG00000106976 | 447.958 | 679.0798 | -0.60109 | 0.003483 | 0.249852 | DNM1 | 9 |
| ENSG00000175197 | 772.3404 | 522.0429 | 0.564527 | 0.003485 | 0.249852 | DDIT3 | 12 |
| ENSG00000171612 | 602.7623 | 415.1189 | 0.537884 | 0.003498 | 0.249852 | SLC25A33 | 1 |
| ENSG00000188486 | 4405.066 | 7017.074 | -0.67173 | 0.003516 | 0.249852 | H2AFX | 11 |
| ENSG00000187514 | 6119.479 | 9706.468 | -0.66553 | 0.00353 | 0.249852 | PTMA | 2 |
| ENSG00000227615 | 280.2706 | 427.19 | -0.60741 | 0.00354 | 0.249852 | AP001324.1 | 11 |
| ENSG00000244486 | 444.3253 | 679.0596 | -0.61292 | 0.003549 | 0.249852 | SCARF2 | 22 |
| ENSG00000212664 | 39.87209 | 83.47883 | -1.06528 | 0.003556 | 0.249852 | AC064799.1 | 15 |
| ENSG00000143552 | 13.11658 | 1.369599 | 3.268048 | 0.003616 | 1 | NUP210L | 1 |
| ENSG00000143847 | 21.26432 | 59.45299 | -1.48107 | 0.003743 | 0.261705 | PPFIA4 | 1 |
| ENSG00000077454 | 166.3161 | 279.1654 | -0.74928 | 0.003889 | 0.270636 | LRCH4 | 7 |
| ENSG00000260668 | 32.32912 | 6.442344 | 2.331587 | 0.003931 | 0.271243 | AC093536.1 | 16 |
| ENSG00000188290 | 255.3654 | 425.5012 | -0.73688 | 0.003964 | 0.271243 | HES4 | 1 |
| ENSG00000136235 | 70.4156 | 158.3484 | -1.16769 | 0.003967 | 0.271243 | GPNMB | 7 |
| ENSG00000215030 | 669.5545 | 1070.298 | -0.67753 | 0.003984 | 0.271243 | RPL13P12 | 17 |
| ENSG00000134072 | 320.7724 | 202.6116 | 0.661668 | 0.003992 | 0.271243 | CAMK1 | 3 |
| ENSG00000126698 | 2228.936 | 3085.195 | -0.46902 | 0.004032 | 0.271243 | DNAJC8 | 1 |
| ENSG00000219392 | 0 | 6.141907 | -5.10411 | 0.004035 | 1 | ZNF602P | 6 |
| ENSG00000136933 | 1482.638 | 1056.195 | 0.488991 | 0.004041 | 0.271243 | RABEPK | 9 |
| ENSG00000113368 | 1121.127 | 1756.828 | -0.64788 | 0.004061 | 0.271243 | LMNB1 | 5 |
| ENSG00000085662 | 12610.85 | 8902.047 | 0.502424 | 0.004073 | 0.271243 | AKR1B1 | 7 |
| ENSG00000251361 | 34.58343 | 10.04426 | 1.777584 | 0.004091 | 0.271243 | AC012625.1 | 5 |
| ENSG00000260428 | 10.9681 | 36.79778 | -1.75887 | 0.004099 | 0.271243 | SCX | 8 |
| ENSG00000197903 | 619.6372 | 941.8619 | -0.60487 | 0.004187 | 0.27259 | HIST1H2BK | 6 |
| ENSG00000130332 | 602.4366 | 950.5863 | -0.65844 | 0.004193 | 0.27259 | LSM7 | 19 |
| ENSG00000140990 | 1084.029 | 1653.214 | -0.60932 | 0.004203 | 0.27259 | NDUFB10 | 16 |
| ENSG00000091947 | 1939.458 | 1328.084 | 0.54631 | 0.004209 | 0.27259 | TMEM101 | 17 |
| ENSG00000130675 | 128.9469 | 216.5781 | -0.74955 | 0.004211 | 0.27259 | MNX1 | 7 |
| ENSG00000140030 | 12.49151 | 1.321662 | 3.222527 | 0.004264 | 1 | GPR65 | 14 |
| ENSG00000233461 | 87.01036 | 167.2244 | -0.94148 | 0.004357 | 0.279776 | AL445524.1 | 1 |
| ENSG00000088356 | 1939.651 | 1377.739 | 0.493483 | 0.00436 | 0.279776 | PDRG1 | 20 |
| ENSG00000226084 | 90.40668 | 151.956 | -0.74975 | 0.004387 | 0.2803 | AC113935.1 | 1 |
| ENSG00000169245 | 0.308295 | 8.929042 | -4.68122 | 0.004449 | 1 | CXCL10 | 4 |
| ENSG00000215835 | 0.980307 | 11.22688 | -3.52709 | 0.004485 | 1 | AL596087.1 | 1 |
| ENSG00000124120 | 1820.375 | 1283.873 | 0.503707 | 0.004486 | 0.285409 | TTPAL | 20 |
| ENSG00000100234 | 103 | 219.3457 | -1.09188 | 0.004529 | 0.286896 | TIMP3 | 22 |
| ENSG00000241399 | 189.0441 | 108.5291 | 0.802465 | 0.004633 | 0.289517 | CD302 | 2 |
| ENSG00000141232 | 177.9764 | 299.9741 | -0.75205 | 0.004657 | 0.289517 | TOB1 | 17 |
| ENSG00000105401 | 2834.474 | 4265.461 | -0.58979 | 0.004702 | 0.289517 | CDC37 | 19 |
| ENSG00000132646 | 3052.667 | 4252.673 | -0.47824 | 0.004712 | 0.289517 | PCNA | 20 |
| ENSG00000275895 | 327.4167 | 36.67576 | 3.15859 | 0.004729 | 0.289517 | U2AF1L5 | 21 |
| ENSG00000111186 | 563.2728 | 378.5799 | 0.57246 | 0.004729 | 0.289517 | WNT5B | 12 |
| ENSG00000135253 | 9.646386 | 30.16254 | -1.64172 | 0.004747 | 0.289517 | KCP | 7 |
| ENSG00000167470 | 1064.959 | 1462.279 | -0.45764 | 0.004748 | 0.289517 | MIDN | 19 |
| ENSG00000168874 | 94.3506 | 203.275 | -1.10809 | 0.004755 | 0.289517 | ATOH8 | 2 |
| ENSG00000173992 | 402.5399 | 261.8721 | 0.619231 | 0.004765 | 0.289517 | CCS | 11 |
| ENSG00000270069 | 161.1108 | 250.9535 | -0.63876 | 0.004818 | 0.290661 | MIR222HG | X |
| ENSG00000131650 | 46.13321 | 97.41048 | -1.08133 | 0.004845 | 0.290661 | KREMEN2 | 16 |
| ENSG00000212724 | 512.8108 | 123.7226 | 2.050984 | 0.004901 | 0.290661 | KRTAP2-3 | 17 |
| ENSG00000186907 | 203.8161 | 335.8584 | -0.72254 | 0.004911 | 0.290661 | RTN4RL2 | 11 |
| ENSG00000163319 | 326.6045 | 207.4507 | 0.654583 | 0.004915 | 0.290661 | MRPS18C | 4 |
| ENSG00000163832 | 1538.981 | 1077.854 | 0.513718 | 0.004923 | 0.290661 | ELP6 | 3 |
| ENSG00000133112 | 18370.04 | 24792.32 | -0.43256 | 0.004943 | 0.290661 | TPT1 | 13 |
| ENSG00000186468 | 1828.369 | 1292.059 | 0.500769 | 0.004955 | 0.290661 | RPS23 | 5 |
| ENSG00000173457 | 4969.012 | 7796.136 | -0.6499 | 0.004961 | 0.290661 | PPP1R14B | 11 |
| ENSG00000177706 | 543.1353 | 847.445 | -0.64257 | 0.004985 | 0.290934 | FAM20C | 7 |
| ENSG00000138675 | 1021.352 | 697.0165 | 0.551611 | 0.005005 | 0.290941 | FGF5 | 4 |
| ENSG00000118418 | 405.0228 | 656.1635 | -0.69507 | 0.005168 | 0.29837 | HMGN3 | 6 |
| ENSG00000088448 | 563.2054 | 793.6267 | -0.49442 | 0.005214 | 0.29837 | ANKRD10 | 13 |
| ENSG00000241627 | 31.81668 | 71.71564 | -1.17504 | 0.005227 | 0.29837 | UBQLN4P1 | 3 |
| ENSG00000101000 | 1610.686 | 1123.233 | 0.519785 | 0.005231 | 0.29837 | PROCR | 20 |
| ENSG00000171159 | 762.2134 | 1149.962 | -0.59393 | 0.005233 | 0.29837 | C9orf16 | 9 |
| ENSG00000129911 | 792.2368 | 1155.359 | -0.54493 | 0.005258 | 0.298649 | KLF16 | 19 |
| ENSG00000175764 | 500.4623 | 328.5258 | 0.606752 | 0.005509 | 0.31172 | TTLL11 | 9 |
| ENSG00000156966 | 0.980307 | 10.7303 | -3.46461 | 0.005547 | 1 | B3GNT7 | 2 |
| ENSG00000100084 | 255.6386 | 164.8539 | 0.632803 | 0.005604 | 0.315897 | HIRA | 22 |
| ENSG00000124143 | 331.8391 | 208.6632 | 0.668162 | 0.005756 | 0.322378 | ARHGAP40 | 20 |
| ENSG00000170381 | 107.8806 | 60.21108 | 0.842869 | 0.005763 | 0.322378 | SEMA3E | 7 |
| ENSG00000177370 | 2134.441 | 1569.142 | 0.44371 | 0.005935 | 0.330764 | TIMM22 | 17 |
| ENSG00000218426 | 28.91067 | 62.80569 | -1.1229 | 0.006069 | 0.332481 | AL590867.2 | 6 |
| ENSG00000082126 | 56.38841 | 26.04688 | 1.113875 | 0.006086 | 0.332481 | MPP4 | 2 |
| ENSG00000196878 | 4489.366 | 2828.497 | 0.666371 | 0.0061 | 0.332481 | LAMB3 | 1 |
| ENSG00000165507 | 66.45688 | 129.808 | -0.96728 | 0.006101 | 0.332481 | DEPP1 | 10 |
| ENSG00000079156 | 272.0798 | 170.0806 | 0.677295 | 0.006118 | 0.332481 | OSBPL6 | 2 |
| ENSG00000131174 | 1145.289 | 1712.976 | -0.58085 | 0.006123 | 0.332481 | COX7B | X |
| ENSG00000113141 | 841.4045 | 1194.64 | -0.50574 | 0.006152 | 0.332481 | IK | 5 |
| ENSG00000065978 | 5632.838 | 8050.89 | -0.5153 | 0.006157 | 0.332481 | YBX1 | 1 |
| ENSG00000215251 | 336.5244 | 546.3053 | -0.69855 | 0.006191 | 0.332481 | FASTKD5 | 20 |
| ENSG00000165424 | 382.1172 | 559.3953 | -0.54996 | 0.006215 | 0.332481 | ZCCHC24 | 10 |
| ENSG00000108561 | 8141.335 | 11339.21 | -0.47804 | 0.006232 | 0.332481 | C1QBP | 17 |
| ENSG00000260001 | 599.8107 | 870.7642 | -0.5385 | 0.006241 | 0.332481 | TGFBR3L | 19 |
| ENSG00000118503 | 1001.662 | 725.9254 | 0.46462 | 0.006258 | 0.332481 | TNFAIP3 | 6 |
| ENSG00000065183 | 3066.694 | 2259.661 | 0.440599 | 0.006358 | 0.335087 | WDR3 | 1 |
| ENSG00000160345 | 37.87437 | 81.54385 | -1.10883 | 0.006366 | 0.335087 | C9orf116 | 9 |
| ENSG00000177954 | 17507.45 | 24946.36 | -0.51089 | 0.006374 | 0.335087 | RPS27 | 1 |
| ENSG00000223891 | 702.2098 | 431.0058 | 0.703566 | 0.006397 | 0.335102 | OSER1-DT | 20 |
| ENSG00000267265 | 71.34859 | 33.85557 | 1.071711 | 0.006438 | 0.336023 | AC011476.3 | 19 |
| ENSG00000269388 | 35.83918 | 73.33087 | -1.03709 | 0.006486 | 0.336722 | AC018755.3 | 19 |
| ENSG00000244363 | 56.26303 | 109.7346 | -0.95968 | 0.006496 | 0.336722 | RPL7P23 | 5 |
| ENSG00000268592 | 8.603135 | 30.91661 | -1.84038 | 0.00669 | 0.34557 | RAET1E-AS1 | 6 |
| ENSG00000273763 | 31.4014 | 10.47628 | 1.585309 | 0.006776 | 0.348698 | AC007318.2 | 2 |
| ENSG00000235174 | 726.5638 | 440.2754 | 0.722061 | 0.006798 | 0.348698 | RPL39P3 | 6 |
| ENSG00000087266 | 1551.586 | 1093.199 | 0.504966 | 0.00685 | 0.349156 | SH3BP2 | 4 |
| ENSG00000128510 | 143.057 | 83.14878 | 0.784017 | 0.006854 | 0.349156 | CPA4 | 7 |
| ENSG00000105617 | 94.88496 | 180.348 | -0.92963 | 0.006898 | 0.349975 | LENG1 | 19 |
| ENSG00000268412 | 4.518659 | 18.6513 | -2.04422 | 0.00696 | 0.349975 | TRMT112P6 | 2 |
| ENSG00000198840 | 41866.57 | 56412.81 | -0.43023 | 0.006962 | 0.349975 | MT-ND3 | MT |
| ENSG00000153774 | 385.5931 | 548.3156 | -0.50743 | 0.006965 | 0.349975 | CFDP1 | 16 |
| ENSG00000116455 | 1543.048 | 1157.12 | 0.415225 | 0.007018 | 0.351466 | WDR77 | 1 |
| ENSG00000244754 | 376.4671 | 569.6821 | -0.59661 | 0.007149 | 0.356822 | N4BP2L2 | 13 |
| ENSG00000136146 | 353.1614 | 525.0094 | -0.5714 | 0.007182 | 0.357133 | MED4 | 13 |
| ENSG00000131469 | 19878.45 | 27701.43 | -0.47878 | 0.007215 | 0.357133 | RPL27 | 17 |
| ENSG00000148677 | 12187.07 | 8214.125 | 0.569155 | 0.007227 | 0.357133 | ANKRD1 | 10 |
| ENSG00000131737 | 193.5973 | 113.8588 | 0.76416 | 0.00733 | 0.360199 | KRT34 | 17 |
| ENSG00000240230 | 311.4632 | 211.106 | 0.561068 | 0.007338 | 0.360199 | COX19 | 7 |
| ENSG00000179588 | 151.2487 | 246.9956 | -0.71002 | 0.007403 | 0.362188 | ZFPM1 | 16 |
| ENSG00000180964 | 533.7511 | 744.6569 | -0.48007 | 0.007468 | 0.364165 | TCEAL8 | X |
| ENSG00000228847 | 16.30942 | 43.27391 | -1.40392 | 0.007573 | 0.368049 | ATP5MC2P4 | X |
| ENSG00000115641 | 3079.171 | 2143.126 | 0.522747 | 0.007623 | 0.369315 | FHL2 | 2 |
| ENSG00000118181 | 6220.551 | 8772.647 | -0.49602 | 0.007719 | 0.372718 | RPS25 | 11 |
| ENSG00000111775 | 1295.192 | 1856.238 | -0.51953 | 0.007771 | 0.373117 | COX6A1 | 12 |
| ENSG00000237172 | 105.4654 | 172.0386 | -0.7064 | 0.007801 | 0.373117 | B3GNT9 | 16 |
| ENSG00000177700 | 1697.762 | 2594.303 | -0.61188 | 0.007805 | 0.373117 | POLR2L | 11 |
| ENSG00000131981 | 2001.81 | 2778.488 | -0.47324 | 0.00786 | 0.373117 | LGALS3 | 14 |
| ENSG00000188368 | 26.9498 | 57.97894 | -1.10321 | 0.007865 | 0.373117 | PRR19 | 19 |
| ENSG00000154978 | 2479.12 | 1850.579 | 0.421804 | 0.007878 | 0.373117 | VOPP1 | 7 |
| ENSG00000213621 | 21.19616 | 5.415763 | 1.967369 | 0.007955 | 0.374945 | RPSAP54 | 13 |
| ENSG00000224078 | 11.33291 | 1.369599 | 3.059212 | 0.007956 | 1 | SNHG14 | 15 |
| ENSG00000126653 | 324.1241 | 461.4433 | -0.5092 | 0.007967 | 0.374945 | NSRP1 | 17 |
| ENSG00000163734 | 113.4741 | 201.8815 | -0.8311 | 0.007995 | 0.375074 | CXCL3 | 4 |
| ENSG00000100982 | 2726.83 | 1911.689 | 0.51217 | 0.008059 | 0.376786 | PCIF1 | 20 |
| ENSG00000279088 | 89.37507 | 156.557 | -0.80756 | 0.008141 | 0.376786 | AC022400.7 | 10 |
| ENSG00000185015 | 547.6094 | 364.2993 | 0.588288 | 0.008154 | 0.376786 | CA13 | 8 |
| ENSG00000102309 | 121.2908 | 199.5648 | -0.72001 | 0.008163 | 0.376786 | PIN4 | X |
| ENSG00000260781 | 24.9083 | 7.480189 | 1.73553 | 0.008167 | 0.376786 | ARHGAP23P1 | 16 |
| ENSG00000233593 | 134.7421 | 76.91273 | 0.806334 | 0.008184 | 0.376786 | AL590094.1 | 1 |
| ENSG00000184209 | 101.1018 | 172.4669 | -0.77077 | 0.008238 | 0.37715 | SNRNP35 | 12 |
| ENSG00000117122 | 77.79887 | 129.7053 | -0.73798 | 0.008252 | 0.37715 | MFAP2 | 1 |
| ENSG00000089154 | 5734.289 | 4257.496 | 0.429533 | 0.008269 | 0.37715 | GCN1 | 12 |
| ENSG00000225721 | 5.388122 | 0 | 4.855498 | 0.008311 | 1 | AL592166.1 | 1 |
| ENSG00000169230 | 3262.16 | 4386.49 | -0.42736 | 0.008329 | 0.377344 | PRELID1 | 5 |
| ENSG00000131188 | 865.2753 | 1217.184 | -0.49286 | 0.008338 | 0.377344 | PRR7 | 5 |
| ENSG00000154814 | 530.0794 | 376.373 | 0.494434 | 0.008353 | 0.377344 | OXNAD1 | 3 |
| ENSG00000183283 | 2323.035 | 3161.297 | -0.44445 | 0.008375 | 0.377344 | DAZAP2 | 12 |
| ENSG00000227008 | 73.28117 | 38.57783 | 0.925248 | 0.008577 | 0.385305 | AL009174.1 | X |
| ENSG00000237222 | 7.139649 | 0.347413 | 4.295568 | 0.008607 | 1 | LINC01968 | 3 |
| ENSG00000204498 | 756.9491 | 488.1742 | 0.63206 | 0.008683 | 0.388871 | NFKBIL1 | 6 |
| ENSG00000243449 | 1277.719 | 1734.324 | -0.44102 | 0.008721 | 0.389394 | C4orf48 | 4 |
| ENSG00000114648 | 809.1894 | 597.9909 | 0.436458 | 0.008752 | 0.389608 | KLHL18 | 3 |
| ENSG00000224126 | 21.21192 | 48.57892 | -1.19866 | 0.008809 | 0.390985 | UBE2SP2 | 17 |
| ENSG00000261512 | 13.0238 | 39.88764 | -1.62568 | 0.008969 | 0.394291 | AC092368.3 | 16 |
| ENSG00000249279 | 30.12881 | 9.41399 | 1.675224 | 0.008976 | 0.394291 | LINC02057 | 5 |
| ENSG00000167604 | 58.92208 | 104.4226 | -0.82816 | 0.008988 | 0.394291 | NFKBID | 19 |
| ENSG00000205544 | 194.3209 | 307.2371 | -0.66141 | 0.00899 | 0.394291 | TMEM256 | 17 |
| ENSG00000280789 | 212.3808 | 313.3806 | -0.56082 | 0.00906 | 0.396163 | PAGR1 | 16 |
| ENSG00000125356 | 745.7344 | 1083.484 | -0.53916 | 0.009095 | 0.396561 | NDUFA1 | X |
| ENSG00000163975 | 1958.666 | 2702.229 | -0.46448 | 0.009126 | 0.396719 | MELTF | 3 |
| ENSG00000183578 | 6.715044 | 26.09422 | -1.95118 | 0.009218 | 0.39925 | TNFAIP8L3 | 15 |
| ENSG00000081870 | 416.2686 | 642.779 | -0.62628 | 0.009256 | 0.39925 | HSPB11 | 1 |
| ENSG00000163191 | 7663.118 | 10478.94 | -0.45155 | 0.009276 | 0.39925 | S100A11 | 1 |
| ENSG00000102804 | 2442.769 | 3220.816 | -0.39887 | 0.009315 | 0.39925 | TSC22D1 | 13 |
| ENSG00000108298 | 18883.96 | 26944.43 | -0.51285 | 0.009319 | 0.39925 | RPL19 | 17 |
| ENSG00000150540 | 450.1554 | 619.5706 | -0.46043 | 0.009455 | 0.403936 | HNMT | 2 |
| ENSG00000260853 | 11.51532 | 31.94965 | -1.46874 | 0.00962 | 0.409807 | AC109460.2 | 16 |
| ENSG00000001461 | 2382.815 | 1713.676 | 0.47546 | 0.009691 | 0.411613 | NIPAL3 | 1 |
| ENSG00000109107 | 248.3443 | 424.1359 | -0.77262 | 0.009751 | 0.413016 | ALDOC | 17 |
| ENSG00000125971 | 5463.789 | 7365.702 | -0.431 | 0.009797 | 0.413334 | DYNLRB1 | 20 |
| ENSG00000175727 | 1603.571 | 1157.574 | 0.469936 | 0.009823 | 0.413334 | MLXIP | 12 |
| ENSG00000196154 | 1045.146 | 628.781 | 0.732651 | 0.009843 | 0.413334 | S100A4 | 1 |
| ENSG00000099958 | 0 | 5.075218 | -4.83035 | 0.010007 | 1 | DERL3 | 22 |
| ENSG00000180616 | 34.96013 | 73.17251 | -1.0681 | 0.010015 | 0.419392 | SSTR2 | 17 |
| ENSG00000197958 | 21017.12 | 29744.69 | -0.50109 | 0.010101 | 0.4218 | RPL12 | 9 |
| ENSG00000227440 | 0.344557 | 6.776572 | -4.28534 | 0.010179 | 1 | ATP5MC1P4 | 3 |
| ENSG00000152223 | 1649.431 | 1203.3 | 0.455079 | 0.010212 | 0.425079 | EPG5 | 18 |
| ENSG00000184924 | 414.2083 | 599.9382 | -0.53502 | 0.010237 | 0.425079 | PTRHD1 | 2 |
| ENSG00000101210 | 2477.04 | 3396.814 | -0.45575 | 0.010315 | 0.427119 | EEF1A2 | 20 |
| ENSG00000174109 | 446.431 | 317.7095 | 0.490427 | 0.010413 | 0.429665 | C16orf91 | 16 |
| ENSG00000213178 | 108.5857 | 181.7946 | -0.74277 | 0.010434 | 0.429665 | RPL22P1 | 3 |
| ENSG00000105270 | 8.724908 | 29.31282 | -1.75122 | 0.010486 | 0.429895 | CLIP3 | 19 |
| ENSG00000178401 | 107.8371 | 172.3606 | -0.67577 | 0.010498 | 0.429895 | DNAJC22 | 12 |
| ENSG00000009844 | 739.9137 | 1023.356 | -0.4675 | 0.010549 | 0.430353 | VTA1 | 6 |
| ENSG00000162542 | 618.3191 | 433.6044 | 0.511208 | 0.010567 | 0.430353 | TMCO4 | 1 |
| ENSG00000053747 | 184.1018 | 275.4885 | -0.58173 | 0.010599 | 0.430473 | LAMA3 | 18 |
| ENSG00000171408 | 4.234989 | 17.65603 | -2.0643 | 0.010685 | 0.431333 | PDE7B | 6 |
| ENSG00000277258 | 631.189 | 908.9015 | -0.52675 | 0.010695 | 0.431333 | PCGF2 | 17 |
| ENSG00000205155 | 32.242 | 68.85227 | -1.09828 | 0.010708 | 0.431333 | PSENEN | 19 |
| ENSG00000224546 | 230.0301 | 154.9704 | 0.570553 | 0.010751 | 0.431923 | EIF4BP3 | 9 |
| ENSG00000001617 | 272.0527 | 392.7947 | -0.53016 | 0.01094 | 0.437178 | SEMA3F | 3 |
| ENSG00000176978 | 1644.256 | 2398.194 | -0.54476 | 0.010969 | 0.437178 | DPP7 | 9 |
| ENSG00000107518 | 95.07695 | 55.78962 | 0.769745 | 0.010971 | 0.437178 | ATRNL1 | 10 |
| ENSG00000231993 | 12.65366 | 2.044372 | 2.63492 | 0.011046 | 1 | EP300-AS1 | 22 |
| ENSG00000168439 | 4350.734 | 6395.768 | -0.55591 | 0.011199 | 0.444872 | STIP1 | 11 |
| ENSG00000171222 | 2294.039 | 3472.932 | -0.59848 | 0.011224 | 0.444872 | SCAND1 | 20 |
| ENSG00000175305 | 50.90461 | 113.7616 | -1.1574 | 0.011258 | 0.44504 | CCNE2 | 8 |
| ENSG00000273253 | 1.951035 | 12.3117 | -2.66029 | 0.01138 | 1 | AL022328.4 | 22 |
| ENSG00000233117 | 78.96499 | 44.29307 | 0.834778 | 0.011438 | 0.448336 | LINC00702 | 10 |
| ENSG00000116285 | 5467.898 | 3557.644 | 0.620037 | 0.011465 | 0.448336 | ERRFI1 | 1 |
| ENSG00000108262 | 2273.392 | 1656.877 | 0.456182 | 0.011491 | 0.448336 | GIT1 | 17 |
| ENSG00000176102 | 401.8431 | 595.081 | -0.56568 | 0.011518 | 0.448336 | CSTF3 | 11 |
| ENSG00000178951 | 1022.385 | 1356.552 | -0.40822 | 0.011567 | 0.448336 | ZBTB7A | 19 |
| ENSG00000248333 | 494.4907 | 678.323 | -0.45623 | 0.011583 | 0.448336 | CDK11B | 1 |
| ENSG00000060642 | 779.6077 | 535.0686 | 0.542506 | 0.011592 | 0.448336 | PIGV | 1 |
| ENSG00000275719 | 40.5241 | 16.0859 | 1.327155 | 0.011607 | 0.448336 | AC008622.2 | 19 |
| ENSG00000174851 | 1837.325 | 1345.962 | 0.448678 | 0.01165 | 0.448336 | YIF1A | 11 |
| ENSG00000185222 | 311.5038 | 496.752 | -0.67192 | 0.011654 | 0.448336 | TCEAL9 | X |
| ENSG00000163382 | 3482.722 | 2547.209 | 0.451214 | 0.011702 | 0.448336 | NAXE | 1 |
| ENSG00000114473 | 49.91325 | 98.89409 | -0.98657 | 0.01171 | 0.448336 | IQCG | 3 |
| ENSG00000234975 | 19.33656 | 44.20118 | -1.19222 | 0.011735 | 0.448336 | FTH1P2 | 1 |
| ENSG00000169955 | 307.9774 | 202.2851 | 0.605842 | 0.011855 | 0.451786 | ZNF747 | 16 |
| ENSG00000099377 | 1005.99 | 735.44 | 0.45179 | 0.011901 | 0.452366 | HSD3B7 | 16 |
| ENSG00000103056 | 40.56789 | 13.61101 | 1.567569 | 0.012027 | 0.455592 | SMPD3 | 16 |
| ENSG00000166261 | 460.7597 | 331.3771 | 0.475466 | 0.012077 | 0.455592 | ZNF202 | 11 |
| ENSG00000073008 | 4303.526 | 3089.65 | 0.477969 | 0.012092 | 0.455592 | PVR | 19 |
| ENSG00000186205 | 432.9919 | 594.8659 | -0.45834 | 0.012112 | 0.455592 | 1-Mar | 1 |
| ENSG00000142677 | 0 | 4.764478 | -4.73817 | 0.012185 | 1 | IL22RA1 | 1 |
| ENSG00000198786 | 15107.54 | 21344.97 | -0.49863 | 0.012195 | 0.455592 | MT-ND5 | MT |
| ENSG00000229373 | 21.86477 | 6.341114 | 1.776739 | 0.012196 | 0.455592 | LINC00452 | 13 |
| ENSG00000165244 | 398.9583 | 578.7758 | -0.53594 | 0.012201 | 0.455592 | ZNF367 | 9 |
| ENSG00000274487 | 59.18563 | 117.769 | -0.99217 | 0.012245 | 0.456093 | AC244154.1 | 17 |
| ENSG00000185338 | 10.74095 | 28.47781 | -1.41007 | 0.012311 | 0.456379 | SOCS1 | 16 |
| ENSG00000251281 | 93.04304 | 46.57543 | 0.995565 | 0.012315 | 0.456379 | AC034223.2 | 5 |
| ENSG00000180535 | 0.308295 | 6.401275 | -4.2043 | 0.012407 | 1 | BHLHA15 | 7 |
| ENSG00000092871 | 468.0271 | 327.2375 | 0.51589 | 0.01242 | 0.459137 | RFFL | 17 |
| ENSG00000164104 | 3463.749 | 4933.73 | -0.51029 | 0.012509 | 0.460921 | HMGB2 | 4 |
| ENSG00000162591 | 179.1004 | 278.3661 | -0.63591 | 0.012554 | 0.460921 | MEGF6 | 1 |
| ENSG00000160124 | 206.0571 | 302.2452 | -0.55265 | 0.012562 | 0.460921 | CCDC58 | 3 |
| ENSG00000115468 | 218.3058 | 310.3385 | -0.50828 | 0.012605 | 0.460921 | EFHD1 | 2 |
| ENSG00000131652 | 1499.235 | 1119.168 | 0.421542 | 0.012624 | 0.460921 | THOC6 | 16 |
| ENSG00000119899 | 1496.691 | 2077.412 | -0.47278 | 0.012655 | 0.460925 | SLC17A5 | 6 |
| ENSG00000161682 | 329.7333 | 492.7179 | -0.58044 | 0.012695 | 0.461235 | FAM171A2 | 17 |
| ENSG00000175866 | 875.7175 | 1362.463 | -0.63797 | 0.012752 | 0.462198 | BAIAP2 | 17 |
| ENSG00000165655 | 195.534 | 287.0282 | -0.55474 | 0.012857 | 0.463797 | ZNF503 | 10 |
| ENSG00000162066 | 98.50008 | 163.0644 | -0.72937 | 0.012864 | 0.463797 | AMDHD2 | 16 |
| ENSG00000198331 | 543.0004 | 792.0972 | -0.54437 | 0.012935 | 0.463797 | HYLS1 | 11 |
| ENSG00000125148 | 62608.48 | 46059.9 | 0.442844 | 0.012941 | 0.463797 | MT2A | 16 |
| ENSG00000080493 | 10.84125 | 31.02068 | -1.50945 | 0.012953 | 0.463797 | SLC4A4 | 4 |
| ENSG00000120727 | 488.4549 | 686.4085 | -0.49049 | 0.012999 | 0.464323 | PAIP2 | 5 |
| ENSG00000109321 | 168.503 | 108.7247 | 0.632659 | 0.013077 | 0.465995 | AREG | 4 |
| ENSG00000157227 | 1321.384 | 1831.563 | -0.4712 | 0.01318 | 0.466892 | MMP14 | 14 |
| ENSG00000188070 | 507.7447 | 684.2649 | -0.43043 | 0.013183 | 0.466892 | C11orf95 | 11 |
| ENSG00000163682 | 12523.36 | 16392.89 | -0.38845 | 0.01329 | 0.466892 | RPL9 | 4 |
| ENSG00000256628 | 94.16602 | 55.7138 | 0.756338 | 0.013363 | 0.466892 | ZBTB11-AS1 | 3 |
| ENSG00000130165 | 480.4666 | 692.4951 | -0.52781 | 0.013373 | 0.466892 | ELOF1 | 19 |
| ENSG00000265666 | 83.74269 | 42.85319 | 0.964224 | 0.013388 | 0.466892 | RARA-AS1 | 17 |
| ENSG00000228232 | 120.3081 | 201.0005 | -0.74165 | 0.013399 | 0.466892 | GAPDHP1 | X |
| ENSG00000136295 | 1214.391 | 1664.374 | -0.45504 | 0.013419 | 0.466892 | TTYH3 | 7 |
| ENSG00000149016 | 188.6779 | 121.0633 | 0.638736 | 0.013422 | 0.466892 | TUT1 | 11 |
| ENSG00000143195 | 34.61134 | 13.48955 | 1.36204 | 0.013431 | 0.466892 | ILDR2 | 1 |
| ENSG00000131116 | 53.24469 | 111.7544 | -1.07481 | 0.013472 | 0.466892 | ZNF428 | 19 |
| ENSG00000171310 | 250.9149 | 361.8978 | -0.52884 | 0.013522 | 0.466892 | CHST11 | 12 |
| ENSG00000274615 | 1.895612 | 14.82133 | -2.95177 | 0.013544 | 0.466892 | AC233968.1 | 17 |
| ENSG00000270714 | 0 | 4.696487 | -4.72003 | 0.01357 | 1 | MINOS1P2 | 17 |
| ENSG00000127418 | 881.1466 | 1240.054 | -0.49318 | 0.013597 | 0.466892 | FGFRL1 | 4 |
| ENSG00000069424 | 1527.014 | 2025.886 | -0.40801 | 0.013604 | 0.466892 | KCNAB2 | 1 |
| ENSG00000229036 | 24.27886 | 53.74713 | -1.14143 | 0.013611 | 0.466892 | VDAC1P8 | 6 |
| ENSG00000225697 | 345.2219 | 496.9633 | -0.52577 | 0.013638 | 0.466892 | SLC26A6 | 3 |
| ENSG00000124107 | 50.11957 | 24.61959 | 1.024306 | 0.01368 | 0.467272 | SLPI | 20 |
| ENSG00000225975 | 7.394579 | 22.49844 | -1.60447 | 0.013781 | 0.46963 | LINC01534 | 19 |
| ENSG00000269954 | 0.317875 | 6.414461 | -4.20688 | 0.013866 | 1 | AC022239.1 | 8 |
| ENSG00000116525 | 64.68556 | 116.9388 | -0.8554 | 0.01403 | 0.47627 | TRIM62 | 1 |
| ENSG00000188157 | 5317.67 | 6970.527 | -0.39054 | 0.01405 | 0.47627 | AGRN | 1 |
| ENSG00000145741 | 5732.456 | 7351.374 | -0.35889 | 0.014072 | 0.47627 | BTF3 | 5 |
| ENSG00000216775 | 403.7426 | 260.4627 | 0.632177 | 0.014241 | 0.480881 | AL109918.1 | 6 |
| ENSG00000153395 | 1288.54 | 1877.364 | -0.54293 | 0.01434 | 0.483112 | LPCAT1 | 5 |
| ENSG00000197448 | 893.9414 | 1254.598 | -0.48936 | 0.014512 | 0.485095 | GSTK1 | 7 |
| ENSG00000262246 | 47.95796 | 91.65728 | -0.93947 | 0.014546 | 0.485095 | CORO7 | 16 |
| ENSG00000186834 | 1053.169 | 1427.857 | -0.43946 | 0.014561 | 0.485095 | HEXIM1 | 17 |
| ENSG00000213866 | 19.20546 | 41.50745 | -1.11589 | 0.014588 | 0.485095 | YBX1P10 | 9 |
| ENSG00000196975 | 764.1758 | 1051.994 | -0.46102 | 0.014649 | 0.485095 | ANXA4 | 2 |
| ENSG00000100116 | 585.7983 | 430.5107 | 0.443988 | 0.014655 | 0.485095 | GCAT | 22 |
| ENSG00000120896 | 913.1018 | 1317.655 | -0.52969 | 0.014656 | 0.485095 | SORBS3 | 8 |
| ENSG00000183153 | 2.902602 | 13.93627 | -2.26153 | 0.01469 | 0.485095 | GJD3 | 17 |
| ENSG00000123349 | 2360.74 | 3386.134 | -0.52061 | 0.014693 | 0.485095 | PFDN5 | 12 |
| ENSG00000164346 | 832.9849 | 1139.23 | -0.45173 | 0.014824 | 0.48832 | NSA2 | 5 |
| ENSG00000171119 | 139.2902 | 223.7208 | -0.68531 | 0.014871 | 0.488786 | NRTN | 19 |
| ENSG00000136270 | 5813.454 | 4367.172 | 0.412609 | 0.01495 | 0.489458 | TBRG4 | 7 |
| ENSG00000105825 | 449.3981 | 631.1808 | -0.48999 | 0.014957 | 0.489458 | TFPI2 | 7 |
| ENSG00000120805 | 1335.138 | 1747.456 | -0.3881 | 0.015 | 0.489785 | ARL1 | 12 |
| ENSG00000127324 | 0.317875 | 6.529427 | -4.22931 | 0.015033 | 1 | TSPAN8 | 12 |
| ENSG00000140961 | 122.335 | 229.7378 | -0.90967 | 0.015064 | 0.48992 | OSGIN1 | 16 |
| ENSG00000186642 | 430.5496 | 285.2358 | 0.593033 | 0.01507 | 0.48992 | PDE2A | 11 |
| ENSG00000101246 | 2213.252 | 1641.607 | 0.430812 | 0.015177 | 0.492293 | ARFRP1 | 20 |
| ENSG00000065060 | 1342.96 | 951.0534 | 0.497692 | 0.015257 | 0.492752 | UHRF1BP1 | 6 |
| ENSG00000198918 | 8970.187 | 11845.93 | -0.40122 | 0.015282 | 0.492752 | RPL39 | X |
| ENSG00000140259 | 542.1467 | 751.2477 | -0.47034 | 0.015313 | 0.492752 | MFAP1 | 15 |
| ENSG00000008513 | 1270.034 | 930.8787 | 0.447901 | 0.015324 | 0.492752 | ST3GAL1 | 8 |
| ENSG00000099968 | 1343.796 | 1017.286 | 0.401489 | 0.015409 | 0.494431 | BCL2L13 | 22 |
| ENSG00000204934 | 275.0786 | 180.867 | 0.603515 | 0.01545 | 0.494652 | ATP6V0E2-AS1 | 7 |
| ENSG00000183696 | 7056.87 | 5260.306 | 0.423821 | 0.015531 | 0.495422 | UPP1 | 7 |
| ENSG00000138061 | 84.64128 | 135.959 | -0.6816 | 0.015542 | 0.495422 | CYP1B1 | 2 |
| ENSG00000166582 | 535.8797 | 723.3345 | -0.43325 | 0.015588 | 0.495422 | CENPV | 17 |
| ENSG00000164077 | 813.0574 | 570.0564 | 0.511577 | 0.015607 | 0.495422 | MON1A | 3 |
| ENSG00000070501 | 525.1193 | 385.4298 | 0.446032 | 0.015682 | 0.495891 | POLB | 8 |
| ENSG00000173599 | 2401.484 | 1701.252 | 0.497092 | 0.015734 | 0.495891 | PC | 11 |
| ENSG00000154328 | 486.5031 | 358.0338 | 0.442044 | 0.015744 | 0.495891 | NEIL2 | 8 |
| ENSG00000220842 | 326.4901 | 474.9016 | -0.54147 | 0.015783 | 0.495891 | RPL21P16 | 10 |
| ENSG00000109016 | 702.2611 | 508.2569 | 0.465919 | 0.015789 | 0.495891 | DHRS7B | 17 |
| ENSG00000254978 | 4.865274 | 0 | 4.704445 | 0.015831 | 1 | ALG1L9P | 11 |
| ENSG00000115053 | 5253.375 | 7000.501 | -0.41415 | 0.015857 | 0.496957 | NCL | 2 |
| ENSG00000253683 | 25.1163 | 57.44166 | -1.20045 | 0.015983 | 0.49909 | AC027309.2 | 5 |
| ENSG00000143797 | 1133.086 | 768.1886 | 0.560851 | 0.015992 | 0.49909 | MBOAT2 | 2 |
| ENSG00000267595 | 12.12985 | 2.336019 | 2.3701 | 0.016068 | 1 | AC060780.3 | 17 |
| ENSG00000070540 | 385.8841 | 553.928 | -0.52216 | 0.016116 | 0.501601 | WIPI1 | 17 |
| ENSG00000182621 | 259.5128 | 160.1349 | 0.696339 | 0.01614 | 0.501601 | PLCB1 | 20 |
| ENSG00000204685 | 187.3257 | 109.3477 | 0.775115 | 0.016233 | 0.502638 | STARD7-AS1 | 2 |
| ENSG00000164845 | 85.23845 | 48.56193 | 0.809306 | 0.016276 | 0.502638 | FAM86FP | 12 |
| ENSG00000233762 | 642.137 | 472.3451 | 0.443024 | 0.016357 | 0.502638 | AC007969.1 | 2 |
| ENSG00000153487 | 279.5146 | 398.844 | -0.51256 | 0.016387 | 0.502638 | ING1 | 13 |
| ENSG00000198804 | 138200.2 | 187165.3 | -0.43756 | 0.016393 | 0.502638 | MT-CO1 | MT |
| ENSG00000230979 | 14.78916 | 34.91346 | -1.23885 | 0.016449 | 0.502638 | AC079250.1 | 2 |
| ENSG00000088826 | 422.5892 | 301.0233 | 0.488652 | 0.016455 | 0.502638 | SMOX | 20 |
| ENSG00000242071 | 189.03 | 291.8738 | -0.62799 | 0.016468 | 0.502638 | RPL7AP6 | 14 |
| ENSG00000261780 | 48.00684 | 21.5599 | 1.155512 | 0.016479 | 0.502638 | LINC02582 | 18 |
| ENSG00000153237 | 132.5713 | 78.32706 | 0.759473 | 0.016538 | 0.502974 | CCDC148 | 2 |
| ENSG00000159247 | 0 | 4.384786 | -4.61998 | 0.016543 | 1 | TUBBP5 | 9 |
| ENSG00000185875 | 312.859 | 208.0016 | 0.588724 | 0.016558 | 0.502974 | THNSL1 | 10 |
| ENSG00000101871 | 697.5066 | 950.3297 | -0.44565 | 0.016621 | 0.503867 | MID1 | X |
| ENSG00000126464 | 488.3878 | 702.9729 | -0.52615 | 0.016705 | 0.50537 | PRR12 | 19 |
| ENSG00000113811 | 1687.259 | 1301.261 | 0.374844 | 0.0168 | 0.505568 | SELENOK | 3 |
| ENSG00000108344 | 2081.378 | 2984.312 | -0.51997 | 0.016828 | 0.505568 | PSMD3 | 17 |
| ENSG00000157911 | 730.8262 | 532.8688 | 0.455186 | 0.016849 | 0.505568 | PEX10 | 1 |
| ENSG00000243199 | 213.0021 | 303.4513 | -0.51172 | 0.016882 | 0.505568 | AC115223.1 | 4 |
| ENSG00000178531 | 465.8515 | 664.3119 | -0.51277 | 0.016882 | 0.505568 | CTXN1 | 19 |
| ENSG00000170152 | 12.35604 | 2.025279 | 2.611482 | 0.016945 | 1 | AL391987.1 | 9 |
| ENSG00000183458 | 82.08818 | 130.2101 | -0.6674 | 0.017116 | 0.50991 | AC138932.1 | 16 |
| ENSG00000205084 | 462.4178 | 313.8666 | 0.5583 | 0.017151 | 0.50991 | TMEM231 | 16 |
| ENSG00000145912 | 575.8719 | 854.1156 | -0.56924 | 0.017216 | 0.50991 | NHP2 | 5 |
| ENSG00000273796 | 1.941455 | 10.73373 | -2.47032 | 0.017255 | 1 | BX322562.1 | 21 |
| ENSG00000165025 | 90.13356 | 139.9144 | -0.63613 | 0.017276 | 0.50991 | SYK | 9 |
| ENSG00000176845 | 110.2925 | 173.1874 | -0.65359 | 0.017297 | 0.50991 | METRNL | 17 |
| ENSG00000161677 | 734.2564 | 1008.422 | -0.45833 | 0.017301 | 0.50991 | JOSD2 | 19 |
| ENSG00000230592 | 3.892489 | 14.86354 | -1.936 | 0.017324 | 0.50991 | RPSAP8 | X |
| ENSG00000260456 | 29.34582 | 10.83839 | 1.433674 | 0.017328 | 0.50991 | C16orf95 | 16 |
| ENSG00000183010 | 4125.929 | 2634.245 | 0.647207 | 0.017343 | 0.50991 | PYCR1 | 17 |
| ENSG00000130811 | 2115.144 | 3103.335 | -0.55331 | 0.017371 | 0.50991 | EIF3G | 19 |
| ENSG00000143575 | 6371.885 | 4664.843 | 0.449796 | 0.017411 | 0.51008 | HAX1 | 1 |
| ENSG00000221869 | 709.4135 | 1007.533 | -0.50624 | 0.017491 | 0.510407 | CEBPD | 8 |
| ENSG00000165072 | 175.3121 | 117.3714 | 0.578691 | 0.017501 | 0.510407 | MAMDC2 | 9 |
| ENSG00000142528 | 668.4539 | 485.0505 | 0.46226 | 0.017533 | 0.510407 | ZNF473 | 19 |
| ENSG00000226525 | 541.0874 | 403.3555 | 0.423697 | 0.017577 | 0.510407 | RPS7P10 | 13 |
| ENSG00000282057 | 192.3128 | 121.215 | 0.665898 | 0.017594 | 0.510407 | AC092807.3 | 1 |
| ENSG00000285712 | 8.77075 | 1.026581 | 3.104169 | 0.017621 | 1 | AC068707.2 | 10 |
| ENSG00000197191 | 84.22817 | 149.5585 | -0.83245 | 0.017674 | 0.510751 | CYSRT1 | 9 |
| ENSG00000186862 | 102.5569 | 60.45269 | 0.763061 | 0.017709 | 0.510751 | PDZD7 | 10 |
| ENSG00000178773 | 247.1618 | 352.8395 | -0.51403 | 0.01771 | 0.510751 | CPNE7 | 16 |
| ENSG00000104765 | 972.944 | 1294.488 | -0.41158 | 0.017816 | 0.512825 | BNIP3L | 8 |
| ENSG00000235552 | 1369.147 | 1798.482 | -0.39373 | 0.017873 | 0.513463 | RPL6P27 | 18 |
| ENSG00000116667 | 345.8739 | 472.5532 | -0.44984 | 0.017954 | 0.514796 | C1orf21 | 1 |
| ENSG00000231770 | 96.28699 | 55.30774 | 0.800992 | 0.018031 | 0.515998 | TMEM44-AS1 | 3 |
| ENSG00000204839 | 132.6812 | 199.1595 | -0.58783 | 0.018219 | 0.519581 | MROH6 | 8 |
| ENSG00000267160 | 4.446135 | 0 | 4.578044 | 0.018227 | 1 | AC091152.2 | 17 |
| ENSG00000062282 | 213.4476 | 113.8639 | 0.905145 | 0.018279 | 0.519581 | DGAT2 | 11 |
| ENSG00000117614 | 426.9047 | 587.9212 | -0.46111 | 0.018337 | 0.519581 | SYF2 | 1 |
| ENSG00000227077 | 8.489138 | 24.0216 | -1.50617 | 0.018337 | 0.519581 | AC107983.1 | 17 |
| ENSG00000155158 | 164.3443 | 102.6693 | 0.680347 | 0.01834 | 0.519581 | TTC39B | 9 |
| ENSG00000250644 | 14.84895 | 3.066558 | 2.276787 | 0.018463 | 0.519581 | AC068580.4 | 11 |
| ENSG00000168924 | 1620.481 | 1245.144 | 0.380031 | 0.018473 | 0.519581 | LETM1 | 4 |
| ENSG00000234118 | 10.77624 | 29.01413 | -1.42093 | 0.018502 | 0.519581 | RPL13AP6 | 10 |
| ENSG00000166441 | 9392.228 | 13459.15 | -0.5191 | 0.018505 | 0.519581 | RPL27A | 11 |
| ENSG00000213683 | 14.64957 | 37.13869 | -1.3353 | 0.018524 | 0.519581 | AC002056.1 | 22 |
| ENSG00000153140 | 287.5014 | 429.0478 | -0.57644 | 0.018541 | 0.519581 | CETN3 | 5 |
| ENSG00000239246 | 15.14876 | 4.057427 | 1.901326 | 0.018587 | 0.519656 | AC008026.1 | 17 |
| ENSG00000100380 | 1695.436 | 2225.214 | -0.39216 | 0.018614 | 0.519656 | ST13 | 22 |
| ENSG00000114850 | 7414.996 | 5491.77 | 0.43318 | 0.018751 | 0.52069 | SSR3 | 3 |
| ENSG00000269176 | 15.89233 | 4.053032 | 1.974368 | 0.018784 | 0.52069 | AP001160.3 | 11 |
| ENSG00000134955 | 718.2827 | 993.1729 | -0.46811 | 0.018786 | 0.52069 | SLC37A2 | 11 |
| ENSG00000134419 | 2679.471 | 3606.051 | -0.42863 | 0.01885 | 0.52069 | RPS15A | 16 |
| ENSG00000236675 | 123.6568 | 180.7704 | -0.54783 | 0.01888 | 0.52069 | MTX1P1 | 1 |
| ENSG00000106305 | 743.2212 | 980.7625 | -0.40041 | 0.018886 | 0.52069 | AIMP2 | 7 |
| ENSG00000124641 | 1406.267 | 972.4094 | 0.532087 | 0.018929 | 0.52069 | MED20 | 6 |
| ENSG00000132793 | 181.2133 | 258.0161 | -0.50975 | 0.018932 | 0.52069 | LPIN3 | 20 |
| ENSG00000203761 | 82.03431 | 43.684 | 0.908145 | 0.018995 | 0.521359 | MSTO2P | 1 |
| ENSG00000250303 | 64.36164 | 35.20301 | 0.871914 | 0.019027 | 0.521359 | AP002884.1 | 11 |
| ENSG00000177169 | 409.574 | 287.4505 | 0.509938 | 0.019226 | 0.525181 | ULK1 | 12 |
| ENSG00000136271 | 4766.025 | 3642.457 | 0.387767 | 0.019237 | 0.525181 | DDX56 | 7 |
| ENSG00000166477 | 234.8915 | 329.7146 | -0.4887 | 0.019359 | 0.526577 | LEO1 | 15 |
| ENSG00000105393 | 1457.471 | 1092.839 | 0.415412 | 0.01937 | 0.526577 | BABAM1 | 19 |
| ENSG00000250508 | 1.305705 | 9.91112 | -2.93061 | 0.019465 | 1 | AP000808.1 | 11 |
| ENSG00000006712 | 1101.835 | 1563.396 | -0.5051 | 0.019479 | 0.526577 | PAF1 | 19 |
| ENSG00000073536 | 2025.884 | 1507.187 | 0.42645 | 0.019496 | 0.526577 | NLE1 | 17 |
| ENSG00000040608 | 201.6147 | 300.8009 | -0.57831 | 0.019499 | 0.526577 | RTN4R | 22 |
| ENSG00000155366 | 2004.395 | 2920.996 | -0.5435 | 0.019523 | 0.526577 | RHOC | 1 |
| ENSG00000258441 | 35.30347 | 66.55064 | -0.91151 | 0.019542 | 0.526577 | LINC00641 | 14 |
| ENSG00000151320 | 39.50111 | 16.50322 | 1.25727 | 0.019573 | 0.526577 | AKAP6 | 14 |
| ENSG00000116922 | 630.4947 | 467.5931 | 0.431647 | 0.019655 | 0.526673 | C1orf109 | 1 |
| ENSG00000225383 | 5684.439 | 4002.635 | 0.505989 | 0.019664 | 0.526673 | SFTA1P | 10 |
| ENSG00000151366 | 1251.125 | 925.8765 | 0.434402 | 0.019709 | 0.526673 | NDUFC2 | 11 |
| ENSG00000067829 | 391.8884 | 607.8291 | -0.63346 | 0.019718 | 0.526673 | IDH3G | X |
| ENSG00000164056 | 222.7109 | 321.4598 | -0.52825 | 0.019772 | 0.527156 | SPRY1 | 4 |
| ENSG00000112306 | 16442.88 | 20993.51 | -0.35249 | 0.019823 | 0.527573 | RPS12 | 6 |
| ENSG00000152076 | 12.83812 | 34.37312 | -1.41731 | 0.020048 | 0.531688 | CCDC74B | 2 |
| ENSG00000235374 | 39.76221 | 16.81396 | 1.239611 | 0.020049 | 0.531688 | SSR4P1 | 21 |
| ENSG00000079482 | 625.8062 | 458.4084 | 0.449162 | 0.020187 | 0.534375 | OPHN1 | X |
| ENSG00000154545 | 6.440954 | 23.19344 | -1.85036 | 0.02024 | 0.53482 | MAGED4 | X |
| ENSG00000272913 | 137.2606 | 87.12964 | 0.654374 | 0.020352 | 0.536695 | AC009237.14 | 2 |
| ENSG00000214185 | 88.88052 | 45.59509 | 0.964634 | 0.020383 | 0.536695 | XPOTP1 | 20 |
| ENSG00000169258 | 319.2465 | 456.6674 | -0.51696 | 0.020487 | 0.538319 | GPRIN1 | 5 |
| ENSG00000068654 | 2849.523 | 2237.621 | 0.348677 | 0.020518 | 0.538319 | POLR1A | 2 |
| ENSG00000163344 | 824.2405 | 606.176 | 0.442808 | 0.020556 | 0.538319 | PMVK | 1 |
| ENSG00000267761 | 8.348204 | 1.006527 | 3.047086 | 0.020604 | 1 | MIR4527HG | 18 |
| ENSG00000133226 | 814.8603 | 1089.672 | -0.41893 | 0.020654 | 0.538319 | SRRM1 | 1 |
| ENSG00000161618 | 868.0965 | 1182.577 | -0.44611 | 0.020657 | 0.538319 | ALDH16A1 | 19 |
| ENSG00000114268 | 342.3308 | 504.5544 | -0.55982 | 0.02068 | 0.538319 | PFKFB4 | 3 |
| ENSG00000115216 | 2037.091 | 2817.324 | -0.46785 | 0.020716 | 0.538319 | NRBP1 | 2 |
| ENSG00000123737 | 438.9266 | 612.2714 | -0.4794 | 0.020735 | 0.538319 | EXOSC9 | 4 |
| ENSG00000187243 | 33.92234 | 2.715711 | 3.64254 | 0.020795 | 0.538922 | MAGED4B | X |
| ENSG00000158869 | 4.227467 | 0 | 4.501023 | 0.020908 | 1 | FCER1G | 1 |
| ENSG00000233476 | 733.8389 | 950.1063 | -0.37275 | 0.020923 | 0.541306 | EEF1A1P6 | 7 |
| ENSG00000161547 | 1519.669 | 2249.852 | -0.5659 | 0.021023 | 0.542455 | SRSF2 | 17 |
| ENSG00000148110 | 1899.163 | 2584.935 | -0.44454 | 0.021071 | 0.542455 | MFSD14B | 9 |
| ENSG00000139514 | 7342.188 | 5097.791 | 0.526286 | 0.021116 | 0.542455 | SLC7A1 | 13 |
| ENSG00000119004 | 701.9727 | 495.481 | 0.503092 | 0.021178 | 0.542455 | CYP20A1 | 2 |
| ENSG00000070388 | 2.540942 | 12.0911 | -2.24077 | 0.0212 | 1 | FGF22 | 19 |
| ENSG00000236383 | 1.958557 | 10.8785 | -2.48028 | 0.021205 | 1 | CCDC200 | 17 |
| ENSG00000280046 | 2.303114 | 12.05196 | -2.40193 | 0.021222 | 1 | AC104581.4 | 17 |
| ENSG00000254531 | 94.6191 | 51.49594 | 0.873992 | 0.021236 | 0.542455 | FLJ20021 | 4 |
| ENSG00000000419 | 1039.558 | 1395.953 | -0.42508 | 0.021251 | 0.542455 | DPM1 | 20 |
| ENSG00000224858 | 97.67402 | 51.93658 | 0.910267 | 0.021295 | 0.542455 | RPL29P11 | 3 |
| ENSG00000240476 | 196.7222 | 131.7246 | 0.578739 | 0.021344 | 0.542455 | LINC00973 | 3 |
| ENSG00000275882 | 7.56631 | 27.40078 | -1.86161 | 0.021427 | 0.542455 | IKBKGP1 | X |
| ENSG00000106799 | 583.986 | 839.2637 | -0.52245 | 0.02143 | 0.542455 | TGFBR1 | 9 |
| ENSG00000174628 | 38.46853 | 68.4816 | -0.82977 | 0.02145 | 0.542455 | IQCK | 16 |
| ENSG00000140470 | 90.67312 | 52.93545 | 0.774407 | 0.021541 | 0.542455 | ADAMTS17 | 15 |
| ENSG00000109920 | 450.8 | 597.6474 | -0.40663 | 0.021546 | 0.542455 | FNBP4 | 11 |
| ENSG00000174371 | 356.4982 | 520.3257 | -0.5449 | 0.021564 | 0.542455 | EXO1 | 1 |
| ENSG00000238024 | 0 | 4.468436 | -4.64419 | 0.021616 | 1 | DDX39BP2 | 6 |
| ENSG00000166997 | 92.83144 | 150.7019 | -0.70098 | 0.02162 | 0.542455 | CNPY4 | 7 |
| ENSG00000124613 | 138.0033 | 85.70602 | 0.687457 | 0.02162 | 0.542455 | ZNF391 | 6 |
| ENSG00000131043 | 3362.989 | 2599.712 | 0.371283 | 0.021639 | 0.542455 | AAR2 | 20 |
| ENSG00000137804 | 70.03922 | 130.0702 | -0.89187 | 0.021702 | 0.542455 | NUSAP1 | 15 |
| ENSG00000158427 | 51.32318 | 22.11911 | 1.219221 | 0.021716 | 0.542455 | TMSB15B | X |
| ENSG00000088881 | 405.657 | 570.7264 | -0.4934 | 0.021724 | 0.542455 | EBF4 | 20 |
| ENSG00000249931 | 0.317875 | 5.810152 | -4.06179 | 0.021878 | 1 | GOLGA8K | 15 |
| ENSG00000138623 | 298.3588 | 211.283 | 0.49683 | 0.021917 | 0.542455 | SEMA7A | 15 |
| ENSG00000162923 | 1395.684 | 1822.044 | -0.38433 | 0.021936 | 0.542455 | WDR26 | 1 |
| ENSG00000244405 | 2940.87 | 2100.546 | 0.485389 | 0.021942 | 0.542455 | ETV5 | 3 |
| ENSG00000149474 | 714.5892 | 537.9822 | 0.409442 | 0.021963 | 0.542455 | KAT14 | 20 |
| ENSG00000198431 | 5949.21 | 7667.992 | -0.3661 | 0.021995 | 0.542455 | TXNRD1 | 12 |
| ENSG00000130311 | 1935.734 | 2523.916 | -0.38298 | 0.022011 | 0.542455 | DDA1 | 19 |
| ENSG00000213442 | 1769.436 | 1190.096 | 0.571846 | 0.022035 | 0.542455 | RPL18AP3 | 12 |
| ENSG00000232187 | 669.5401 | 468.7269 | 0.514924 | 0.022047 | 0.542455 | FTH1P7 | 13 |
| ENSG00000233913 | 552.681 | 728.4954 | -0.39835 | 0.022063 | 0.542455 | RPL10P9 | 5 |
| ENSG00000112578 | 1995.328 | 1527.269 | 0.385436 | 0.022066 | 0.542455 | BYSL | 6 |
| ENSG00000026297 | 363.0456 | 512.5976 | -0.49879 | 0.022199 | 0.544823 | RNASET2 | 6 |
| ENSG00000179988 | 246.936 | 174.4073 | 0.502446 | 0.022302 | 0.545494 | PSTK | 10 |
| ENSG00000148180 | 1154.922 | 857.2312 | 0.42978 | 0.022364 | 0.545494 | GSN | 9 |
| ENSG00000135829 | 2388.933 | 3615.649 | -0.59779 | 0.02238 | 0.545494 | DHX9 | 1 |
| ENSG00000160439 | 662.573 | 503.8916 | 0.394917 | 0.02243 | 0.545494 | RDH13 | 19 |
| ENSG00000234268 | 36.75763 | 66.15721 | -0.84646 | 0.0225 | 0.545494 | AP000936.3 | 11 |
| ENSG00000132967 | 267.1442 | 418.1873 | -0.64548 | 0.022501 | 0.545494 | HMGB1P5 | 3 |
| ENSG00000226124 | 176.7542 | 109.7289 | 0.686309 | 0.022519 | 0.545494 | FTCDNL1 | 2 |
| ENSG00000233581 | 54.85445 | 22.94918 | 1.252118 | 0.022653 | 0.545494 | AC069155.1 | 2 |
| ENSG00000171992 | 241.073 | 170.6568 | 0.497754 | 0.022678 | 0.545494 | SYNPO | 5 |
| ENSG00000196205 | 1902.57 | 2555.63 | -0.42591 | 0.022681 | 0.545494 | EEF1A1P5 | 9 |
| ENSG00000187097 | 388.2778 | 281.1373 | 0.466355 | 0.022688 | 0.545494 | ENTPD5 | 14 |
| ENSG00000122729 | 5083.335 | 4049.454 | 0.328044 | 0.022714 | 0.545494 | ACO1 | 9 |
| ENSG00000006534 | 1244.237 | 1750.118 | -0.49249 | 0.022723 | 0.545494 | ALDH3B1 | 11 |
| ENSG00000233836 | 13.67434 | 32.71247 | -1.2623 | 0.022763 | 0.545494 | AC139769.1 | 19 |
| ENSG00000176148 | 419.1962 | 303.6417 | 0.465237 | 0.022778 | 0.545494 | TCP11L1 | 11 |
| ENSG00000164161 | 72.47067 | 35.77371 | 1.015949 | 0.022834 | 0.545964 | HHIP | 4 |
| ENSG00000127325 | 61.95032 | 33.91363 | 0.869576 | 0.023026 | 0.549653 | BEST3 | 12 |
| ENSG00000229862 | 0 | 4.037373 | -4.50135 | 0.023074 | 1 | AL121972.1 | 6 |
| ENSG00000132432 | 3657.67 | 2684.874 | 0.446204 | 0.023192 | 0.552723 | SEC61G | 7 |
| ENSG00000090565 | 601.1429 | 800.3044 | -0.41319 | 0.023239 | 0.552955 | RAB11FIP3 | 16 |
| ENSG00000154767 | 122.8522 | 184.4389 | -0.5878 | 0.023313 | 0.553617 | XPC | 3 |
| ENSG00000185950 | 523.4188 | 689.3395 | -0.39725 | 0.023341 | 0.553617 | IRS2 | 13 |
| ENSG00000186017 | 191.7538 | 126.7541 | 0.598002 | 0.023387 | 0.553617 | ZNF566 | 19 |
| ENSG00000138166 | 4072.667 | 3040.047 | 0.421783 | 0.023532 | 0.553617 | DUSP5 | 10 |
| ENSG00000171227 | 3.239637 | 13.4206 | -2.05409 | 0.023626 | 0.553617 | TMEM37 | 2 |
| ENSG00000069275 | 6923.554 | 9633.192 | -0.47644 | 0.023829 | 0.553617 | NUCKS1 | 1 |
| ENSG00000163347 | 652.5482 | 415.3904 | 0.651541 | 0.023852 | 0.553617 | CLDN1 | 3 |
| ENSG00000188612 | 3865.096 | 5085.807 | -0.39592 | 0.02386 | 0.553617 | SUMO2 | 17 |
| ENSG00000136167 | 5930.147 | 7998.39 | -0.43162 | 0.023877 | 0.553617 | LCP1 | 13 |
| ENSG00000233276 | 21647.25 | 16394.16 | 0.400976 | 0.023902 | 0.553617 | GPX1 | 3 |
| ENSG00000248527 | 9693.26 | 12725.62 | -0.3927 | 0.023927 | 0.553617 | MTATP6P1 | 1 |
| ENSG00000120647 | 178.7431 | 259.7292 | -0.53849 | 0.023931 | 0.553617 | CCDC77 | 12 |
| ENSG00000244716 | 1824.475 | 2373.227 | -0.37921 | 0.023933 | 0.553617 | BX679664.3 | 1 |
| ENSG00000165985 | 14.13631 | 32.18974 | -1.1842 | 0.023994 | 0.553617 | C1QL3 | 10 |
| ENSG00000089737 | 2795.443 | 3792.287 | -0.44002 | 0.024013 | 0.553617 | DDX24 | 14 |
| ENSG00000225663 | 732.249 | 1018.536 | -0.47676 | 0.024032 | 0.553617 | MCRIP1 | 17 |
| ENSG00000253549 | 5.525649 | 0.347413 | 3.925568 | 0.024039 | 1 | CA3-AS1 | 8 |
| ENSG00000130768 | 130.2291 | 85.71344 | 0.602816 | 0.024052 | 0.553617 | SMPDL3B | 1 |
| ENSG00000106330 | 285.6306 | 414.2093 | -0.53748 | 0.024102 | 0.553617 | MOSPD3 | 7 |
| ENSG00000167004 | 2740.12 | 3713.466 | -0.43852 | 0.024139 | 0.553617 | PDIA3 | 15 |
| ENSG00000204876 | 82.36922 | 48.18357 | 0.775664 | 0.02415 | 0.553617 | AC021218.1 | 7 |
| ENSG00000139973 | 2.177225 | 13.07012 | -2.56136 | 0.024172 | 1 | SYT16 | 14 |
| ENSG00000270647 | 1032.407 | 1547.982 | -0.58428 | 0.024242 | 0.553617 | TAF15 | 17 |
| ENSG00000169583 | 184.4076 | 299.6778 | -0.70151 | 0.024245 | 0.553617 | CLIC3 | 9 |
| ENSG00000232553 | 8.028271 | 1.026581 | 2.97886 | 0.024299 | 1 | CLK2P1 | 7 |
| ENSG00000224831 | 106.2276 | 160.3703 | -0.59284 | 0.024303 | 0.553617 | TMEM183B | 3 |
| ENSG00000019485 | 514.565 | 368.961 | 0.479233 | 0.024313 | 0.553617 | PRDM11 | 11 |
| ENSG00000184076 | 1518.878 | 1994.614 | -0.39327 | 0.024383 | 0.553617 | UQCR10 | 22 |
| ENSG00000125534 | 2303.901 | 3238.993 | -0.49165 | 0.024408 | 0.553617 | PPDPF | 20 |
| ENSG00000109220 | 1131.326 | 722.1518 | 0.647362 | 0.024445 | 0.553617 | CHIC2 | 4 |
| ENSG00000135540 | 125.1352 | 79.84313 | 0.646535 | 0.024469 | 0.553617 | NHSL1 | 6 |
| ENSG00000169612 | 63.88766 | 109.9474 | -0.78554 | 0.024482 | 0.553617 | RAMAC | 15 |
| ENSG00000176490 | 26.38864 | 10.4587 | 1.335297 | 0.024651 | 0.553617 | DIRAS1 | 19 |
| ENSG00000105662 | 420.7831 | 556.705 | -0.40423 | 0.024665 | 0.553617 | CRTC1 | 19 |
| ENSG00000124243 | 963.8431 | 732.2628 | 0.396329 | 0.02467 | 0.553617 | BCAS4 | 20 |
| ENSG00000100941 | 505.0187 | 696.1759 | -0.46232 | 0.024737 | 0.553617 | PNN | 14 |
| ENSG00000173085 | 898.5498 | 696.2974 | 0.367856 | 0.02482 | 0.553617 | COQ2 | 4 |
| ENSG00000212443 | 8.391025 | 0.674773 | 3.640571 | 0.024825 | 1 | SNORA53 | 12 |
| ENSG00000163870 | 1077.435 | 1421.457 | -0.40014 | 0.024864 | 0.553617 | TPRA1 | 3 |
| ENSG00000126067 | 1735.757 | 2453.201 | -0.49913 | 0.024875 | 0.553617 | PSMB2 | 1 |
| ENSG00000279583 | 0.652852 | 6.753084 | -3.38145 | 0.024891 | 1 | AC009086.3 | 16 |
| ENSG00000090006 | 2052.086 | 2701.979 | -0.39716 | 0.024915 | 0.553617 | LTBP4 | 19 |
| ENSG00000240914 | 26.37629 | 52.49748 | -0.99127 | 0.024921 | 0.553617 | RPL15P2 | 14 |
| ENSG00000140104 | 385.7256 | 277.0432 | 0.47698 | 0.024954 | 0.553617 | CLBA1 | 14 |
| ENSG00000160801 | 96.54673 | 58.27004 | 0.726045 | 0.024971 | 0.553617 | PTH1R | 3 |
| ENSG00000275111 | 175.291 | 120.1584 | 0.544562 | 0.025003 | 0.553617 | ZNF2 | 2 |
| ENSG00000102317 | 2794.416 | 4185.302 | -0.58272 | 0.025153 | 0.553617 | RBM3 | X |
| ENSG00000081041 | 455.8878 | 706.0783 | -0.63069 | 0.025156 | 0.553617 | CXCL2 | 4 |
| ENSG00000160284 | 256.2359 | 372.4902 | -0.5413 | 0.025208 | 0.553617 | SPATC1L | 21 |
| ENSG00000181524 | 17.06765 | 37.69524 | -1.14287 | 0.025267 | 0.553617 | RPL24P4 | 6 |
| ENSG00000250794 | 20.0024 | 6.059219 | 1.721097 | 0.02528 | 0.553617 | ALG1L12P | 8 |
| ENSG00000147642 | 472.3875 | 315.3005 | 0.582718 | 0.025305 | 0.553617 | SYBU | 8 |
| ENSG00000106028 | 1509.037 | 1997.142 | -0.40455 | 0.025333 | 0.553617 | SSBP1 | 7 |
| ENSG00000114270 | 40.51768 | 79.77831 | -0.97716 | 0.025353 | 0.553617 | COL7A1 | 3 |
| ENSG00000108924 | 12.0498 | 2.703486 | 2.160278 | 0.025357 | 1 | HLF | 17 |
| ENSG00000165312 | 250.8639 | 340.0572 | -0.43872 | 0.025404 | 0.553617 | OTUD1 | 10 |
| ENSG00000168802 | 916.7684 | 1268.534 | -0.46877 | 0.025405 | 0.553617 | CHTF8 | 16 |
| ENSG00000125846 | 326.3004 | 222.7311 | 0.549502 | 0.025412 | 0.553617 | ZNF133 | 20 |
| ENSG00000089060 | 1853.473 | 1271.591 | 0.54332 | 0.025422 | 0.553617 | SLC8B1 | 12 |
| ENSG00000173692 | 1103.323 | 1705.703 | -0.62829 | 0.025542 | 0.553617 | PSMD1 | 2 |
| ENSG00000115548 | 721.7642 | 983.7466 | -0.44628 | 0.025547 | 0.553617 | KDM3A | 2 |
| ENSG00000136068 | 21093.34 | 15259.88 | 0.46702 | 0.025573 | 0.553617 | FLNB | 3 |
| ENSG00000175938 | 483.1107 | 659.6432 | -0.44981 | 0.02559 | 0.553617 | ORAI3 | 16 |
| ENSG00000090520 | 535.5005 | 710.3842 | -0.40764 | 0.025598 | 0.553617 | DNAJB11 | 3 |
| ENSG00000164051 | 880.1614 | 646.7441 | 0.444042 | 0.025602 | 0.553617 | CCDC51 | 3 |
| ENSG00000257167 | 31.04727 | 65.30077 | -1.06866 | 0.025689 | 0.553617 | TMPO-AS1 | 12 |
| ENSG00000141127 | 620.6595 | 832.1066 | -0.42254 | 0.025696 | 0.553617 | PRPSAP2 | 17 |
| ENSG00000123144 | 2436.212 | 3390.185 | -0.4769 | 0.025777 | 0.553617 | TRIR | 19 |
| ENSG00000260404 | 118.1745 | 172.9804 | -0.55045 | 0.025854 | 0.553617 | AC110079.1 | 4 |
| ENSG00000172113 | 992.9867 | 772.1776 | 0.362705 | 0.025854 | 0.553617 | NME6 | 3 |
| ENSG00000196550 | 87.24574 | 142.8699 | -0.71075 | 0.025863 | 0.553617 | FAM72A | 1 |
| ENSG00000212907 | 15489.38 | 11268.42 | 0.459003 | 0.025883 | 0.553617 | MT-ND4L | MT |
| ENSG00000162407 | 100.8696 | 176.2455 | -0.80339 | 0.025899 | 0.553617 | PLPP3 | 1 |
| ENSG00000182899 | 10271.71 | 13152.17 | -0.35666 | 0.025909 | 0.553617 | RPL35A | 3 |
| ENSG00000166444 | 395.842 | 545.5575 | -0.46299 | 0.025912 | 0.553617 | ST5 | 11 |
| ENSG00000174227 | 1088.705 | 823.3784 | 0.402802 | 0.025933 | 0.553617 | PIGG | 4 |
| ENSG00000180425 | 102.0294 | 60.33639 | 0.757131 | 0.025961 | 0.553617 | C11orf71 | 11 |
| ENSG00000241343 | 31.83012 | 60.19332 | -0.91638 | 0.025983 | 0.553617 | RPL36A | X |
| ENSG00000125798 | 460.1828 | 608.9662 | -0.40468 | 0.025993 | 0.553617 | FOXA2 | 20 |
| ENSG00000229117 | 9468.594 | 13908.49 | -0.5548 | 0.026097 | 0.554771 | RPL41 | 12 |
| ENSG00000013441 | 1136.057 | 835.6297 | 0.443168 | 0.026122 | 0.554771 | CLK1 | 2 |
| ENSG00000106366 | 7342.874 | 4841.225 | 0.600919 | 0.026201 | 0.555663 | SERPINE1 | 7 |
| ENSG00000136244 | 304.8483 | 468.9015 | -0.62061 | 0.026259 | 0.556091 | IL6 | 7 |
| ENSG00000224209 | 6.996657 | 0.666943 | 3.381925 | 0.026266 | 1 | LINC00466 | 1 |
| ENSG00000171914 | 527.8355 | 397.3908 | 0.409083 | 0.026397 | 0.556357 | TLN2 | 15 |
| ENSG00000100554 | 1408.143 | 1978.681 | -0.49061 | 0.026434 | 0.556357 | ATP6V1D | 14 |
| ENSG00000107281 | 1001.182 | 1349.057 | -0.43055 | 0.026447 | 0.556357 | NPDC1 | 9 |
| ENSG00000140854 | 995.7848 | 746.5264 | 0.41518 | 0.026458 | 0.556357 | KATNB1 | 16 |
| ENSG00000196683 | 886.2993 | 671.8331 | 0.399761 | 0.026459 | 0.556357 | TOMM7 | 7 |
| ENSG00000271966 | 5.436022 | 0.347413 | 3.905296 | 0.026476 | 1 | AC021321.1 | 8 |
| ENSG00000110492 | 796.0847 | 1097.752 | -0.46408 | 0.026512 | 0.556678 | MDK | 11 |
| ENSG00000134864 | 136.6496 | 85.83531 | 0.670542 | 0.026552 | 0.556729 | GGACT | 13 |
| ENSG00000088367 | 502.2083 | 666.5184 | -0.40894 | 0.026604 | 0.557039 | EPB41L1 | 20 |
| ENSG00000174749 | 459.6565 | 317.7791 | 0.532722 | 0.026653 | 0.557276 | FAM241A | 4 |
| ENSG00000107864 | 95.03355 | 56.97722 | 0.738417 | 0.026712 | 0.557277 | CPEB3 | 10 |
| ENSG00000158292 | 356.509 | 474.5283 | -0.41249 | 0.026772 | 0.557277 | GPR153 | 1 |
| ENSG00000136999 | 794.7394 | 1133.108 | -0.5114 | 0.026799 | 0.557277 | NOV | 8 |
| ENSG00000168826 | 91.10551 | 55.77932 | 0.70937 | 0.026829 | 0.557277 | ZBTB49 | 4 |
| ENSG00000234380 | 248.7546 | 169.4215 | 0.553563 | 0.026841 | 0.557277 | LINC01426 | 21 |
| ENSG00000124813 | 208.0794 | 287.3638 | -0.46518 | 0.026891 | 0.55753 | RUNX2 | 6 |
| ENSG00000161016 | 20505.88 | 28313.65 | -0.46548 | 0.026945 | 0.557874 | RPL8 | 8 |
| ENSG00000170291 | 1817.654 | 1396.156 | 0.380408 | 0.027017 | 0.558568 | ELP5 | 17 |
| ENSG00000157036 | 389.3092 | 288.7263 | 0.430669 | 0.027104 | 0.559078 | EXOG | 3 |
| ENSG00000183691 | 265.797 | 356.8937 | -0.42521 | 0.027117 | 0.559078 | NOG | 17 |
| ENSG00000205220 | 33.64691 | 60.84382 | -0.85887 | 0.027161 | 0.559221 | PSMB10 | 16 |
| ENSG00000177606 | 3795.651 | 2771.135 | 0.453734 | 0.027225 | 0.55975 | JUN | 1 |
| ENSG00000232229 | 20.86299 | 7.032506 | 1.565625 | 0.027304 | 0.560462 | LINC00865 | 10 |
| ENSG00000253968 | 4.678753 | 0 | 4.644052 | 0.02731 | 1 | AC016573.1 | 5 |
| ENSG00000245532 | 834.0285 | 1175.514 | -0.49465 | 0.027335 | 0.560462 | NEAT1 | 11 |
| ENSG00000086061 | 4235.056 | 5572.498 | -0.39585 | 0.027482 | 0.562694 | DNAJA1 | 9 |
| ENSG00000166886 | 561.2938 | 778.3982 | -0.47235 | 0.027567 | 0.562957 | NAB2 | 12 |
| ENSG00000142233 | 5.768942 | 17.94576 | -1.63286 | 0.02758 | 0.562957 | NTN5 | 19 |
| ENSG00000171368 | 63.36809 | 102.6752 | -0.69366 | 0.027644 | 0.562957 | TPPP | 5 |
| ENSG00000165046 | 312.9329 | 227.3055 | 0.460944 | 0.027675 | 0.562957 | LETM2 | 8 |
| ENSG00000175756 | 1141.839 | 1621.476 | -0.50621 | 0.027685 | 0.562957 | AURKAIP1 | 1 |
| ENSG00000165275 | 198.7972 | 132.1754 | 0.590411 | 0.027776 | 0.563995 | TRMT10B | 9 |
| ENSG00000004799 | 33.19877 | 59.20263 | -0.83353 | 0.027839 | 0.563995 | PDK4 | 7 |
| ENSG00000164105 | 230.3708 | 356.1095 | -0.62776 | 0.02785 | 0.563995 | SAP30 | 4 |
| ENSG00000198435 | 51.03764 | 87.91709 | -0.78599 | 0.028005 | 0.566233 | NRARP | 9 |
| ENSG00000158793 | 987.705 | 738.2125 | 0.419661 | 0.028037 | 0.566233 | NIT1 | 1 |
| ENSG00000004660 | 110.7225 | 167.2221 | -0.59515 | 0.028103 | 0.566527 | CAMKK1 | 17 |
| ENSG00000219932 | 0.317875 | 5.621838 | -4.02011 | 0.028117 | 1 | RPL12P8 | 10 |
| ENSG00000087087 | 1272.833 | 1770.933 | -0.47654 | 0.028128 | 0.566527 | SRRT | 7 |
| ENSG00000262691 | 19.08503 | 41.65817 | -1.12808 | 0.028345 | 0.569725 | AC040160.1 | 16 |
| ENSG00000143341 | 78.32171 | 47.26472 | 0.728713 | 0.028363 | 0.569725 | HMCN1 | 1 |
| ENSG00000252355 | 4.770438 | 0 | 4.669978 | 0.028485 | 1 | RN7SKP287 | 11 |
| ENSG00000129559 | 917.4745 | 1231.971 | -0.42532 | 0.028646 | 0.5739 | NEDD8 | 14 |
| ENSG00000055070 | 1596.337 | 2131.739 | -0.41751 | 0.028649 | 0.5739 | SZRD1 | 1 |
| ENSG00000131097 | 3.892489 | 0 | 4.382421 | 0.028692 | 1 | HIGD1B | 17 |
| ENSG00000145439 | 366.5263 | 255.2363 | 0.522945 | 0.029004 | 0.580229 | CBR4 | 4 |
| ENSG00000267655 | 3.982116 | 0 | 4.411345 | 0.029057 | 1 | AC125437.1 | 18 |
| ENSG00000214517 | 1213.534 | 1569.229 | -0.37094 | 0.029144 | 0.581774 | PPME1 | 11 |
| ENSG00000101280 | 47.26036 | 19.06633 | 1.305563 | 0.029159 | 0.581774 | ANGPT4 | 20 |
| ENSG00000279837 | 0.317875 | 5.091837 | -3.87258 | 0.029183 | 1 | AC112694.2 | 11 |
| ENSG00000249286 | 7.731614 | 1.006527 | 2.935891 | 0.029229 | 1 | AMD1P3 | 5 |
| ENSG00000100167 | 27.98277 | 56.59428 | -1.01659 | 0.029272 | 0.582477 | 3-Sep | 22 |
| ENSG00000151388 | 414.3289 | 280.4358 | 0.562607 | 0.029273 | 0.582477 | ADAMTS12 | 5 |
| ENSG00000079101 | 5.198194 | 0.339584 | 3.837466 | 0.029289 | 1 | CLUL1 | 18 |
| ENSG00000128000 | 183.0937 | 118.9987 | 0.623146 | 0.029499 | 0.586179 | ZNF780B | 19 |
| ENSG00000156463 | 223.7555 | 158.8014 | 0.494155 | 0.029629 | 0.587983 | SH3RF2 | 5 |
| ENSG00000285725 | 0.934465 | 7.492413 | -2.97168 | 0.029763 | 1 | AC004967.2 | 7 |
| ENSG00000239672 | 1629.504 | 1173.39 | 0.473702 | 0.029772 | 0.590023 | NME1 | 17 |
| ENSG00000174306 | 2151.677 | 1688.607 | 0.349585 | 0.029873 | 0.590723 | ZHX3 | 20 |
| ENSG00000179562 | 610.0962 | 443.9432 | 0.458349 | 0.029887 | 0.590723 | GCC1 | 7 |
| ENSG00000212864 | 248.7211 | 366.7268 | -0.56159 | 0.029932 | 0.590832 | RNF208 | 9 |
| ENSG00000183762 | 217.2177 | 314.8802 | -0.53571 | 0.029999 | 0.590878 | KREMEN1 | 22 |
| ENSG00000163814 | 3424.612 | 2661.438 | 0.363667 | 0.030014 | 0.590878 | CDCP1 | 3 |
| ENSG00000282851 | 13.22647 | 3.430591 | 1.956291 | 0.030107 | 0.591928 | BISPR | 19 |
| ENSG00000131791 | 653.0164 | 458.5778 | 0.510032 | 0.030157 | 0.592111 | PRKAB2 | 1 |
| ENSG00000160633 | 1145.257 | 1534.996 | -0.42269 | 0.030231 | 0.592776 | SAFB | 19 |
| ENSG00000089820 | 17.19148 | 37.16144 | -1.11604 | 0.030566 | 0.597213 | ARHGAP4 | X |
| ENSG00000197150 | 732.3174 | 551.2412 | 0.409877 | 0.030572 | 0.597213 | ABCB8 | 7 |
| ENSG00000124549 | 55.95351 | 90.03212 | -0.68644 | 0.030663 | 0.597213 | BTN2A3P | 6 |
| ENSG00000244462 | 1210.859 | 1643.702 | -0.44066 | 0.03067 | 0.597213 | RBM12 | 20 |
| ENSG00000167182 | 374.1852 | 505.4113 | -0.4343 | 0.030672 | 0.597213 | SP2 | 17 |
| ENSG00000214922 | 25.06293 | 50.49665 | -1.01719 | 0.030699 | 0.597213 | HLA-F-AS1 | 6 |
| ENSG00000176170 | 816.3962 | 1089.96 | -0.41713 | 0.030876 | 0.59988 | SPHK1 | 17 |
| ENSG00000180869 | 4.530297 | 0 | 4.601975 | 0.030949 | 1 | LINC01555 | 1 |
| ENSG00000238041 | 1.324865 | 8.382052 | -2.68224 | 0.030982 | 1 | AP004245.1 | 11 |
| ENSG00000114982 | 2246.388 | 1721.031 | 0.384171 | 0.030996 | 0.600959 | KANSL3 | 2 |
| ENSG00000147789 | 475.2134 | 352.6389 | 0.43025 | 0.031039 | 0.600959 | ZNF7 | 8 |
| ENSG00000100219 | 7338.378 | 4925.044 | 0.575284 | 0.031053 | 0.600959 | XBP1 | 22 |
| ENSG00000165355 | 172.8738 | 252.1594 | -0.5437 | 0.031118 | 0.601137 | FBXO33 | 14 |
| ENSG00000095397 | 117.0419 | 173.2646 | -0.56769 | 0.031144 | 0.601137 | WHRN | 9 |
| ENSG00000229097 | 8.658557 | 23.43144 | -1.43184 | 0.031212 | 0.601669 | CALM2P2 | 10 |
| ENSG00000151090 | 64.43597 | 36.80162 | 0.809431 | 0.031304 | 0.602662 | THRB | 3 |
| ENSG00000148291 | 397.8504 | 563.682 | -0.5037 | 0.031372 | 0.602764 | SURF2 | 9 |
| ENSG00000105127 | 468.6183 | 642.2555 | -0.45448 | 0.031391 | 0.602764 | AKAP8 | 19 |
| ENSG00000238035 | 6.749249 | 19.18569 | -1.50586 | 0.031485 | 0.603148 | AC138035.1 | 5 |
| ENSG00000272072 | 0.344557 | 5.108457 | -3.87672 | 0.031504 | 1 | AC004492.1 | 7 |
| ENSG00000198715 | 718.3311 | 998.2533 | -0.47496 | 0.031515 | 0.603148 | GLMP | 1 |
| ENSG00000197956 | 32294.04 | 44360.55 | -0.45802 | 0.031533 | 0.603148 | S100A6 | 1 |
| ENSG00000275720 | 4.40235 | 0 | 4.564553 | 0.031585 | 1 | AC243830.2 | 17 |
| ENSG00000003400 | 276.6858 | 202.7359 | 0.448452 | 0.031675 | 0.605094 | CASP10 | 2 |
| ENSG00000051128 | 805.7291 | 1133.924 | -0.49345 | 0.031717 | 0.605108 | HOMER3 | 19 |
| ENSG00000226781 | 22.46631 | 45.16495 | -1.00945 | 0.031818 | 0.606254 | TBCAP1 | X |
| ENSG00000215795 | 0 | 3.697789 | -4.37475 | 0.03194 | 1 | AL390728.2 | 1 |
| ENSG00000278867 | 0.944045 | 7.496809 | -2.96644 | 0.03202 | 1 | AC090616.6 | 17 |
| ENSG00000135821 | 1685.525 | 2288.26 | -0.44104 | 0.032037 | 0.608273 | GLUL | 1 |
| ENSG00000277182 | 6.814251 | 0.694827 | 3.317555 | 0.032057 | 1 | AC006449.5 | 17 |
| ENSG00000152284 | 85.408 | 126.7212 | -0.56864 | 0.032063 | 0.608273 | TCF7L1 | 2 |
| ENSG00000228363 | 0.61659 | 6.837694 | -3.42843 | 0.032079 | 1 | AC015971.1 | 2 |
| ENSG00000164117 | 344.1146 | 469.3747 | -0.44707 | 0.032123 | 0.608273 | FBXO8 | 4 |
| ENSG00000228594 | 546.9049 | 746.7676 | -0.45025 | 0.03216 | 0.608273 | FNDC10 | 1 |
| ENSG00000181163 | 18122.4 | 23318.7 | -0.3637 | 0.032208 | 0.608273 | NPM1 | 5 |
| ENSG00000169884 | 210.0712 | 149.3385 | 0.492906 | 0.03221 | 0.608273 | WNT10B | 12 |
| ENSG00000077713 | 586.4026 | 447.1706 | 0.391106 | 0.032211 | 0.608273 | SLC25A43 | X |
| ENSG00000198113 | 2926.602 | 3773.321 | -0.36673 | 0.032396 | 0.610985 | TOR4A | 9 |
| ENSG00000237330 | 0 | 3.705618 | -4.37739 | 0.032515 | 1 | RNF223 | 1 |
| ENSG00000173163 | 269.8333 | 189.0236 | 0.513376 | 0.032533 | 0.6128 | COMMD1 | 2 |
| ENSG00000136490 | 244.5679 | 341.9394 | -0.48496 | 0.032625 | 0.613081 | LIMD2 | 17 |
| ENSG00000145982 | 747.3532 | 572.637 | 0.38371 | 0.032684 | 0.613081 | FARS2 | 6 |
| ENSG00000186283 | 1700.742 | 1299.835 | 0.387789 | 0.0327 | 0.613081 | TOR3A | 1 |
| ENSG00000100014 | 600.8919 | 423.5625 | 0.504274 | 0.032786 | 0.613081 | SPECC1L | 22 |
| ENSG00000167106 | 602.9196 | 790.3022 | -0.3908 | 0.032855 | 0.613081 | FAM102A | 9 |
| ENSG00000185885 | 29.74824 | 62.44815 | -1.07462 | 0.032868 | 0.613081 | IFITM1 | 11 |
| ENSG00000120162 | 111.557 | 73.1036 | 0.609907 | 0.032874 | 0.613081 | MOB3B | 9 |
| ENSG00000126870 | 172.2584 | 243.1335 | -0.49571 | 0.032879 | 0.613081 | WDR60 | 7 |
| ENSG00000161082 | 6.741727 | 0.694827 | 3.304218 | 0.032909 | 1 | CELF5 | 19 |
| ENSG00000137547 | 1855.422 | 1471.955 | 0.333906 | 0.032989 | 0.61389 | MRPL15 | 8 |
| ENSG00000067221 | 511.5847 | 381.1898 | 0.423649 | 0.033036 | 0.61389 | STOML1 | 15 |
| ENSG00000101439 | 4827.541 | 6295.271 | -0.38308 | 0.033056 | 0.61389 | CST3 | 20 |
| ENSG00000182768 | 1217.151 | 958.3719 | 0.344661 | 0.033108 | 0.61389 | NGRN | 15 |
| ENSG00000188766 | 302.6699 | 415.0578 | -0.45678 | 0.03313 | 0.61389 | SPRED3 | 19 |
| ENSG00000108551 | 23.34876 | 46.26447 | -0.98678 | 0.033202 | 0.614472 | RASD1 | 17 |
| ENSG00000066379 | 558.5446 | 406.3732 | 0.458475 | 0.033285 | 0.615237 | ZNRD1 | 6 |
| ENSG00000131018 | 573.0609 | 400.1055 | 0.518293 | 0.033386 | 0.615608 | SYNE1 | 6 |
| ENSG00000168878 | 7.573833 | 1.006527 | 2.901585 | 0.033388 | 1 | SFTPB | 2 |
| ENSG00000060656 | 221.0703 | 321.3429 | -0.54109 | 0.033437 | 0.615608 | PTPRU | 1 |
| ENSG00000184992 | 585.4555 | 784.7594 | -0.42214 | 0.033463 | 0.615608 | BRI3BP | 12 |
| ENSG00000257671 | 62.03049 | 35.35236 | 0.810915 | 0.033474 | 0.615608 | KRT7-AS | 12 |
| ENSG00000183161 | 511.7101 | 381.4893 | 0.423506 | 0.033522 | 0.615608 | FANCF | 11 |
| ENSG00000163867 | 237.0086 | 157.4106 | 0.591231 | 0.0336 | 0.615608 | ZMYM6 | 1 |
| ENSG00000178038 | 657.7724 | 497.875 | 0.40135 | 0.033608 | 0.615608 | ALS2CL | 3 |
| ENSG00000083807 | 228.6372 | 156.7587 | 0.542679 | 0.033675 | 0.615608 | SLC27A5 | 19 |
| ENSG00000174365 | 804.7056 | 570.7575 | 0.495137 | 0.033679 | 0.615608 | SNHG11 | 20 |
| ENSG00000125657 | 137.9263 | 223.9416 | -0.69928 | 0.033884 | 0.618588 | TNFSF9 | 19 |
| ENSG00000178404 | 5.137307 | 0.339584 | 3.822948 | 0.033887 | 1 | CEP295NL | 17 |
| ENSG00000236017 | 0.62617 | 6.356772 | -3.31768 | 0.033903 | 1 | ASMTL-AS1 | X |
| ENSG00000107331 | 1238.994 | 1635.885 | -0.40122 | 0.034172 | 0.622722 | ABCA2 | 9 |
| ENSG00000275496 | 100.214 | 62.15555 | 0.691652 | 0.034194 | 0.622722 | CU633906.1 | 21 |
| ENSG00000153292 | 20.43221 | 6.713937 | 1.604649 | 0.034393 | 0.624346 | ADGRF1 | 6 |
| ENSG00000162231 | 1182.204 | 1576.015 | -0.41493 | 0.034396 | 0.624346 | NXF1 | 11 |
| ENSG00000285793 | 110.6074 | 66.91338 | 0.722629 | 0.034503 | 0.624346 | AC125232.2 | 2 |
| ENSG00000138759 | 972.5812 | 739.9111 | 0.394317 | 0.034544 | 0.624346 | FRAS1 | 4 |
| ENSG00000203883 | 28.74852 | 53.45419 | -0.89616 | 0.034551 | 0.624346 | SOX18 | 20 |
| ENSG00000247746 | 19.07957 | 7.060389 | 1.431018 | 0.034553 | 0.624346 | USP51 | X |
| ENSG00000214655 | 437.197 | 599.791 | -0.45673 | 0.034578 | 0.624346 | ZSWIM8 | 10 |
| ENSG00000213985 | 7.584507 | 1.022186 | 2.902467 | 0.03463 | 1 | AC078899.1 | 19 |
| ENSG00000164885 | 407.0282 | 569.7195 | -0.48532 | 0.034706 | 0.625849 | CDK5 | 7 |
| ENSG00000149541 | 737.8725 | 1004.804 | -0.44573 | 0.034854 | 0.625849 | B3GAT3 | 11 |
| ENSG00000036448 | 219.6158 | 144.3664 | 0.604439 | 0.034867 | 0.625849 | MYOM2 | 8 |
| ENSG00000143198 | 2276.626 | 2988.847 | -0.39281 | 0.034902 | 0.625849 | MGST3 | 1 |
| ENSG00000232442 | 395.2082 | 284.068 | 0.475243 | 0.034935 | 0.625849 | MHENCR | 20 |
| ENSG00000204219 | 432.6154 | 570.3334 | -0.39938 | 0.03494 | 0.625849 | TCEA3 | 1 |
| ENSG00000155097 | 1103.811 | 1459.966 | -0.40304 | 0.034991 | 0.625849 | ATP6V1C1 | 8 |
| ENSG00000236896 | 7.358317 | 1.026581 | 2.853694 | 0.035009 | 1 | AL354726.1 | 9 |
| ENSG00000230583 | 10.02515 | 1.737066 | 2.54673 | 0.035019 | 1 | GTF2IRD1P1 | 7 |
| ENSG00000189223 | 2108.604 | 1615.662 | 0.383956 | 0.035039 | 0.625849 | PAX8-AS1 | 2 |
| ENSG00000280374 | 23.97809 | 9.359735 | 1.354322 | 0.035046 | 0.625849 | AC019080.5 | 2 |
| ENSG00000167747 | 4454.459 | 3426.429 | 0.378425 | 0.03512 | 0.625849 | C19orf48 | 19 |
| ENSG00000242960 | 78.90667 | 130.7857 | -0.72527 | 0.035126 | 0.625849 | FTH1P23 | 3 |
| ENSG00000112855 | 553.5746 | 418.1407 | 0.404285 | 0.035257 | 0.626785 | HARS2 | 5 |
| ENSG00000171703 | 1571.731 | 2088.951 | -0.4107 | 0.035263 | 0.626785 | TCEA2 | 20 |
| ENSG00000234630 | 3.783703 | 0 | 4.346435 | 0.035429 | 1 | AC245060.2 | 22 |
| ENSG00000128482 | 12.95443 | 29.3978 | -1.18363 | 0.035439 | 0.629154 | RNF112 | 17 |
| ENSG00000260081 | 2.683934 | 12.06418 | -2.19212 | 0.035458 | 1 | AF274858.1 | X |
| ENSG00000185477 | 0 | 3.746687 | -4.39143 | 0.035563 | 1 | GPRIN3 | 4 |
| ENSG00000205213 | 802.5047 | 1075.307 | -0.42166 | 0.035592 | 0.629868 | LGR4 | 11 |
| ENSG00000167895 | 134.3627 | 223.8366 | -0.73768 | 0.035598 | 0.629868 | TMC8 | 17 |
| ENSG00000166803 | 423.2866 | 625.3754 | -0.56223 | 0.035659 | 0.629868 | PCLAF | 15 |
| ENSG00000133026 | 872.4014 | 1144.856 | -0.39178 | 0.035731 | 0.629868 | MYH10 | 17 |
| ENSG00000167377 | 31.11966 | 12.78442 | 1.278976 | 0.035739 | 0.629868 | ZNF23 | 16 |
| ENSG00000237758 | 15.31708 | 33.56086 | -1.13462 | 0.035878 | 0.629868 | BANF1P3 | 2 |
| ENSG00000116062 | 881.892 | 1129.94 | -0.35722 | 0.035953 | 0.629868 | MSH6 | 2 |
| ENSG00000107651 | 489.4799 | 659.2892 | -0.42901 | 0.035973 | 0.629868 | SEC23IP | 10 |
| ENSG00000141380 | 716.8645 | 968.2019 | -0.43313 | 0.035986 | 0.629868 | SS18 | 18 |
| ENSG00000272463 | 38.1783 | 18.42975 | 1.046822 | 0.036002 | 0.629868 | AL357054.4 | 6 |
| ENSG00000172366 | 750.2846 | 1011.379 | -0.43147 | 0.036008 | 0.629868 | MCRIP2 | 16 |
| ENSG00000255737 | 17.33241 | 5.726503 | 1.593051 | 0.036048 | 0.629868 | AGAP2-AS1 | 12 |
| ENSG00000145050 | 901.1171 | 1179.771 | -0.38908 | 0.036116 | 0.629868 | MANF | 3 |
| ENSG00000143674 | 324.5975 | 440.1484 | -0.43853 | 0.036181 | 0.629868 | MAP3K21 | 1 |
| ENSG00000174028 | 45.28824 | 75.71405 | -0.73862 | 0.03619 | 0.629868 | FAM3C2 | X |
| ENSG00000187642 | 10.26092 | 24.51932 | -1.25498 | 0.036195 | 0.629868 | PERM1 | 1 |
| ENSG00000110660 | 2303.802 | 1751.306 | 0.395582 | 0.036201 | 0.629868 | SLC35F2 | 11 |
| ENSG00000197102 | 12510.09 | 10060.76 | 0.314339 | 0.03625 | 0.629969 | DYNC1H1 | 14 |
| ENSG00000168884 | 1972.67 | 1549.953 | 0.34771 | 0.036297 | 0.630014 | TNIP2 | 4 |
| ENSG00000158966 | 213.116 | 153.869 | 0.470111 | 0.036358 | 0.630014 | CACHD1 | 1 |
| ENSG00000181852 | 3733.25 | 2624.071 | 0.508516 | 0.036449 | 0.630014 | RNF41 | 12 |
| ENSG00000168495 | 782.5847 | 602.3094 | 0.377389 | 0.036458 | 0.630014 | POLR3D | 8 |
| ENSG00000243335 | 804.9061 | 629.7274 | 0.354149 | 0.036499 | 0.630014 | KCTD7 | 7 |
| ENSG00000074219 | 392.8738 | 548.3491 | -0.4812 | 0.036507 | 0.630014 | TEAD2 | 19 |
| ENSG00000235530 | 7.884185 | 1.04224 | 2.937352 | 0.036545 | 1 | AC087294.1 | 17 |
| ENSG00000129422 | 48.8147 | 82.38005 | -0.75072 | 0.03659 | 0.630701 | MTUS1 | 8 |
| ENSG00000177971 | 1320.093 | 1028.512 | 0.360202 | 0.036662 | 0.631042 | IMP3 | 15 |
| ENSG00000006756 | 236.1756 | 169.4255 | 0.478821 | 0.036694 | 0.631042 | ARSD | X |
| ENSG00000141526 | 3951.101 | 5401.114 | -0.45105 | 0.036741 | 0.631104 | SLC16A3 | 17 |
| ENSG00000115267 | 42.34186 | 82.4073 | -0.95785 | 0.03683 | 0.631104 | IFIH1 | 2 |
| ENSG00000160211 | 1126.339 | 1474.826 | -0.38928 | 0.036853 | 0.631104 | G6PD | X |
| ENSG00000105865 | 192.0888 | 132.62 | 0.535536 | 0.036868 | 0.631104 | DUS4L | 7 |
| ENSG00000185634 | 2.938864 | 11.57429 | -1.98301 | 0.036996 | 1 | SHC4 | 15 |
| ENSG00000100359 | 4.907001 | 0.347413 | 3.752888 | 0.036999 | 1 | SGSM3 | 22 |
| ENSG00000259803 | 35.18607 | 60.0898 | -0.77226 | 0.037016 | 0.631233 | SLC22A31 | 16 |
| ENSG00000145247 | 1493.449 | 1875.423 | -0.3286 | 0.037061 | 0.631233 | OCIAD2 | 4 |
| ENSG00000134716 | 25.19281 | 9.34751 | 1.427698 | 0.037093 | 0.631233 | CYP2J2 | 1 |
| ENSG00000168310 | 267.8863 | 361.2089 | -0.43085 | 0.03716 | 0.631233 | IRF2 | 4 |
| ENSG00000260588 | 24.77939 | 9.34751 | 1.401849 | 0.037183 | 0.631233 | AC027702.1 | 8 |
| ENSG00000168661 | 78.11674 | 47.8715 | 0.705693 | 0.037187 | 0.631233 | ZNF30 | 19 |
| ENSG00000105711 | 70.96995 | 117.0103 | -0.72516 | 0.037235 | 0.631233 | SCN1B | 19 |
| ENSG00000197119 | 663.6352 | 874.8447 | -0.39925 | 0.037257 | 0.631233 | SLC25A29 | 14 |
| ENSG00000080854 | 36.9934 | 18.14249 | 1.026291 | 0.037276 | 0.631233 | IGSF9B | 11 |
| ENSG00000159917 | 32.87338 | 14.00046 | 1.226578 | 0.037302 | 0.631233 | ZNF235 | 19 |
| ENSG00000242951 | 21.20439 | 41.67667 | -0.97824 | 0.037621 | 0.63457 | AC007182.2 | 14 |
| ENSG00000275120 | 0.308295 | 4.740029 | -3.76982 | 0.037715 | 1 | AC048382.5 | 15 |
| ENSG00000143878 | 248.0457 | 341.8036 | -0.4628 | 0.037731 | 0.63457 | RHOB | 2 |
| ENSG00000099899 | 1150.104 | 887.4854 | 0.373628 | 0.037735 | 0.63457 | TRMT2A | 22 |
| ENSG00000232385 | 26.99153 | 12.17668 | 1.150132 | 0.037739 | 0.63457 | RPS3AP25 | 7 |
| ENSG00000163931 | 5643.995 | 7713.629 | -0.45073 | 0.037742 | 0.63457 | TKT | 3 |
| ENSG00000108433 | 920.7306 | 723.9584 | 0.34687 | 0.037834 | 0.63457 | GOSR2 | 17 |
| ENSG00000167797 | 1117.005 | 1534.822 | -0.45864 | 0.037886 | 0.63457 | CDK2AP2 | 11 |
| ENSG00000119705 | 1656.041 | 1255.121 | 0.399957 | 0.037937 | 0.63457 | SLIRP | 14 |
| ENSG00000213468 | 34.577 | 17.1839 | 1.007216 | 0.038022 | 0.63457 | FIRRE | X |
| ENSG00000116809 | 608.1838 | 444.3925 | 0.451847 | 0.038053 | 0.63457 | ZBTB17 | 1 |
| ENSG00000231365 | 313.8244 | 228.9937 | 0.454426 | 0.038066 | 0.63457 | AL359915.2 | 1 |
| ENSG00000135747 | 3.601297 | 0 | 4.268531 | 0.038071 | 1 | ZNF670-ZNF695 | 1 |
| ENSG00000177508 | 93.54737 | 137.8486 | -0.55959 | 0.038106 | 0.63457 | IRX3 | 16 |
| ENSG00000136819 | 668.9336 | 864.3946 | -0.37014 | 0.038118 | 0.63457 | C9orf78 | 9 |
| ENSG00000196465 | 448.1614 | 600.6114 | -0.42325 | 0.03816 | 0.63457 | MYL6B | 12 |
| ENSG00000184588 | 10.12545 | 26.21225 | -1.36244 | 0.038167 | 0.63457 | PDE4B | 1 |
| ENSG00000230102 | 20.66798 | 7.750821 | 1.417233 | 0.038287 | 0.63457 | LINC02028 | 3 |
| ENSG00000266258 | 6.159341 | 0.666943 | 3.19408 | 0.038299 | 1 | LINC01909 | 18 |
| ENSG00000065802 | 2224.498 | 1645.662 | 0.434689 | 0.038305 | 0.63457 | ASB1 | 2 |
| ENSG00000064225 | 78.06074 | 124.5642 | -0.67139 | 0.038331 | 0.63457 | ST3GAL6 | 3 |
| ENSG00000122257 | 671.2651 | 863.3987 | -0.3627 | 0.038338 | 0.63457 | RBBP6 | 16 |
| ENSG00000226085 | 240.4303 | 168.4433 | 0.51345 | 0.038355 | 0.63457 | UQCRFS1P1 | 22 |
| ENSG00000262786 | 0.344557 | 4.776703 | -3.77942 | 0.038388 | 1 | AC005224.2 | 17 |
| ENSG00000102753 | 934.1928 | 1261.829 | -0.43322 | 0.038449 | 0.635419 | KPNA3 | 13 |
| ENSG00000100906 | 1160.141 | 1552.855 | -0.42082 | 0.038556 | 0.635935 | NFKBIA | 14 |
| ENSG00000111674 | 1755.99 | 2438.672 | -0.47378 | 0.038566 | 0.635935 | ENO2 | 12 |
| ENSG00000102174 | 0.317875 | 4.776703 | -3.77943 | 0.038626 | 1 | PHEX | X |
| ENSG00000105737 | 0.317875 | 4.784532 | -3.78147 | 0.038706 | 1 | GRIK5 | 19 |
| ENSG00000060709 | 2.830077 | 11.51413 | -2.00781 | 0.03878 | 1 | RIMBP2 | 12 |
| ENSG00000179085 | 1326.91 | 995.0597 | 0.414766 | 0.038828 | 0.638476 | DPM3 | 1 |
| ENSG00000116754 | 913.5027 | 1221.092 | -0.41817 | 0.038862 | 0.638476 | SRSF11 | 1 |
| ENSG00000126249 | 542.0127 | 418.7162 | 0.371922 | 0.038931 | 0.638476 | PDCD2L | 19 |
| ENSG00000261428 | 3.239637 | 12.25153 | -1.91929 | 0.03896 | 1 | AC097461.1 | 2 |
| ENSG00000174720 | 327.4023 | 430.3514 | -0.39413 | 0.038986 | 0.638476 | LARP7 | 4 |
| ENSG00000103254 | 612.9117 | 465.4907 | 0.396447 | 0.039017 | 0.638476 | FAM173A | 16 |
| ENSG00000207870 | 0.308295 | 4.699921 | -3.75932 | 0.03904 | 1 | MIR221 | X |
| ENSG00000077274 | 32.42003 | 15.2843 | 1.088489 | 0.039047 | 0.638476 | CAPN6 | X |
| ENSG00000105204 | 333.3894 | 446.3077 | -0.42156 | 0.039144 | 0.638476 | DYRK1B | 19 |
| ENSG00000167720 | 142.9239 | 198.7171 | -0.47467 | 0.039187 | 0.638476 | SRR | 17 |
| ENSG00000113328 | 1129.596 | 1480.247 | -0.38962 | 0.039194 | 0.638476 | CCNG1 | 5 |
| ENSG00000137824 | 868.1558 | 665.9567 | 0.382236 | 0.039225 | 0.638476 | RMDN3 | 15 |
| ENSG00000237523 | 640.1218 | 494.437 | 0.372286 | 0.039229 | 0.638476 | LINC00857 | 10 |
| ENSG00000125650 | 142.0729 | 203.1783 | -0.51831 | 0.03924 | 0.638476 | PSPN | 19 |
| ENSG00000144792 | 0 | 4.001661 | -4.48965 | 0.039244 | 1 | ZNF660 | 3 |
| ENSG00000146278 | 559.3163 | 722.0463 | -0.36811 | 0.03928 | 0.638476 | PNRC1 | 6 |
| ENSG00000271797 | 5.563969 | 0.347413 | 3.934942 | 0.0394 | 1 | AC008494.3 | 5 |
| ENSG00000138795 | 225.517 | 161.8525 | 0.478911 | 0.039582 | 0.641435 | LEF1 | 4 |
| ENSG00000172922 | 2694.106 | 2123.865 | 0.343056 | 0.039588 | 0.641435 | RNASEH2C | 11 |
| ENSG00000106571 | 445.6006 | 581.4314 | -0.38344 | 0.039608 | 0.641435 | GLI3 | 7 |
| ENSG00000198680 | 253.091 | 342.8286 | -0.43821 | 0.039737 | 0.641435 | TUSC1 | 9 |
| ENSG00000165752 | 868.9065 | 659.8025 | 0.396644 | 0.039808 | 0.641435 | STK32C | 10 |
| ENSG00000183617 | 945.3097 | 669.3902 | 0.498002 | 0.039871 | 0.641435 | MRPL54 | 19 |
| ENSG00000161021 | 1159.265 | 1456.758 | -0.32967 | 0.039881 | 0.641435 | MAML1 | 5 |
| ENSG00000143367 | 574.6818 | 441.1296 | 0.381022 | 0.039903 | 0.641435 | TUFT1 | 1 |
| ENSG00000284689 | 4.065315 | 0 | 4.450462 | 0.039938 | 1 | OR7E84P | 4 |
| ENSG00000279476 | 11.5112 | 3.030846 | 1.925413 | 0.039962 | 1 | AC092139.3 | 16 |
| ENSG00000147813 | 258.9772 | 376.5604 | -0.54065 | 0.040012 | 0.641435 | NAPRT | 8 |
| ENSG00000176171 | 1740.943 | 2325.05 | -0.41726 | 0.040038 | 0.641435 | BNIP3 | 10 |
| ENSG00000189143 | 478.823 | 640.2687 | -0.4196 | 0.040038 | 0.641435 | CLDN4 | 7 |
| ENSG00000254827 | 30.74334 | 13.83105 | 1.151042 | 0.040144 | 0.641435 | SLC22A18AS | 11 |
| ENSG00000095587 | 3.902069 | 13.2468 | -1.7661 | 0.040191 | 0.641435 | TLL2 | 10 |
| ENSG00000268350 | 6.825889 | 24.48626 | -1.84721 | 0.0403 | 0.641435 | FAM156A | X |
| ENSG00000168003 | 13267.94 | 10005.95 | 0.407054 | 0.040316 | 0.641435 | SLC3A2 | 11 |
| ENSG00000125691 | 20230.23 | 25756.28 | -0.34843 | 0.040329 | 0.641435 | RPL23 | 17 |
| ENSG00000174132 | 175.3563 | 240.951 | -0.45743 | 0.04033 | 0.641435 | FAM174A | 5 |
| ENSG00000173486 | 220.8326 | 323.5493 | -0.55214 | 0.040335 | 0.641435 | FKBP2 | 11 |
| ENSG00000186318 | 454.0481 | 591.5541 | -0.38199 | 0.040357 | 0.641435 | BACE1 | 11 |
| ENSG00000235508 | 16.33719 | 37.78425 | -1.20091 | 0.040386 | 0.641435 | RPS2P7 | 20 |
| ENSG00000171724 | 2.004399 | 9.477585 | -2.26373 | 0.040396 | 1 | VAT1L | 16 |
| ENSG00000147403 | 20193.81 | 25359 | -0.3286 | 0.040421 | 0.641435 | RPL10 | X |
| ENSG00000279382 | 8.29484 | 21.72816 | -1.38242 | 0.040485 | 0.641435 | AC018665.1 | 17 |
| ENSG00000241431 | 4.627446 | 15.75222 | -1.78042 | 0.040487 | 0.641435 | RPL37P6 | 8 |
| ENSG00000171148 | 8367.396 | 6398.352 | 0.387003 | 0.040501 | 0.641435 | TADA3 | 3 |
| ENSG00000213199 | 32.80728 | 57.69398 | -0.81554 | 0.040727 | 0.644333 | ASIC3 | 7 |
| ENSG00000151882 | 209.1088 | 280.2992 | -0.42284 | 0.040804 | 0.644812 | CCL28 | 5 |
| ENSG00000130024 | 1089.238 | 1398.332 | -0.36001 | 0.040857 | 0.644812 | PHF10 | 6 |
| ENSG00000171124 | 0.61659 | 7.275255 | -3.52728 | 0.040861 | 1 | FUT3 | 19 |
| ENSG00000164938 | 58.95481 | 100.6296 | -0.76866 | 0.040904 | 0.644812 | TP53INP1 | 8 |
| ENSG00000136940 | 350.4499 | 468.4184 | -0.41816 | 0.040931 | 0.644812 | PDCL | 9 |
| ENSG00000122877 | 6.079294 | 0.686997 | 3.162176 | 0.040986 | 1 | EGR2 | 10 |
| ENSG00000160799 | 232.4151 | 335.0881 | -0.52863 | 0.040986 | 0.644987 | CCDC12 | 3 |
| ENSG00000120910 | 162.7186 | 224.5475 | -0.46378 | 0.041101 | 0.645006 | PPP3CC | 8 |
| ENSG00000170946 | 240.4595 | 325.3681 | -0.43537 | 0.041105 | 0.645006 | DNAJC24 | 11 |
| ENSG00000212978 | 251.5688 | 181.9542 | 0.466942 | 0.041184 | 0.645006 | AC016747.1 | 2 |
| ENSG00000080824 | 11364.99 | 16329.09 | -0.52282 | 0.041214 | 0.645006 | HSP90AA1 | 14 |
| ENSG00000110074 | 1390.99 | 1095.156 | 0.344772 | 0.041298 | 0.645006 | FOXRED1 | 11 |
| ENSG00000116251 | 1462.408 | 1834.775 | -0.32716 | 0.041305 | 0.645006 | RPL22 | 1 |
| ENSG00000115009 | 12.61946 | 28.23753 | -1.16129 | 0.041336 | 0.645006 | CCL20 | 2 |
| ENSG00000233966 | 96.18218 | 151.9463 | -0.66148 | 0.041352 | 0.645006 | UBE2SP1 | 17 |
| ENSG00000107815 | 828.7996 | 614.9161 | 0.430171 | 0.041379 | 0.645006 | TWNK | 10 |
| ENSG00000066135 | 407.2319 | 291.6371 | 0.480876 | 0.041574 | 0.646699 | KDM4A | 1 |
| ENSG00000077616 | 19.12335 | 37.17403 | -0.96063 | 0.041606 | 0.646699 | NAALAD2 | 11 |
| ENSG00000089327 | 2017.503 | 2669.407 | -0.40422 | 0.041808 | 0.646699 | FXYD5 | 19 |
| ENSG00000277383 | 34.47053 | 16.83306 | 1.030654 | 0.041814 | 0.646699 | AC010331.1 | 19 |
| ENSG00000135519 | 25.26765 | 11.1012 | 1.183566 | 0.04183 | 0.646699 | KCNH3 | 12 |
| ENSG00000272853 | 52.72332 | 27.46153 | 0.939476 | 0.04185 | 0.646699 | AC069544.1 | 10 |
| ENSG00000088726 | 53.34539 | 28.38097 | 0.91367 | 0.041854 | 0.646699 | TMEM40 | 3 |
| ENSG00000168394 | 1556.66 | 1204.409 | 0.369784 | 0.041869 | 0.646699 | TAP1 | 6 |
| ENSG00000047648 | 36.31046 | 17.15355 | 1.081047 | 0.04188 | 0.646699 | ARHGAP6 | X |
| ENSG00000272473 | 103.0625 | 62.97797 | 0.709308 | 0.042123 | 0.648382 | AC006273.1 | 19 |
| ENSG00000235194 | 133.2158 | 187.6613 | -0.49427 | 0.042133 | 0.648382 | PPP1R3E | 14 |
| ENSG00000238062 | 14.67831 | 4.02611 | 1.864636 | 0.042156 | 0.648382 | SPATA3-AS1 | 2 |
| ENSG00000170684 | 55.37389 | 89.15832 | -0.68812 | 0.042204 | 0.648382 | ZNF296 | 19 |
| ENSG00000151687 | 87.56716 | 54.73804 | 0.679188 | 0.042221 | 0.648382 | ANKAR | 2 |
| ENSG00000108448 | 419.0788 | 550.4624 | -0.39413 | 0.042273 | 0.648382 | TRIM16L | 17 |
| ENSG00000085644 | 437.3255 | 300.1097 | 0.542547 | 0.042295 | 0.648382 | ZNF213 | 16 |
| ENSG00000142227 | 3910.177 | 5199.262 | -0.41119 | 0.042402 | 0.648769 | EMP3 | 19 |
| ENSG00000270673 | 46.60861 | 25.40589 | 0.875849 | 0.042436 | 0.648769 | YTHDF3-AS1 | 8 |
| ENSG00000129757 | 115.5508 | 172.4571 | -0.579 | 0.042452 | 0.648769 | CDKN1C | 11 |
| ENSG00000141295 | 846.9885 | 661.3185 | 0.356623 | 0.042499 | 0.648826 | SCRN2 | 17 |
| ENSG00000068001 | 600.505 | 830.7049 | -0.46843 | 0.042565 | 0.649156 | HYAL2 | 3 |
| ENSG00000259538 | 3.690923 | 0 | 4.300275 | 0.042704 | 1 | UBE2Q2P11 | 15 |
| ENSG00000117877 | 279.3537 | 209.4264 | 0.415524 | 0.042722 | 0.650849 | CD3EAP | 19 |
| ENSG00000073910 | 68.58776 | 42.25653 | 0.699603 | 0.042764 | 0.650849 | FRY | 13 |
| ENSG00000163739 | 875.2515 | 1175.3 | -0.42516 | 0.042911 | 0.65133 | CXCL1 | 4 |
| ENSG00000090432 | 1407.805 | 1119.29 | 0.330613 | 0.042924 | 0.65133 | MUL1 | 1 |
| ENSG00000105229 | 495.3523 | 657.6463 | -0.4097 | 0.042927 | 0.65133 | PIAS4 | 19 |
| ENSG00000235527 | 4.829012 | 0.339584 | 3.733157 | 0.042974 | 1 | HIPK1-AS1 | 1 |
| ENSG00000003096 | 62.12108 | 100.6873 | -0.69609 | 0.043074 | 0.652898 | KLHL13 | X |
| ENSG00000185043 | 1523.835 | 2014.404 | -0.40279 | 0.043282 | 0.655234 | CIB1 | 15 |
| ENSG00000232931 | 12.39436 | 28.66767 | -1.2036 | 0.043317 | 0.655234 | LINC00342 | 2 |
| ENSG00000197982 | 1224.977 | 955.4294 | 0.358291 | 0.043536 | 0.657874 | C1orf122 | 1 |
| ENSG00000253978 | 3.745382 | 0 | 4.332863 | 0.043701 | 1 | CTB-178M22.2 | 5 |
| ENSG00000122705 | 6758.158 | 8641.935 | -0.35475 | 0.043732 | 0.660173 | CLTA | 9 |
| ENSG00000276317 | 62.51585 | 35.75114 | 0.803846 | 0.043912 | 0.6617 | AL357033.3 | 20 |
| ENSG00000253200 | 3.654661 | 12.83139 | -1.82839 | 0.043923 | 0.6617 | AC037459.3 | 8 |
| ENSG00000237440 | 29.1314 | 51.81969 | -0.83133 | 0.04415 | 0.662907 | ZNF737 | 19 |
| ENSG00000031691 | 359.8387 | 480.1306 | -0.41512 | 0.044336 | 0.662907 | CENPQ | 6 |
| ENSG00000224725 | 10.97877 | 2.651154 | 2.038011 | 0.044369 | 1 | CEP57L1P1 | 10 |
| ENSG00000226887 | 8.336566 | 1.701354 | 2.299438 | 0.044369 | 1 | ERVMER34-1 | 4 |
| ENSG00000049323 | 102.9579 | 65.58024 | 0.65353 | 0.044372 | 0.662907 | LTBP1 | 2 |
| ENSG00000105397 | 2160.031 | 1707.074 | 0.33935 | 0.044373 | 0.662907 | TYK2 | 19 |
| ENSG00000196937 | 560.2585 | 732.4307 | -0.38598 | 0.044405 | 0.662907 | FAM3C | 7 |
| ENSG00000280202 | 134.3829 | 88.3352 | 0.606247 | 0.044413 | 0.662907 | AC005831.1 | 12 |
| ENSG00000116521 | 2242.417 | 2793.337 | -0.31706 | 0.044431 | 0.662907 | SCAMP3 | 1 |
| ENSG00000173705 | 559.3079 | 435.6352 | 0.360784 | 0.044442 | 0.662907 | SUSD5 | 3 |
| ENSG00000141642 | 119.6363 | 82.1541 | 0.542701 | 0.044495 | 0.662907 | ELAC1 | 18 |
| ENSG00000189362 | 340.2054 | 240.3799 | 0.500995 | 0.044503 | 0.662907 | NEMP2 | 2 |
| ENSG00000181827 | 278.5027 | 373.0765 | -0.42073 | 0.044653 | 0.662907 | RFX7 | 15 |
| ENSG00000180385 | 46.21821 | 74.67982 | -0.69257 | 0.044719 | 0.662907 | EMC3-AS1 | 3 |
| ENSG00000196943 | 212.2753 | 298.3083 | -0.49133 | 0.044804 | 0.662907 | NOP9 | 14 |
| ENSG00000139266 | 441.87 | 326.3563 | 0.436636 | 0.044818 | 0.662907 | 9-Mar | 12 |
| ENSG00000105855 | 13.02805 | 3.665511 | 1.825129 | 0.044858 | 0.662907 | ITGB8 | 7 |
| ENSG00000266921 | 0.689115 | 6.121853 | -3.20491 | 0.04488 | 1 | AC006213.1 | 19 |
| ENSG00000245556 | 191.8125 | 138.5652 | 0.468484 | 0.044919 | 0.662907 | SCAMP1-AS1 | 5 |
| ENSG00000145331 | 81.11527 | 128.9214 | -0.66594 | 0.045022 | 0.662907 | TRMT10A | 4 |
| ENSG00000166224 | 2465.749 | 1879.421 | 0.391659 | 0.045094 | 0.662907 | SGPL1 | 10 |
| ENSG00000234498 | 328.9903 | 227.819 | 0.529454 | 0.045124 | 0.662907 | RPL13AP20 | 12 |
| ENSG00000136715 | 624.7176 | 834.3402 | -0.41723 | 0.045157 | 0.662907 | SAP130 | 2 |
| ENSG00000161010 | 285.0474 | 373.8701 | -0.39126 | 0.04519 | 0.662907 | MRNIP | 5 |
| ENSG00000227097 | 2084.503 | 2686.621 | -0.36616 | 0.045201 | 0.662907 | RPS28P7 | 11 |
| ENSG00000237493 | 73.83835 | 45.77077 | 0.692443 | 0.045226 | 0.662907 | AC034102.1 | 12 |
| ENSG00000169715 | 7563.748 | 5939.558 | 0.34877 | 0.045229 | 0.662907 | MT1E | 16 |
| ENSG00000226856 | 14.91299 | 5.079613 | 1.553917 | 0.045292 | 0.662907 | THORLNC | 2 |
| ENSG00000136371 | 40.78521 | 20.37614 | 1.00084 | 0.045313 | 0.662907 | MTHFS | 15 |
| ENSG00000284879 | 0.944045 | 27.54709 | -4.85892 | 0.04532 | 0.662907 | AC133644.3 | 2 |
| ENSG00000143850 | 557.8361 | 725.3629 | -0.37911 | 0.045323 | 0.662907 | PLEKHA6 | 1 |
| ENSG00000263535 | 55.00593 | 30.02848 | 0.869913 | 0.045328 | 0.662907 | AK4P1 | 17 |
| ENSG00000167987 | 1263.742 | 996.0027 | 0.343206 | 0.045417 | 0.662907 | VPS37C | 11 |
| ENSG00000261338 | 8.357784 | 21.86071 | -1.38274 | 0.04546 | 0.662907 | AC021016.2 | 2 |
| ENSG00000171861 | 1423.504 | 1141.484 | 0.318421 | 0.045536 | 0.662907 | MRM3 | 17 |
| ENSG00000175556 | 234.9226 | 165.1611 | 0.50941 | 0.045537 | 0.662907 | LONRF3 | X |
| ENSG00000196199 | 369.9759 | 499.3191 | -0.43153 | 0.045541 | 0.662907 | MPHOSPH8 | 13 |
| ENSG00000136052 | 327.4252 | 241.0107 | 0.44297 | 0.045568 | 0.662907 | SLC41A2 | 12 |
| ENSG00000176438 | 1144.506 | 836.3104 | 0.452374 | 0.045709 | 0.664249 | SYNE3 | 14 |
| ENSG00000065518 | 2143.49 | 2691.392 | -0.32846 | 0.0458 | 0.664249 | NDUFB4 | 3 |
| ENSG00000113068 | 2369.373 | 1909.776 | 0.310969 | 0.045814 | 0.664249 | PFDN1 | 5 |
| ENSG00000197256 | 2021.585 | 2511.343 | -0.31304 | 0.04584 | 0.664249 | KANK2 | 19 |
| ENSG00000100031 | 30.30395 | 54.38142 | -0.84693 | 0.045993 | 0.664851 | GGT1 | 22 |
| ENSG00000145945 | 265.3107 | 190.3643 | 0.477777 | 0.045996 | 0.664851 | FAM50B | 6 |
| ENSG00000180801 | 641.685 | 498.9274 | 0.36309 | 0.046016 | 0.664851 | ARSJ | 4 |
| ENSG00000213638 | 103.1029 | 151.7035 | -0.56007 | 0.046063 | 0.66488 | ADAT3 | 19 |
| ENSG00000230870 | 4.634968 | 0.347413 | 3.668317 | 0.046223 | 1 | FBXW11P1 | 21 |
| ENSG00000068912 | 611.9584 | 801.1325 | -0.3879 | 0.04624 | 0.666798 | ERLEC1 | 2 |
| ENSG00000170345 | 21.72589 | 51.49417 | -1.2442 | 0.046289 | 0.666848 | FOS | 14 |
| ENSG00000100075 | 1725.2 | 2426.744 | -0.49237 | 0.046467 | 0.66816 | SLC25A1 | 22 |
| ENSG00000168280 | 32.9151 | 14.1716 | 1.215243 | 0.046514 | 0.66816 | KIF5C | 2 |
| ENSG00000091164 | 1091.565 | 1384.71 | -0.3431 | 0.046555 | 0.66816 | TXNL1 | 18 |
| ENSG00000134802 | 2765.283 | 2185.361 | 0.339553 | 0.046594 | 0.66816 | SLC43A3 | 11 |
| ENSG00000163040 | 43.23883 | 76.06545 | -0.81784 | 0.046605 | 0.66816 | CCDC74A | 2 |
| ENSG00000173660 | 1872.268 | 2405.775 | -0.3618 | 0.046741 | 0.668222 | UQCRH | 1 |
| ENSG00000028277 | 564.7445 | 412.5561 | 0.45237 | 0.046824 | 0.668222 | POU2F2 | 19 |
| ENSG00000187098 | 531.4025 | 381.3429 | 0.478911 | 0.046869 | 0.668222 | MITF | 3 |
| ENSG00000140830 | 590.4401 | 452.5823 | 0.383277 | 0.046874 | 0.668222 | TXNL4B | 16 |
| ENSG00000085871 | 444.2269 | 606.8136 | -0.44989 | 0.046886 | 0.668222 | MGST2 | 4 |
| ENSG00000254999 | 3202.388 | 4077.933 | -0.34879 | 0.046912 | 0.668222 | BRK1 | 3 |
| ENSG00000101745 | 245.6435 | 344.2187 | -0.48515 | 0.04696 | 0.668222 | ANKRD12 | 18 |
| ENSG00000163638 | 11.5112 | 25.7621 | -1.15701 | 0.04697 | 0.668222 | ADAMTS9 | 3 |
| ENSG00000226329 | 2.90466 | 12.71048 | -2.12641 | 0.047274 | 1 | AC005682.1 | 7 |
| ENSG00000132570 | 179.2701 | 126.3219 | 0.506085 | 0.047319 | 0.672537 | PCBD2 | 5 |
| ENSG00000230555 | 47.48488 | 24.48457 | 0.952923 | 0.047461 | 0.67379 | AL450326.1 | 10 |
| ENSG00000047230 | 673.1913 | 514.8374 | 0.386851 | 0.047526 | 0.67379 | CTPS2 | X |
| ENSG00000066322 | 3882.493 | 3129.337 | 0.311033 | 0.047543 | 0.67379 | ELOVL1 | 1 |
| ENSG00000145860 | 1144.066 | 1483.449 | -0.3745 | 0.047666 | 0.674031 | RNF145 | 5 |
| ENSG00000100632 | 2022.421 | 2534.809 | -0.32582 | 0.047688 | 0.674031 | ERH | 14 |
| ENSG00000261159 | 31.96463 | 14.75641 | 1.114248 | 0.047732 | 0.674031 | AC112484.3 | 3 |
| ENSG00000116001 | 354.5507 | 483.2689 | -0.44561 | 0.047806 | 0.674031 | TIA1 | 2 |
| ENSG00000043355 | 198.1838 | 266.9768 | -0.4302 | 0.047902 | 0.674031 | ZIC2 | 13 |
| ENSG00000111653 | 270.2942 | 356.927 | -0.40207 | 0.047916 | 0.674031 | ING4 | 12 |
| ENSG00000103653 | 2205.037 | 1747.073 | 0.335644 | 0.047918 | 0.674031 | CSK | 15 |
| ENSG00000170144 | 2223.935 | 3072.809 | -0.46631 | 0.047924 | 0.674031 | HNRNPA3 | 2 |
| ENSG00000105486 | 519.3542 | 756.3568 | -0.54234 | 0.048128 | 0.675879 | LIG1 | 19 |
| ENSG00000212747 | 607.0849 | 767.9089 | -0.33933 | 0.048149 | 0.675879 | RTL8B | X |
| ENSG00000087842 | 118.816 | 178.2328 | -0.58568 | 0.048253 | 0.675879 | PIR | X |
| ENSG00000169814 | 559.4852 | 438.2391 | 0.352601 | 0.048294 | 0.675879 | BTD | 3 |
| ENSG00000108175 | 334.4325 | 462.6145 | -0.46789 | 0.048367 | 0.675879 | ZMIZ1 | 10 |
| ENSG00000085840 | 172.3999 | 262.954 | -0.60805 | 0.048412 | 0.675879 | ORC1 | 1 |
| ENSG00000204920 | 87.79431 | 56.28373 | 0.643366 | 0.048469 | 0.675879 | ZNF155 | 19 |
| ENSG00000224597 | 597.3976 | 459.5893 | 0.378339 | 0.048493 | 0.675879 | SVIL-AS1 | 10 |
| ENSG00000060566 | 5.535229 | 16.0962 | -1.54601 | 0.04851 | 0.675879 | CREB3L3 | 19 |
| ENSG00000185085 | 958.5531 | 752.4071 | 0.348992 | 0.048549 | 0.675879 | INTS5 | 11 |
| ENSG00000151689 | 1009.242 | 792.9458 | 0.347608 | 0.048557 | 0.675879 | INPP1 | 2 |
| ENSG00000105427 | 181.8688 | 130.5394 | 0.477827 | 0.048704 | 0.67712 | CNFN | 19 |
| ENSG00000100092 | 21.4689 | 41.41291 | -0.94951 | 0.048738 | 0.67712 | SH3BP1 | 22 |
| ENSG00000162642 | 440.5404 | 566.9406 | -0.3634 | 0.048885 | 0.678531 | C1orf52 | 1 |
| ENSG00000143845 | 272.3793 | 367.6508 | -0.43386 | 0.049065 | 0.68039 | ETNK2 | 1 |
| ENSG00000104635 | 3584.953 | 2677.913 | 0.420825 | 0.049197 | 0.681121 | SLC39A14 | 8 |
| ENSG00000095321 | 1063.638 | 1408.583 | -0.40565 | 0.049209 | 0.681121 | CRAT | 9 |
| ENSG00000166676 | 0.662432 | 6.103721 | -3.22423 | 0.049216 | 1 | TVP23A | 16 |
| ENSG00000103202 | 1004.829 | 1336.164 | -0.41152 | 0.049266 | 0.681274 | NME4 | 16 |
| ENSG00000142534 | 18634.92 | 23604.39 | -0.34107 | 0.049362 | 0.681966 | RPS11 | 19 |
| ENSG00000212829 | 14.6218 | 30.12243 | -1.04518 | 0.049548 | 0.682743 | RPS26P3 | 9 |
| ENSG00000198440 | 83.88749 | 53.11841 | 0.660669 | 0.049593 | 0.682743 | ZNF583 | 19 |
| ENSG00000204054 | 594.2521 | 419.3722 | 0.502247 | 0.049622 | 0.682743 | LINC00963 | 9 |
| ENSG00000118197 | 194.9211 | 269.1383 | -0.46453 | 0.04963 | 0.682743 | DDX59 | 1 |
| ENSG00000132906 | 374.3891 | 282.1346 | 0.407525 | 0.04968 | 0.682743 | CASP9 | 1 |
| ENSG00000144134 | 40.66588 | 65.98799 | -0.69784 | 0.049708 | 0.682743 | RABL2A | 2 |
| ENSG00000100522 | 1228.98 | 965.7577 | 0.347938 | 0.049741 | 0.682743 | GNPNAT1 | 14 |
| ENSG00000160325 | 236.3113 | 313.4584 | -0.40832 | 0.049863 | 0.683703 | CACFD1 | 9 |
| ENSG00000110619 | 4470.402 | 3118.709 | 0.519358 | 0.049903 | 0.683703 | CARS | 11 |
| ENSG00000284727 | 0 | 3.330322 | -4.22535 | 0.049905 | 1 | AC116562.4 | 4 |

| **Table S6. Differentially translated genes between FIBCD1-overexpressing and control cells** | | | | | | | |
| --- | --- | --- | --- | --- | --- | --- | --- |
| **gene_id** | **FIBCD1** | **EV** | **log2FoldChange** | **pvalue** | **padj** | **gene_name** | **gene_chr** |
| ENSG00000130720 | 306.6087 | 25.95636 | 2.850198 | 3.68E-31 | 1.26E-27 | FIBCD1 | 9 |
| ENSG00000207725 | 3063.146 | 7788.156 | -1.2495 | 5.55E-15 | 9.53E-12 | MIR222 | X |
| ENSG00000155657 | 251.1275 | 997.1419 | -1.69403 | 1.38E-14 | 1.59E-11 | TTN | 2 |
| ENSG00000207726 | 119.373 | 313.1201 | -1.20066 | 1.45E-08 | 1.25E-05 | MIR455 | 9 |
| ENSG00000171723 | 69.81755 | 13.85331 | 1.56255 | 5.21E-08 | 1 | GPHN | 14 |
| ENSG00000140465 | 58.24745 | 159.1032 | -1.17035 | 1.33E-06 | 0.000891 | CYP1A1 | 15 |
| ENSG00000031698 | 1360.773 | 782.8351 | 0.743118 | 1.67E-06 | 0.000891 | SARS | 1 |
| ENSG00000198987 | 29.87367 | 107.4236 | -1.32803 | 1.78E-06 | 1 | MIR16-2 | 3 |
| ENSG00000106105 | 951.7541 | 539.0221 | 0.762581 | 1.82E-06 | 0.000891 | GARS | 7 |
| ENSG00000221540 | 63.81518 | 207.841 | -1.24192 | 6.40E-06 | 0.002748 | MIR1180 | 17 |
| ENSG00000196305 | 1998.475 | 1214.771 | 0.67183 | 1.10E-05 | 0.004182 | IARS | 9 |
| ENSG00000148344 | 198.5675 | 399.9892 | -0.88792 | 1.27E-05 | 0.00436 | PTGES | 9 |
| ENSG00000248473 | 118.2956 | 296.7682 | -1.06065 | 1.81E-05 | 0.005658 | LINC01962 | 5 |
| ENSG00000249020 | 104.4108 | 244.6165 | -0.99428 | 3.99E-05 | 0.010216 | SNORA58 | 3 |
| ENSG00000207691 | 157.0758 | 394.9093 | -1.04177 | 4.27E-05 | 0.010216 | MIR183 | 7 |
| ENSG00000245937 | 651.6328 | 1206.581 | -0.79058 | 4.55E-05 | 0.010216 | LINC01184 | 5 |
| ENSG00000148303 | 1109.103 | 688.8946 | 0.639319 | 4.82E-05 | 0.010216 | RPL7A | 9 |
| ENSG00000100889 | 232.0141 | 113.101 | 0.891332 | 4.87E-05 | 0.010216 | PCK2 | 14 |
| ENSG00000207971 | 28.03317 | 92.90049 | -1.16808 | 5.04E-05 | 1 | MIR125B1 | 11 |
| ENSG00000134440 | 544.9835 | 308.9427 | 0.741231 | 5.06E-05 | 0.010216 | NARS | 18 |
| ENSG00000069869 | 216.0546 | 114.6188 | 0.807294 | 5.95E-05 | 0.011353 | NEDD4 | 15 |
| ENSG00000115053 | 705.2511 | 444.1894 | 0.623572 | 6.51E-05 | 0.011762 | NCL | 2 |
| ENSG00000152223 | 89.41973 | 35.76037 | 1.026593 | 6.64E-05 | 1 | EPG5 | 18 |
| ENSG00000200087 | 74.33094 | 181.1895 | -1.00477 | 7.88E-05 | 0.013533 | SNORA73B | 1 |
| ENSG00000226674 | 36.4384 | 102.5417 | -1.07885 | 9.55E-05 | 1 | TEX41 | 2 |
| ENSG00000148677 | 630.2355 | 370.7905 | 0.692715 | 0.00011 | 0.017348 | ANKRD1 | 10 |
| ENSG00000112592 | 142.3689 | 66.45527 | 0.902516 | 0.000111 | 0.017348 | TBP | 6 |
| ENSG00000198804 | 729.9256 | 1135.841 | -0.59531 | 0.000118 | 0.017615 | MT-CO1 | MT |
| ENSG00000267200 | 26.52173 | 101.9581 | -1.15465 | 0.00014 | 1 | MIR132 | 17 |
| ENSG00000135046 | 1161.045 | 725.4388 | 0.626546 | 0.000166 | 0.023823 | ANXA1 | 9 |
| ENSG00000201595 | 72.83852 | 191.0303 | -1.01202 | 0.000224 | 0.030494 | RNA5SP132 | 3 |
| ENSG00000163975 | 225.6636 | 372.3644 | -0.65578 | 0.000231 | 0.030494 | MELTF | 3 |
| ENSG00000100234 | 8.565199 | 34.89429 | -1.12657 | 0.000245 | 1 | TIMP3 | 22 |
| ENSG00000207994 | 13638.19 | 42516.47 | -1.07698 | 0.000247 | 0.031356 | MIR100 | 11 |
| ENSG00000156475 | 10.29471 | 575.9334 | -0.99285 | 0.000309 | 0.037365 | PPP2R2B | 5 |
| ENSG00000197102 | 964.1927 | 597.946 | 0.630248 | 0.000316 | 0.037365 | DYNC1H1 | 14 |
| ENSG00000130202 | 173.4605 | 291.4232 | -0.67386 | 0.000346 | 0.038833 | NECTIN2 | 19 |
| ENSG00000151651 | 163.1826 | 281.626 | -0.70032 | 0.000351 | 0.038833 | ADAM8 | 10 |
| ENSG00000230530 | 407.9394 | 771.203 | -0.7808 | 0.000406 | 0.042684 | LIMD1-AS1 | 3 |
| ENSG00000133706 | 254.5386 | 141.5737 | 0.736045 | 0.00041 | 0.042684 | LARS | 5 |
| ENSG00000251733 | 128.0103 | 258.7569 | -0.83417 | 0.000503 | 0.050848 | SCARNA8 | 9 |
| ENSG00000104419 | 399.9424 | 599.1509 | -0.54256 | 0.000541 | 0.05183 | NDRG1 | 8 |
| ENSG00000167658 | 999.1634 | 666.6821 | 0.544039 | 0.000543 | 0.05183 | EEF2 | 19 |
| ENSG00000171345 | 37.03642 | 10.86738 | 1.037462 | 0.000648 | 1 | KRT19 | 17 |
| ENSG00000166340 | 185.0729 | 310.5276 | -0.66247 | 0.000739 | 0.065498 | TPP1 | 11 |
| ENSG00000142089 | 227.8519 | 426.7457 | -0.76234 | 0.000739 | 0.065498 | IFITM3 | 11 |
| ENSG00000125977 | 886.3521 | 597.8238 | 0.529469 | 0.000744 | 0.065498 | EIF2S2 | 20 |
| ENSG00000176845 | 27.46786 | 79.94229 | -0.99249 | 0.000748 | 1 | METRNL | 17 |
| ENSG00000123689 | 339.0444 | 185.3416 | 0.738444 | 0.000912 | 0.078276 | G0S2 | 1 |
| ENSG00000221585 | 154.2838 | 280.8142 | -0.72898 | 0.001028 | 0.086143 | MIR1226 | 3 |
| ENSG00000012171 | 162.9249 | 283.2842 | -0.69223 | 0.001087 | 0.087721 | SEMA3B | 3 |
| ENSG00000182718 | 2977.223 | 2115.044 | 0.46501 | 0.001119 | 0.087721 | ANXA2 | 15 |
| ENSG00000100239 | 75.88971 | 141.0442 | -0.75263 | 0.001124 | 0.087721 | PPP6R2 | 22 |
| ENSG00000107937 | 154.5916 | 86.27115 | 0.715224 | 0.0013 | 0.09377 | GTPBP4 | 10 |
| ENSG00000141736 | 96.25621 | 169.9294 | -0.69877 | 0.001303 | 0.09377 | ERBB2 | 17 |
| ENSG00000189046 | 72.0917 | 33.0118 | 0.858005 | 0.001313 | 1 | ALKBH2 | 12 |
| ENSG00000113013 | 440.1789 | 263.4664 | 0.653244 | 0.001365 | 0.09377 | HSPA9 | 5 |
| ENSG00000114757 | 224.2386 | 840.8852 | -0.99043 | 0.001373 | 0.09377 | PEX5L | 3 |
| ENSG00000138326 | 1118.89 | 707.343 | 0.594582 | 0.001377 | 0.09377 | RPS24 | 10 |
| ENSG00000204628 | 2921.591 | 2084.712 | 0.459374 | 0.001381 | 0.09377 | RACK1 | 5 |
| ENSG00000162337 | 217.5379 | 346.0516 | -0.60123 | 0.001393 | 0.09377 | LRP5 | 11 |
| ENSG00000182944 | 328.2593 | 184.0572 | 0.707984 | 0.00145 | 0.09499 | EWSR1 | 22 |
| ENSG00000004399 | 18.87002 | 51.09765 | -0.93424 | 0.001471 | 1 | PLXND1 | 3 |
| ENSG00000101255 | 214.4827 | 119.1734 | 0.71511 | 0.00149 | 0.09499 | TRIB3 | 20 |
| ENSG00000147274 | 111.0582 | 55.46611 | 0.80044 | 0.001494 | 0.09499 | RBMX | X |
| ENSG00000135269 | 123.5047 | 62.44433 | 0.787224 | 0.001582 | 0.098772 | TES | 7 |
| ENSG00000206754 | 38.92218 | 81.70907 | -0.8259 | 0.001606 | 1 | SNORD101 | 6 |
| ENSG00000071967 | 108.4735 | 194.5385 | -0.7139 | 0.001612 | 0.09888 | CYBRD1 | 2 |
| ENSG00000238835 | 8.206874 | 31.56586 | -0.97639 | 0.001616 | 1 | SCARNA18 | 5 |
| ENSG00000108679 | 1529.183 | 2140.471 | -0.45654 | 0.001651 | 0.09945 | LGALS3BP | 17 |
| ENSG00000205542 | 428.6109 | 760.9203 | -0.70165 | 0.00173 | 0.102406 | TMSB4X | X |
| ENSG00000090861 | 364.59 | 231.8699 | 0.587161 | 0.001779 | 0.103567 | AARS | 16 |
| ENSG00000135069 | 475.2745 | 312.4668 | 0.551913 | 0.001887 | 0.108009 | PSAT1 | 9 |
| ENSG00000181523 | 66.22422 | 119.4386 | -0.71446 | 0.001935 | 0.108936 | SGSH | 17 |
| ENSG00000162576 | 148.0031 | 237.0795 | -0.60214 | 0.00209 | 0.115769 | MXRA8 | 1 |
| ENSG00000107984 | 811.9352 | 562.0337 | 0.493298 | 0.002162 | 0.117845 | DKK1 | 10 |
| ENSG00000223224 | 12.30498 | 39.48168 | -0.93998 | 0.002191 | 1 | SNORD71 | 16 |
| ENSG00000113407 | 613.3829 | 424.0992 | 0.494714 | 0.002235 | 0.119934 | TARS | 5 |
| ENSG00000187109 | 479.7327 | 322.4811 | 0.522803 | 0.002311 | 0.12207 | NAP1L1 | 12 |
| ENSG00000228253 | 20.9227 | 66.3063 | -0.93562 | 0.002312 | 1 | MT-ATP8 | MT |
| ENSG00000251898 | 73.95322 | 150.3339 | -0.78731 | 0.002402 | 0.12222 | SCARNA11 | 12 |
| ENSG00000204525 | 153.5304 | 253.8724 | -0.63325 | 0.002405 | 0.12222 | HLA-C | 6 |
| ENSG00000119655 | 337.5092 | 511.478 | -0.54691 | 0.00242 | 0.12222 | NPC2 | 14 |
| ENSG00000233822 | 19.46082 | 49.11464 | -0.88535 | 0.002576 | 1 | HIST1H2BN | 6 |
| ENSG00000104231 | 171.4447 | 101.8142 | 0.647092 | 0.002604 | 0.126201 | ZFAND1 | 8 |
| ENSG00000125944 | 203.8939 | 123.818 | 0.624464 | 0.002605 | 0.126201 | HNRNPR | 1 |
| ENSG00000252712 | 157.2771 | 291.023 | -0.72136 | 0.002609 | 0.126201 | SCARNA14 | 15 |
| ENSG00000265961 | 305.8316 | 493.1861 | -0.60471 | 0.00269 | 0.127389 | RF00019 | 1 |
| ENSG00000185201 | 128.2976 | 235.5707 | -0.72012 | 0.002708 | 0.127389 | IFITM2 | 11 |
| ENSG00000251221 | 4.886365 | 28.92509 | -0.89595 | 0.00286 | 1 | LINC01337 | 5 |
| ENSG00000144867 | 228.8547 | 126.0158 | 0.710763 | 0.002944 | 0.136176 | SRPRB | 3 |
| ENSG00000151012 | 107.0038 | 56.76374 | 0.734185 | 0.002979 | 0.136176 | SLC7A11 | 4 |
| ENSG00000102804 | 443.0717 | 651.0484 | -0.51018 | 0.003014 | 0.136176 | TSC22D1 | 13 |
| ENSG00000135842 | 253.3733 | 156.7836 | 0.607357 | 0.00308 | 0.137379 | FAM129A | 1 |
| ENSG00000256222 | 37.10112 | 86.96013 | -0.84719 | 0.003087 | 1 | MTRNR2L3 | 20 |
| ENSG00000175711 | 40.47587 | 14.56249 | 0.892108 | 0.003135 | 1 | B3GNTL1 | 17 |
| ENSG00000235408 | 559.9107 | 857.2447 | -0.55222 | 0.00321 | 0.138028 | SNORA71B | 20 |
| ENSG00000117394 | 271.5334 | 404.9511 | -0.52591 | 0.003249 | 0.138028 | SLC2A1 | 1 |
| ENSG00000135486 | 278.4949 | 177.5489 | 0.574495 | 0.003256 | 0.138028 | HNRNPA1 | 12 |
| ENSG00000180287 | 95.12889 | 195.5463 | -0.7817 | 0.003291 | 0.138028 | PLD5 | 1 |
| ENSG00000136068 | 539.7233 | 337.6539 | 0.597072 | 0.003331 | 0.138028 | FLNB | 3 |
| ENSG00000146733 | 141.473 | 81.37106 | 0.667285 | 0.003336 | 0.138028 | PSPH | 7 |
| ENSG00000196611 | 2886.528 | 3970.571 | -0.43228 | 0.003377 | 0.138052 | MMP1 | 11 |
| ENSG00000284485 | 31.19601 | 6.030102 | 0.885427 | 0.003494 | 1 | MIR205 | 1 |
| ENSG00000136999 | 64.33076 | 114.4158 | -0.68662 | 0.003537 | 0.140175 | CCN3 | 8 |
| ENSG00000212402 | 44.25678 | 87.32592 | -0.75302 | 0.003579 | 1 | SNORA74B | 5 |
| ENSG00000125356 | 601.1698 | 422.9572 | 0.471458 | 0.003587 | 0.140175 | NDUFA1 | X |
| ENSG00000026025 | 1490.047 | 1004.852 | 0.517693 | 0.00362 | 0.140175 | VIM | 10 |
| ENSG00000109606 | 202.0608 | 121.1258 | 0.633747 | 0.003648 | 0.140175 | DHX15 | 4 |
| ENSG00000198805 | 235.0424 | 152.9645 | 0.557623 | 0.003648 | 0.140175 | PNP | 14 |
| ENSG00000166197 | 147.2462 | 76.17079 | 0.738298 | 0.003677 | 0.140175 | NOLC1 | 10 |
| ENSG00000065183 | 187.7667 | 107.7665 | 0.668956 | 0.00372 | 0.140175 | WDR3 | 1 |
| ENSG00000104979 | 285.5699 | 175.0317 | 0.60797 | 0.003755 | 0.140175 | C19orf53 | 19 |
| ENSG00000205213 | 147.7658 | 230.5157 | -0.57096 | 0.00386 | 0.14147 | LGR4 | 11 |
| ENSG00000159210 | 113.0747 | 59.54155 | 0.729428 | 0.003872 | 0.14147 | SNF8 | 17 |
| ENSG00000172927 | 54.04049 | 23.9257 | 0.816145 | 0.003998 | 1 | MYEOV | 11 |
| ENSG00000207571 | 15.30121 | 2.195617 | 0.84766 | 0.004024 | 1 | MIR615 | 12 |
| ENSG00000166508 | 348.4099 | 227.0135 | 0.554604 | 0.004065 | 0.145585 | MCM7 | 7 |
| ENSG00000150687 | 249.8623 | 382.3082 | -0.54767 | 0.004112 | 0.145585 | PRSS23 | 11 |
| ENSG00000114867 | 299.4116 | 196.1717 | 0.546624 | 0.004112 | 0.145585 | EIF4G1 | 3 |
| ENSG00000164934 | 161.9849 | 100.1369 | 0.599588 | 0.004261 | 0.149303 | DCAF13 | 8 |
| ENSG00000122406 | 917.1105 | 665.1773 | 0.432119 | 0.004406 | 0.152825 | RPL5 | 1 |
| ENSG00000185483 | 26.24313 | 55.69144 | -0.78663 | 0.00446 | 1 | ROR1 | 1 |
| ENSG00000201470 | 253.4598 | 158.9893 | 0.588356 | 0.004463 | 0.153243 | RNY4P7 | 2 |
| ENSG00000037897 | 41.35156 | 16.72139 | 0.837999 | 0.004687 | 1 | METTL1 | 12 |
| ENSG00000144381 | 420.8881 | 271.5701 | 0.561308 | 0.004695 | 0.159504 | HSPD1 | 2 |
| ENSG00000152291 | 317.9473 | 451.9466 | -0.46724 | 0.004738 | 0.159504 | TGOLN2 | 2 |
| ENSG00000212443 | 39.13843 | 84.50616 | -0.78704 | 0.004954 | 1 | SNORA53 | 12 |
| ENSG00000212464 | 296.6144 | 504.4566 | -0.64075 | 0.005163 | 0.172117 | SNORA12 | 10 |
| ENSG00000104881 | 142.1917 | 87.73683 | 0.600833 | 0.00538 | 0.17765 | PPP1R13L | 19 |
| ENSG00000078900 | 9.764614 | 0.372055 | 0.735028 | 0.005454 | 1 | TP73 | 1 |
| ENSG00000138092 | 258.1268 | 157.7433 | 0.606285 | 0.005551 | 0.181544 | CENPO | 2 |
| ENSG00000101444 | 450.5436 | 306.6611 | 0.504293 | 0.005694 | 0.184477 | AHCY | 20 |
| ENSG00000132964 | 31.84184 | 11.08968 | 0.848764 | 0.005718 | 1 | CDK8 | 13 |
| ENSG00000004700 | 81.18995 | 43.20115 | 0.706117 | 0.005825 | 1 | RECQL | 12 |
| ENSG00000254986 | 115.9186 | 65.69831 | 0.66872 | 0.005828 | 0.187053 | DPP3 | 11 |
| ENSG00000100567 | 786.343 | 570.9942 | 0.42932 | 0.006004 | 0.189238 | PSMA3 | 14 |
| ENSG00000089154 | 248.466 | 166.82 | 0.515768 | 0.006056 | 0.189238 | GCN1 | 12 |
| ENSG00000237973 | 17.86418 | 46.14143 | -0.82851 | 0.006062 | 1 | MTCO1P12 | 1 |
| ENSG00000003436 | 460.6969 | 690.652 | -0.52272 | 0.006101 | 0.189238 | TFPI | 2 |
| ENSG00000136938 | 533.4638 | 367.5751 | 0.48739 | 0.006117 | 0.189238 | ANP32B | 9 |
| ENSG00000100219 | 135.5826 | 80.31415 | 0.626836 | 0.006263 | 0.189492 | XBP1 | 22 |
| ENSG00000198727 | 569.4538 | 799.2756 | -0.45171 | 0.006384 | 0.189492 | MT-CYB | MT |
| ENSG00000143621 | 289.445 | 196.3786 | 0.504246 | 0.006408 | 0.189492 | ILF2 | 1 |
| ENSG00000051523 | 200.0904 | 305.5226 | -0.54372 | 0.006478 | 0.189492 | CYBA | 16 |
| ENSG00000132470 | 523.5583 | 707.2375 | -0.40689 | 0.006535 | 0.189492 | ITGB4 | 17 |
| ENSG00000164733 | 1256.472 | 1708.573 | -0.41432 | 0.006583 | 0.189492 | CTSB | 8 |
| ENSG00000207714 | 15.98357 | 44.29688 | -0.83242 | 0.006615 | 1 | MIR584 | 5 |
| ENSG00000197694 | 530.7185 | 354.2714 | 0.523092 | 0.006624 | 0.189492 | SPTAN1 | 9 |
| ENSG00000173442 | 82.37011 | 133.7031 | -0.595 | 0.006631 | 0.189492 | EHBP1L1 | 11 |
| ENSG00000153187 | 631.3338 | 442.3339 | 0.471451 | 0.006635 | 0.189492 | HNRNPU | 1 |
| ENSG00000149100 | 622.1439 | 456.8284 | 0.417367 | 0.006677 | 0.189492 | EIF3M | 11 |
| ENSG00000065328 | 51.31246 | 23.33368 | 0.780411 | 0.006757 | 1 | MCM10 | 10 |
| ENSG00000253894 | 11.57337 | 30.83839 | -0.82385 | 0.006836 | 1 | AC011124.2 | 8 |
| ENSG00000104763 | 273.9627 | 388.1771 | -0.46089 | 0.006962 | 0.195964 | ASAH1 | 8 |
| ENSG00000151353 | 97.98713 | 51.09765 | 0.712471 | 0.007247 | 0.20152 | TMEM18 | 2 |
| ENSG00000134955 | 105.3622 | 166.2875 | -0.57068 | 0.007332 | 0.20152 | SLC37A2 | 11 |
| ENSG00000212607 | 2029.801 | 5216.866 | -0.81405 | 0.007335 | 0.20152 | SNORA3B | 11 |
| ENSG00000206702 | 44.31708 | 91.79328 | -0.74798 | 0.007396 | 1 | RNU1-11P | 6 |
| ENSG00000137154 | 1777.898 | 1267.975 | 0.448912 | 0.007423 | 0.202312 | RPS6 | 9 |
| ENSG00000175334 | 1180.721 | 812.7365 | 0.486576 | 0.007556 | 0.204322 | BANF1 | 11 |
| ENSG00000257907 | 23.29192 | 6.002879 | 0.821935 | 0.007611 | 1 | EEF1A1P17 | 12 |
| ENSG00000113580 | 165.8786 | 245.5565 | -0.506 | 0.00783 | 0.210052 | NR3C1 | 5 |
| ENSG00000199436 | 0.830187 | 10.36152 | -0.70928 | 0.00807 | 1 | SNORD9 | 14 |
| ENSG00000117395 | 183.4988 | 119.811 | 0.538458 | 0.008139 | 0.21665 | EBNA1BP2 | 1 |
| ENSG00000184117 | 81.68806 | 135.074 | -0.60326 | 0.008379 | 0.221332 | NIPSNAP1 | 22 |
| ENSG00000173545 | 59.57812 | 30.56521 | 0.715204 | 0.008395 | 1 | ZNF622 | 5 |
| ENSG00000249784 | 659.5828 | 1050.993 | -0.5737 | 0.008519 | 0.223309 | SCARNA22 | 4 |
| ENSG00000176624 | 29.50286 | 61.32695 | -0.74198 | 0.008579 | 1 | MEX3C | 18 |
| ENSG00000007255 | 2.875302 | 13.64463 | -0.78783 | 0.008626 | 1 | TRAPPC6A | 19 |
| ENSG00000124942 | 3971.263 | 2722.335 | 0.490062 | 0.008745 | 0.223944 | AHNAK | 11 |
| ENSG00000283696 | 91.66575 | 158.8954 | -0.63649 | 0.008768 | 0.223944 | AL592295.4 | 1 |
| ENSG00000006468 | 36.21301 | 70.76068 | -0.71281 | 0.008781 | 1 | ETV1 | 7 |
| ENSG00000136997 | 396.8127 | 269.0523 | 0.502861 | 0.008791 | 0.223944 | MYC | 8 |
| ENSG00000050405 | 168.5905 | 106.7059 | 0.569886 | 0.008804 | 0.223944 | LIMA1 | 12 |
| ENSG00000153395 | 95.14798 | 149.7599 | -0.56261 | 0.008941 | 0.225752 | LPCAT1 | 5 |
| ENSG00000041982 | 112.6087 | 68.58247 | 0.598981 | 0.009033 | 0.226417 | TNC | 9 |
| ENSG00000089248 | 328.4699 | 212.8849 | 0.539678 | 0.009214 | 0.229273 | ERP29 | 12 |
| ENSG00000100316 | 1246.925 | 857.1718 | 0.485003 | 0.009323 | 0.230324 | RPL3 | 22 |
| ENSG00000221740 | 2851.62 | 4497.625 | -0.56313 | 0.009407 | 0.230748 | SNORD93 | 7 |
| ENSG00000035862 | 80.15474 | 128.9353 | -0.57946 | 0.009514 | 0.231717 | TIMP2 | 17 |
| ENSG00000119899 | 152.9703 | 234.3681 | -0.53566 | 0.00968 | 0.232921 | SLC17A5 | 6 |
| ENSG00000117862 | 147.052 | 87.50541 | 0.611624 | 0.009699 | 0.232921 | TXNDC12 | 1 |
| ENSG00000227583 | 33.7035 | 11.17494 | 0.800749 | 0.009749 | 1 | RPS3AP37 | 10 |
| ENSG00000165119 | 585.2277 | 407.476 | 0.472177 | 0.00978 | 0.233224 | HNRNPK | 9 |
| ENSG00000234948 | 0.247505 | 7.849543 | -0.64141 | 0.00982 | 1 | LINC01524 | 20 |
| ENSG00000111678 | 13.13898 | 33.57267 | -0.78671 | 0.00988 | 1 | C12orf57 | 12 |
| ENSG00000206885 | 39.5534 | 77.4004 | -0.70395 | 0.010067 | 1 | SNORA75 | 2 |
| ENSG00000130402 | 1374.23 | 1009.804 | 0.41341 | 0.010107 | 0.236263 | ACTN4 | 19 |
| ENSG00000201818 | 183.6753 | 285.968 | -0.55093 | 0.01011 | 0.236263 | RNY4P17 | 4 |
| ENSG00000198840 | 133.3671 | 204.2947 | -0.53479 | 0.010142 | 0.236263 | MT-ND3 | MT |
| ENSG00000083845 | 1424.44 | 1016.251 | 0.445038 | 0.010183 | 0.236263 | RPS5 | 19 |
| ENSG00000070669 | 19.86083 | 5.594999 | 0.791608 | 0.01028 | 1 | ASNS | 7 |
| ENSG00000164128 | 1.664192 | 10.74072 | -0.73035 | 0.010491 | 1 | NPY1R | 4 |
| ENSG00000092969 | 130.7222 | 196.2843 | -0.51475 | 0.010493 | 0.241839 | TGFB2 | 1 |
| ENSG00000083720 | 38.2671 | 16.54853 | 0.762967 | 0.010613 | 1 | OXCT1 | 5 |
| ENSG00000159131 | 204.666 | 136.2288 | 0.515739 | 0.010652 | 0.242663 | GART | 21 |
| ENSG00000096746 | 445.5491 | 316.6544 | 0.446563 | 0.01067 | 0.242663 | HNRNPH3 | 10 |
| ENSG00000209042 | 26.73074 | 62.67164 | -0.76456 | 0.010748 | 1 | SNORD12C | 20 |
| ENSG00000082497 | 8.922974 | 24.63309 | -0.78571 | 0.010974 | 1 | SERTAD4 | 1 |
| ENSG00000224597 | 93.04357 | 49.72406 | 0.675116 | 0.011029 | 1 | SVIL-AS1 | 10 |
| ENSG00000188895 | 12.3197 | 30.87188 | -0.77384 | 0.011113 | 1 | MSL1 | 17 |
| ENSG00000123091 | 29.36212 | 57.53867 | -0.70375 | 0.011149 | 1 | RNF11 | 1 |
| ENSG00000146278 | 34.13058 | 66.97586 | -0.70617 | 0.011181 | 1 | PNRC1 | 6 |
| ENSG00000070413 | 53.98711 | 92.084 | -0.62267 | 0.01121 | 1 | DGCR2 | 22 |
| ENSG00000084234 | 1708.706 | 2183.256 | -0.33642 | 0.011413 | 0.254158 | APLP2 | 11 |
| ENSG00000106028 | 398.3221 | 268.3802 | 0.502894 | 0.011437 | 0.254158 | SSBP1 | 7 |
| ENSG00000138738 | 24.45721 | 8.534726 | 0.782482 | 0.01148 | 1 | PRDM5 | 4 |
| ENSG00000125730 | 227.8436 | 323.1235 | -0.45446 | 0.011489 | 0.254158 | C3 | 19 |
| ENSG00000106628 | 270.4702 | 185.6134 | 0.480935 | 0.011513 | 0.254158 | POLD2 | 7 |
| ENSG00000156508 | 1466.883 | 1106.372 | 0.381374 | 0.011546 | 0.254158 | EEF1A1 | 6 |
| ENSG00000215093 | 33.26483 | 12.13096 | 0.779223 | 0.01164 | 1 | EEF1A1P29 | X |
| ENSG00000129757 | 7.971063 | 23.22298 | -0.78159 | 0.011652 | 1 | CDKN1C | 11 |
| ENSG00000101017 | 64.19686 | 106.3603 | -0.59904 | 0.01181 | 0.25831 | CD40 | 20 |
| ENSG00000204580 | 34.89982 | 66.00085 | -0.6835 | 0.011847 | 1 | DDR1 | 6 |
| ENSG00000127837 | 305.1168 | 186.5832 | 0.588019 | 0.011887 | 0.258357 | AAMP | 2 |
| ENSG00000060656 | 6.725902 | 21.4974 | -0.77713 | 0.011998 | 1 | PTPRU | 1 |
| ENSG00000100297 | 75.67062 | 40.83445 | 0.672533 | 0.012006 | 1 | MCM5 | 22 |
| ENSG00000137054 | 87.5739 | 49.33892 | 0.649439 | 0.01209 | 1 | POLR1E | 9 |
| ENSG00000200879 | 5.171739 | 20.6236 | -0.76311 | 0.012109 | 1 | SNORD14E | 11 |
| ENSG00000226752 | 2.540125 | 12.47669 | -0.73965 | 0.012126 | 1 | CUTALP | 9 |
| ENSG00000162729 | 41.17264 | 73.81098 | -0.65028 | 0.012283 | 1 | IGSF8 | 1 |
| ENSG00000115839 | 232.0901 | 159.124 | 0.486268 | 0.012362 | 0.262713 | RAB3GAP1 | 2 |
| ENSG00000136156 | 874.922 | 1193.721 | -0.4132 | 0.012399 | 0.262713 | ITM2B | 13 |
| ENSG00000181163 | 718.0574 | 526.061 | 0.412845 | 0.012446 | 0.262713 | NPM1 | 5 |
| ENSG00000083444 | 177.6578 | 261.1314 | -0.49308 | 0.012461 | 0.262713 | PLOD1 | 1 |
| ENSG00000169919 | 174.4837 | 258.223 | -0.50188 | 0.01247 | 0.262713 | GUSB | 7 |
| ENSG00000182199 | 534.9332 | 400.4738 | 0.388309 | 0.012632 | 0.264495 | SHMT2 | 12 |
| ENSG00000207445 | 30.44696 | 74.03189 | -0.75622 | 0.01271 | 1 | SNORD15B | 11 |
| ENSG00000234036 | 37.11254 | 10.31923 | 0.764244 | 0.012787 | 1 | TXNP6 | 4 |
| ENSG00000166710 | 2602.056 | 3492.842 | -0.3951 | 0.012858 | 0.2676 | B2M | 15 |
| ENSG00000242114 | 0.247505 | 7.095941 | -0.60878 | 0.013043 | 1 | MTFP1 | 22 |
| ENSG00000243279 | 39.65356 | 72.63246 | -0.66574 | 0.013089 | 1 | PRAF2 | X |
| ENSG00000087086 | 571.4758 | 826.8609 | -0.4771 | 0.013099 | 0.269412 | FTL | 19 |
| ENSG00000128708 | 266.6981 | 181.4237 | 0.488975 | 0.013123 | 0.269412 | HAT1 | 2 |
| ENSG00000202400 | 44.75943 | 86.71347 | -0.68451 | 0.013132 | 1 | SNORD82 | 2 |
| ENSG00000033100 | 117.9522 | 176.6232 | -0.50695 | 0.01318 | 0.269412 | CHPF2 | 7 |
| ENSG00000119004 | 160.3611 | 97.28631 | 0.585267 | 0.013272 | 0.269688 | CYP20A1 | 2 |
| ENSG00000134825 | 57.7728 | 105.8224 | -0.65971 | 0.013419 | 0.271057 | TMEM258 | 11 |
| ENSG00000103024 | 38.09884 | 68.13876 | -0.64398 | 0.01355 | 1 | NME3 | 16 |
| ENSG00000161016 | 2619.687 | 1856.031 | 0.449035 | 0.013618 | 0.272479 | RPL8 | 8 |
| ENSG00000092199 | 684.6647 | 477.3904 | 0.466634 | 0.013648 | 0.272479 | HNRNPC | 14 |
| ENSG00000067182 | 341.9704 | 464.4426 | -0.40811 | 0.013851 | 0.273578 | TNFRSF1A | 12 |
| ENSG00000153485 | 1.957442 | 11.42179 | -0.70195 | 0.013854 | 1 | TMEM251 | 14 |
| ENSG00000167526 | 454.5331 | 325.736 | 0.434841 | 0.013862 | 0.273578 | RPL13 | 16 |
| ENSG00000183665 | 1.458615 | 10.79161 | -0.67547 | 0.014 | 1 | TRMT12 | 8 |
| ENSG00000066422 | 25.71981 | 7.465657 | 0.754078 | 0.01424 | 1 | ZBTB11 | 3 |
| ENSG00000204272 | 29.4544 | 57.49727 | -0.69225 | 0.01429 | 1 | NBDY | X |
| ENSG00000138363 | 266.5542 | 179.6122 | 0.499889 | 0.014341 | 0.281417 | ATIC | 2 |
| ENSG00000130827 | 22.328 | 46.43324 | -0.70736 | 0.014494 | 1 | PLXNA3 | X |
| ENSG00000116171 | 153.7919 | 219.4888 | -0.46023 | 0.014671 | 0.286257 | SCP2 | 1 |
| ENSG00000112655 | 277.3918 | 382.2343 | -0.42075 | 0.014843 | 0.287624 | PTK7 | 6 |
| ENSG00000105976 | 325.54 | 452.2578 | -0.4294 | 0.014909 | 0.287624 | MET | 7 |
| ENSG00000251718 | 16.10196 | 4.378512 | 0.744852 | 0.014911 | 1 | RNU2-13P | 2 |
| ENSG00000102359 | 5.06092 | 19.22545 | -0.74028 | 0.014988 | 1 | SRPX2 | X |
| ENSG00000201291 | 5.209849 | 17.51728 | -0.74853 | 0.015056 | 1 | RNU1-34P | 6 |
| ENSG00000146411 | 3.530145 | 15.07392 | -0.7285 | 0.015114 | 1 | SLC2A12 | 6 |
| ENSG00000207105 | 70.88671 | 119.6878 | -0.60691 | 0.015345 | 0.294381 | RF00019 | 15 |
| ENSG00000249471 | 3.66738 | 15.9755 | -0.71966 | 0.015497 | 1 | ZNF324B | 19 |
| ENSG00000125651 | 106.5229 | 65.81972 | 0.57514 | 0.015501 | 0.295728 | GTF2F1 | 19 |
| ENSG00000234745 | 216.2258 | 305.8571 | -0.44926 | 0.01574 | 0.29862 | HLA-B | 6 |
| ENSG00000156453 | 8.469891 | 0.619795 | 0.629015 | 0.015793 | 1 | PCDH1 | 5 |
| ENSG00000206828 | 16.28446 | 3.236903 | 0.705178 | 0.015802 | 1 | RF00003 | 1 |
| ENSG00000158805 | 7.486957 | 0.309898 | 0.603249 | 0.015862 | 1 | ZNF276 | 16 |
| ENSG00000073803 | 173.9672 | 113.4299 | 0.522809 | 0.016022 | 0.299151 | MAP3K13 | 3 |
| ENSG00000152556 | 150.1618 | 95.0902 | 0.554594 | 0.016023 | 0.299151 | PFKM | 12 |
| ENSG00000107331 | 49.7863 | 82.90816 | -0.59151 | 0.016079 | 1 | ABCA2 | 9 |
| ENSG00000113282 | 131.0412 | 75.6261 | 0.612795 | 0.016115 | 0.299151 | CLINT1 | 5 |
| ENSG00000104823 | 94.63037 | 146.6127 | -0.53813 | 0.016142 | 0.299151 | ECH1 | 19 |
| ENSG00000164283 | 61.55186 | 29.7894 | 0.70354 | 0.016185 | 1 | ESM1 | 5 |
| ENSG00000204386 | 143.5495 | 211.4558 | -0.49289 | 0.016203 | 0.299151 | NEU1 | 6 |
| ENSG00000114859 | 8.83936 | 23.90259 | -0.74449 | 0.01621 | 1 | CLCN2 | 3 |
| ENSG00000095485 | 39.13461 | 16.93957 | 0.723649 | 0.016291 | 1 | CWF19L1 | 10 |
| ENSG00000263932 | 7.891026 | 23.73441 | -0.74359 | 0.016349 | 1 | MIR4448 | 3 |
| ENSG00000135047 | 556.5377 | 725.1619 | -0.35764 | 0.016519 | 0.300786 | CTSL | 9 |
| ENSG00000198832 | 107.8759 | 163.9805 | -0.5178 | 0.016544 | 0.300786 | SELENOM | 22 |
| ENSG00000170498 | 350.7062 | 481.5361 | -0.41819 | 0.016555 | 0.300786 | KISS1 | 1 |
| ENSG00000084674 | 1.08151 | 8.98166 | -0.64467 | 0.016559 | 1 | APOB | 2 |
| ENSG00000239043 | 7.197524 | 22.92992 | -0.74042 | 0.016596 | 1 | SNORD127 | 14 |
| ENSG00000205339 | 312.0736 | 216.0265 | 0.467141 | 0.016701 | 0.300857 | IPO7 | 11 |
| ENSG00000152234 | 1351.927 | 1020.129 | 0.378783 | 0.016784 | 0.300857 | ATP5F1A | 18 |
| ENSG00000101191 | 91.72676 | 53.39481 | 0.616717 | 0.016833 | 1 | DIDO1 | 20 |
| ENSG00000139514 | 435.5219 | 316.927 | 0.416822 | 0.01694 | 0.300857 | SLC7A1 | 13 |
| ENSG00000121390 | 87.71275 | 51.34249 | 0.60949 | 0.016947 | 1 | PSPC1 | 13 |
| ENSG00000130713 | 204.1528 | 136.7369 | 0.501767 | 0.016977 | 0.300857 | EXOSC2 | 9 |
| ENSG00000137876 | 163.8846 | 100.7948 | 0.571874 | 0.017065 | 0.300857 | RSL24D1 | 15 |
| ENSG00000206503 | 683.9989 | 1007.763 | -0.48972 | 0.017084 | 0.300857 | HLA-A | 6 |
| ENSG00000076201 | 126.4378 | 72.90383 | 0.617597 | 0.017257 | 0.302346 | PTPN23 | 3 |
| ENSG00000138764 | 68.86237 | 108.9315 | -0.55099 | 0.017437 | 0.303369 | CCNG2 | 4 |
| ENSG00000202111 | 46.42138 | 83.69063 | -0.63815 | 0.017468 | 1 | VTRNA1-2 | 5 |
| ENSG00000163584 | 120.5097 | 72.7281 | 0.578506 | 0.017492 | 0.303369 | RPL22L1 | 3 |
| ENSG00000231164 | 8.751447 | 0.74411 | 0.618231 | 0.017598 | 1 | RPL7P56 | X |
| ENSG00000165934 | 241.4593 | 160.1041 | 0.510148 | 0.017992 | 0.309521 | CPSF2 | 14 |
| ENSG00000080345 | 190.4679 | 130.4807 | 0.481466 | 0.018027 | 0.309521 | RIF1 | 2 |
| ENSG00000177685 | 11.70679 | 27.87322 | -0.72214 | 0.018106 | 1 | CRACR2B | 11 |
| ENSG00000077454 | 5.373499 | 17.35068 | -0.72643 | 0.018232 | 1 | LRCH4 | 7 |
| ENSG00000163918 | 192.7827 | 125.4144 | 0.525155 | 0.018354 | 0.313564 | RFC4 | 3 |
| ENSG00000283871 | 9.036511 | 26.92903 | -0.72965 | 0.01846 | 1 | MIR130B | 22 |
| ENSG00000125534 | 149.71 | 233.7181 | -0.54292 | 0.018487 | 0.313729 | PPDPF | 20 |
| ENSG00000160255 | 92.22494 | 145.7212 | -0.54937 | 0.018546 | 0.313729 | ITGB2 | 21 |
| ENSG00000206838 | 39.70862 | 75.32586 | -0.65409 | 0.01879 | 1 | SNORA5A | 7 |
| ENSG00000241469 | 0.247505 | 7.404389 | -0.55612 | 0.019006 | 1 | LINC00635 | 3 |
| ENSG00000181690 | 2.87912 | 12.82798 | -0.68947 | 0.019079 | 1 | PLAG1 | 8 |
| ENSG00000177189 | 51.466 | 85.44894 | -0.58424 | 0.019086 | 1 | RPS6KA3 | X |
| ENSG00000071553 | 170.1052 | 253.8284 | -0.50087 | 0.019111 | 0.31986 | ATP6AP1 | X |
| ENSG00000154518 | 496.6959 | 373.348 | 0.382746 | 0.019228 | 0.31986 | ATP5MC3 | 2 |
| ENSG00000084774 | 125.3055 | 79.88907 | 0.545176 | 0.019363 | 0.31986 | CAD | 2 |
| ENSG00000137776 | 100.2428 | 61.30094 | 0.57323 | 0.019392 | 0.31986 | SLTM | 15 |
| ENSG00000229798 | 29.35462 | 8.942606 | 0.717717 | 0.019495 | 1 | KRT18P26 | 2 |
| ENSG00000151458 | 79.71885 | 42.56181 | 0.653451 | 0.01955 | 1 | ANKRD50 | 4 |
| ENSG00000135677 | 393.3123 | 520.2958 | -0.37306 | 0.019629 | 0.31986 | GNS | 12 |
| ENSG00000151414 | 71.31459 | 112.9933 | -0.55014 | 0.01963 | 0.31986 | NEK7 | 1 |
| ENSG00000141522 | 882.9224 | 644.1371 | 0.41396 | 0.019647 | 0.31986 | ARHGDIA | 17 |
| ENSG00000136628 | 554.0855 | 391.8682 | 0.44827 | 0.019717 | 0.31986 | EPRS | 1 |
| ENSG00000275714 | 239.7639 | 372.1658 | -0.53446 | 0.019765 | 0.31986 | HIST1H3A | 6 |
| ENSG00000132823 | 226.1029 | 148.1734 | 0.513717 | 0.019887 | 0.31986 | OSER1 | 20 |
| ENSG00000165732 | 148.2039 | 95.66804 | 0.534424 | 0.01994 | 0.31986 | DDX21 | 10 |
| ENSG00000198715 | 54.79384 | 91.51186 | -0.58652 | 0.019941 | 1 | GLMP | 1 |
| ENSG00000143436 | 113.1647 | 73.18239 | 0.527264 | 0.020026 | 0.31986 | MRPL9 | 1 |
| ENSG00000107140 | 4.836562 | 16.19869 | -0.71168 | 0.020036 | 1 | TESK1 | 9 |
| ENSG00000030582 | 2058.074 | 2684.024 | -0.35775 | 0.02015 | 0.320102 | GRN | 17 |
| ENSG00000163125 | 60.85709 | 33.43151 | 0.636336 | 0.020391 | 1 | RPRD2 | 1 |
| ENSG00000125630 | 93.10341 | 56.27719 | 0.578007 | 0.020402 | 0.320102 | POLR1B | 2 |
| ENSG00000047188 | 46.83533 | 22.30779 | 0.691096 | 0.020414 | 1 | YTHDC2 | 5 |
| ENSG00000166226 | 315.5993 | 226.9152 | 0.428952 | 0.020439 | 0.320102 | CCT2 | 12 |
| ENSG00000204899 | 376.2461 | 279.6775 | 0.391212 | 0.020464 | 0.320102 | MZT1 | 13 |
| ENSG00000200959 | 168.7351 | 445.9904 | -0.7174 | 0.020568 | 0.320102 | SNORA74A | 5 |
| ENSG00000117691 | 30.28276 | 59.6277 | -0.66381 | 0.020637 | 1 | NENF | 1 |
| ENSG00000198712 | 602.514 | 877.3802 | -0.47292 | 0.020651 | 0.320102 | MT-CO2 | MT |
| ENSG00000010803 | 1.99937 | 10.13365 | -0.66184 | 0.020676 | 1 | SCMH1 | 1 |
| ENSG00000163346 | 152.2503 | 215.5144 | -0.44806 | 0.020694 | 0.320102 | PBXIP1 | 1 |
| ENSG00000144233 | 43.27464 | 20.21463 | 0.686892 | 0.020698 | 1 | AMMECR1L | 2 |
| ENSG00000077616 | 2.155384 | 12.93008 | -0.64289 | 0.021071 | 1 | NAALAD2 | 11 |
| ENSG00000010278 | 928.8911 | 1209.006 | -0.35552 | 0.021096 | 0.324411 | CD9 | 12 |
| ENSG00000156017 | 28.56677 | 11.71985 | 0.708078 | 0.021134 | 1 | CARNMT1 | 9 |
| ENSG00000206925 | 96.03186 | 152.1895 | -0.54509 | 0.021161 | 0.324411 | RF00019 | 19 |
| ENSG00000199135 | 14.12385 | 2.382089 | 0.640249 | 0.021411 | 1 | MIR101-1 | 1 |
| ENSG00000266922 | 4.090229 | 14.51305 | -0.69642 | 0.021566 | 1 | AC008543.3 | 19 |
| ENSG00000178035 | 413.8693 | 312.9553 | 0.372699 | 0.021574 | 0.328405 | IMPDH2 | 3 |
| ENSG00000108846 | 24.769 | 47.4068 | -0.65201 | 0.021603 | 1 | ABCC3 | 17 |
| ENSG00000187051 | 101.8428 | 59.74466 | 0.59202 | 0.021613 | 0.328405 | RPS19BP1 | 22 |
| ENSG00000129038 | 22.20628 | 44.48605 | -0.67156 | 0.021652 | 1 | LOXL1 | 15 |
| ENSG00000047644 | 35.74146 | 64.04939 | -0.62076 | 0.021755 | 1 | WWC3 | X |
| ENSG00000099194 | 276.519 | 376.91 | -0.40642 | 0.021876 | 0.330928 | SCD | 10 |
| ENSG00000088205 | 80.83998 | 44.74203 | 0.626495 | 0.02195 | 1 | DDX18 | 2 |
| ENSG00000125505 | 577.5062 | 801.3504 | -0.42629 | 0.022303 | 0.33591 | MBOAT7 | 19 |
| ENSG00000224051 | 9.47845 | 27.17051 | -0.70746 | 0.022384 | 1 | CPTP | 1 |
| ENSG00000171448 | 8.085151 | 0.74411 | 0.589078 | 0.022508 | 1 | ZBTB26 | 9 |
| ENSG00000274452 | 72.94205 | 234.9862 | -0.6986 | 0.02259 | 0.337723 | RF00004 | 17 |
| ENSG00000173465 | 258.1007 | 181.4857 | 0.452267 | 0.02271 | 0.337723 | ZNRD2 | 11 |
| ENSG00000089009 | 237.4706 | 164.1382 | 0.470301 | 0.02272 | 0.337723 | AC004086.1 | 12 |
| ENSG00000113460 | 148.5374 | 96.69104 | 0.520121 | 0.022853 | 0.337723 | BRIX1 | 5 |
| ENSG00000143429 | 4.707203 | 17.28763 | -0.68245 | 0.022901 | 1 | LSP1P4 | 2 |
| ENSG00000185022 | 44.56482 | 79.16862 | -0.61305 | 0.022913 | 1 | MAFF | 22 |
| ENSG00000107175 | 143.6166 | 93.87441 | 0.515978 | 0.022915 | 0.337723 | CREB3 | 9 |
| ENSG00000060138 | 91.62179 | 54.35944 | 0.587579 | 0.023179 | 1 | YBX3 | 12 |
| ENSG00000115363 | 96.74421 | 58.39457 | 0.577909 | 0.023377 | 0.343063 | EVA1A | 2 |
| ENSG00000126062 | 91.50708 | 135.729 | -0.48795 | 0.023689 | 0.345321 | TMEM115 | 3 |
| ENSG00000160953 | 22.50716 | 8.534726 | 0.700437 | 0.023815 | 1 | PWWP3A | 19 |
| ENSG00000107581 | 434.8268 | 321.6367 | 0.397419 | 0.023833 | 0.345321 | EIF3A | 10 |
| ENSG00000090339 | 785.8088 | 1034.684 | -0.3663 | 0.023841 | 0.345321 | ICAM1 | 19 |
| ENSG00000148672 | 223.1372 | 159.6166 | 0.432708 | 0.023936 | 0.345321 | GLUD1 | 10 |
| ENSG00000167779 | 460.4842 | 604.5421 | -0.36329 | 0.024124 | 0.345321 | IGFBP6 | 12 |
| ENSG00000267195 | 4.356514 | 16.28807 | -0.6772 | 0.024126 | 1 | MIR212 | 17 |
| ENSG00000139182 | 69.69965 | 110.356 | -0.53757 | 0.024134 | 0.345321 | CLSTN3 | 12 |
| ENSG00000207313 | 4.627167 | 15.4173 | -0.68617 | 0.024252 | 1 | SNORA2B | 12 |
| ENSG00000160783 | 20.3556 | 7.158099 | 0.696587 | 0.024375 | 1 | PMF1 | 1 |
| ENSG00000120451 | 58.37052 | 93.12315 | -0.54467 | 0.024452 | 0.348412 | SNX19 | 11 |
| ENSG00000170006 | 27.30689 | 10.75345 | 0.695537 | 0.024496 | 1 | TMEM154 | 4 |
| ENSG00000240682 | 0.247505 | 6.029212 | -0.52829 | 0.024515 | 1 | ISY1 | 3 |
| ENSG00000166123 | 72.69856 | 42.89214 | 0.583151 | 0.024742 | 1 | GPT2 | 16 |
| ENSG00000251333 | 25.85901 | 8.880448 | 0.694041 | 0.024807 | 1 | RTN3P1 | 4 |
| ENSG00000132842 | 79.92474 | 47.56872 | 0.581382 | 0.024849 | 1 | AP3B1 | 5 |
| ENSG00000123136 | 386.4912 | 286.863 | 0.390988 | 0.024886 | 0.351781 | DDX39A | 19 |
| ENSG00000141526 | 494.7523 | 654.268 | -0.37182 | 0.024893 | 0.351781 | SLC16A3 | 17 |
| ENSG00000185610 | 7.250905 | 21.83452 | -0.69138 | 0.024986 | 1 | DBX2 | 12 |
| ENSG00000003096 | 2.699957 | 11.90544 | -0.64954 | 0.025291 | 1 | KLHL13 | X |
| ENSG00000101000 | 163.7568 | 101.8069 | 0.554443 | 0.025349 | 0.356737 | PROCR | 20 |
| ENSG00000128272 | 78.42062 | 46.60198 | 0.57977 | 0.025409 | 1 | ATF4 | 22 |
| ENSG00000117143 | 169.7175 | 112.6042 | 0.497275 | 0.025544 | 0.356737 | UAP1 | 1 |
| ENSG00000226284 | 6.763771 | 0.372055 | 0.545503 | 0.025576 | 1 | ARPC3P1 | 20 |
| ENSG00000132382 | 120.7399 | 78.68655 | 0.51197 | 0.025711 | 0.356737 | MYBBP1A | 17 |
| ENSG00000142949 | 188.1526 | 257.9388 | -0.41232 | 0.025736 | 0.356737 | PTPRF | 1 |
| ENSG00000114942 | 282.1374 | 196.5811 | 0.456585 | 0.025763 | 0.356737 | EEF1B2 | 2 |
| ENSG00000108774 | 68.77446 | 109.8655 | -0.54019 | 0.026035 | 0.358673 | RAB5C | 17 |
| ENSG00000261236 | 123.2716 | 81.25835 | 0.50573 | 0.026112 | 0.358673 | BOP1 | 8 |
| ENSG00000266104 | 43.22515 | 76.35822 | -0.60299 | 0.026404 | 1 | MIR4326 | 20 |
| ENSG00000105723 | 41.77307 | 70.67598 | -0.58026 | 0.026441 | 1 | GSK3A | 19 |
| ENSG00000278172 | 0.875932 | 7.569209 | -0.57224 | 0.026509 | 1 | RF00017 | 13 |
| ENSG00000134107 | 27.88799 | 51.18862 | -0.62212 | 0.026516 | 1 | BHLHE40 | 3 |
| ENSG00000076043 | 203.0396 | 144.033 | 0.436451 | 0.026523 | 0.362863 | REXO2 | 11 |
| ENSG00000173402 | 452.6448 | 598.5014 | -0.37238 | 0.026784 | 0.363573 | DAG1 | 3 |
| ENSG00000059804 | 124.9463 | 188.588 | -0.50112 | 0.026786 | 0.363573 | SLC2A3 | 12 |
| ENSG00000140396 | 32.04766 | 14.03889 | 0.675934 | 0.02691 | 1 | NCOA2 | 8 |
| ENSG00000116062 | 84.63927 | 46.01854 | 0.627811 | 0.026972 | 1 | MSH6 | 2 |
| ENSG00000149932 | 41.26382 | 69.75812 | -0.58318 | 0.026976 | 1 | TMEM219 | 16 |
| ENSG00000131023 | 24.35761 | 47.16051 | -0.64016 | 0.027021 | 1 | LATS1 | 6 |
| ENSG00000222293 | 19.83689 | 50.58484 | -0.68439 | 0.027103 | 1 | RNU2-36P | 9 |
| ENSG00000248180 | 24.98992 | 6.91985 | 0.66443 | 0.027108 | 1 | GAPDHP60 | 4 |
| ENSG00000198900 | 182.8075 | 117.1074 | 0.529568 | 0.027119 | 0.364374 | TOP1 | 20 |
| ENSG00000168003 | 1292.495 | 1000.992 | 0.343332 | 0.027157 | 0.364374 | SLC3A2 | 11 |
| ENSG00000189060 | 161.7365 | 228.2314 | -0.44145 | 0.027164 | 0.364374 | H1F0 | 22 |
| ENSG00000135316 | 207.8953 | 138.0813 | 0.496953 | 0.027328 | 0.365159 | SYNCRIP | 6 |
| ENSG00000151694 | 145.3656 | 203.4087 | -0.43115 | 0.027451 | 0.365378 | ADAM17 | 2 |
| ENSG00000088812 | 126.9855 | 178.4442 | -0.43524 | 0.027592 | 0.365835 | ATRN | 20 |
| ENSG00000103811 | 77.70634 | 118.7961 | -0.51104 | 0.02772 | 0.366117 | CTSH | 15 |
| ENSG00000101654 | 83.05948 | 52.16059 | 0.541654 | 0.027978 | 1 | RNMT | 18 |
| ENSG00000188493 | 29.34636 | 54.01049 | -0.61621 | 0.028171 | 1 | C19orf54 | 19 |
| ENSG00000138182 | 123.0818 | 78.85348 | 0.526527 | 0.028174 | 0.370684 | KIF20B | 10 |
| ENSG00000123395 | 87.46976 | 52.81298 | 0.563629 | 0.028198 | 1 | ATG101 | 12 |
| ENSG00000267959 | 0.742515 | 7.74207 | -0.55054 | 0.028247 | 1 | MIR3188 | 19 |
| ENSG00000152582 | 12.42288 | 3.249625 | 0.653313 | 0.02841 | 1 | SPEF2 | 5 |
| ENSG00000150403 | 63.70288 | 102.1213 | -0.54346 | 0.028455 | 0.371109 | TMCO3 | 13 |
| ENSG00000202314 | 58.7815 | 97.8717 | -0.56635 | 0.028527 | 0.371109 | SNORD6 | 11 |
| ENSG00000150540 | 74.40795 | 114.1701 | -0.51266 | 0.028701 | 0.371109 | HNMT | 2 |
| ENSG00000100201 | 513.1231 | 387.3014 | 0.374876 | 0.028803 | 0.371109 | DDX17 | 22 |
| ENSG00000171566 | 196.4479 | 130.0702 | 0.50005 | 0.028818 | 0.371109 | PLRG1 | 4 |
| ENSG00000011295 | 138.7271 | 228.9476 | -0.56201 | 0.028854 | 0.371109 | TTC19 | 17 |
| ENSG00000143515 | 8.698066 | 21.86946 | -0.67648 | 0.028954 | 1 | ATP8B2 | 1 |
| ENSG00000164251 | 93.93439 | 138.9156 | -0.48367 | 0.028982 | 0.371306 | F2RL1 | 5 |
| ENSG00000196262 | 1935.067 | 1489.2 | 0.350772 | 0.029226 | 0.371306 | PPIA | 7 |
| ENSG00000111142 | 219.7186 | 158.9952 | 0.419991 | 0.029293 | 0.371306 | METAP2 | 12 |
| ENSG00000138604 | 80.99717 | 121.9318 | -0.49826 | 0.029302 | 0.371306 | GLCE | 15 |
| ENSG00000158186 | 7.068715 | 0.446934 | 0.527699 | 0.029417 | 1 | MRAS | 3 |
| ENSG00000143669 | 12.11031 | 28.5505 | -0.67194 | 0.029432 | 1 | LYST | 1 |
| ENSG00000102543 | 24.76216 | 10.2843 | 0.67251 | 0.029651 | 1 | CDADC1 | 13 |
| ENSG00000177542 | 58.9111 | 94.73927 | -0.54506 | 0.029677 | 0.374678 | SLC25A22 | 11 |
| ENSG00000137478 | 10.65551 | 25.81609 | -0.67172 | 0.02976 | 1 | FCHSD2 | 11 |
| ENSG00000150991 | 606.6583 | 450.5249 | 0.388458 | 0.030017 | 0.376432 | UBC | 12 |
| ENSG00000198380 | 232.7308 | 151.3624 | 0.514817 | 0.030049 | 0.376432 | GFPT1 | 2 |
| ENSG00000163558 | 102.6225 | 66.16132 | 0.518048 | 0.030145 | 0.376432 | PRKCI | 3 |
| ENSG00000130449 | 34.12573 | 60.12262 | -0.59291 | 0.030271 | 1 | ZSWIM6 | 5 |
| ENSG00000197714 | 82.41179 | 49.37633 | 0.5656 | 0.030301 | 1 | ZNF460 | 19 |
| ENSG00000127481 | 382.4532 | 285.6272 | 0.382554 | 0.030459 | 0.378216 | UBR4 | 1 |
| ENSG00000115504 | 48.44583 | 24.60943 | 0.638155 | 0.030487 | 1 | EHBP1 | 2 |
| ENSG00000087087 | 125.2022 | 77.21696 | 0.550269 | 0.030548 | 0.378216 | SRRT | 7 |
| ENSG00000116005 | 180.597 | 253.1652 | -0.43104 | 0.030631 | 0.378216 | PCYOX1 | 2 |
| ENSG00000163950 | 267.2414 | 191.7709 | 0.425523 | 0.030772 | 0.378216 | SLBP | 4 |
| ENSG00000168487 | 41.09993 | 70.62819 | -0.57997 | 0.030841 | 1 | BMP1 | 8 |
| ENSG00000143401 | 566.9164 | 431.5758 | 0.361281 | 0.030906 | 0.378216 | ANP32E | 1 |
| ENSG00000255008 | 41.60264 | 75.73094 | -0.60682 | 0.030929 | 1 | AP000442.1 | 11 |
| ENSG00000008517 | 306.21 | 222.0232 | 0.413549 | 0.030949 | 0.378216 | IL32 | 16 |
| ENSG00000171798 | 5.545026 | 0 | 0.479492 | 0.031004 | 1 | KNDC1 | 10 |
| ENSG00000221716 | 7.410978 | 53.40994 | -0.56089 | 0.031094 | 1 | SNORA11 | X |
| ENSG00000104213 | 24.06078 | 45.35682 | -0.62336 | 0.031137 | 1 | PDGFRL | 8 |
| ENSG00000126464 | 24.08774 | 44.20572 | -0.61335 | 0.031178 | 1 | PRR12 | 19 |
| ENSG00000128591 | 205.9385 | 139.7138 | 0.481055 | 0.031188 | 0.379791 | FLNC | 7 |
| ENSG00000152382 | 61.77622 | 35.89652 | 0.579705 | 0.031252 | 1 | TADA1 | 1 |
| ENSG00000242125 | 64.55518 | 38.19157 | 0.571049 | 0.031298 | 1 | SNHG3 | 1 |
| ENSG00000156970 | 59.49347 | 34.79396 | 0.580393 | 0.031321 | 1 | BUB1B | 15 |
| ENSG00000204498 | 8.797192 | 23.10044 | -0.66685 | 0.031422 | 1 | NFKBIL1 | 6 |
| ENSG00000111530 | 221.9592 | 158.0905 | 0.433061 | 0.031441 | 0.38019 | CAND1 | 12 |
| ENSG00000105426 | 137.9373 | 194.9273 | -0.43705 | 0.031443 | 0.38019 | PTPRS | 19 |
| ENSG00000006712 | 184.959 | 123.1701 | 0.494547 | 0.031665 | 0.38082 | PAF1 | 19 |
| ENSG00000162585 | 11.3226 | 26.7651 | -0.66453 | 0.031679 | 1 | FAAP20 | 1 |
| ENSG00000164904 | 155.8973 | 108.9587 | 0.450847 | 0.031717 | 0.38082 | ALDH7A1 | 5 |
| ENSG00000146143 | 24.32029 | 9.934456 | 0.664557 | 0.031721 | 1 | PRIM2 | 6 |
| ENSG00000120699 | 608.3407 | 415.1601 | 0.471343 | 0.031831 | 0.380861 | EXOSC8 | 13 |
| ENSG00000182492 | 6.146798 | 0 | 0.473231 | 0.031962 | 1 | BGN | X |
| ENSG00000120533 | 453.1822 | 344.4838 | 0.364338 | 0.032045 | 0.381272 | ENY2 | 8 |
| ENSG00000227036 | 1.664192 | 8.744302 | -0.59272 | 0.032046 | 1 | LINC00511 | 17 |
| ENSG00000122966 | 127.1304 | 84.63875 | 0.493979 | 0.032127 | 0.381272 | CIT | 12 |
| ENSG00000120337 | 61.6256 | 32.31155 | 0.62139 | 0.032129 | 1 | TNFSF18 | 1 |
| ENSG00000133740 | 32.74109 | 14.47868 | 0.65901 | 0.032163 | 1 | E2F5 | 8 |
| ENSG00000122390 | 3.865322 | 13.47088 | -0.64217 | 0.032211 | 1 | NAA60 | 16 |
| ENSG00000127920 | 149.1362 | 103.5531 | 0.454777 | 0.032214 | 0.381272 | GNG11 | 7 |
| ENSG00000167635 | 43.47179 | 72.30625 | -0.55927 | 0.032229 | 1 | ZNF146 | 19 |
| ENSG00000151914 | 221.6668 | 158.2523 | 0.432979 | 0.032309 | 0.381272 | DST | 6 |
| ENSG00000093167 | 71.63026 | 41.73692 | 0.57795 | 0.032545 | 1 | LRRFIP2 | 3 |
| ENSG00000091317 | 138.8762 | 196.1042 | -0.4385 | 0.032578 | 0.382461 | CMTM6 | 3 |
| ENSG00000111145 | 25.6147 | 46.30424 | -0.60209 | 0.032614 | 1 | ELK3 | 12 |
| ENSG00000115306 | 259.4467 | 175.766 | 0.479902 | 0.032654 | 0.382461 | SPTBN1 | 2 |
| ENSG00000164611 | 386.0962 | 293.769 | 0.361115 | 0.032744 | 0.382461 | PTTG1 | 5 |
| ENSG00000188483 | 26.39914 | 48.32199 | -0.60867 | 0.032848 | 1 | IER5L | 9 |
| ENSG00000073111 | 82.10757 | 50.53478 | 0.548165 | 0.032874 | 1 | MCM2 | 3 |
| ENSG00000152133 | 40.02175 | 19.96867 | 0.634882 | 0.03291 | 1 | GPATCH11 | 2 |
| ENSG00000144028 | 276.5669 | 188.7917 | 0.473336 | 0.033069 | 0.383719 | SNRNP200 | 2 |
| ENSG00000152332 | 102.1209 | 146.1327 | -0.44902 | 0.033092 | 0.383719 | UHMK1 | 1 |
| ENSG00000145439 | 111.2275 | 69.50679 | 0.538649 | 0.033243 | 0.383719 | CBR4 | 4 |
| ENSG00000199515 | 58.39294 | 97.25827 | -0.55882 | 0.033299 | 0.383719 | RF00019 | 22 |
| ENSG00000092208 | 42.93571 | 22.23113 | 0.626259 | 0.033407 | 1 | GEMIN2 | 14 |
| ENSG00000147364 | 26.52523 | 11.68403 | 0.65423 | 0.033407 | 1 | FBXO25 | 8 |
| ENSG00000099783 | 202.0725 | 128.6302 | 0.526203 | 0.033645 | 0.386412 | HNRNPM | 19 |
| ENSG00000141642 | 7.44582 | 0.681953 | 0.530846 | 0.033777 | 1 | ELAC1 | 18 |
| ENSG00000197153 | 378.9192 | 282.9904 | 0.379486 | 0.033811 | 0.386772 | HIST1H3J | 6 |
| ENSG00000089280 | 256.7534 | 178.7524 | 0.450179 | 0.033902 | 0.386772 | FUS | 16 |
| ENSG00000247596 | 252.3679 | 177.6888 | 0.443182 | 0.034072 | 0.387425 | TWF2 | 3 |
| ENSG00000106066 | 249.8967 | 330.3141 | -0.36673 | 0.034381 | 0.388377 | CPVL | 7 |
| ENSG00000117335 | 133.9806 | 188.8878 | -0.43352 | 0.034382 | 0.388377 | CD46 | 1 |
| ENSG00000246228 | 14.63929 | 4.293251 | 0.633028 | 0.034552 | 1 | CASC8 | 8 |
| ENSG00000009307 | 561.5674 | 422.5248 | 0.374664 | 0.034619 | 0.38966 | CSDE1 | 1 |
| ENSG00000134371 | 238.3594 | 164.766 | 0.454932 | 0.034722 | 0.38966 | CDC73 | 1 |
| ENSG00000115525 | 7.087495 | 18.97916 | -0.65255 | 0.034799 | 1 | ST3GAL5 | 2 |
| ENSG00000141367 | 928.8208 | 670.7298 | 0.417221 | 0.034965 | 0.391107 | CLTC | 17 |
| ENSG00000056558 | 21.6691 | 7.82644 | 0.650226 | 0.035212 | 1 | TRAF1 | 9 |
| ENSG00000181045 | 21.95496 | 40.67253 | -0.60622 | 0.035432 | 1 | SLC26A11 | 17 |
| ENSG00000244722 | 7.201342 | 0.619795 | 0.519663 | 0.035473 | 1 | RPSAP29 | 3 |
| ENSG00000170540 | 3.454166 | 13.03844 | -0.61647 | 0.035736 | 1 | ARL6IP1 | 16 |
| ENSG00000010704 | 34.10313 | 58.72345 | -0.57218 | 0.035745 | 1 | HFE | 6 |
| ENSG00000197345 | 179.7294 | 120.0899 | 0.482757 | 0.035749 | 0.397632 | MRPL21 | 11 |
| ENSG00000140848 | 33.54463 | 57.594 | -0.57547 | 0.035761 | 1 | CPNE2 | 16 |
| ENSG00000105193 | 1133.583 | 848.3886 | 0.379351 | 0.035833 | 0.397632 | RPS16 | 19 |
| ENSG00000107263 | 25.42003 | 46.6763 | -0.60013 | 0.035842 | 1 | RAPGEF1 | 9 |
| ENSG00000108639 | 181.4624 | 258.5299 | -0.44281 | 0.036032 | 0.397632 | SYNGR2 | 17 |
| ENSG00000111581 | 89.94982 | 57.39247 | 0.517767 | 0.036042 | 0.397632 | NUP107 | 12 |
| ENSG00000076003 | 216.2183 | 153.7421 | 0.431703 | 0.036127 | 0.397632 | MCM6 | 2 |
| ENSG00000249346 | 6.668463 | 19.00639 | -0.64415 | 0.036244 | 1 | LINC01016 | 6 |
| ENSG00000167105 | 21.06812 | 40.08444 | -0.61692 | 0.03643 | 1 | TMEM92 | 17 |
| ENSG00000165046 | 44.56211 | 21.36985 | 0.633141 | 0.036441 | 1 | LETM2 | 8 |
| ENSG00000084754 | 144.6675 | 101.8116 | 0.438779 | 0.036522 | 0.399742 | HADHA | 2 |
| ENSG00000222414 | 340.4033 | 219.7033 | 0.512956 | 0.036552 | 0.399742 | RNU2-59P | 10 |
| ENSG00000233830 | 7.194257 | 0.681953 | 0.518856 | 0.036964 | 1 | EIF4HP1 | 7 |
| ENSG00000104356 | 19.5823 | 7.045055 | 0.642878 | 0.037 | 1 | POP1 | 8 |
| ENSG00000155254 | 2.418401 | 10.92954 | -0.58588 | 0.037067 | 1 | MARVELD1 | 10 |
| ENSG00000252343 | 9.18575 | 1.376627 | 0.550975 | 0.037289 | 1 | RNU2-34P | 4 |
| ENSG00000204592 | 347.8383 | 449.7323 | -0.34095 | 0.037337 | 0.407037 | HLA-E | 6 |
| ENSG00000119547 | 23.03726 | 42.83555 | -0.6043 | 0.037507 | 1 | ONECUT2 | 18 |
| ENSG00000179335 | 42.46306 | 21.69748 | 0.620322 | 0.037673 | 1 | CLK3 | 15 |
| ENSG00000161013 | 121.428 | 174.5732 | -0.44638 | 0.037714 | 0.408171 | MGAT4B | 5 |
| ENSG00000120324 | 1.70612 | 8.509283 | -0.57257 | 0.037752 | 1 | PCDHB10 | 5 |
| ENSG00000165280 | 331.6929 | 229.2353 | 0.457561 | 0.037798 | 0.408171 | VCP | 9 |
| ENSG00000122566 | 1445.537 | 1152.67 | 0.307058 | 0.037849 | 0.408171 | HNRNPA2B1 | 7 |
| ENSG00000149177 | 125.6627 | 177.3051 | -0.42828 | 0.037917 | 0.408171 | PTPRJ | 11 |
| ENSG00000120053 | 87.11669 | 53.21941 | 0.539804 | 0.038089 | 1 | GOT1 | 10 |
| ENSG00000145623 | 454.5345 | 599.7677 | -0.36311 | 0.038114 | 0.409011 | OSMR | 5 |
| ENSG00000137692 | 180.4749 | 128.2116 | 0.42897 | 0.038643 | 0.413399 | DCUN1D5 | 11 |
| ENSG00000103061 | 66.9135 | 37.9465 | 0.581907 | 0.038811 | 1 | SLC7A6OS | 16 |
| ENSG00000116793 | 16.45894 | 36.14693 | -0.6338 | 0.038876 | 1 | PHTF1 | 1 |
| ENSG00000143367 | 33.23951 | 13.51886 | 0.640054 | 0.038924 | 1 | TUFT1 | 1 |
| ENSG00000096092 | 326.2415 | 227.6109 | 0.444445 | 0.039007 | 0.415992 | TMEM14A | 6 |
| ENSG00000169299 | 86.56665 | 51.29395 | 0.561672 | 0.03901 | 1 | PGM2 | 4 |
| ENSG00000123352 | 16.62744 | 33.76451 | -0.62425 | 0.039025 | 1 | SPATS2 | 12 |
| ENSG00000181350 | 1.86977 | 10.16232 | -0.55961 | 0.039155 | 1 | LRRC75A | 17 |
| ENSG00000145216 | 68.72552 | 41.81503 | 0.545739 | 0.039301 | 1 | FIP1L1 | 4 |
| ENSG00000166788 | 176.6811 | 125.1139 | 0.428793 | 0.039309 | 0.416684 | SAAL1 | 11 |
| ENSG00000173064 | 52.39126 | 30.31981 | 0.575015 | 0.039315 | 1 | HECTD4 | 12 |
| ENSG00000180667 | 2.783812 | 10.98846 | -0.59453 | 0.039376 | 1 | YOD1 | 1 |
| ENSG00000274020 | 14.40729 | 30.90378 | -0.63066 | 0.039412 | 1 | LINC01138 | 1 |
| ENSG00000145685 | 10.16837 | 23.15848 | -0.63658 | 0.039427 | 1 | LHFPL2 | 5 |
| ENSG00000086758 | 652.5781 | 512.4766 | 0.32544 | 0.039464 | 0.416684 | HUWE1 | X |
| ENSG00000139289 | 85.12496 | 128.2924 | -0.48976 | 0.039649 | 0.416684 | PHLDA1 | 12 |
| ENSG00000127184 | 109.504 | 171.2493 | -0.51619 | 0.0397 | 0.416684 | COX7C | 5 |
| ENSG00000253729 | 738.2788 | 586.0529 | 0.312229 | 0.039724 | 0.416684 | PRKDC | 8 |
| ENSG00000171298 | 170.2417 | 233.5779 | -0.4043 | 0.0398 | 0.416684 | GAA | 17 |
| ENSG00000166851 | 115.7434 | 77.70598 | 0.474321 | 0.039992 | 0.417096 | PLK1 | 16 |
| ENSG00000164692 | 58.21598 | 26.27931 | 0.632883 | 0.040167 | 1 | COL1A2 | 7 |
| ENSG00000183087 | 84.83886 | 124.2646 | -0.46496 | 0.040175 | 0.417096 | GAS6 | 13 |
| ENSG00000165733 | 92.67565 | 58.98513 | 0.519198 | 0.040203 | 0.417096 | BMS1 | 10 |
| ENSG00000116120 | 76.92541 | 46.86277 | 0.542196 | 0.040232 | 1 | FARSB | 2 |
| ENSG00000226179 | 15.53097 | 34.22024 | -0.63134 | 0.040248 | 1 | LINC00685 | X |
| ENSG00000196110 | 8.964901 | 1.885719 | 0.572623 | 0.040357 | 1 | ZNF699 | 19 |
| ENSG00000154153 | 13.78237 | 29.73116 | -0.62918 | 0.040399 | 1 | RETREG1 | 5 |
| ENSG00000015532 | 29.8498 | 51.51535 | -0.56765 | 0.040432 | 1 | XYLT2 | 17 |
| ENSG00000169967 | 15.64427 | 31.75537 | -0.61806 | 0.040499 | 1 | MAP3K2 | 2 |
| ENSG00000131018 | 95.20439 | 61.95333 | 0.497838 | 0.040548 | 0.418851 | SYNE1 | 6 |
| ENSG00000170043 | 368.7739 | 514.3738 | -0.42166 | 0.040617 | 0.418851 | TRAPPC1 | 17 |
| ENSG00000126216 | 74.12186 | 109.3145 | -0.46994 | 0.040974 | 0.419732 | TUBGCP3 | 13 |
| ENSG00000149136 | 306.8801 | 223.1443 | 0.407621 | 0.041249 | 0.419732 | SSRP1 | 11 |
| ENSG00000080839 | 164.8739 | 117.2833 | 0.429007 | 0.04132 | 0.419732 | RBL1 | 20 |
| ENSG00000207628 | 4.349428 | 14.64775 | -0.60436 | 0.041348 | 1 | MIR651 | X |
| ENSG00000184371 | 449.8339 | 577.9124 | -0.33258 | 0.041363 | 0.419732 | CSF1 | 1 |
| ENSG00000197283 | 2.924865 | 11.27292 | -0.58783 | 0.041395 | 1 | SYNGAP1 | 6 |
| ENSG00000207744 | 539.1468 | 234.0172 | 0.631184 | 0.041415 | 0.419732 | MIR10B | 2 |
| ENSG00000179409 | 277.9291 | 209.4724 | 0.367838 | 0.041578 | 0.419732 | GEMIN4 | 17 |
| ENSG00000196365 | 215.1593 | 152.9403 | 0.43068 | 0.041581 | 0.419732 | LONP1 | 19 |
| ENSG00000243317 | 68.20433 | 41.64039 | 0.538131 | 0.041659 | 1 | STMP1 | 7 |
| ENSG00000130255 | 839.6142 | 656.6679 | 0.328618 | 0.04168 | 0.419732 | RPL36 | 19 |
| ENSG00000265992 | 0 | 5.294593 | -0.4215 | 0.041805 | 1 | ESRG | 3 |
| ENSG00000159167 | 20.68338 | 40.29042 | -0.60741 | 0.041977 | 1 | STC1 | 8 |
| ENSG00000133318 | 1151.572 | 902.5058 | 0.32685 | 0.042046 | 0.422178 | RTN3 | 11 |
| ENSG00000054267 | 48.73478 | 27.52516 | 0.578624 | 0.042089 | 1 | ARID4B | 1 |
| ENSG00000127603 | 224.9374 | 156.5668 | 0.447728 | 0.042393 | 0.424428 | MACF1 | 1 |
| ENSG00000092621 | 36.04148 | 18.76725 | 0.603346 | 0.042489 | 1 | PHGDH | 1 |
| ENSG00000121879 | 71.10223 | 43.86949 | 0.528595 | 0.042512 | 1 | PIK3CA | 3 |
| ENSG00000234498 | 0.335177 | 5.419798 | -0.45909 | 0.042673 | 1 | RPL13AP20 | 12 |
| ENSG00000158470 | 126.0226 | 173.6159 | -0.40896 | 0.042731 | 0.424535 | B4GALT5 | 20 |
| ENSG00000168078 | 126.0612 | 83.85736 | 0.484103 | 0.042789 | 0.424535 | PBK | 8 |
| ENSG00000142173 | 70.66745 | 108.0624 | -0.49154 | 0.042817 | 0.424535 | COL6A2 | 21 |
| ENSG00000100353 | 182.0661 | 130.5073 | 0.420389 | 0.042899 | 0.424535 | EIF3D | 22 |
| ENSG00000205426 | 33.48344 | 15.96367 | 0.617813 | 0.042985 | 1 | KRT81 | 12 |
| ENSG00000105613 | 4.699327 | 14.36474 | -0.6131 | 0.043081 | 1 | MAST1 | 19 |
| ENSG00000127914 | 79.64748 | 49.78744 | 0.523319 | 0.043094 | 1 | AKAP9 | 7 |
| ENSG00000169251 | 103.7273 | 70.19411 | 0.468878 | 0.043161 | 0.425908 | NMD3 | 3 |
| ENSG00000137936 | 233.3886 | 166.0634 | 0.428231 | 0.043385 | 0.426754 | BCAR3 | 1 |
| ENSG00000115484 | 304.6413 | 226.4643 | 0.384741 | 0.043496 | 0.426754 | CCT4 | 2 |
| ENSG00000132326 | 5.011907 | 0 | 0.431364 | 0.043516 | 1 | PER2 | 2 |
| ENSG00000132970 | 1.165364 | 7.366785 | -0.5205 | 0.043602 | 1 | WASF3 | 13 |
| ENSG00000078401 | 22.59563 | 9.314661 | 0.625294 | 0.043647 | 1 | EDN1 | 6 |
| ENSG00000147894 | 19.50959 | 37.70413 | -0.60148 | 0.043727 | 1 | C9orf72 | 9 |
| ENSG00000113583 | 55.15949 | 86.58306 | -0.51013 | 0.043882 | 1 | C5orf15 | 5 |
| ENSG00000251257 | 5.469048 | 17.43436 | -0.60393 | 0.044014 | 1 | AC010457.1 | 5 |
| ENSG00000239183 | 33.24708 | 67.51716 | -0.61053 | 0.044025 | 1 | SNORA84 | 9 |
| ENSG00000156671 | 104.1881 | 148.6166 | -0.43977 | 0.044077 | 0.429274 | SAMD8 | 10 |
| ENSG00000169504 | 1300.236 | 930.0614 | 0.420573 | 0.044115 | 0.429274 | CLIC4 | 1 |
| ENSG00000198892 | 3.461802 | 12.22179 | -0.59279 | 0.044176 | 1 | SHISA4 | 1 |
| ENSG00000073464 | 67.68291 | 41.59273 | 0.533121 | 0.044212 | 1 | CLCN4 | X |
| ENSG00000005022 | 321.1368 | 239.976 | 0.37814 | 0.044256 | 0.429274 | SLC25A5 | X |
| ENSG00000113810 | 104.6139 | 68.10851 | 0.502351 | 0.044422 | 0.429274 | SMC4 | 3 |
| ENSG00000109805 | 107.5889 | 73.54406 | 0.460412 | 0.044451 | 0.429274 | NCAPG | 4 |
| ENSG00000131389 | 137.1272 | 192.7436 | -0.42231 | 0.044562 | 0.429274 | SLC6A6 | 3 |
| ENSG00000145730 | 207.1925 | 271.7778 | -0.35534 | 0.044674 | 0.429274 | PAM | 5 |
| ENSG00000023734 | 370.3803 | 286.7862 | 0.337785 | 0.044753 | 0.429274 | STRAP | 12 |
| ENSG00000094804 | 79.78338 | 48.96218 | 0.536944 | 0.044794 | 1 | CDC6 | 17 |
| ENSG00000109062 | 53.54572 | 83.3454 | -0.50933 | 0.045089 | 1 | SLC9A3R1 | 17 |
| ENSG00000111639 | 532.3329 | 408.0493 | 0.349704 | 0.04536 | 0.432292 | MRPL51 | 12 |
| ENSG00000225206 | 4.832744 | 0 | 0.425179 | 0.045499 | 1 | MIR137HG | 1 |
| ENSG00000070061 | 115.4385 | 80.21617 | 0.444269 | 0.045529 | 0.432292 | ELP1 | 9 |
| ENSG00000138442 | 183.8613 | 128.0448 | 0.443702 | 0.045553 | 0.432292 | WDR12 | 2 |
| ENSG00000207349 | 235.0109 | 341.4188 | -0.45094 | 0.045571 | 0.432292 | RNVU1-17 | 1 |
| ENSG00000164181 | 26.20908 | 47.04245 | -0.57911 | 0.045626 | 1 | ELOVL7 | 5 |
| ENSG00000066629 | 9.825321 | 23.07645 | -0.61912 | 0.04565 | 1 | EML1 | 14 |
| ENSG00000102144 | 491.9265 | 381.2289 | 0.338017 | 0.045808 | 0.433347 | PGK1 | X |
| ENSG00000092203 | 80.2085 | 45.74538 | 0.565041 | 0.045927 | 1 | TOX4 | 14 |
| ENSG00000238344 | 4.27266 | 14.52666 | -0.5921 | 0.045963 | 1 | SNORD126 | 14 |
| ENSG00000144354 | 44.13943 | 24.08583 | 0.578628 | 0.046146 | 1 | CDCA7 | 2 |
| ENSG00000198121 | 299.5218 | 388.0756 | -0.34475 | 0.046297 | 0.436771 | LPAR1 | 9 |
| ENSG00000074181 | 33.2618 | 58.55616 | -0.56464 | 0.046341 | 1 | NOTCH3 | 19 |
| ENSG00000013588 | 372.8543 | 489.228 | -0.35709 | 0.04659 | 0.438326 | GPRC5A | 12 |
| ENSG00000158863 | 12.63173 | 27.96943 | -0.61445 | 0.046609 | 1 | FAM160B2 | 8 |
| ENSG00000184500 | 332.8757 | 438.1727 | -0.35891 | 0.046888 | 0.439925 | PROS1 | 3 |
| ENSG00000105376 | 50.79541 | 81.2215 | -0.51586 | 0.046923 | 1 | ICAM5 | 19 |
| ENSG00000189091 | 282.9843 | 193.0408 | 0.46127 | 0.047016 | 0.439925 | SF3B3 | 16 |
| ENSG00000106462 | 41.80736 | 22.91163 | 0.581228 | 0.047261 | 1 | EZH2 | 7 |
| ENSG00000186184 | 60.34736 | 36.53461 | 0.540136 | 0.047524 | 1 | POLR1D | 13 |
| ENSG00000207595 | 10.22939 | 24.10736 | -0.61321 | 0.047874 | 1 | MIR181A2 | 9 |
| ENSG00000187535 | 20.38934 | 8.359525 | 0.612782 | 0.047948 | 1 | IFT140 | 16 |
| ENSG00000207927 | 11.84808 | 91.31679 | -0.48629 | 0.047992 | 1 | MIR302A | 4 |
| ENSG00000101210 | 129.9054 | 178.0929 | -0.39989 | 0.048162 | 0.449422 | EEF1A2 | 20 |
| ENSG00000142910 | 84.69478 | 123.1166 | -0.45601 | 0.048353 | 0.449986 | TINAGL1 | 1 |
| ENSG00000207705 | 0 | 5.598229 | -0.40071 | 0.048447 | 1 | MIR129-1 | 7 |
| ENSG00000233588 | 5.312792 | 0 | 0.414343 | 0.048459 | 1 | CYP51A1P2 | 13 |
| ENSG00000238363 | 46.6078 | 86.2937 | -0.58231 | 0.048568 | 1 | SNORA13 | 5 |
| ENSG00000163683 | 36.11746 | 60.05008 | -0.53548 | 0.048577 | 1 | SMIM14 | 4 |
| ENSG00000172366 | 5.305157 | 14.93276 | -0.60018 | 0.048665 | 1 | MCRIP2 | 16 |
| ENSG00000154727 | 21.59742 | 9.178515 | 0.610907 | 0.048715 | 1 | GABPA | 21 |
| ENSG00000111666 | 51.06162 | 81.89086 | -0.51858 | 0.048911 | 1 | CHPT1 | 12 |
| ENSG00000137434 | 53.49615 | 32.27718 | 0.537635 | 0.049026 | 1 | C6orf52 | 6 |
| ENSG00000196352 | 528.6087 | 665.456 | -0.31002 | 0.049084 | 0.455551 | CD55 | 1 |
| ENSG00000153930 | 7.864061 | 1.301748 | 0.519183 | 0.049125 | 1 | ANKFN1 | 17 |
| ENSG00000100410 | 269.3188 | 191.7682 | 0.420448 | 0.049319 | 0.456502 | PHF5A | 22 |
| ENSG00000134516 | 21.65056 | 8.681254 | 0.608481 | 0.049437 | 1 | DOCK2 | 5 |
| ENSG00000019549 | 1.748047 | 8.570551 | -0.52763 | 0.049685 | 1 | SNAI2 | 8 |
| ENSG00000119048 | 55.50691 | 85.14675 | -0.49435 | 0.049751 | 1 | UBE2B | 5 |
| ENSG00000213639 | 446.2247 | 349.3853 | 0.32618 | 0.049803 | 0.459743 | PPP1CB | 2 |

| **Table S7. TE of differentially expressed genes by FIBCD1** | | | | | | |
| --- | --- | --- | --- | --- | --- | --- |
| **Gene_id** | **TEEV_TE** | **TE-FIBCD1_TE** | **log2 Fold Change** | **P-value** | **P-adjusted** | **Gene_name** |
| ENSG00000165406 | 0.03820013 | 0.019026666 | -1.005554838 | 0.02439809 | 0.40599313 | 8-Mar |
| ENSG00000094914 | 0.08687275 | 0.138616389 | 0.674122154 | 0.021911184 | 0.400562807 | AAAS |
| ENSG00000275700 | 0.07705298 | 0.118479324 | 0.620712695 | 0.018531773 | 0.376403414 | AATF |
| ENSG00000154265 | 0.08333968 | 0.177764248 | 1.092889703 | 0.045586318 | 0.504879453 | ABCA5 |
| ENSG00000091262 | 0.00381607 | 0.040854444 | 3.420333241 | 0.016580077 | 0.36320919 | ABCC6 |
| ENSG00000069431 | 5.16730387 | 0.658911348 | -2.971255447 | 0.020843371 | 0.395798212 | ABCC9 |
| ENSG00000163686 | 0.12808868 | 0.079356573 | -0.690721331 | 0.024720962 | 0.408332514 | ABHD6 |
| ENSG00000213683 | 0.01874294 | 0.329827479 | 4.137292178 | 0.000770059 | 0.111447705 | AC002056.1 |
| ENSG00000089009 | 0.00793509 | 0.012543715 | 0.66064576 | 0.00960455 | 0.30080542 | AC004086.1 |
| ENSG00000262227 | 1.8110848 | 0.047397946 | -5.255885733 | 0.002219208 | 0.168618164 | AC004771.3 |
| ENSG00000224046 | 0.40475052 | 0.028054154 | -3.850746595 | 0.007676635 | 0.27610902 | AC005076.1 |
| ENSG00000131845 | 0.06964453 | 0.02746953 | -1.342177906 | 0.011280808 | 0.321126116 | AC005261.1 |
| ENSG00000272121 | 0.00989545 | 0.072990351 | 2.882868287 | 0.014395618 | 0.34780523 | AC006058.3 |
| ENSG00000232445 | 0.03634595 | 0.264833771 | 2.865220395 | 0.035320959 | 0.469376604 | AC006329.1 |
| ENSG00000242951 | 0.02445412 | 0.144655321 | 2.564470117 | 0.028202253 | 0.426092576 | AC007182.2 |
| ENSG00000233937 | 0.08517392 | 0.23067148 | 1.437355931 | 0.010328995 | 0.310048366 | AC008443.1 |
| ENSG00000237440 | 0.00722027 | 0.102081853 | 3.821529488 | 0.00376434 | 0.206140023 | AC008554.1 |
| ENSG00000257027 | 0.01207613 | 0.040065186 | 1.730190584 | 0.002500576 | 0.180949455 | AC010186.3 |
| ENSG00000261542 | 5.00185151 | 0.798960968 | -2.646265299 | 0.009040234 | 0.296533481 | AC011978.2 |
| ENSG00000261777 | 0.07689772 | 0.267546179 | 1.798775128 | 0.005751895 | 0.240287771 | AC012184.3 |
| ENSG00000272994 | 0.13307765 | 0.013801815 | -3.269338438 | 0.011469174 | 0.323073567 | AC012360.3 |
| ENSG00000254777 | 0.73183646 | 0.07842999 | -3.222043959 | 0.035032971 | 0.469376604 | AC022182.1 |
| ENSG00000279088 | 0.0325495 | 0.098943589 | 1.603970964 | 0.014105878 | 0.345040551 | AC022400.8 |
| ENSG00000274021 | 0.44251376 | 1.913179166 | 2.112177761 | 0.018538488 | 0.376403414 | AC024909.1 |
| ENSG00000267390 | 0.19768473 | 0.047225864 | -2.065552347 | 0.000491848 | 0.090596883 | AC036176.1 |
| ENSG00000279259 | 0.47196818 | 1.532087523 | 1.698737199 | 0.009977154 | 0.302474461 | AC087741.3 |
| ENSG00000198039 | 0.05989702 | 0.022327379 | -1.423670227 | 0.039642728 | 0.488356916 | AC092161.1 |
| ENSG00000267309 | 0.10878479 | 0.017604519 | -2.627459168 | 0.00752005 | 0.274481884 | AC092295.2 |
| ENSG00000240859 | 0.09887985 | 0.027521413 | -1.845122064 | 0.000956119 | 0.119062573 | AC093627.4 |
| ENSG00000242474 | 0.02989827 | 0.308290902 | 3.366158345 | 0.003194949 | 0.199184608 | AC093627.5 |
| ENSG00000271943 | 22.4991395 | 2.474004727 | -3.184949665 | 0.0220987 | 0.401177406 | AC098614.3 |
| ENSG00000255224 | 0.05554694 | 0.167787638 | 1.594856967 | 0.027953445 | 0.425333513 | AC109322.1 |
| ENSG00000279602 | 1.80641498 | 5.425340721 | 1.58658439 | 0.00651249 | 0.257053071 | AC109326.1 |
| ENSG00000273987 | 2.34434544 | 0.34839461 | -2.750390957 | 0.042211723 | 0.49919058 | AC121761.2 |
| ENSG00000275494 | 0.020999 | 0.126774968 | 2.59387762 | 0.006920149 | 0.266676147 | AC133552.5 |
| ENSG00000111271 | 0.04949626 | 0.092214885 | 0.897680162 | 0.03538024 | 0.469376604 | ACAD10 |
| ENSG00000213763 | 0.11700643 | 0.037609306 | -1.637426229 | 0.011445004 | 0.323073567 | ACTBP2 |
| ENSG00000130402 | 0.07153014 | 0.101126972 | 0.499544768 | 0.039428286 | 0.488356916 | ACTN4 |
| ENSG00000135503 | 0.09572895 | 0.059528837 | -0.685366594 | 0.02991027 | 0.438447748 | ACVR1B |
| ENSG00000119640 | 0.13883704 | 0.236337297 | 0.767454852 | 0.030475848 | 0.441591044 | ACYP1 |
| ENSG00000143382 | 0.11780772 | 0.065321136 | -0.850812342 | 0.023626547 | 0.405084178 | ADAMTSL4 |
| ENSG00000197381 | 0.01623825 | 0.037045405 | 1.18989806 | 0.023559993 | 0.405084178 | ADARB1 |
| ENSG00000065457 | 0.05378682 | 0.022489815 | -1.25798093 | 0.002405901 | 0.179131877 | ADAT1 |
| ENSG00000213638 | 0.02945431 | 0.077996803 | 1.404936367 | 0.039501191 | 0.488356916 | ADAT3 |
| ENSG00000063761 | 0.10100078 | 0.036984539 | -1.449372226 | 0.045965764 | 0.505789031 | ADCK1 |
| ENSG00000153292 | 0.37992863 | 0.069976464 | -2.440786782 | 0.040728278 | 0.490792918 | ADGRF1 |
| ENSG00000144843 | 0.1635033 | 0.01748004 | -3.225539349 | 0.018424319 | 0.376403414 | ADPRH |
| ENSG00000106624 | 0.30209964 | 3.712417636 | 3.619262635 | 0.001587018 | 0.142153335 | AEBP1 |
| ENSG00000156709 | 0.07914386 | 0.121299606 | 0.616025478 | 0.032394006 | 0.454225984 | AIFM1 |
| ENSG00000106305 | 0.0532874 | 0.095162765 | 0.836602651 | 0.003251133 | 0.200979115 | AIMP2 |
| ENSG00000127914 | 0.22788421 | 0.385290517 | 0.757645687 | 0.048869009 | 0.513607879 | AKAP9 |
| ENSG00000229771 | 0.05060175 | 0.007053122 | -2.842853522 | 0.022599624 | 0.40226007 | AL035665.1 |
| ENSG00000260464 | 9.10505898 | 1.766078275 | -2.366119077 | 0.008972443 | 0.296533481 | AL049796.1 |
| ENSG00000281091 | 0.45465142 | 0.057158553 | -2.991719538 | 0.032205654 | 0.452629065 | AL117327.1 |
| ENSG00000260708 | 0.09395043 | 0.234379495 | 1.318874722 | 0.023211577 | 0.405084178 | AL118516.1 |
| ENSG00000258457 | 2.53374062 | 0.463094848 | -2.451889232 | 0.014054406 | 0.345040551 | AL132780.2 |
| ENSG00000228436 | 0.0765399 | 0.521392934 | 2.768087165 | 0.008942312 | 0.296533481 | AL139260.1 |
| ENSG00000246203 | 0.02971607 | 0.18739772 | 2.656788329 | 0.005626216 | 0.239720683 | AL353807.3 |
| ENSG00000276317 | 0.21467351 | 0.05900291 | -1.86328615 | 0.013238485 | 0.340494851 | AL357033.3 |
| ENSG00000161618 | 0.0275154 | 0.044235682 | 0.684971415 | 0.041878147 | 0.498641163 | ALDH16A1 |
| ENSG00000164904 | 0.09072212 | 0.135575263 | 0.579567636 | 0.006399423 | 0.255615174 | ALDH7A1 |
| ENSG00000189046 | 0.05966655 | 0.099461843 | 0.737220843 | 0.04601366 | 0.505789031 | ALKBH2 |
| ENSG00000123505 | 0.02054397 | 0.037119529 | 0.853463168 | 0.033873782 | 0.462181339 | AMD1 |
| ENSG00000139344 | 0.07308291 | 0.021936806 | -1.736180533 | 0.045667851 | 0.504879453 | AMDHD1 |
| ENSG00000144233 | 0.02147348 | 0.038038499 | 0.82490427 | 0.025590278 | 0.412873312 | AMMECR1L |
| ENSG00000029534 | 0.11758827 | 0.044713083 | -1.39497518 | 0.04434897 | 0.502426364 | ANK1 |
| ENSG00000153930 | 0.01447403 | 0.064502804 | 2.155894723 | 0.045398942 | 0.504879453 | ANKFN1 |
| ENSG00000107890 | 0.05468066 | 0.110871632 | 1.019787696 | 0.024445749 | 0.40599313 | ANKRD26 |
| ENSG00000136938 | 0.06441994 | 0.098908306 | 0.618584265 | 0.0054997 | 0.238025957 | ANP32B |
| ENSG00000135046 | 0.07005437 | 0.112147878 | 0.678855446 | 0.002399496 | 0.179131877 | ANXA1 |
| ENSG00000182718 | 0.06095861 | 0.086899784 | 0.511522569 | 0.016534061 | 0.36320919 | ANXA2 |
| ENSG00000138772 | 0.09514066 | 0.138531065 | 0.542075618 | 0.020234101 | 0.391697398 | ANXA3 |
| ENSG00000164111 | 0.15227258 | 0.199979556 | 0.393196295 | 0.044457906 | 0.502426364 | ANXA5 |
| ENSG00000254612 | 0.00416511 | 0.021850978 | 2.391270558 | 0.045627249 | 0.504879453 | AP001000.1 |
| ENSG00000196961 | 0.03970408 | 0.055947711 | 0.494791862 | 0.035324319 | 0.469376604 | AP2A1 |
| ENSG00000132842 | 0.06122317 | 0.116866567 | 0.932712546 | 0.006116822 | 0.249537582 | AP3B1 |
| ENSG00000254470 | 0.00934655 | 0.032076735 | 1.779021466 | 0.001193052 | 0.129499425 | AP5B1 |
| ENSG00000171388 | 0.00803673 | 0.088078789 | 3.454113622 | 0.005403562 | 0.237693156 | APLN |
| ENSG00000130203 | 0.32971141 | 0.022629756 | -3.864910917 | 0.013871194 | 0.344966687 | APOE |
| ENSG00000101199 | 0.03460661 | 0.05944154 | 0.78042385 | 0.017386894 | 0.368887387 | ARFGAP1 |
| ENSG00000164144 | 0.1160593 | 0.186980602 | 0.688026499 | 0.030020448 | 0.438447748 | ARFIP1 |
| ENSG00000141522 | 0.10867815 | 0.256744229 | 1.240269874 | 7.35E-05 | 0.040908317 | ARHGDIA |
| ENSG00000240771 | 0.04956578 | 0.123745335 | 1.31995785 | 0.026670871 | 0.416672417 | ARHGEF25 |
| ENSG00000054267 | 0.06245558 | 0.141396542 | 1.178844516 | 0.000663376 | 0.107195358 | ARID4B |
| ENSG00000130429 | 0.01839223 | 0.081306758 | 2.144278545 | 0.043982824 | 0.502426364 | ARPC1B |
| ENSG00000226284 | 0.01964844 | 0.329709377 | 4.068708141 | 0.000979149 | 0.119062573 | ARPC3P1 |
| ENSG00000241553 | 0.00045584 | 0.002846123 | 2.64240431 | 0.007914756 | 0.2807695 | ARPC4 |
| ENSG00000141480 | 0.05624824 | 0.09976001 | 0.826653754 | 0.028021334 | 0.425333513 | ARRB2 |
| ENSG00000204147 | 0.04942482 | 0.011063994 | -2.159363406 | 0.003633892 | 0.206140023 | ASAH2B |
| ENSG00000105011 | 0.10179757 | 0.177889159 | 0.805275527 | 0.036778005 | 0.477603236 | ASF1B |
| ENSG00000235919 | 0.05476314 | 0.196597948 | 1.843971175 | 0.0373353 | 0.479242828 | ASH1L-AS1 |
| ENSG00000070669 | 0.02817506 | 0.101840633 | 1.853822924 | 0.04549651 | 0.504879453 | ASNS |
| ENSG00000128203 | 0.03149815 | 0.121923218 | 1.952633985 | 0.006612354 | 0.258477138 | ASPHD2 |
| ENSG00000119778 | 0.01598039 | 0.056323678 | 1.817439265 | 0.039741799 | 0.488356916 | ATAD2B |
| ENSG00000138363 | 0.0697244 | 0.108795117 | 0.641878255 | 0.010776599 | 0.316409856 | ATIC |
| ENSG00000196296 | 0.04538965 | 0.243964551 | 2.426236217 | 0.034582702 | 0.468313295 | ATP2A1 |
| ENSG00000124172 | 0.11479053 | 0.071223719 | -0.688573891 | 0.043515853 | 0.502426364 | ATP5F1E |
| ENSG00000135390 | 0.05299621 | 0.092490443 | 0.803415029 | 0.002641487 | 0.183607934 | ATP5MC2 |
| ENSG00000154518 | 0.09449215 | 0.162111219 | 0.778717483 | 0.004251166 | 0.222554422 | ATP5MC3 |
| ENSG00000167863 | 0.13744893 | 0.233587506 | 0.765067392 | 0.009632246 | 0.30080542 | ATP5PD |
| ENSG00000155097 | 0.09840721 | 0.154356547 | 0.649430776 | 0.049436347 | 0.516495431 | ATP6V1C1 |
| ENSG00000047249 | 0.07074352 | 0.106507645 | 0.590287099 | 0.043705688 | 0.502426364 | ATP6V1H |
| ENSG00000143515 | 0.01890166 | 0.006903746 | -1.453061379 | 0.000362643 | 0.086444205 | ATP8B2 |
| ENSG00000127423 | 0.03289963 | 0.088831783 | 1.433004631 | 0.025807375 | 0.415000343 | AUNIP |
| ENSG00000175756 | 0.06824184 | 0.11384203 | 0.738304783 | 0.039743696 | 0.488356916 | AURKAIP1 |
| ENSG00000135407 | 0.06190204 | 0.358115885 | 2.532367721 | 0.015690506 | 0.358551664 | AVIL |
| ENSG00000237172 | 0.03773089 | 0.012474215 | -1.596796937 | 0.048884699 | 0.513607879 | B3GNT9 |
| ENSG00000175711 | 0.0532203 | 0.130816772 | 1.297498932 | 0.000908585 | 0.118770642 | B3GNTL1 |
| ENSG00000139044 | 0.08721092 | 0.022670923 | -1.943665628 | 0.023826855 | 0.405084178 | B4GALNT3 |
| ENSG00000123810 | 0.02486745 | 0.07145605 | 1.522797617 | 0.036934383 | 0.477671592 | B9D2 |
| ENSG00000247081 | 0.03945538 | 0.205086115 | 2.377936047 | 0.032059976 | 0.452149806 | BAALC-AS1 |
| ENSG00000112208 | 0.14793634 | 0.209332124 | 0.500817194 | 0.035955217 | 0.472038414 | BAG2 |
| ENSG00000095739 | 0.07251963 | 0.215079857 | 1.568429031 | 0.001908157 | 0.157805871 | BAMBI |
| ENSG00000175334 | 0.1262023 | 0.198650427 | 0.654493642 | 0.02744301 | 0.421530402 | BANF1 |
| ENSG00000181004 | 0.12831665 | 0.043872523 | -1.548318778 | 0.026494268 | 0.416672417 | BBS12 |
| ENSG00000141376 | 0.08368896 | 0.044560622 | -0.909268013 | 0.034430546 | 0.467678376 | BCAS3 |
| ENSG00000107949 | 0.14596418 | 0.269398519 | 0.884127507 | 0.007091901 | 0.267871649 | BCCIP |
| ENSG00000099968 | 0.04557741 | 0.02771647 | -0.717575559 | 0.036583326 | 0.477192592 | BCL2L13 |
| ENSG00000129473 | 0.00192135 | 0.006681445 | 1.798038024 | 0.000989168 | 0.119062573 | BCL2L2 |
| ENSG00000258643 | 0.63015889 | 8.644686329 | 3.778026074 | 0.014751235 | 0.35232888 | BCL2L2-PABPN1 |
| ENSG00000113916 | 0.06232718 | 0.116640837 | 0.904139617 | 0.017129905 | 0.366399131 | BCL6 |
| ENSG00000183337 | 0.07613894 | 0.13671836 | 0.844500531 | 0.00467284 | 0.230925678 | BCOR |
| ENSG00000164039 | 0.08521352 | 0.047196225 | -0.852410882 | 0.049542864 | 0.516591873 | BDH2 |
| ENSG00000123095 | 0.07871599 | 0.182405338 | 1.212419288 | 0.015554954 | 0.357071263 | BHLHE41 |
| ENSG00000197299 | 0.06017692 | 0.117171051 | 0.961334051 | 0.040167423 | 0.489418046 | BLM |
| ENSG00000104164 | 0.01358426 | 0.028305721 | 1.059157558 | 0.0262282 | 0.416672417 | BLOC1S6 |
| ENSG00000168487 | 0.14033169 | 0.087910619 | -0.674731538 | 0.047759869 | 0.510030853 | BMP1 |
| ENSG00000165733 | 0.05041828 | 0.09145938 | 0.85918414 | 0.003020376 | 0.195312271 | BMS1 |
| ENSG00000166164 | 0.01699912 | 0.044061125 | 1.374046583 | 0.000311267 | 0.080669571 | BRD7 |
| ENSG00000113460 | 0.09839096 | 0.185123145 | 0.911887561 | 0.003843768 | 0.20861022 | BRIX1 |
| ENSG00000145741 | 0.14206125 | 0.209929401 | 0.563391184 | 0.022070268 | 0.401177406 | BTF3 |
| ENSG00000156970 | 0.05241988 | 0.129985781 | 1.310167843 | 0.001021064 | 0.119356439 | BUB1B |
| ENSG00000106245 | 0.13416673 | 0.217116027 | 0.694439275 | 0.021586012 | 0.397728112 | BUD31 |
| ENSG00000137720 | 0.01076902 | 0.032143369 | 1.577633734 | 0.008075473 | 0.282920839 | C11orf1 |
| ENSG00000174370 | 0.01179247 | 0.037920727 | 1.685121154 | 0.022751015 | 0.4036381 | C11orf45 |
| ENSG00000100802 | 0.09236965 | 0.227906793 | 1.302953064 | 0.010847262 | 0.316679518 | C14orf93 |
| ENSG00000125149 | 0.09379907 | 0.051130254 | -0.875396382 | 0.016471505 | 0.36320919 | C16orf70 |
| ENSG00000125319 | 0.0214838 | 0.047119258 | 1.133067862 | 0.033257007 | 0.46153588 | C17orf53 |
| ENSG00000228300 | 0.02471434 | 0.045385734 | 0.876890572 | 0.023728494 | 0.405084178 | C19orf24 |
| ENSG00000119559 | 0.0354408 | 0.016204273 | -1.129036984 | 0.0424845 | 0.49982388 | C19orf25 |
| ENSG00000104979 | 0.10199559 | 0.2314739 | 1.182342815 | 0.001596351 | 0.142153335 | C19orf53 |
| ENSG00000188493 | 0.09313113 | 0.047996126 | -0.956345457 | 0.007210413 | 0.268064193 | C19orf54 |
| ENSG00000108561 | 0.06601323 | 0.117555964 | 0.832520654 | 0.000289787 | 0.078287599 | C1QBP |
| ENSG00000131094 | 0.02015696 | 0.080835965 | 2.00371931 | 0.01319943 | 0.340494851 | C1QL1 |
| ENSG00000205208 | 0.05421906 | 0.099442221 | 0.875058383 | 0.046843397 | 0.507102816 | C4orf46 |
| ENSG00000172244 | 0.09426299 | 0.035370197 | -1.414157251 | 0.017493977 | 0.369395776 | C5orf34 |
| ENSG00000137434 | 0.10110132 | 0.183982211 | 0.863764378 | 0.012875312 | 0.338799064 | C6orf52 |
| ENSG00000149679 | 0.01309601 | 0.024717793 | 0.916423127 | 0.019027685 | 0.382205864 | CABLES2 |
| ENSG00000102001 | 0.13318125 | 1.448902662 | 3.443497762 | 0.033004587 | 0.458556296 | CACNA1F |
| ENSG00000182389 | 0.02635445 | 0.005161209 | -2.352265666 | 0.045398921 | 0.504879453 | CACNB4 |
| ENSG00000229097 | 0.05143997 | 0.315544934 | 2.616883788 | 0.021375942 | 0.397220313 | CALM2P2 |
| ENSG00000004660 | 0.01370296 | 0.051005926 | 1.89617779 | 0.013932602 | 0.344966687 | CAMKK1 |
| ENSG00000111530 | 0.09562436 | 0.146121143 | 0.6117149 | 0.026445738 | 0.416672417 | CAND1 |
| ENSG00000105483 | 0.07460009 | 0.118673492 | 0.669748463 | 0.038600551 | 0.484377942 | CARD8 |
| ENSG00000156017 | 0.03505851 | 0.088294025 | 1.332551045 | 0.007728424 | 0.27677828 | CARNMT1 |
| ENSG00000177640 | 0.14155602 | 0.032775266 | -2.110693676 | 0.021041035 | 0.395798212 | CASC2 |
| ENSG00000246228 | 0.03917504 | 0.11895362 | 1.602392364 | 0.007031215 | 0.267510526 | CASC8 |
| ENSG00000127995 | 0.04385225 | 0.016236536 | -1.433407132 | 0.030690669 | 0.442612992 | CASD1 |
| ENSG00000067955 | 0.06927889 | 0.114131861 | 0.720213946 | 0.019052131 | 0.382205864 | CBFB |
| ENSG00000159231 | 0.03048668 | 0.109121548 | 1.839684862 | 0.046112999 | 0.506097126 | CBR3 |
| ENSG00000100211 | 0.03792319 | 0.012327203 | -1.621234964 | 0.000710759 | 0.11131941 | CBY1 |
| ENSG00000160799 | 0.41292001 | 0.79835057 | 0.951160058 | 0.007041488 | 0.267510526 | CCDC12 |
| ENSG00000135637 | 0.01796406 | 0.060955493 | 1.762642814 | 0.00525298 | 0.235647504 | CCDC142 |
| ENSG00000153237 | 0.09243713 | 0.037584991 | -1.298315886 | 0.047912391 | 0.510030853 | CCDC148 |
| ENSG00000285825 | 0.14051261 | 0.73249785 | 2.382124879 | 0.002206927 | 0.168618164 | CCDC15-DT |
| ENSG00000236383 | 0.1451308 | 2.188496999 | 3.91451475 | 0.000449858 | 0.088208388 | CCDC200 |
| ENSG00000147419 | 0.09251429 | 0.158147862 | 0.773525861 | 0.025905524 | 0.415479489 | CCDC25 |
| ENSG00000109881 | 0.14017608 | 0.263466838 | 0.910381196 | 0.030662443 | 0.442612992 | CCDC34 |
| ENSG00000160124 | 0.10176255 | 0.202830624 | 0.995068733 | 0.008558476 | 0.291443681 | CCDC58 |
| ENSG00000104983 | 0.02174289 | 0.076544478 | 1.815754371 | 0.03717966 | 0.478806278 | CCDC61 |
| ENSG00000216937 | 0.16919881 | 0.052642871 | -1.684409351 | 0.047963438 | 0.510030853 | CCDC7 |
| ENSG00000186714 | 3.38971135 | 0.202571536 | -4.064659049 | 0.00496634 | 0.233222988 | CCDC73 |
| ENSG00000120647 | 0.05872361 | 0.127643002 | 1.120101887 | 0.006149034 | 0.250012715 | CCDC77 |
| ENSG00000186166 | 0.01692953 | 0.040509665 | 1.258724359 | 0.028874546 | 0.429654664 | CCDC84 |
| ENSG00000115009 | 0.6468007 | 1.65698896 | 1.357170838 | 0.013426506 | 0.342911825 | CCL20 |
| ENSG00000175305 | 0.15661623 | 0.416085529 | 1.409646431 | 0.044146731 | 0.502426364 | CCNE2 |
| ENSG00000107443 | 0.0213657 | 0.004311719 | -2.308961879 | 0.018983168 | 0.38208339 | CCNJ |
| ENSG00000260916 | 0.059608 | 0.012373566 | -2.268244611 | 0.005612397 | 0.239720683 | CCPG1 |
| ENSG00000166226 | 0.04474946 | 0.071172958 | 0.669458869 | 0.003635158 | 0.206140023 | CCT2 |
| ENSG00000115484 | 0.04339373 | 0.07051887 | 0.700522866 | 0.015596351 | 0.357071263 | CCT4 |
| ENSG00000150753 | 0.12439571 | 0.185637586 | 0.577552068 | 0.04887221 | 0.513607879 | CCT5 |
| ENSG00000132141 | 0.17195135 | 0.017553469 | -3.292172385 | 0.004265459 | 0.222554422 | CCT6B |
| ENSG00000156261 | 0.10404312 | 0.147110417 | 0.499717868 | 0.047692051 | 0.510030853 | CCT8 |
| ENSG00000101017 | 0.09906682 | 0.06523943 | -0.602657718 | 0.032904869 | 0.457693934 | CD40 |
| ENSG00000102543 | 0.05009638 | 0.143668625 | 1.519966647 | 0.000256104 | 0.072405882 | CDADC1 |
| ENSG00000105401 | 0.11630416 | 0.225207325 | 0.953351071 | 0.002460826 | 0.180218409 | CDC37 |
| ENSG00000094804 | 0.02479216 | 0.051198263 | 1.046210815 | 0.005005946 | 0.233222988 | CDC6 |
| ENSG00000134371 | 0.11688467 | 0.188789525 | 0.691692991 | 0.03686795 | 0.477603236 | CDC73 |
| ENSG00000144354 | 0.04276098 | 0.114133186 | 1.416351466 | 0.002415697 | 0.179131877 | CDCA7 |
| ENSG00000163814 | 0.20250902 | 0.141718186 | -0.514961243 | 0.039019179 | 0.487519178 | CDCP1 |
| ENSG00000062038 | 0.42941116 | 1.002389412 | 1.223011484 | 0.01380817 | 0.344966687 | CDH3 |
| ENSG00000101391 | 0.03717372 | 0.055010218 | 0.565416512 | 0.042121687 | 0.49919058 | CDK5RAP1 |
| ENSG00000132964 | 0.04754149 | 0.136303643 | 1.519565155 | 0.000585162 | 0.098802927 | CDK8 |
| ENSG00000138769 | 0.26902489 | 0.04256553 | -2.659982165 | 0.005805883 | 0.240894614 | CDKL2 |
| ENSG00000167513 | 0.01749775 | 0.031929743 | 0.867731677 | 0.025605909 | 0.412873312 | CDT1 |
| ENSG00000008300 | 0.00403239 | 0.014363047 | 1.83265299 | 0.007951167 | 0.2807695 | CELSR3 |
| ENSG00000138778 | 0.11432426 | 0.256871976 | 1.167917865 | 0.037087532 | 0.478418048 | CENPE |
| ENSG00000102384 | 0.02899654 | 0.095295604 | 1.716528955 | 0.024924683 | 0.409035682 | CENPI |
| ENSG00000166451 | 0.109615 | 0.177030601 | 0.691553525 | 0.040588174 | 0.490000424 | CENPN |
| ENSG00000159079 | 0.04430857 | 0.12644041 | 1.512799912 | 0.001185941 | 0.129499425 | CFAP298 |
| ENSG00000105792 | 0.02979692 | 0.262941535 | 3.141507013 | 0.011473628 | 0.323073567 | CFAP69 |
| ENSG00000153774 | 0.13097114 | 0.217692112 | 0.733040166 | 0.014629326 | 0.351479685 | CFDP1 |
| ENSG00000000971 | 0.69462827 | 0.333870715 | -1.056951564 | 0.043950094 | 0.502426364 | CFH |
| ENSG00000172757 | 0.11166712 | 0.169316482 | 0.600518024 | 0.031554351 | 0.448662268 | CFL1 |
| ENSG00000198824 | 0.05114309 | 0.08820225 | 0.78627607 | 0.028262005 | 0.426092576 | CHAMP1 |
| ENSG00000163528 | 0.04141507 | 0.025710477 | -0.687799315 | 0.046843503 | 0.507102816 | CHCHD4 |
| ENSG00000171316 | 0.20814935 | 0.133334223 | -0.642571899 | 0.049453046 | 0.516495431 | CHD7 |
| ENSG00000085872 | 0.02208569 | 0.04750413 | 1.104941187 | 0.003748753 | 0.206140023 | CHERP |
| ENSG00000204116 | 0.07195557 | 0.024956594 | -1.527685318 | 0.019287168 | 0.384370217 | CHIC1 |
| ENSG00000176108 | 0.03419817 | 0.015686006 | -1.124441158 | 0.017061714 | 0.366399131 | CHMP6 |
| ENSG00000110172 | 0.0617341 | 0.096735417 | 0.647976591 | 0.046760393 | 0.507102816 | CHORDC1 |
| ENSG00000111666 | 0.03350204 | 0.019647668 | -0.769890676 | 0.011899641 | 0.328781684 | CHPT1 |
| ENSG00000133019 | 0.11793245 | 0.027293499 | -2.11133141 | 0.031861216 | 0.450391633 | CHRM3 |
| ENSG00000104859 | 0.03044512 | 0.056187353 | 0.884034666 | 0.025309911 | 0.410383263 | CLASRP |
| ENSG00000114859 | 0.09893186 | 0.042430914 | -1.221319395 | 0.003664728 | 0.206140023 | CLCN2 |
| ENSG00000165215 | 0.13161463 | 0.038794105 | -1.762410524 | 0.025300846 | 0.410383263 | CLDN3 |
| ENSG00000160318 | 0.08251733 | 0.343978347 | 2.059548701 | 0.04053224 | 0.490000424 | CLDND2 |
| ENSG00000159212 | 0.0637529 | 0.104994092 | 0.719745296 | 0.03272799 | 0.456277724 | CLIC6 |
| ENSG00000113282 | 0.08087676 | 0.137318928 | 0.763733351 | 0.034918806 | 0.469376604 | CLINT1 |
| ENSG00000179335 | 0.02999896 | 0.064942927 | 1.114259811 | 0.0048023 | 0.232710694 | CLK3 |
| ENSG00000125656 | 0.02649669 | 0.043910639 | 0.728758153 | 0.029618203 | 0.43731045 | CLPP |
| ENSG00000122705 | 0.04987974 | 0.072763774 | 0.544766567 | 0.028208507 | 0.426092576 | CLTA |
| ENSG00000105427 | 0.29391711 | 0.134219967 | -1.13081004 | 0.001956545 | 0.160714304 | CNFN |
| ENSG00000088038 | 0.02768533 | 0.057862108 | 1.0634973 | 0.00125267 | 0.132423529 | CNOT3 |
| ENSG00000080802 | 0.04342237 | 0.085443713 | 0.976535814 | 0.01717821 | 0.366399131 | CNOT4 |
| ENSG00000166997 | 0.11211308 | 0.273521043 | 1.286697181 | 0.008230442 | 0.284628907 | CNPY4 |
| ENSG00000168542 | 0.05864984 | 0.487344916 | 3.054744224 | 0.043814934 | 0.502426364 | COL3A1 |
| ENSG00000142173 | 0.18659226 | 0.123313442 | -0.597559084 | 0.04826993 | 0.51161076 | COL6A2 |
| ENSG00000114270 | 0.01893466 | 0.098446123 | 2.378304514 | 0.020806653 | 0.395798212 | COL7A1 |
| ENSG00000144810 | 0.13115928 | 0.091013492 | -0.527167558 | 0.044275191 | 0.502426364 | COL8A1 |
| ENSG00000049089 | 0.0062989 | 0.033380915 | 2.405851092 | 0.017089349 | 0.366399131 | COL9A2 |
| ENSG00000148444 | 0.00216662 | 0.014827504 | 2.774759503 | 0.003522606 | 0.206140023 | COMMD3 |
| ENSG00000169019 | 0.05110934 | 0.090611093 | 0.826100765 | 0.041164914 | 0.493381361 | COMMD8 |
| ENSG00000005243 | 0.03221053 | 0.142923209 | 2.149636089 | 0.000423896 | 0.088208388 | COPZ2 |
| ENSG00000132423 | 0.10479083 | 0.19857324 | 0.922158698 | 0.042203991 | 0.49919058 | COQ3 |
| ENSG00000167113 | 0.04839787 | 0.071623003 | 0.565479421 | 0.045482522 | 0.504879453 | COQ4 |
| ENSG00000103647 | 0.06556351 | 0.026152833 | -1.325925925 | 0.01889453 | 0.381562785 | CORO2B |
| ENSG00000126267 | 0.13907105 | 0.238728914 | 0.779551173 | 0.036837039 | 0.477603236 | COX6B1 |
| ENSG00000106034 | 0.15422461 | 0.026790806 | -2.525223105 | 0.007450881 | 0.272832425 | CPED1 |
| ENSG00000140848 | 0.03574138 | 0.020025386 | -0.835765454 | 0.006790885 | 0.264605076 | CPNE2 |
| ENSG00000165934 | 0.09335376 | 0.148523368 | 0.669909951 | 0.033637442 | 0.462181339 | CPSF2 |
| ENSG00000149532 | 0.03024408 | 0.049194235 | 0.701836677 | 0.005341264 | 0.237693156 | CPSF7 |
| ENSG00000224051 | 0.03188713 | 0.011226166 | -1.506109202 | 0.002729167 | 0.187449063 | CPTP |
| ENSG00000177685 | 0.04909722 | 0.022800197 | -1.106594925 | 0.004118962 | 0.219623776 | CRACR2B |
| ENSG00000107175 | 0.04756494 | 0.071778151 | 0.593646063 | 0.047402958 | 0.510030853 | CREB3 |
| ENSG00000215908 | 0.01457694 | 0.004498682 | -1.696113888 | 0.04958976 | 0.516591873 | CROCCP2 |
| ENSG00000108342 | 0.0471757 | 0.107314715 | 1.185731976 | 0.003289409 | 0.201098109 | CSF3 |
| ENSG00000160213 | 0.1089752 | 0.170817725 | 0.648457832 | 0.021553023 | 0.397728112 | CSTB |
| ENSG00000177613 | 0.0839734 | 0.141564757 | 0.753457903 | 0.014088718 | 0.345040551 | CSTF2T |
| ENSG00000176102 | 0.24403184 | 0.390188593 | 0.67710221 | 0.044322332 | 0.502426364 | CSTF3 |
| ENSG00000164733 | 0.18494365 | 0.129508146 | -0.514042926 | 0.021125949 | 0.395798212 | CTSB |
| ENSG00000103811 | 0.11738374 | 0.076336861 | -0.62078078 | 0.02366826 | 0.405084178 | CTSH |
| ENSG00000055130 | 0.09406374 | 0.138512601 | 0.558306634 | 0.043451952 | 0.502426364 | CUL1 |
| ENSG00000226752 | 0.03360483 | 0.006565091 | -2.355781615 | 9.92E-05 | 0.044719976 | CUTALP |
| ENSG00000095485 | 0.02360462 | 0.052116981 | 1.142684255 | 0.002699741 | 0.186481542 | CWF19L1 |
| ENSG00000071967 | 0.10399612 | 0.068857466 | -0.59484476 | 0.047761727 | 0.510030853 | CYBRD1 |
| ENSG00000232973 | 0.48730677 | 5.592328616 | 3.520546957 | 0.002790825 | 0.188489189 | CYP1B1-AS1 |
| ENSG00000146233 | 0.10336906 | 0.296646361 | 1.52093972 | 0.02599074 | 0.415749251 | CYP39A1 |
| ENSG00000233588 | 0.24161902 | 1.346170134 | 2.478054838 | 0.0035367 | 0.206140023 | CYP51A1P2 |
| ENSG00000008256 | 0.02646029 | 0.013238911 | -0.999044655 | 0.007362579 | 0.272057364 | CYTH3 |
| ENSG00000153071 | 0.08359212 | 0.040076842 | -1.060598087 | 0.008982952 | 0.296533481 | DAB2 |
| ENSG00000115866 | 0.10578918 | 0.158342183 | 0.581853517 | 0.033503645 | 0.462181339 | DARS |
| ENSG00000003249 | 0.02947293 | 0.012955491 | -1.185827089 | 0.011785423 | 0.326852306 | DBNDD1 |
| ENSG00000164934 | 0.09354748 | 0.145378601 | 0.636044208 | 0.016734043 | 0.363928005 | DCAF13 |
| ENSG00000163257 | 0.03265996 | 0.055714291 | 0.770524619 | 0.02123004 | 0.395798212 | DCAF16 |
| ENSG00000150401 | 0.04149347 | 0.015261739 | -1.442964843 | 0.031963766 | 0.451316495 | DCUN1D2 |
| ENSG00000013573 | 0.01335807 | 0.034872289 | 1.384369677 | 0.011802925 | 0.326852306 | DDX11 |
| ENSG00000088205 | 0.02106117 | 0.041126818 | 0.965494208 | 0.01099883 | 0.319123576 | DDX18 |
| ENSG00000168872 | 0.1212239 | 0.042519964 | -1.51146194 | 0.005411651 | 0.237693156 | DDX19A |
| ENSG00000064703 | 0.0480131 | 0.082188654 | 0.775511041 | 0.028462718 | 0.428244138 | DDX20 |
| ENSG00000174243 | 0.01801014 | 0.031108993 | 0.788522529 | 0.027461716 | 0.421530402 | DDX23 |
| ENSG00000089737 | 0.02217237 | 0.042535276 | 0.939896686 | 0.004626955 | 0.230174477 | DDX24 |
| ENSG00000123136 | 0.09680057 | 0.164987967 | 0.769273384 | 0.01893904 | 0.381827385 | DDX39A |
| ENSG00000035499 | 0.03293585 | 0.077694934 | 1.238161825 | 0.012803859 | 0.337649696 | DEPDC1B |
| ENSG00000169598 | 0.09844644 | 0.036997696 | -1.41190362 | 0.009106243 | 0.296533481 | DFFB |
| ENSG00000070413 | 0.05549569 | 0.033037819 | -0.748257175 | 0.009131839 | 0.296533481 | DGCR2 |
| ENSG00000128191 | 0.05041471 | 0.094625272 | 0.908380868 | 0.009146998 | 0.296533481 | DGCR8 |
| ENSG00000109606 | 0.04131186 | 0.070221397 | 0.765354619 | 0.006391445 | 0.255615174 | DHX15 |
| ENSG00000135829 | 0.02019824 | 0.044798704 | 1.149227635 | 0.016647668 | 0.36351266 | DHX9 |
| ENSG00000101191 | 0.01855135 | 0.030709235 | 0.727148339 | 0.008715928 | 0.295094016 | DIDO1 |
| ENSG00000144535 | 0.04992397 | 0.09596486 | 0.942773477 | 0.032796331 | 0.456706755 | DIS3L2 |
| ENSG00000140323 | 0.08732781 | 0.043069027 | -1.019790382 | 0.043554766 | 0.502426364 | DISP2 |
| ENSG00000132535 | 0.03269451 | 0.01447682 | -1.175303842 | 0.042539034 | 0.49982388 | DLG4 |
| ENSG00000185800 | 0.00633042 | 0.013524607 | 1.095212813 | 0.045638024 | 0.504879453 | DMWD |
| ENSG00000183914 | 0.03324069 | 0.058903337 | 0.825399201 | 0.04667899 | 0.507102816 | DNAH2 |
| ENSG00000069345 | 0.08729083 | 0.129668477 | 0.570925783 | 0.019189297 | 0.384073661 | DNAJA2 |
| ENSG00000090520 | 0.21630107 | 0.371088291 | 0.778721653 | 0.002011077 | 0.162991121 | DNAJB11 |
| ENSG00000205981 | 0.01801862 | 0.009400739 | -0.938642462 | 0.034081183 | 0.463450714 | DNAJC19 |
| ENSG00000101152 | 0.01367928 | 0.008548371 | -0.678271252 | 0.00567101 | 0.239720683 | DNAJC5 |
| ENSG00000119661 | 0.03946026 | 0.072805323 | 0.88364351 | 0.028921027 | 0.429820201 | DNAL1 |
| ENSG00000119772 | 0.02151302 | 0.050441415 | 1.229398743 | 0.039698857 | 0.488356916 | DNMT3A |
| ENSG00000134516 | 0.06381502 | 0.157661785 | 1.304865027 | 0.008766754 | 0.295094016 | DOCK2 |
| ENSG00000136908 | 0.03501693 | 0.022316722 | -0.649927579 | 0.033556294 | 0.462181339 | DPM2 |
| ENSG00000254986 | 0.04605066 | 0.092782266 | 1.010627344 | 0.006894703 | 0.266676147 | DPP3 |
| ENSG00000175550 | 0.10282597 | 0.192376475 | 0.903727665 | 0.005501793 | 0.238025957 | DRAP1 |
| ENSG00000133059 | 0.01742496 | 0.036479623 | 1.065935862 | 0.015527457 | 0.357071263 | DSTYK |
| ENSG00000143476 | 0.07131159 | 0.112431116 | 0.656832905 | 0.042849605 | 0.500406003 | DTL |
| ENSG00000164086 | 0.02538107 | 0.014855941 | -0.772712917 | 0.024933101 | 0.409035682 | DUSP7 |
| ENSG00000127334 | 0.02346908 | 0.054800642 | 1.223431634 | 0.01830689 | 0.375940647 | DYRK2 |
| ENSG00000143479 | 0.03038619 | 0.054156301 | 0.833713453 | 0.03948779 | 0.488356916 | DYRK3 |
| ENSG00000133740 | 0.04003366 | 0.079653464 | 0.992523754 | 0.018172166 | 0.375712616 | E2F5 |
| ENSG00000117395 | 0.05416806 | 0.088504106 | 0.708302078 | 0.001306573 | 0.134907466 | EBNA1BP2 |
| ENSG00000122882 | 0.05877603 | 0.097874969 | 0.735712108 | 0.004969789 | 0.233222988 | ECD |
| ENSG00000179151 | 0.02303933 | 0.03954497 | 0.779395272 | 0.019266976 | 0.384370217 | EDC3 |
| ENSG00000156508 | 0.00369653 | 0.005730868 | 0.632582834 | 0.003757082 | 0.206140023 | EEF1A1 |
| ENSG00000243746 | 1.62967982 | 9.586077504 | 2.556352057 | 0.039437378 | 0.488356916 | EEF1A1P10 |
| ENSG00000228502 | 0.04694457 | 0.166268052 | 1.824480834 | 0.028539665 | 0.428341617 | EEF1A1P11 |
| ENSG00000250182 | 0.09038769 | 0.388139069 | 2.102375419 | 0.00043633 | 0.088208388 | EEF1A1P13 |
| ENSG00000249855 | 0.02275196 | 0.245638815 | 3.432476087 | 0.010122855 | 0.305909629 | EEF1A1P19 |
| ENSG00000233476 | 0.00286057 | 0.00741329 | 1.373811126 | 0.045420529 | 0.504879453 | EEF1A1P6 |
| ENSG00000249264 | 0.39431455 | 0.973430745 | 1.303731385 | 0.025317714 | 0.410383263 | EEF1A1P9 |
| ENSG00000114942 | 0.01659252 | 0.028175708 | 0.763918701 | 0.004592631 | 0.230174477 | EEF1B2 |
| ENSG00000104529 | 0.01599341 | 0.029396843 | 0.878183565 | 0.013132671 | 0.340494851 | EEF1D |
| ENSG00000172638 | 0.00957798 | 0.096428333 | 3.331663401 | 0.023984894 | 0.405084178 | EFEMP2 |
| ENSG00000115468 | 0.03279219 | 0.066375252 | 1.017293167 | 0.047480148 | 0.510030853 | EFHD1 |
| ENSG00000115504 | 0.05403395 | 0.104955223 | 0.957835979 | 0.010613512 | 0.313175884 | EHBP1 |
| ENSG00000181090 | 0.02895091 | 0.045637875 | 0.656622675 | 0.022781997 | 0.4036381 | EHMT1 |
| ENSG00000100353 | 0.02147511 | 0.037770843 | 0.81460747 | 0.003308351 | 0.201098109 | EIF3D |
| ENSG00000130811 | 0.09607227 | 0.159691671 | 0.733097111 | 0.04026662 | 0.489418046 | EIF3G |
| ENSG00000178982 | 0.07601848 | 0.128181284 | 0.753763543 | 0.016556374 | 0.36320919 | EIF3K |
| ENSG00000149100 | 0.11907727 | 0.158186911 | 0.40973224 | 0.016445301 | 0.36320919 | EIF3M |
| ENSG00000114867 | 0.02177745 | 0.034530729 | 0.665045929 | 0.006295217 | 0.254496623 | EIF4G1 |
| ENSG00000233830 | 0.00521095 | 0.07429381 | 3.833623168 | 0.000159289 | 0.050959942 | EIF4HP1 |
| ENSG00000141642 | 0.00848035 | 0.064778195 | 2.93331306 | 0.0045274 | 0.229331663 | ELAC1 |
| ENSG00000163435 | 0.26910461 | 0.61326227 | 1.188337068 | 0.035246681 | 0.469376604 | ELF3 |
| ENSG00000102034 | 0.01441189 | 0.030675028 | 1.089804769 | 0.014826003 | 0.35232888 | ELF4 |
| ENSG00000111145 | 0.03551525 | 0.018445391 | -0.945178292 | 0.0149641 | 0.352555358 | ELK3 |
| ENSG00000110675 | 0.02535406 | 0.135336867 | 2.416266001 | 0.000767921 | 0.111447705 | ELMOD1 |
| ENSG00000127774 | 1.27265929 | 0.572268577 | -1.153081949 | 0.042594188 | 0.49982388 | EMC6 |
| ENSG00000132205 | 0.0103098 | 0.02565711 | 1.315342296 | 0.026729587 | 0.416672417 | EMILIN2 |
| ENSG00000197217 | 0.04392256 | 0.021500353 | -1.03060189 | 0.026586189 | 0.416672417 | ENTPD4 |
| ENSG00000120533 | 0.23477221 | 0.360889621 | 0.620296016 | 0.005444691 | 0.237693156 | ENY2 |
| ENSG00000231993 | 1.55068989 | 0.189792833 | -3.030412783 | 0.019738956 | 0.385178947 | EP300-AS1 |
| ENSG00000185684 | 0.0569529 | 0.013512656 | -2.075458048 | 0.018446471 | 0.376403414 | EP400P1 |
| ENSG00000086289 | 0.05966781 | 0.040281557 | -0.56683342 | 0.045551552 | 0.504879453 | EPDR1 |
| ENSG00000152223 | 0.03003867 | 0.056734931 | 0.917416363 | 0.002069934 | 0.166009597 | EPG5 |
| ENSG00000116106 | 0.12443642 | 0.059015986 | -1.076231093 | 0.021084342 | 0.395798212 | EPHA4 |
| ENSG00000141736 | 0.08536182 | 0.049271191 | -0.792846545 | 0.002137224 | 0.167285336 | ERBB2 |
| ENSG00000065361 | 0.18220812 | 0.099751681 | -0.869174217 | 0.047677335 | 0.510030853 | ERBB3 |
| ENSG00000124882 | 0.10471562 | 0.053909132 | -0.957875111 | 0.043623067 | 0.502426364 | EREG |
| ENSG00000089248 | 0.06830788 | 0.11732876 | 0.780432782 | 0.009819254 | 0.30080542 | ERP29 |
| ENSG00000089048 | 0.04284964 | 0.078267305 | 0.86912685 | 0.035319869 | 0.469376604 | ESF1 |
| ENSG00000140009 | 0.4705082 | 0.046588213 | -3.336182951 | 0.00480812 | 0.232710694 | ESR2 |
| ENSG00000182944 | 0.06431847 | 0.14701815 | 1.192689271 | 0.001989602 | 0.162332811 | EWSR1 |
| ENSG00000178997 | 0.50504752 | 0.132917855 | -1.925884226 | 0.048357348 | 0.512090833 | EXD1 |
| ENSG00000174371 | 0.05270525 | 0.102377117 | 0.957874602 | 0.01940992 | 0.385178947 | EXO1 |
| ENSG00000171824 | 0.05587099 | 0.093741291 | 0.746585387 | 0.02335483 | 0.405084178 | EXOSC10 |
| ENSG00000123737 | 0.12055079 | 0.188119904 | 0.64201135 | 0.04274627 | 0.500406003 | EXOSC9 |
| ENSG00000151348 | 0.14155435 | 0.10983766 | -0.365983263 | 0.04658855 | 0.507102816 | EXT2 |
| ENSG00000106462 | 0.0249721 | 0.054883569 | 1.136057144 | 0.003624134 | 0.206140023 | EZH2 |
| ENSG00000092820 | 0.02050105 | 0.03444305 | 0.748514774 | 0.0451945 | 0.504879453 | EZR |
| ENSG00000158769 | 0.00836519 | 0.001614191 | -2.373586753 | 0.033859069 | 0.462181339 | F11R |
| ENSG00000162585 | 0.03435226 | 0.015065498 | -1.189156486 | 0.004249906 | 0.222554422 | FAAP20 |
| ENSG00000175182 | 0.0150315 | 0.007085052 | -1.08513858 | 0.012505023 | 0.335746964 | FAM131A |
| ENSG00000204442 | 0.033171 | 0.113870289 | 1.779397008 | 0.003429213 | 0.204933179 | FAM155A |
| ENSG00000051009 | 0.03673275 | 0.059830369 | 0.703811114 | 0.036535949 | 0.477185192 | FAM160A2 |
| ENSG00000158863 | 0.04177088 | 0.019128892 | -1.126744327 | 0.011248683 | 0.321009949 | FAM160B2 |
| ENSG00000156050 | 0.01287015 | 0.042610564 | 1.727181948 | 0.041489988 | 0.495475231 | FAM161B |
| ENSG00000119812 | 0.03656672 | 0.065385992 | 0.838450263 | 0.007541083 | 0.274481884 | FAM98A |
| ENSG00000203780 | 0.02154497 | 0.43095598 | 4.322117441 | 0.002551172 | 0.181371889 | FANK1 |
| ENSG00000116120 | 0.0490531 | 0.082470726 | 0.749537653 | 0.01407707 | 0.345040551 | FARSB |
| ENSG00000215251 | 0.04978816 | 0.100902394 | 1.019085825 | 0.018546158 | 0.376403414 | FASTKD5 |
| ENSG00000105202 | 0.06050408 | 0.100471265 | 0.731678583 | 0.01014079 | 0.305909629 | FBL |
| ENSG00000147364 | 0.03152456 | 0.063958686 | 1.020664182 | 0.024802543 | 0.409035682 | FBXO25 |
| ENSG00000163013 | 0.00212104 | 0.008972097 | 2.080676385 | 0.016131563 | 0.36320919 | FBXO41 |
| ENSG00000037637 | 0.06889004 | 0.03709875 | -0.892924881 | 0.01620025 | 0.36320919 | FBXO42 |
| ENSG00000132879 | 0.0778297 | 0.036603941 | -1.088321815 | 0.012529578 | 0.335746964 | FBXO44 |
| ENSG00000204923 | 0.05295061 | 0.141331389 | 1.416362578 | 0.047961332 | 0.510030853 | FBXO48 |
| ENSG00000137478 | 0.03741121 | 0.01505992 | -1.312756537 | 0.006412982 | 0.255615174 | FCHSD2 |
| ENSG00000161513 | 0.0355003 | 0.083888907 | 1.240648959 | 0.008241291 | 0.284628907 | FDXR |
| ENSG00000180263 | 0.07077723 | 0.028525455 | -1.311035367 | 0.007967877 | 0.2807695 | FGD6 |
| ENSG00000129682 | 0.17861124 | 0.020841691 | -3.099278638 | 0.030871457 | 0.442917637 | FGF13 |
| ENSG00000172456 | 0.32820362 | 0.07607103 | -2.109172112 | 0.0056039 | 0.239720683 | FGGY |
| ENSG00000130720 | 0.09922757 | 0.468675224 | 2.239775598 | 1.16E-11 | 1.41E-07 | FIBCD1 |
| ENSG00000145216 | 0.21362092 | 0.352579353 | 0.722895064 | 0.019639485 | 0.385178947 | FIP1L1 |
| ENSG00000180211 | 0.08614376 | 1.344711777 | 3.964406937 | 0.000484053 | 0.090596883 | FO393411.1 |
| ENSG00000175592 | 0.06361275 | 0.10377518 | 0.706073577 | 0.0288083 | 0.429477935 | FOSL1 |
| ENSG00000149531 | 0.00300334 | 0.013901146 | 2.210563295 | 0.031815593 | 0.450270272 | FRG1BP |
| ENSG00000167996 | 0.00769884 | 0.015086861 | 0.970579687 | 0.013876273 | 0.344966687 | FTH1 |
| ENSG00000068438 | 0.03558757 | 0.054226259 | 0.607618267 | 0.030989607 | 0.443089419 | FTSJ1 |
| ENSG00000162613 | 0.06556961 | 0.16886495 | 1.364770719 | 0.009088921 | 0.296533481 | FUBP1 |
| ENSG00000114416 | 0.10603009 | 0.172138833 | 0.699098878 | 0.021229539 | 0.395798212 | FXR1 |
| ENSG00000163820 | 0.06052983 | 0.032922648 | -0.878565993 | 0.023126293 | 0.405084178 | FYCO1 |
| ENSG00000104290 | 0.16411234 | 0.087248728 | -0.911477697 | 0.042981762 | 0.501467643 | FZD3 |
| ENSG00000160211 | 0.04811122 | 0.072500872 | 0.591624981 | 0.042783609 | 0.500406003 | G6PD |
| ENSG00000154727 | 0.02621322 | 0.063280038 | 1.271455733 | 0.003824087 | 0.208472776 | GABPA |
| ENSG00000156958 | 0.10615409 | 0.179481606 | 0.757675997 | 0.040250338 | 0.489418046 | GALK2 |
| ENSG00000139629 | 0.05367767 | 0.028600335 | -0.908290089 | 0.021015341 | 0.395798212 | GALNT6 |
| ENSG00000183087 | 0.03372353 | 0.022280923 | -0.597946488 | 0.029807148 | 0.438168687 | GAS6 |
| ENSG00000257218 | 0.01258056 | 0.003868011 | -1.701533067 | 0.001897151 | 0.157805871 | GATC |
| ENSG00000005436 | 0.06838182 | 0.036069526 | -0.922832415 | 0.007935604 | 0.2807695 | GCFC2 |
| ENSG00000131979 | 0.04612645 | 0.016461322 | -1.486514055 | 0.011719776 | 0.326782828 | GCH1 |
| ENSG00000092208 | 0.06646463 | 0.157496043 | 1.244656779 | 0.000431938 | 0.088208388 | GEMIN2 |
| ENSG00000179409 | 0.06903364 | 0.092074421 | 0.415500843 | 0.037454272 | 0.479242828 | GEMIN4 |
| ENSG00000152147 | 0.10514749 | 0.177070374 | 0.751908495 | 0.022208801 | 0.401177406 | GEMIN6 |
| ENSG00000178295 | 0.06291651 | 0.645203758 | 3.358244292 | 0.007174176 | 0.267871649 | GEN1 |
| ENSG00000100083 | 0.02752804 | 0.04527906 | 0.717941874 | 0.037853928 | 0.480908927 | GGA1 |
| ENSG00000103365 | 0.01358937 | 0.027868193 | 1.036141316 | 0.026048878 | 0.415970645 | GGA2 |
| ENSG00000149328 | 0.04911447 | 0.100699908 | 1.03584244 | 0.01055078 | 0.312726175 | GLB1L2 |
| ENSG00000138604 | 0.24537262 | 0.148163787 | -0.727781418 | 0.010421653 | 0.310529505 | GLCE |
| ENSG00000122694 | 0.06085078 | 0.022531141 | -1.433355287 | 0.012719798 | 0.337649696 | GLIPR2 |
| ENSG00000148672 | 0.03544084 | 0.047871571 | 0.433756317 | 0.046167781 | 0.506097126 | GLUD1 |
| ENSG00000250959 | 0.0363212 | 0.169390428 | 2.221468624 | 0.015213044 | 0.355663414 | GLUD1P3 |
| ENSG00000146535 | 0.00641931 | 0.002865576 | -1.163593785 | 0.005771492 | 0.240287771 | GNA12 |
| ENSG00000127920 | 0.10384614 | 0.156270822 | 0.589600874 | 0.011107571 | 0.319596419 | GNG11 |
| ENSG00000163938 | 0.05788207 | 0.102995523 | 0.83139331 | 0.00473568 | 0.232143812 | GNL3 |
| ENSG00000152133 | 0.06191569 | 0.115480481 | 0.899272125 | 0.012453377 | 0.335746964 | GPATCH11 |
| ENSG00000136235 | 0.32674323 | 0.677411208 | 1.051874504 | 0.049674583 | 0.517032452 | GPNMB |
| ENSG00000143147 | 0.02032609 | 0.010866278 | -0.903474914 | 0.037489506 | 0.479242828 | GPR161 |
| ENSG00000013588 | 0.07766017 | 0.054097243 | -0.521619727 | 0.028966359 | 0.429968281 | GPRC5A |
| ENSG00000185477 | 0.20777734 | 4.914901158 | 4.564052161 | 1.53E-05 | 0.023175769 | GPRIN3 |
| ENSG00000075240 | 0.09742001 | 0.056216624 | -0.793221267 | 0.026763526 | 0.416672417 | GRAMD4 |
| ENSG00000141738 | 0.01147112 | 0.079677151 | 2.79616035 | 0.011054448 | 0.319391043 | GRB7 |
| ENSG00000125388 | 0.02543576 | 0.061878787 | 1.282586938 | 0.024243524 | 0.405403744 | GRK4 |
| ENSG00000132463 | 0.09925566 | 0.140089827 | 0.497130908 | 0.020629383 | 0.393707086 | GRSF1 |
| ENSG00000125651 | 0.02824636 | 0.053918273 | 0.932709249 | 0.001538543 | 0.141697451 | GTF2F1 |
| ENSG00000119041 | 0.07013949 | 0.11901153 | 0.762802455 | 0.009536252 | 0.30080542 | GTF3C3 |
| ENSG00000107937 | 0.03557396 | 0.065974139 | 0.891079062 | 0.000656778 | 0.107195358 | GTPBP4 |
| ENSG00000165996 | 0.03741746 | 0.05516304 | 0.559990236 | 0.03368229 | 0.462181339 | HACD1 |
| ENSG00000084754 | 0.04113884 | 0.065812363 | 0.677857473 | 0.011793046 | 0.326852306 | HADHA |
| ENSG00000180423 | 0.08790851 | 0.036916219 | -1.251748046 | 0.044346799 | 0.502426364 | HARBI1 |
| ENSG00000128708 | 0.17310762 | 0.322300713 | 0.896738129 | 0.003546431 | 0.206140023 | HAT1 |
| ENSG00000152240 | 0.12109135 | 0.212413205 | 0.810777664 | 0.00366642 | 0.206140023 | HAUS1 |
| ENSG00000227214 | 0.09031686 | 0.012658239 | -2.83491869 | 0.004862097 | 0.232710694 | HCG15 |
| ENSG00000099822 | 0.02108943 | 0.005234017 | -2.010529844 | 0.015413779 | 0.357071263 | HCN2 |
| ENSG00000173064 | 0.03834389 | 0.061734937 | 0.687090273 | 0.015100193 | 0.355073579 | HECTD4 |
| ENSG00000148634 | 0.09103523 | 0.143280319 | 0.65434357 | 0.045544044 | 0.504879453 | HERC4 |
| ENSG00000179111 | 0.71166403 | 1.430532401 | 1.007283953 | 0.012728984 | 0.337649696 | HES7 |
| ENSG00000064393 | 0.01332811 | 0.043781757 | 1.7158573 | 0.010367773 | 0.31044585 | HIPK2 |
| ENSG00000100084 | 0.02256862 | 0.004417953 | -2.352868218 | 0.027145663 | 0.420187674 | HIRA |
| ENSG00000233822 | 3.58512433 | 1.196184645 | -1.583583046 | 0.001338274 | 0.135578353 | HIST1H2BN |
| ENSG00000274641 | 58.4915701 | 24.57197862 | -1.251214682 | 0.021231141 | 0.395798212 | HIST1H2BO |
| ENSG00000127124 | 0.04475093 | 0.009962027 | -2.167406447 | 0.005415941 | 0.237693156 | HIVEP3 |
| ENSG00000123485 | 0.03838971 | 0.075352695 | 0.972939276 | 0.027872715 | 0.425333513 | HJURP |
| ENSG00000206503 | 0.18760783 | 0.117169345 | -0.67912486 | 0.035851169 | 0.471698475 | HLA-A |
| ENSG00000164104 | 0.14227804 | 0.22027401 | 0.630586293 | 0.022556527 | 0.40226007 | HMGB2 |
| ENSG00000112972 | 0.05604685 | 0.173862923 | 1.633245122 | 0.02175581 | 0.399524742 | HMGCS1 |
| ENSG00000118418 | 0.08197898 | 0.139590313 | 0.767872817 | 0.043383177 | 0.502426364 | HMGN3 |
| ENSG00000135486 | 0.02242555 | 0.045485699 | 1.020269691 | 0.001368872 | 0.136404738 | HNRNPA1 |
| ENSG00000092199 | 0.03181202 | 0.04746591 | 0.577319721 | 0.035324605 | 0.469376604 | HNRNPC |
| ENSG00000204253 | 0.00262417 | 0.032036419 | 3.609780184 | 0.003435598 | 0.204933179 | HNRNPCP2 |
| ENSG00000138668 | 0.19213265 | 0.287046379 | 0.579181139 | 0.019713074 | 0.385178947 | HNRNPD |
| ENSG00000152795 | 0.07232695 | 0.135138609 | 0.901834774 | 0.007135644 | 0.267871649 | HNRNPDL |
| ENSG00000126945 | 0.06767777 | 0.131177381 | 0.954764967 | 0.019318105 | 0.384370217 | HNRNPH2 |
| ENSG00000096746 | 0.2531962 | 0.37747829 | 0.576137941 | 0.048965331 | 0.513607879 | HNRNPH3 |
| ENSG00000165119 | 0.04425934 | 0.075763071 | 0.775512788 | 0.004707896 | 0.231716148 | HNRNPK |
| ENSG00000099783 | 0.02975473 | 0.066813075 | 1.167011523 | 0.009076712 | 0.296533481 | HNRNPM |
| ENSG00000125944 | 0.05953711 | 0.094927973 | 0.67304399 | 0.013836928 | 0.344966687 | HNRNPR |
| ENSG00000153187 | 0.05166216 | 0.08560018 | 0.728505938 | 0.000955539 | 0.119062573 | HNRNPU |
| ENSG00000214753 | 0.01750799 | 0.00691833 | -1.339517579 | 0.03586876 | 0.471698475 | HNRNPUL2 |
| ENSG00000051128 | 0.01643162 | 0.032545527 | 0.98598439 | 0.032502379 | 0.454616267 | HOMER3 |
| ENSG00000164120 | 0.07248525 | 0.328656748 | 2.180822222 | 0.001482666 | 0.140261844 | HPGD |
| ENSG00000119471 | 0.08646825 | 0.148688296 | 0.782048636 | 0.00514187 | 0.233910516 | HSDL2 |
| ENSG00000096384 | 0.02344775 | 0.034784415 | 0.568991711 | 0.040257342 | 0.489418046 | HSP90AB1 |
| ENSG00000204388 | 0.05056537 | 0.105523736 | 1.061345863 | 0.01529658 | 0.356246208 | HSPA1B |
| ENSG00000170606 | 0.0699714 | 0.110309189 | 0.656715625 | 0.042010189 | 0.49919058 | HSPA4 |
| ENSG00000109971 | 0.01899191 | 0.041325217 | 1.121637462 | 0.001080594 | 0.122773641 | HSPA8 |
| ENSG00000113013 | 0.04734584 | 0.072929391 | 0.623262685 | 0.028267094 | 0.426092576 | HSPA9 |
| ENSG00000144381 | 0.02742673 | 0.050784684 | 0.88881088 | 0.005188444 | 0.233910516 | HSPD1 |
| ENSG00000166411 | 0.09351155 | 0.161066274 | 0.784437972 | 0.026647938 | 0.416672417 | IDH3A |
| ENSG00000148057 | 0.04224681 | 0.086111866 | 1.027369576 | 0.0278869 | 0.425333513 | IDNK |
| ENSG00000160888 | 0.00974238 | 0.003366958 | -1.532828842 | 0.018144679 | 0.375712616 | IER2 |
| ENSG00000187535 | 0.02426899 | 0.051534765 | 1.086431819 | 0.037369439 | 0.479242828 | IFT140 |
| ENSG00000162729 | 0.16973901 | 0.089970841 | -0.915788792 | 0.003256797 | 0.200979115 | IGSF8 |
| ENSG00000113141 | 0.03313294 | 0.07178059 | 1.115327462 | 0.000559465 | 0.097163063 | IK |
| ENSG00000160712 | 0.04072756 | 0.117158755 | 1.524387434 | 0.005064264 | 0.233692211 | IL6R |
| ENSG00000143621 | 0.03281725 | 0.055731746 | 0.764044936 | 0.010888545 | 0.316679518 | ILF2 |
| ENSG00000267100 | 0.01748786 | 0.035094499 | 1.004890935 | 0.034053139 | 0.463450714 | ILF3-DT |
| ENSG00000132323 | 0.01830307 | 0.037669743 | 1.041320388 | 0.020379394 | 0.392565178 | ILKAP |
| ENSG00000153487 | 0.04052731 | 0.082194641 | 1.020149981 | 0.025948594 | 0.415621945 | ING1 |
| ENSG00000169592 | 0.11889458 | 0.223629237 | 0.911425812 | 0.021879982 | 0.400562807 | INO80E |
| ENSG00000164880 | 0.02630564 | 0.04732201 | 0.847139388 | 0.020504967 | 0.392565178 | INTS1 |
| ENSG00000205339 | 0.05773865 | 0.095469871 | 0.725508149 | 0.014350284 | 0.34780523 | IPO7 |
| ENSG00000259673 | 0.01101654 | 0.066195165 | 2.58705475 | 0.013098328 | 0.340494851 | IQCH-AS1 |
| ENSG00000140575 | 0.06674975 | 0.098720323 | 0.564584599 | 0.048262168 | 0.51161076 | IQGAP1 |
| ENSG00000146243 | 0.02001442 | 0.112766586 | 2.494227953 | 0.007238833 | 0.268300293 | IRAK1BP1 |
| ENSG00000167378 | 0.038755 | 0.073920457 | 0.931591085 | 0.014734219 | 0.35232888 | IRGQ |
| ENSG00000185950 | 0.02250017 | 0.044660977 | 0.98907899 | 0.013349715 | 0.342389222 | IRS2 |
| ENSG00000240682 | 0.01410921 | 0.000497845 | -4.824795225 | 2.29E-05 | 0.027854597 | ISY1 |
| ENSG00000111203 | 0.02114389 | 0.060079655 | 1.506635509 | 0.038688079 | 0.484377942 | ITFG2 |
| ENSG00000132470 | 0.29419196 | 0.178902614 | -0.717583354 | 0.029902654 | 0.438447748 | ITGB4 |
| ENSG00000082781 | 0.07073323 | 0.043083751 | -0.71524424 | 0.012080891 | 0.331529111 | ITGB5 |
| ENSG00000148841 | 0.04717208 | 0.023735766 | -0.990870531 | 0.005552088 | 0.239350141 | ITPRIP |
| ENSG00000163166 | 0.15788612 | 0.224109246 | 0.50531785 | 0.033794401 | 0.462181339 | IWS1 |
| ENSG00000102221 | 0.03572044 | 0.070621627 | 0.983360117 | 0.040083681 | 0.489418046 | JADE3 |
| ENSG00000096968 | 0.02498392 | 0.090465078 | 1.856361402 | 0.029574868 | 0.43731045 | JAK2 |
| ENSG00000153814 | 0.15960713 | 0.049333508 | -1.693885306 | 0.003687941 | 0.206140023 | JAZF1 |
| ENSG00000149596 | 0.02549008 | 0.001245612 | -4.355008963 | 0.000408402 | 0.088208388 | JPH2 |
| ENSG00000130522 | 0.00890433 | 0.015990041 | 0.844594938 | 0.016150139 | 0.36320919 | JUND |
| ENSG00000182359 | 0.21277154 | 0.375691986 | 0.820245161 | 0.015585643 | 0.357071263 | KBTBD3 |
| ENSG00000168676 | 0.05365268 | 0.309639954 | 2.528869442 | 0.000132509 | 0.050959942 | KCTD19 |
| ENSG00000180901 | 0.0315127 | 0.016339444 | -0.947574365 | 0.008145864 | 0.282940761 | KCTD2 |
| ENSG00000115548 | 0.07228303 | 0.121772483 | 0.752459283 | 0.029723006 | 0.437991009 | KDM3A |
| ENSG00000121774 | 0.07684062 | 0.122972819 | 0.678398487 | 0.044178565 | 0.502426364 | KHDRBS1 |
| ENSG00000120549 | 0.16101643 | 0.322570746 | 1.002407679 | 0.001602834 | 0.142153335 | KIAA1217 |
| ENSG00000138182 | 0.11200242 | 0.237784915 | 1.086127289 | 0.013124399 | 0.340494851 | KIF20B |
| ENSG00000186638 | 0.02102602 | 0.04784956 | 1.186329695 | 0.024311606 | 0.405642519 | KIF24 |
| ENSG00000155090 | 0.03711664 | 0.066081316 | 0.832176149 | 0.02199334 | 0.401027305 | KLF10 |
| ENSG00000067082 | 0.05722275 | 0.03746142 | -0.611183353 | 0.017458667 | 0.369395776 | KLF6 |
| ENSG00000119138 | 0.25496676 | 0.081532852 | -1.644855774 | 0.005357617 | 0.237693156 | KLF9 |
| ENSG00000185909 | 0.0101678 | 0.039037384 | 1.940848288 | 0.010649006 | 0.313462384 | KLHDC8B |
| ENSG00000162755 | 0.06032294 | 0.009956989 | -2.598925225 | 0.00634025 | 0.255226563 | KLHDC9 |
| ENSG00000003096 | 0.12038606 | 0.045683958 | -1.397908756 | 0.046739594 | 0.507102816 | KLHL13 |
| ENSG00000102271 | 0.37573572 | 1.766048707 | 2.232734969 | 0.019523413 | 0.385178947 | KLHL4 |
| ENSG00000171798 | 0.02588312 | 0.188125827 | 2.861614609 | 3.31E-05 | 0.03390847 | KNDC1 |
| ENSG00000137812 | 0.05920758 | 0.117102971 | 0.98392395 | 0.03778604 | 0.480908927 | KNL1 |
| ENSG00000214012 | 0.39424192 | 3.200523447 | 3.021154785 | 0.000558688 | 0.097163063 | KRT18P38 |
| ENSG00000002549 | 0.09651123 | 0.166188028 | 0.784047694 | 0.00600969 | 0.246823668 | LAP3 |
| ENSG00000237008 | 0.08698788 | 1.970029319 | 4.501258842 | 0.004004038 | 0.216342649 | LAPTM4BP1 |
| ENSG00000174720 | 0.056891 | 0.108367196 | 0.929655673 | 0.021516339 | 0.397728112 | LARP7 |
| ENSG00000131023 | 0.11874512 | 0.06027194 | -0.978309825 | 0.01599317 | 0.362065114 | LATS1 |
| ENSG00000148346 | 0.43982149 | 0.959415177 | 1.125237152 | 0.001867342 | 0.157805871 | LCN2 |
| ENSG00000169744 | 0.14058031 | 0.360836046 | 1.359948912 | 0.027959714 | 0.425333513 | LDB2 |
| ENSG00000105617 | 0.19563032 | 0.439769538 | 1.168617714 | 0.012974371 | 0.339353641 | LENG1 |
| ENSG00000167615 | 0.02403478 | 0.045651593 | 0.925541562 | 0.005678996 | 0.239720683 | LENG8 |
| ENSG00000050426 | 0.02780834 | 0.055592276 | 0.999366694 | 0.004053583 | 0.218050454 | LETMD1 |
| ENSG00000145685 | 0.08345833 | 0.042931637 | -0.959014942 | 0.016419113 | 0.36320919 | LHFPL2 |
| ENSG00000105486 | 0.02147562 | 0.055317924 | 1.365046988 | 0.005183009 | 0.233910516 | LIG1 |
| ENSG00000182541 | 0.03288195 | 0.066385221 | 1.013566114 | 0.037613033 | 0.479808703 | LIMK2 |
| ENSG00000227036 | 0.1253847 | 0.035243277 | -1.830941327 | 0.014368891 | 0.34780523 | LINC00511 |
| ENSG00000237854 | 0.04802653 | 0.014128479 | -1.765225337 | 0.022176803 | 0.401177406 | LINC00674 |
| ENSG00000245060 | 0.01305005 | 0.004770144 | -1.451950834 | 0.044412972 | 0.502426364 | LINC00847 |
| ENSG00000224914 | 0.03125659 | 0.004534602 | -2.785112627 | 0.005007091 | 0.233222988 | LINC00863 |
| ENSG00000231711 | 0.00540879 | 0.041399876 | 2.936248885 | 0.033637532 | 0.462181339 | LINC00899 |
| ENSG00000240476 | 0.02384463 | 0.061526229 | 1.367537338 | 0.039730597 | 0.488356916 | LINC00973 |
| ENSG00000274020 | 0.85845932 | 0.290722567 | -1.562106706 | 0.001174816 | 0.129499425 | LINC01138 |
| ENSG00000245937 | 2.98632697 | 1.313164941 | -1.185323995 | 0.0004087 | 0.088208388 | LINC01184 |
| ENSG00000234380 | 0.08828278 | 0.045867621 | -0.944656038 | 0.042307236 | 0.499348607 | LINC01426 |
| ENSG00000223485 | 0.23418858 | 0.067430331 | -1.796201178 | 0.028799789 | 0.429477935 | LINC01615 |
| ENSG00000223784 | 0.01840629 | 0.044217429 | 1.264416551 | 0.030126073 | 0.439139898 | LINP1 |
| ENSG00000113368 | 0.06291549 | 0.116546491 | 0.889418504 | 0.013464092 | 0.343150868 | LMNB1 |
| ENSG00000253741 | 0.10669132 | 0.01622505 | -2.717147936 | 0.046726372 | 0.507102816 | LNCOC1 |
| ENSG00000175556 | 0.04822259 | 0.019354351 | -1.317051108 | 0.041180344 | 0.493381361 | LONRF3 |
| ENSG00000113083 | 0.14318302 | 0.096363225 | -0.571305861 | 0.045330464 | 0.504879453 | LOX |
| ENSG00000138131 | 0.28878342 | 0.093928419 | -1.620354281 | 0.006446383 | 0.255890575 | LOXL4 |
| ENSG00000175445 | 0.64271577 | 2.264884688 | 1.817184816 | 0.011623329 | 0.325587133 | LPL |
| ENSG00000110031 | 0.06164455 | 0.016783517 | -1.876928369 | 0.014375585 | 0.34780523 | LPXN |
| ENSG00000010626 | 0.05692274 | 0.159852594 | 1.489665166 | 0.045769449 | 0.504917595 | LRRC23 |
| ENSG00000181350 | 0.03146598 | 0.006130723 | -2.359663547 | 0.00285143 | 0.190466149 | LRRC75A |
| ENSG00000093167 | 0.0509958 | 0.10392304 | 1.027065161 | 0.001707059 | 0.148233687 | LRRFIP2 |
| ENSG00000154237 | 0.04894391 | 0.022909455 | -1.09518621 | 0.012221456 | 0.333879182 | LRRK1 |
| ENSG00000143429 | 0.05503092 | 0.012058707 | -2.190167256 | 7.74E-05 | 0.040908317 | LSP1P4 |
| ENSG00000111144 | 0.12263579 | 0.191341268 | 0.64176798 | 0.031016624 | 0.443089419 | LTA4H |
| ENSG00000135521 | 0.08277451 | 0.1565216 | 0.919103316 | 0.047790248 | 0.510030853 | LTV1 |
| ENSG00000169641 | 0.01920811 | 0.032677601 | 0.766586314 | 0.038539338 | 0.484377942 | LUZP1 |
| ENSG00000145220 | 0.0982429 | 0.157495905 | 0.680889308 | 0.034592855 | 0.468313295 | LYAR |
| ENSG00000163155 | 0.02637846 | 0.06124455 | 1.215221366 | 0.027981921 | 0.425333513 | LYSMD1 |
| ENSG00000143669 | 0.09135548 | 0.044814025 | -1.027540957 | 0.023568871 | 0.405084178 | LYST |
| ENSG00000185022 | 0.04099901 | 0.023293474 | -0.815663228 | 0.015593664 | 0.357071263 | MAFF |
| ENSG00000081026 | 0.04437506 | 0.117720835 | 1.407548623 | 0.004849098 | 0.232710694 | MAGI3 |
| ENSG00000162385 | 0.56997645 | 0.936527423 | 0.716418928 | 0.011975969 | 0.330140261 | MAGOH |
| ENSG00000165072 | 0.28295598 | 0.102235817 | -1.468676919 | 7.94E-05 | 0.040908317 | MAMDC2 |
| ENSG00000177239 | 0.12167388 | 0.079430167 | -0.615260555 | 0.021558085 | 0.397728112 | MAN1B1 |
| ENSG00000145050 | 0.29769791 | 0.42164302 | 0.502172993 | 0.040496517 | 0.490000424 | MANF |
| ENSG00000073803 | 0.23071436 | 0.419739115 | 0.863385127 | 0.001045235 | 0.119876609 | MAP3K13 |
| ENSG00000180815 | 0.02225498 | 0.066899728 | 1.587872031 | 0.018087682 | 0.375712616 | MAP3K15 |
| ENSG00000169967 | 0.05244956 | 0.02409297 | -1.122318333 | 0.007002321 | 0.267510526 | MAP3K2 |
| ENSG00000142733 | 0.01108315 | 0.033792238 | 1.608323661 | 0.032253846 | 0.452782917 | MAP3K6 |
| ENSG00000006432 | 0.01617471 | 0.006077247 | -1.412249821 | 0.016913846 | 0.365874784 | MAP3K9 |
| ENSG00000084764 | 0.10630227 | 0.052525909 | -1.0170713 | 0.024857308 | 0.409035682 | MAPRE3 |
| ENSG00000075413 | 0.03968121 | 0.062612499 | 0.657994627 | 0.028535133 | 0.428341617 | MARK3 |
| ENSG00000007047 | 0.02202294 | 0.045921687 | 1.060168251 | 0.030349791 | 0.440458386 | MARK4 |
| ENSG00000155254 | 0.01309054 | 0.003679331 | -1.831008818 | 0.003743726 | 0.206140023 | MARVELD1 |
| ENSG00000105613 | 0.05251136 | 0.019372676 | -1.438606375 | 0.018690946 | 0.378709719 | MAST1 |
| ENSG00000168906 | 0.06063827 | 0.085308124 | 0.49245452 | 0.044715486 | 0.502873418 | MAT2A |
| ENSG00000171444 | 0.11181294 | 0.041939053 | -1.414720967 | 0.00445021 | 0.229242368 | MCC |
| ENSG00000101977 | 0.04094749 | 0.349313076 | 3.092673792 | 0.039815934 | 0.488356916 | MCF2 |
| ENSG00000065328 | 0.04662131 | 0.142261774 | 1.609486641 | 6.71E-05 | 0.040908317 | MCM10 |
| ENSG00000073111 | 0.02355583 | 0.042076325 | 0.836924356 | 0.008897071 | 0.296533481 | MCM2 |
| ENSG00000104738 | 0.03423053 | 0.060121222 | 0.812590811 | 0.021044585 | 0.395798212 | MCM4 |
| ENSG00000100297 | 0.02145397 | 0.047428654 | 1.1445144 | 0.001444174 | 0.139339887 | MCM5 |
| ENSG00000076003 | 0.06411421 | 0.094381557 | 0.55786092 | 0.038667938 | 0.484377942 | MCM6 |
| ENSG00000166508 | 0.04240996 | 0.07276192 | 0.778780318 | 0.007783865 | 0.27750279 | MCM7 |
| ENSG00000111877 | 0.04099753 | 0.103024946 | 1.329384677 | 0.044228554 | 0.502426364 | MCM9 |
| ENSG00000153898 | 0.13468322 | 0.069400066 | -0.956561165 | 0.009897938 | 0.300823082 | MCOLN2 |
| ENSG00000172366 | 0.01501719 | 0.00734592 | -1.031599799 | 0.046837309 | 0.507102816 | MCRIP2 |
| ENSG00000050393 | 0.01523917 | 0.024922883 | 0.709686854 | 0.046646398 | 0.507102816 | MCUR1 |
| ENSG00000137337 | 0.05073667 | 0.089774167 | 0.823271487 | 0.00570435 | 0.239957738 | MDC1 |
| ENSG00000135272 | 0.03066989 | 0.055699473 | 0.860840758 | 0.035150048 | 0.469376604 | MDFIC |
| ENSG00000175221 | 0.04036769 | 0.023924711 | -0.75469963 | 0.024157772 | 0.405084178 | MED16 |
| ENSG00000148297 | 0.02524708 | 0.013914602 | -0.859516939 | 0.039849474 | 0.488356916 | MED22 |
| ENSG00000136146 | 0.1433209 | 0.223476774 | 0.640875933 | 0.048181899 | 0.51156973 | MED4 |
| ENSG00000105976 | 0.21247871 | 0.132931371 | -0.67663671 | 0.015439243 | 0.357071263 | MET |
| ENSG00000037897 | 0.06673057 | 0.171985803 | 1.365869695 | 0.000137767 | 0.050959942 | METTL1 |
| ENSG00000171806 | 0.0869653 | 0.201337472 | 1.211104007 | 0.006106883 | 0.249537582 | METTL18 |
| ENSG00000165171 | 0.02142205 | 0.108892589 | 2.345737095 | 0.021190904 | 0.395798212 | METTL27 |
| ENSG00000176624 | 0.09576528 | 0.04595924 | -1.05914776 | 0.00500454 | 0.233222988 | MEX3C |
| ENSG00000181588 | 0.00882559 | 0.018242762 | 1.047559431 | 0.017384833 | 0.368887387 | MEX3D |
| ENSG00000140259 | 0.08183406 | 0.127075196 | 0.634909154 | 0.042254368 | 0.499209277 | MFAP1 |
| ENSG00000198948 | 0.0918149 | 0.02973006 | -1.626805937 | 0.043034681 | 0.501603655 | MFAP3L |
| ENSG00000156875 | 2.09327834 | 0.333244363 | -2.651111778 | 0.007660969 | 0.27610902 | MFSD14A |
| ENSG00000085871 | 0.13070016 | 0.204618855 | 0.646678132 | 0.047017313 | 0.508079535 | MGST2 |
| ENSG00000027001 | 0.05085615 | 0.093598879 | 0.880068873 | 0.014896524 | 0.35232888 | MIPEP |
| ENSG00000233325 | 0.10159161 | 0.598623308 | 2.558867201 | 0.026625382 | 0.416672417 | MIPEPP3 |
| ENSG00000225206 | 0.0132739 | 0.049762543 | 1.90646738 | 0.00044133 | 0.088208388 | MIR137HG |
| ENSG00000207721 | 33.1514714 | 1154.173521 | 5.121643424 | 0.000982692 | 0.119062573 | MIR186 |
| ENSG00000207870 | 2027.00352 | 22896.29244 | 3.497693507 | 0.005102617 | 0.233692211 | MIR221 |
| ENSG00000270069 | 0.06805269 | 0.162987044 | 1.260033219 | 0.003742972 | 0.206140023 | MIR222HG |
| ENSG00000268471 | 0.01990929 | 0.079246147 | 1.99289881 | 0.001320555 | 0.134907466 | MIR4453HG |
| ENSG00000247516 | 0.052363 | 0.016304924 | -1.68324014 | 0.010572547 | 0.312726175 | MIR4458HG |
| ENSG00000167965 | 0.02276793 | 0.03732273 | 0.713051283 | 0.042544339 | 0.49982388 | MLST8 |
| ENSG00000175727 | 0.02155216 | 0.011661989 | -0.886018704 | 0.024324537 | 0.405642519 | MLXIP |
| ENSG00000132763 | 0.03148278 | 0.06195044 | 0.976551804 | 0.036763791 | 0.477603236 | MMACHC |
| ENSG00000070444 | 0.03164294 | 0.058546666 | 0.887703458 | 0.040406329 | 0.490000424 | MNT |
| ENSG00000142961 | 0.00403764 | 0.02357756 | 2.545830486 | 0.025607179 | 0.412873312 | MOB3C |
| ENSG00000103111 | 0.05161444 | 0.028270964 | -0.868453674 | 0.013247846 | 0.340494851 | MON1B |
| ENSG00000060762 | 0.05519651 | 0.022021663 | -1.325653754 | 0.001573238 | 0.142153335 | MPC1 |
| ENSG00000158186 | 0.0015138 | 0.020523755 | 3.761045686 | 0.000548454 | 0.097163063 | MRAS |
| ENSG00000204839 | 0.00187873 | 0.030928531 | 4.041109171 | 0.001700246 | 0.148233687 | MROH6 |
| ENSG00000169288 | 0.03601892 | 0.061857856 | 0.780201809 | 0.031615 | 0.448842787 | MRPL1 |
| ENSG00000180992 | 0.04824806 | 0.030355433 | -0.668516159 | 0.017818287 | 0.374194788 | MRPL14 |
| ENSG00000143314 | 0.11836735 | 0.206418589 | 0.802301687 | 0.009283643 | 0.29843347 | MRPL24 |
| ENSG00000204316 | 0.04186278 | 0.137931425 | 1.720211125 | 0.037975863 | 0.480908927 | MRPL38 |
| ENSG00000175581 | 0.09703298 | 0.148023904 | 0.609283024 | 0.038428128 | 0.483613619 | MRPL48 |
| ENSG00000143436 | 0.06046674 | 0.149856932 | 1.309372187 | 9.17E-06 | 0.01592516 | MRPL9 |
| ENSG00000102738 | 0.10297474 | 0.159445951 | 0.630776929 | 0.039687282 | 0.488356916 | MRPS31 |
| ENSG00000186260 | 0.04194595 | 0.079994499 | 0.931369292 | 0.020166113 | 0.391628485 | MRTFB |
| ENSG00000116062 | 0.040791 | 0.10062499 | 1.302665784 | 0.000818094 | 0.113280615 | MSH6 |
| ENSG00000188895 | 0.02220856 | 0.009085665 | -1.289451856 | 0.000328403 | 0.081477359 | MSL1 |
| ENSG00000125148 | 0.06179863 | 0.025091134 | -1.300397055 | 0.011483804 | 0.323073567 | MT2A |
| ENSG00000198938 | 0.00442829 | 0.005839722 | 0.399150989 | 0.044593078 | 0.502426364 | MT-CO3 |
| ENSG00000120832 | 0.11896922 | 0.034704275 | -1.777403134 | 0.016563493 | 0.36320919 | MTERF2 |
| ENSG00000143033 | 0.07401905 | 0.131200371 | 0.825803343 | 0.042785525 | 0.500406003 | MTF2 |
| ENSG00000242114 | 1.04940065 | 0.039073499 | -4.747231324 | 0.000508198 | 0.092211386 | MTFP1 |
| ENSG00000177000 | 0.02580591 | 0.048916738 | 0.922626713 | 0.018292023 | 0.375940647 | MTHFR |
| ENSG00000136371 | 0.2324336 | 0.045406122 | -2.355859895 | 0.040575674 | 0.490000424 | MTHFS |
| ENSG00000198695 | 0.18401831 | 0.288803853 | 0.650240691 | 0.044544664 | 0.502426364 | MT-ND6 |
| ENSG00000132613 | 0.01547044 | 0.032588687 | 1.0748568 | 0.035296636 | 0.469376604 | MTSS2 |
| ENSG00000173171 | 0.16017208 | 0.24703479 | 0.625091588 | 0.023384374 | 0.405084178 | MTX1 |
| ENSG00000185499 | 0.06597096 | 0.026174336 | -1.333678206 | 0.044399241 | 0.502426364 | MUC1 |
| ENSG00000183486 | 0.0088608 | 0.049699398 | 2.487719938 | 0.049578928 | 0.516591873 | MX2 |
| ENSG00000119950 | 0.04625324 | 0.023761858 | -0.96090666 | 0.037652176 | 0.479808703 | MXI1 |
| ENSG00000118513 | 0.03510273 | 0.230085897 | 2.712517481 | 0.000353555 | 0.085963419 | MYB |
| ENSG00000132382 | 0.03422133 | 0.053080882 | 0.633296684 | 0.019570864 | 0.385178947 | MYBBP1A |
| ENSG00000104177 | 0.11795822 | 0.197967901 | 0.746990513 | 0.017932466 | 0.375223734 | MYEF2 |
| ENSG00000172927 | 0.00944517 | 0.019818167 | 1.069174694 | 0.001859899 | 0.157805871 | MYEOV |
| ENSG00000176182 | 0.00209835 | 0.015657232 | 2.899501293 | 0.013588386 | 0.344872665 | MYPOP |
| ENSG00000204899 | 0.1746116 | 0.284204286 | 0.702778873 | 0.012274465 | 0.334575508 | MZT1 |
| ENSG00000122390 | 0.01748488 | 0.005898537 | -1.567679018 | 0.006556192 | 0.257881042 | NAA60 |
| ENSG00000187109 | 0.04502522 | 0.074224552 | 0.721163296 | 0.003600684 | 0.206140023 | NAP1L1 |
| ENSG00000177432 | 0.11653299 | 0.050848876 | -1.196450626 | 0.036889812 | 0.477603236 | NAP1L5 |
| ENSG00000204272 | 0.03796714 | 0.018922821 | -1.004624104 | 0.002183621 | 0.16801443 | NBDY |
| ENSG00000151503 | 0.05066463 | 0.077207622 | 0.607764341 | 0.04017186 | 0.489418046 | NCAPD3 |
| ENSG00000109805 | 0.09349586 | 0.192539649 | 1.04218121 | 0.03562564 | 0.471646631 | NCAPG |
| ENSG00000123338 | 0.40602427 | 3.235320053 | 2.994270551 | 0.042823094 | 0.500406003 | NCKAP1L |
| ENSG00000115053 | 0.06404908 | 0.140455699 | 1.13286533 | 4.56E-07 | 0.001847272 | NCL |
| ENSG00000140396 | 0.04642537 | 0.116893988 | 1.332215371 | 0.004620116 | 0.230174477 | NCOA2 |
| ENSG00000188211 | 0.07871254 | 0.012112725 | -2.700069981 | 0.032185207 | 0.452629065 | NCR3LG1 |
| ENSG00000125356 | 0.39400067 | 0.843035486 | 1.097395287 | 4.43E-05 | 0.03390847 | NDUFA1 |
| ENSG00000170906 | 0.19161284 | 0.507672681 | 1.405704411 | 3.82E-05 | 0.03390847 | NDUFA3 |
| ENSG00000237037 | 0.42161599 | 0.04561681 | -3.208292086 | 0.003132343 | 0.199184608 | NDUFA6-DT |
| ENSG00000117691 | 0.02489647 | 0.01337269 | -0.896651306 | 0.017093772 | 0.366399131 | NENF |
| ENSG00000100968 | 0.01940099 | 0.082660464 | 2.091067535 | 0.004510759 | 0.229331663 | NFATC4 |
| ENSG00000008441 | 0.01416565 | 0.033783942 | 1.253940567 | 0.044682223 | 0.502873418 | NFIX |
| ENSG00000204498 | 0.04756214 | 0.012106772 | -1.973999198 | 0.0001921 | 0.058383979 | NFKBIL1 |
| ENSG00000144802 | 0.05190076 | 0.089855181 | 0.791845961 | 0.025035302 | 0.409078177 | NFKBIZ |
| ENSG00000129460 | 0.09209407 | 0.134966099 | 0.551416974 | 0.043310756 | 0.502413036 | NGDN |
| ENSG00000177453 | 0.13458745 | 0.023836085 | -2.497324681 | 0.039174623 | 0.488356916 | NIM1K |
| ENSG00000170113 | 0.0428946 | 0.023149355 | -0.889823946 | 0.035847797 | 0.471698475 | NIPA1 |
| ENSG00000001461 | 0.05627401 | 0.033574213 | -0.745115143 | 0.013894279 | 0.344966687 | NIPAL3 |
| ENSG00000285967 | 0.0071519 | 0.042336419 | 2.565500587 | 0.000828875 | 0.113280615 | NIPBL-DT |
| ENSG00000184117 | 0.09508138 | 0.060059881 | -0.662761157 | 0.040228717 | 0.489418046 | NIPSNAP1 |
| ENSG00000197885 | 0.0834169 | 0.150202734 | 0.848499431 | 0.027373517 | 0.421530402 | NKIRAS1 |
| ENSG00000186416 | 0.0385409 | 0.079343552 | 1.041722846 | 0.017570644 | 0.370201596 | NKRF |
| ENSG00000167034 | 0.00257209 | 0.026227779 | 3.350082239 | 0.002897633 | 0.191448478 | NKX3-1 |
| ENSG00000022556 | 0.03049716 | 0.073059735 | 1.260401681 | 0.004496926 | 0.229331663 | NLRP2 |
| ENSG00000135577 | 0.10861498 | 2.020526294 | 4.217436133 | 0.017502013 | 0.369395776 | NMBR |
| ENSG00000053438 | 0.05417759 | 2.231882034 | 5.36442068 | 0.001532624 | 0.141697451 | NNAT |
| ENSG00000188976 | 0.04470451 | 0.066856469 | 0.580646687 | 0.038080902 | 0.481369204 | NOC2L |
| ENSG00000166197 | 0.03007617 | 0.065749005 | 1.128348406 | 0.001298934 | 0.134907466 | NOLC1 |
| ENSG00000146909 | 0.02149927 | 0.039597128 | 0.881108097 | 0.009252511 | 0.29843347 | NOM1 |
| ENSG00000147140 | 0.01687383 | 0.02765574 | 0.712791304 | 0.009784693 | 0.30080542 | NONO |
| ENSG00000182117 | 0.10652711 | 0.168084455 | 0.657965644 | 0.023462253 | 0.405084178 | NOP10 |
| ENSG00000131697 | 0.04365759 | 0.016472393 | -1.406182241 | 0.018749129 | 0.379256515 | NPHP4 |
| ENSG00000181163 | 0.02281627 | 0.041425003 | 0.860439138 | 0.000469033 | 0.09050855 | NPM1 |
| ENSG00000235677 | 1.33563747 | 8.560540256 | 2.680173378 | 0.019696844 | 0.385178947 | NPM1P26 |
| ENSG00000225159 | 0.53211462 | 7.312976166 | 3.780649706 | 0.001613651 | 0.142153335 | NPM1P39 |
| ENSG00000169418 | 0.02814226 | 0.128534283 | 2.191343078 | 0.026248484 | 0.416672417 | NPR1 |
| ENSG00000156642 | 0.13053178 | 0.188425717 | 0.529594788 | 0.024074357 | 0.405084178 | NPTN |
| ENSG00000164128 | 0.51287979 | 0.067149857 | -2.933164493 | 0.000311875 | 0.080669571 | NPY1R |
| ENSG00000115216 | 0.03954352 | 0.068405795 | 0.790677135 | 0.016685043 | 0.36351266 | NRBP1 |
| ENSG00000091129 | 0.02558112 | 0.293476709 | 3.520094711 | 0.001789308 | 0.154273891 | NRCAM |
| ENSG00000175352 | 0.22413402 | 0.017538987 | -3.675724368 | 0.013635033 | 0.344938512 | NRIP3 |
| ENSG00000126653 | 0.0520539 | 0.119019141 | 1.193115446 | 0.000443075 | 0.088208388 | NSRP1 |
| ENSG00000037474 | 0.05731268 | 0.089104159 | 0.636638355 | 0.017211426 | 0.36644362 | NSUN2 |
| ENSG00000074590 | 0.03771335 | 0.016273126 | -1.212583747 | 0.003013265 | 0.195312271 | NUAK1 |
| ENSG00000069275 | 0.11250544 | 0.174487799 | 0.633131447 | 0.029265012 | 0.433871641 | NUCKS1 |
| ENSG00000090273 | 0.11497087 | 0.170860055 | 0.571546747 | 0.044586441 | 0.502426364 | NUDC |
| ENSG00000112874 | 0.04444482 | 0.092806113 | 1.06220462 | 0.033611598 | 0.462181339 | NUDT12 |
| ENSG00000083635 | 0.04055593 | 0.076370728 | 0.913107064 | 0.041377069 | 0.495099439 | NUFIP1 |
| ENSG00000108256 | 0.04539715 | 0.06921515 | 0.60848626 | 0.026532807 | 0.416672417 | NUFIP2 |
| ENSG00000111581 | 0.06500365 | 0.117842209 | 0.85826369 | 0.009660432 | 0.30080542 | NUP107 |
| ENSG00000030066 | 0.05603903 | 0.088697766 | 0.662465837 | 0.037904996 | 0.480908927 | NUP160 |
| ENSG00000226328 | 0.02023155 | 0.004057892 | -2.317804271 | 0.002166387 | 0.167750125 | NUP50-DT |
| ENSG00000145247 | 0.09522155 | 0.129394219 | 0.442413198 | 0.049167667 | 0.51439873 | OCIAD2 |
| ENSG00000111325 | 0.21123282 | 0.09546914 | -1.145727636 | 0.005296733 | 0.236736683 | OGFOD2 |
| ENSG00000119547 | 0.51879076 | 0.240305829 | -1.110281149 | 0.00155083 | 0.141755164 | ONECUT2 |
| ENSG00000172057 | 0.02788865 | 0.013301604 | -1.068078033 | 0.016949824 | 0.366001793 | ORMDL3 |
| ENSG00000092094 | 0.10058859 | 0.144565754 | 0.523259156 | 0.049020237 | 0.513740534 | OSGEP |
| ENSG00000089723 | 0.08801163 | 0.024354799 | -1.853488153 | 0.003150827 | 0.199184608 | OTUB2 |
| ENSG00000154124 | 0.03401656 | 0.019094425 | -0.833085595 | 0.042131149 | 0.49919058 | OTULIN |
| ENSG00000155463 | 0.0714716 | 0.108576772 | 0.603273424 | 0.015778489 | 0.359885722 | OXA1L |
| ENSG00000083720 | 0.04234088 | 0.134674814 | 1.669356999 | 0.00100491 | 0.119356439 | OXCT1 |
| ENSG00000175591 | 0.04384207 | 0.015000023 | -1.547351072 | 0.048758958 | 0.513607879 | P2RY2 |
| ENSG00000100836 | 0.03170086 | 0.072162423 | 1.186725688 | 0.00845166 | 0.288614691 | PABPN1 |
| ENSG00000006712 | 0.07936531 | 0.175194864 | 1.142379954 | 0.001356757 | 0.136314801 | PAF1 |
| ENSG00000120727 | 0.23938092 | 0.388140084 | 0.69726929 | 0.016574532 | 0.36320919 | PAIP2 |
| ENSG00000130669 | 0.02229349 | 0.042233095 | 0.921751479 | 0.018373748 | 0.376403414 | PAK4 |
| ENSG00000227345 | 0.03350667 | 0.05982361 | 0.836266774 | 0.01558264 | 0.357071263 | PARG |
| ENSG00000143799 | 0.0260121 | 0.042059181 | 0.693237902 | 0.028605805 | 0.428804893 | PARP1 |
| ENSG00000041880 | 0.03838196 | 0.021226836 | -0.854538833 | 0.028253289 | 0.426092576 | PARP3 |
| ENSG00000166889 | 0.0328288 | 0.047926239 | 0.545853669 | 0.023154794 | 0.405084178 | PATL1 |
| ENSG00000214106 | 0.08001514 | 0.024040392 | -1.734812505 | 0.01482685 | 0.35232888 | PAXIP1-AS2 |
| ENSG00000168078 | 0.08577106 | 0.189989923 | 1.147360028 | 0.011507021 | 0.323073567 | PBK |
| ENSG00000185630 | 1.11108615 | 0.264331707 | -2.07154929 | 0.016236761 | 0.36320919 | PBX1 |
| ENSG00000163346 | 0.24799679 | 0.165243793 | -0.585725347 | 0.022809852 | 0.4036381 | PBXIP1 |
| ENSG00000156453 | 0.04697013 | 0.569550502 | 3.600008425 | 0.000736674 | 0.111447705 | PCDH1 |
| ENSG00000120324 | 0.33927762 | 0.040710252 | -3.059002177 | 4.46E-05 | 0.03390847 | PCDHB10 |
| ENSG00000240184 | 0.08481457 | 0.2346905 | 1.468375337 | 0.017179198 | 0.366399131 | PCDHGC3 |
| ENSG00000165494 | 0.0475893 | 0.092822092 | 0.96383087 | 0.002431254 | 0.179131877 | PCF11 |
| ENSG00000100982 | 0.0400365 | 0.02320529 | -0.786862322 | 0.014882981 | 0.35232888 | PCIF1 |
| ENSG00000171408 | 0.11353748 | 1.248493349 | 3.458947665 | 0.000251574 | 0.072405882 | PDE7B |
| ENSG00000180867 | 0.00492227 | 0.029050294 | 2.561156025 | 0.008856571 | 0.296533481 | PDIA3P1 |
| ENSG00000175087 | 0.13788484 | 0.056411051 | -1.289414115 | 0.033849234 | 0.462181339 | PDIK1L |
| ENSG00000241360 | 0.0091101 | 0.070047827 | 2.942801126 | 0.000729235 | 0.111447705 | PDXP |
| ENSG00000132326 | 0.01327712 | 0.062508687 | 2.235114164 | 0.000158401 | 0.050959942 | PER2 |
| ENSG00000152556 | 0.05899991 | 0.092594002 | 0.65020596 | 0.021698549 | 0.399076037 | PFKM |
| ENSG00000142102 | 0.04049431 | 0.102384294 | 1.338203179 | 0.016070897 | 0.363148505 | PGGHG |
| ENSG00000079739 | 0.06343901 | 0.138081552 | 1.122078348 | 0.00122543 | 0.130680255 | PGM1 |
| ENSG00000139289 | 0.02161006 | 0.011921524 | -0.858134475 | 0.013871612 | 0.344966687 | PHLDA1 |
| ENSG00000246640 | 0.18137329 | 0.016124445 | -3.491640595 | 0.010876425 | 0.316679518 | PICART1 |
| ENSG00000108474 | 0.24869038 | 0.116364014 | -1.095705746 | 0.001412284 | 0.138368597 | PIGL |
| ENSG00000121879 | 0.08541027 | 0.148021258 | 0.793322891 | 0.030232127 | 0.440158043 | PIK3CA |
| ENSG00000105851 | 0.10779852 | 0.32631217 | 1.597915466 | 0.00334238 | 0.202155776 | PIK3CG |
| ENSG00000117461 | 0.04110816 | 0.223377079 | 2.441984621 | 0.000714232 | 0.11131941 | PIK3R3 |
| ENSG00000196455 | 0.06890152 | 0.124024492 | 0.848017311 | 0.009527112 | 0.30080542 | PIK3R4 |
| ENSG00000170965 | 0.22520535 | 0.049369957 | -2.189535805 | 0.005113279 | 0.233692211 | PLAC1 |
| ENSG00000181690 | 0.11274008 | 0.024420455 | -2.206838457 | 0.000908065 | 0.118770642 | PLAG1 |
| ENSG00000114805 | 0.01297531 | 0.140731147 | 3.43910121 | 0.002775916 | 0.188489189 | PLCH1 |
| ENSG00000100558 | 0.04692599 | 0.027321496 | -0.780350814 | 0.047114509 | 0.508677697 | PLEK2 |
| ENSG00000143850 | 0.01442162 | 0.027768994 | 0.945241421 | 0.034729797 | 0.46912238 | PLEKHA6 |
| ENSG00000166689 | 0.03417938 | 0.012994575 | -1.395216853 | 0.039753891 | 0.488356916 | PLEKHA7 |
| ENSG00000134297 | 0.17999765 | 0.010079046 | -4.15854715 | 0.002895612 | 0.191448478 | PLEKHA8P1 |
| ENSG00000171680 | 0.01985876 | 0.052459832 | 1.401437619 | 0.043244261 | 0.502413036 | PLEKHG5 |
| ENSG00000023902 | 0.01756386 | 0.044271807 | 1.333778551 | 0.011124282 | 0.319596419 | PLEKHO1 |
| ENSG00000166851 | 0.02787857 | 0.055862823 | 1.002732094 | 0.046889733 | 0.507151673 | PLK1 |
| ENSG00000188313 | 0.08920491 | 0.165764794 | 0.893942548 | 0.005195018 | 0.233910516 | PLSCR1 |
| ENSG00000130827 | 0.07604928 | 0.038477914 | -0.982904044 | 0.003127523 | 0.199184608 | PLXNA3 |
| ENSG00000196576 | 0.09822515 | 0.069619859 | -0.496593588 | 0.041697379 | 0.497463233 | PLXNB2 |
| ENSG00000004399 | 0.04161175 | 0.020017681 | -1.055716249 | 0.007632338 | 0.27610902 | PLXND1 |
| ENSG00000160783 | 0.01993725 | 0.048337339 | 1.277671545 | 0.015196401 | 0.355663414 | PMF1 |
| ENSG00000165688 | 0.05329024 | 0.081782403 | 0.617919028 | 0.022165514 | 0.401177406 | PMPCA |
| ENSG00000100941 | 0.08203107 | 0.161695496 | 0.979037064 | 0.004182804 | 0.221088474 | PNN |
| ENSG00000198805 | 0.06647476 | 0.110468372 | 0.732754872 | 0.003656002 | 0.206140023 | PNP |
| ENSG00000106628 | 0.03568624 | 0.06036155 | 0.758261826 | 0.006301183 | 0.254496623 | POLD2 |
| ENSG00000100479 | 0.02847016 | 0.059835351 | 1.071547475 | 0.033860018 | 0.462181339 | POLE2 |
| ENSG00000186184 | 0.01637346 | 0.027908168 | 0.769328369 | 0.008321976 | 0.285791686 | POLR1D |
| ENSG00000137054 | 0.04949486 | 0.082159301 | 0.731145286 | 0.009447391 | 0.30080542 | POLR1E |
| ENSG00000168002 | 0.12911407 | 0.193266272 | 0.581943656 | 0.006891459 | 0.266676147 | POLR2G |
| ENSG00000255529 | 0.01354593 | 0.003244051 | -2.061991267 | 0.003649234 | 0.206140023 | POLR2M |
| ENSG00000058600 | 0.03653367 | 0.020392671 | -0.841176028 | 0.037552901 | 0.479548962 | POLR3E |
| ENSG00000113356 | 0.0209355 | 0.055383267 | 1.403498512 | 0.008620865 | 0.29274821 | POLR3G |
| ENSG00000266066 | 0.04010883 | 0.005444198 | -2.881128547 | 0.019548105 | 0.385178947 | POLRMTP1 |
| ENSG00000104356 | 0.01428979 | 0.034173291 | 1.257884004 | 0.009608891 | 0.30080542 | POP1 |
| ENSG00000143847 | 0.01885265 | 0.16437999 | 3.124195268 | 0.009843806 | 0.30080542 | PPFIA4 |
| ENSG00000163590 | 0.16261717 | 0.042823361 | -1.925009665 | 0.008144951 | 0.282940761 | PPM1L |
| ENSG00000104881 | 0.05543473 | 0.096327317 | 0.797154936 | 0.014870256 | 0.35232888 | PPP1R13L |
| ENSG00000173457 | 0.01209125 | 0.023482136 | 0.957600224 | 0.021592544 | 0.397728112 | PPP1R14B |
| ENSG00000073711 | 0.21086612 | 0.10958857 | -0.944229983 | 0.039948979 | 0.489083316 | PPP2R3A |
| ENSG00000120910 | 0.1163103 | 0.205687329 | 0.822474019 | 0.022922486 | 0.405041662 | PPP3CC |
| ENSG00000124224 | 0.12063034 | 0.212073527 | 0.813971719 | 0.023550336 | 0.405084178 | PPP4R1L |
| ENSG00000100239 | 0.14401218 | 0.077876393 | -0.886932873 | 0.00138096 | 0.136490496 | PPP6R2 |
| ENSG00000110075 | 0.10376919 | 0.158457851 | 0.610720919 | 0.026684445 | 0.416672417 | PPP6R3 |
| ENSG00000130711 | 0.14915681 | 0.015261191 | -3.288890339 | 0.007740776 | 0.27677828 | PRDM12 |
| ENSG00000138738 | 0.03847865 | 0.119325503 | 1.63277221 | 0.000145528 | 0.050959942 | PRDM5 |
| ENSG00000146143 | 0.00980526 | 0.029056607 | 1.567238783 | 0.000487738 | 0.090596883 | PRIM2 |
| ENSG00000106617 | 0.01486774 | 0.026270812 | 0.821275862 | 0.023348107 | 0.405084178 | PRKAG2 |
| ENSG00000163558 | 0.05459267 | 0.09238372 | 0.758931295 | 0.005993572 | 0.246823668 | PRKCI |
| ENSG00000138669 | 0.08133465 | 0.221058183 | 1.442484198 | 0.016434518 | 0.36320919 | PRKG2 |
| ENSG00000223960 | 0.07339613 | 0.020594975 | -1.833411721 | 0.027201423 | 0.420187674 | PRKRA-AS1 |
| ENSG00000113494 | 0.30187467 | 0.055891663 | -2.433244706 | 0.024712999 | 0.408332514 | PRLR |
| ENSG00000185238 | 0.09181588 | 0.148829647 | 0.696846374 | 0.04503204 | 0.504879453 | PRMT3 |
| ENSG00000117360 | 0.05561199 | 0.09136976 | 0.716320775 | 0.047467006 | 0.510030853 | PRPF3 |
| ENSG00000141127 | 0.08739962 | 0.134738445 | 0.624462701 | 0.032436513 | 0.454298026 | PRPSAP2 |
| ENSG00000164099 | 0.03684922 | 0.076019723 | 1.0447398 | 0.047786778 | 0.510030853 | PRSS12 |
| ENSG00000196415 | 0.0233922 | 0.230656834 | 3.301648562 | 0.026995185 | 0.41913213 | PRTN3 |
| ENSG00000156011 | 0.01832908 | 0.048572903 | 1.406017039 | 0.041164455 | 0.493381361 | PSD3 |
| ENSG00000205155 | 0.48076034 | 1.28060169 | 1.413432036 | 0.005647999 | 0.239720683 | PSENEN |
| ENSG00000164985 | 0.05556535 | 0.112062711 | 1.012048795 | 0.011673148 | 0.326230951 | PSIP1 |
| ENSG00000100567 | 0.25642441 | 0.380305274 | 0.568624327 | 0.027446546 | 0.421530402 | PSMA3 |
| ENSG00000257621 | 0.05083648 | 0.097000032 | 0.932121173 | 0.030036101 | 0.438447748 | PSMA3-AS1 |
| ENSG00000205220 | 0.1002204 | 0.470978377 | 2.232484659 | 0.000146746 | 0.050959942 | PSMB10 |
| ENSG00000126067 | 0.10578524 | 0.163533598 | 0.628448782 | 0.046733223 | 0.507102816 | PSMB2 |
| ENSG00000100764 | 0.02480087 | 0.049918886 | 1.009195169 | 0.024685281 | 0.408332514 | PSMC1 |
| ENSG00000161057 | 0.0993352 | 0.151903741 | 0.612780488 | 0.033295043 | 0.461536874 | PSMC2 |
| ENSG00000100519 | 0.07266841 | 0.117143808 | 0.688880518 | 0.014936579 | 0.352555358 | PSMC6 |
| ENSG00000101843 | 0.03313692 | 0.057038645 | 0.783500162 | 0.04600473 | 0.505789031 | PSMD10 |
| ENSG00000108671 | 0.06032883 | 0.085522619 | 0.503458533 | 0.042077802 | 0.49919058 | PSMD11 |
| ENSG00000159352 | 0.06063945 | 0.095042397 | 0.648314628 | 0.037961338 | 0.480908927 | PSMD4 |
| ENSG00000121390 | 0.03754537 | 0.083358458 | 1.150693687 | 0.000116156 | 0.048693202 | PSPC1 |
| ENSG00000112655 | 0.10570173 | 0.072355072 | -0.546832886 | 0.020811044 | 0.395798212 | PTK7 |
| ENSG00000187514 | 0.06868608 | 0.107954307 | 0.65233117 | 0.043295703 | 0.502413036 | PTMA |
| ENSG00000159335 | 0.02375549 | 0.044795362 | 0.915088511 | 0.008310332 | 0.285791686 | PTMS |
| ENSG00000105894 | 0.23271792 | 0.671188186 | 1.528135001 | 0.004626696 | 0.230174477 | PTN |
| ENSG00000076201 | 0.03220291 | 0.063239781 | 0.973641329 | 0.005767356 | 0.240287771 | PTPN23 |
| ENSG00000060656 | 0.06763976 | 0.03178539 | -1.089507797 | 0.028827342 | 0.429477935 | PTPRU |
| ENSG00000162927 | 0.03474707 | 0.093444039 | 1.427211442 | 0.043115244 | 0.502061318 | PUS10 |
| ENSG00000160953 | 0.01120918 | 0.028613216 | 1.352000644 | 0.000979713 | 0.119062573 | PWWP3A |
| ENSG00000155893 | 0.07759066 | 0.021994718 | -1.818725808 | 0.008787039 | 0.295094016 | PXYLP1 |
| ENSG00000172053 | 0.04824312 | 0.066860143 | 0.470823114 | 0.046414007 | 0.507102816 | QARS |
| ENSG00000179912 | 0.0340256 | 0.012971186 | -1.391309951 | 0.024098369 | 0.405084178 | R3HDM2 |
| ENSG00000139998 | 0.00227652 | 0.012385327 | 2.443732282 | 0.014037428 | 0.345040551 | RAB15 |
| ENSG00000100228 | 0.05381694 | 0.02416031 | -1.155421454 | 0.046547784 | 0.507102816 | RAB36 |
| ENSG00000127328 | 0.03661075 | 0.0637257 | 0.7996079 | 0.024100111 | 0.405084178 | RAB3IP |
| ENSG00000141542 | 0.02468224 | 0.006842254 | -1.850929729 | 0.043554307 | 0.502426364 | RAB40B |
| ENSG00000101084 | 0.01987444 | 0.038111352 | 0.939306287 | 0.016280343 | 0.36320919 | RAB5IF |
| ENSG00000183155 | 0.14639975 | 0.211258394 | 0.529095543 | 0.047412478 | 0.510030853 | RABIF |
| ENSG00000204628 | 0.06555899 | 0.120965841 | 0.883734227 | 0.002534314 | 0.181233262 | RACK1 |
| ENSG00000002016 | 0.05386768 | 0.098656962 | 0.873001001 | 0.036528766 | 0.477185192 | RAD52 |
| ENSG00000147231 | 0.21172131 | 0.589872913 | 1.478237665 | 0.012502532 | 0.335746964 | RADX |
| ENSG00000144118 | 0.13192328 | 0.096834999 | -0.44609867 | 0.026107231 | 0.415970645 | RALB |
| ENSG00000160271 | 0.03362241 | 0.003525286 | -3.253610863 | 0.015345865 | 0.356710675 | RALGDS |
| ENSG00000099901 | 0.03074788 | 0.049255222 | 0.679789816 | 0.040298385 | 0.489418046 | RANBP1 |
| ENSG00000031823 | 0.05804169 | 0.085924484 | 0.56597966 | 0.047187581 | 0.509014576 | RANBP3 |
| ENSG00000107263 | 0.01980977 | 0.010119749 | -0.969038472 | 0.007404306 | 0.272770155 | RAPGEF1 |
| ENSG00000079337 | 0.03594853 | 0.011988329 | -1.584301998 | 0.045709038 | 0.504879453 | RAPGEF3 |
| ENSG00000108551 | 0.06609807 | 0.296644868 | 2.166056667 | 0.008369005 | 0.286597172 | RASD1 |
| ENSG00000105538 | 0.02368551 | 0.145528815 | 2.619228179 | 0.034790705 | 0.469376604 | RASIP1 |
| ENSG00000101265 | 0.02900151 | 0.010331538 | -1.489073022 | 0.030361448 | 0.440458386 | RASSF2 |
| ENSG00000153179 | 0.04524493 | 0.028006808 | -0.691978691 | 0.022734581 | 0.4036381 | RASSF3 |
| ENSG00000146587 | 0.01452835 | 0.052841387 | 1.862797451 | 0.015153465 | 0.355638371 | RBAK |
| ENSG00000122257 | 0.04467947 | 0.095719932 | 1.099207399 | 0.00147513 | 0.140261844 | RBBP6 |
| ENSG00000080839 | 0.13735209 | 0.223232041 | 0.700665252 | 0.016234204 | 0.36320919 | RBL1 |
| ENSG00000227354 | 0.16119921 | 0.033758815 | -2.255508535 | 0.003188025 | 0.199184608 | RBM26-AS1 |
| ENSG00000163694 | 0.10098192 | 0.476533041 | 2.238479274 | 0.019468046 | 0.385178947 | RBM47 |
| ENSG00000147274 | 0.02479611 | 0.054660909 | 1.140395367 | 0.001904886 | 0.157805871 | RBMX |
| ENSG00000134597 | 0.04946524 | 0.089158731 | 0.849960997 | 0.019647735 | 0.385178947 | RBMX2 |
| ENSG00000136161 | 0.20494257 | 0.034133518 | -2.585958686 | 0.000380605 | 0.088208388 | RCBTB2 |
| ENSG00000004700 | 0.08247286 | 0.182013303 | 1.142052469 | 0.000883615 | 0.118045084 | RECQL |
| ENSG00000164620 | 0.00703675 | 0.044405317 | 2.657751536 | 0.023916561 | 0.405084178 | RELL2 |
| ENSG00000154153 | 0.18702252 | 0.098854664 | -0.919831033 | 0.045724461 | 0.504879453 | RETREG1 |
| ENSG00000076043 | 0.07941773 | 0.118153061 | 0.573123899 | 0.026768212 | 0.416672417 | REXO2 |
| ENSG00000222881 | 0.13630938 | 12.11470625 | 6.47373074 | 0.001488342 | 0.140261844 | RF00019 |
| ENSG00000252671 | 0.11816231 | 0.777317741 | 2.717734451 | 0.002643036 | 0.183607934 | RF00019 |
| ENSG00000163918 | 0.10622404 | 0.167611225 | 0.658008418 | 0.02996597 | 0.438447748 | RFC4 |
| ENSG00000072422 | 0.08540749 | 0.037143507 | -1.20125263 | 0.040453196 | 0.490000424 | RHOBTB1 |
| ENSG00000119729 | 0.04733568 | 0.021090531 | -1.166332506 | 0.024098334 | 0.405084178 | RHOQ |
| ENSG00000080345 | 0.14133159 | 0.229868724 | 0.701726174 | 0.024015472 | 0.405084178 | RIF1 |
| ENSG00000177181 | 0.07702693 | 0.008892166 | -3.114756135 | 0.041011066 | 0.493381361 | RIMKLA |
| ENSG00000166532 | 0.07236028 | 0.129681951 | 0.841707774 | 0.027615398 | 0.422821654 | RIMKLB |
| ENSG00000176406 | 0.03841863 | 0.015570722 | -1.302970058 | 0.021163301 | 0.395798212 | RIMS2 |
| ENSG00000100599 | 0.00904088 | 0.01892175 | 1.065510129 | 0.030728417 | 0.442612992 | RIN3 |
| ENSG00000104312 | 0.05337789 | 0.027709346 | -0.945869523 | 0.023795089 | 0.405084178 | RIPK2 |
| ENSG00000199691 | 2.83690058 | 0.2435781 | -3.54185926 | 0.013166674 | 0.340494851 | RN7SKP173 |
| ENSG00000123091 | 0.0366384 | 0.02169235 | -0.756170143 | 0.019711736 | 0.385178947 | RNF11 |
| ENSG00000120925 | 0.07097009 | 0.033721854 | -1.073527197 | 0.023292881 | 0.405084178 | RNF170 |
| ENSG00000099999 | 0.07047952 | 0.041149775 | -0.776319645 | 0.04317593 | 0.502286875 | RNF215 |
| ENSG00000011275 | 0.06397701 | 0.036745732 | -0.799976972 | 0.012750452 | 0.337649696 | RNF216 |
| ENSG00000196204 | 0.01655403 | 0.003900394 | -2.085490645 | 0.004086211 | 0.218837274 | RNF216P1 |
| ENSG00000179859 | 0.02049541 | 0.07400577 | 1.852337168 | 0.007009285 | 0.267510526 | RNF227 |
| ENSG00000101236 | 0.0420119 | 0.025717382 | -0.70805435 | 0.031677808 | 0.448842787 | RNF24 |
| ENSG00000170633 | 0.10231532 | 0.155402262 | 0.602985284 | 0.038130998 | 0.481369204 | RNF34 |
| ENSG00000101654 | 0.08386733 | 0.132105313 | 0.655507715 | 0.035217381 | 0.469376604 | RNMT |
| ENSG00000185483 | 0.18870942 | 0.07522613 | -1.326860646 | 6.83E-05 | 0.040908317 | ROR1 |
| ENSG00000132383 | 0.04446063 | 0.076451566 | 0.782017581 | 0.023306442 | 0.405084178 | RPA1 |
| ENSG00000106399 | 0.15583689 | 0.224372972 | 0.525862075 | 0.036543507 | 0.477185192 | RPA3 |
| ENSG00000142676 | 0.09295394 | 0.192170569 | 1.047799537 | 0.001422726 | 0.138368597 | RPL11 |
| ENSG00000167526 | 0.00915864 | 0.016007226 | 0.805517891 | 0.003298201 | 0.201098109 | RPL13 |
| ENSG00000234498 | 0.024108 | 0.001064871 | -4.500761188 | 6.07E-05 | 0.040908317 | RPL13AP20 |
| ENSG00000198242 | 0.02117643 | 0.048173003 | 1.185765762 | 0.000154676 | 0.050959942 | RPL23A |
| ENSG00000161970 | 0.05132419 | 0.133919002 | 1.383649924 | 0.000945636 | 0.119062573 | RPL26 |
| ENSG00000131469 | 0.01660793 | 0.02606413 | 0.650193694 | 0.009516819 | 0.30080542 | RPL27 |
| ENSG00000108107 | 0.04016007 | 0.070756811 | 0.817107288 | 0.008750721 | 0.295094016 | RPL28 |
| ENSG00000156482 | 0.08909831 | 0.048964829 | -0.863652251 | 0.0294454 | 0.436014278 | RPL30 |
| ENSG00000071082 | 0.04933231 | 0.071278336 | 0.530930933 | 0.04786654 | 0.510030853 | RPL31 |
| ENSG00000130255 | 0.09323881 | 0.160947618 | 0.787588685 | 0.020502329 | 0.392565178 | RPL36 |
| ENSG00000165502 | 0.05709945 | 0.121547033 | 1.089965951 | 0.00103352 | 0.119661914 | RPL36AL |
| ENSG00000226243 | 1.05555457 | 6.25306334 | 2.566561965 | 0.048487575 | 0.512583902 | RPL37AP1 |
| ENSG00000122406 | 0.03013634 | 0.042040886 | 0.480288828 | 0.014280338 | 0.34780523 | RPL5 |
| ENSG00000148303 | 0.02263871 | 0.046718417 | 1.045199756 | 8.06E-05 | 0.040908317 | RPL7A |
| ENSG00000161016 | 0.06653072 | 0.133212312 | 1.001634841 | 0.003438872 | 0.204933179 | RPL8 |
| ENSG00000152464 | 0.06669511 | 0.121370779 | 0.863768314 | 0.021259869 | 0.395798212 | RPP38 |
| ENSG00000163125 | 0.04034705 | 0.075795596 | 0.909650776 | 0.002530166 | 0.181233262 | RPRD2 |
| ENSG00000105193 | 0.03497167 | 0.053166201 | 0.604322682 | 0.037445991 | 0.479242828 | RPS16 |
| ENSG00000187051 | 0.03212009 | 0.064539962 | 1.006716964 | 0.009582148 | 0.30080542 | RPS19BP1 |
| ENSG00000138326 | 0.04517646 | 0.118649805 | 1.393066631 | 1.93E-05 | 0.026120989 | RPS24 |
| ENSG00000118181 | 0.06086295 | 0.089761836 | 0.56053798 | 0.047886718 | 0.510030853 | RPS25 |
| ENSG00000212829 | 0.17757675 | 0.93162317 | 2.391303839 | 0.002494353 | 0.180949455 | RPS26P3 |
| ENSG00000178429 | 0.07416852 | 0.24777144 | 1.740131011 | 0.032148766 | 0.452629065 | RPS3AP5 |
| ENSG00000083845 | 0.02941064 | 0.047194109 | 0.682268741 | 0.013981181 | 0.345040551 | RPS5 |
| ENSG00000137154 | 0.07440636 | 0.131866317 | 0.82557817 | 0.001108072 | 0.124729886 | RPS6 |
| ENSG00000108443 | 0.04802351 | 0.088245873 | 0.877788007 | 0.041747335 | 0.497570931 | RPS6KB1 |
| ENSG00000167325 | 0.06087301 | 0.110741396 | 0.863319889 | 0.025529678 | 0.412873312 | RRM1 |
| ENSG00000171848 | 0.06741108 | 0.11355843 | 0.752377185 | 0.046762347 | 0.507102816 | RRM2 |
| ENSG00000137876 | 0.03583156 | 0.062759586 | 0.808605045 | 0.020986761 | 0.395798212 | RSL24D1 |
| ENSG00000251333 | 0.234014 | 0.61642992 | 1.397342037 | 0.022363054 | 0.40226007 | RTN3P1 |
| ENSG00000124813 | 0.11128018 | 0.180257536 | 0.695862942 | 0.031200168 | 0.445188307 | RUNX2 |
| ENSG00000013392 | 0.03961331 | 0.098087974 | 1.308090876 | 0.016410811 | 0.36320919 | RWDD2A |
| ENSG00000170989 | 0.36546201 | 0.047942704 | -2.930338268 | 0.00042858 | 0.088208388 | S1PR1 |
| ENSG00000267534 | 0.06322588 | 0.014587575 | -2.11577507 | 0.008031108 | 0.282179695 | S1PR2 |
| ENSG00000100347 | 0.06316102 | 0.106046961 | 0.747596817 | 0.01702266 | 0.366399131 | SAMM50 |
| ENSG00000136715 | 0.07026148 | 0.120744298 | 0.781149244 | 0.027170306 | 0.420187674 | SAP130 |
| ENSG00000156304 | 0.05131787 | 0.082187576 | 0.679458997 | 0.035015536 | 0.469376604 | SCAF4 |
| ENSG00000010803 | 0.02086348 | 0.003674309 | -2.505434957 | 0.000102999 | 0.044719976 | SCMH1 |
| ENSG00000234684 | 0.08930084 | 0.00675474 | -3.724701609 | 0.002089288 | 0.166009597 | SDCBP2-AS1 |
| ENSG00000117118 | 0.07784014 | 0.123899985 | 0.6705899 | 0.004941819 | 0.233222988 | SDHB |
| ENSG00000100445 | 0.66885782 | 0.208428948 | -1.682143898 | 0.000426665 | 0.088208388 | SDR39U1 |
| ENSG00000065665 | 0.05484453 | 0.136527705 | 1.315773972 | 0.002422633 | 0.179131877 | SEC61A2 |
| ENSG00000127922 | 0.32866393 | 0.571504736 | 0.798152327 | 0.00031862 | 0.080697168 | SEM1 |
| ENSG00000166192 | 0.06454342 | 0.153838356 | 1.253073331 | 0.048857134 | 0.513607879 | SENP8 |
| ENSG00000142864 | 0.0352985 | 0.058870353 | 0.737934499 | 0.014261552 | 0.34780523 | SERBP1 |
| ENSG00000082497 | 0.06820752 | 0.028474081 | -1.260281559 | 0.00213259 | 0.167285336 | SERTAD4 |
| ENSG00000103037 | 0.01359049 | 0.048584718 | 1.837904626 | 0.023724033 | 0.405084178 | SETD6 |
| ENSG00000104897 | 0.04505904 | 0.099962115 | 1.149564992 | 0.005879574 | 0.243122382 | SF3A2 |
| ENSG00000183431 | 0.05724818 | 0.085694097 | 0.581965947 | 0.023903334 | 0.405084178 | SF3A3 |
| ENSG00000198089 | 0.04972081 | 0.022204917 | -1.162970749 | 0.00979699 | 0.30080542 | SFI1 |
| ENSG00000181523 | 0.1364402 | 0.090023321 | -0.599898132 | 0.027520516 | 0.421900264 | SGSH |
| ENSG00000141258 | 0.03608846 | 0.022445594 | -0.685105368 | 0.040734561 | 0.490792918 | SGSM2 |
| ENSG00000175137 | 0.02715052 | 0.015797952 | -0.781242533 | 0.02241441 | 0.40226007 | SH3BP5L |
| ENSG00000125089 | 0.02675452 | 0.119361347 | 2.157481279 | 0.018204846 | 0.375749251 | SH3TC1 |
| ENSG00000148082 | 0.02583975 | 0.05219051 | 1.014195383 | 0.025758989 | 0.414770897 | SHC3 |
| ENSG00000185634 | 0.10412738 | 0.73187745 | 2.813252678 | 0.023251942 | 0.405084178 | SHC4 |
| ENSG00000198892 | 0.04223589 | 0.01449892 | -1.542524013 | 0.012971993 | 0.339353641 | SHISA4 |
| ENSG00000168779 | 0.02800756 | 0.062117963 | 1.149194395 | 0.020448465 | 0.392565178 | SHOX2 |
| ENSG00000163950 | 0.08108643 | 0.144537738 | 0.833913868 | 0.000757827 | 0.111447705 | SLBP |
| ENSG00000124067 | 0.08496769 | 0.057112483 | -0.573108274 | 0.022989406 | 0.405084178 | SLC12A4 |
| ENSG00000079215 | 0.10103864 | 0.453812035 | 2.167187688 | 0.024925334 | 0.409035682 | SLC1A3 |
| ENSG00000168575 | 0.04186403 | 0.023794685 | -0.815071783 | 0.026711258 | 0.416672417 | SLC20A2 |
| ENSG00000163393 | 0.02868234 | 0.010287525 | -1.479266723 | 0.015814421 | 0.360029795 | SLC22A15 |
| ENSG00000004864 | 0.04384786 | 0.094221009 | 1.103542476 | 0.021885693 | 0.400562807 | SLC25A13 |
| ENSG00000075303 | 0.09885605 | 0.186587441 | 0.916450756 | 0.044389338 | 0.502426364 | SLC25A40 |
| ENSG00000181035 | 0.02647636 | 0.009851565 | -1.426279922 | 0.033827328 | 0.462181339 | SLC25A42 |
| ENSG00000181045 | 0.10751269 | 0.064615843 | -0.734547076 | 0.024101987 | 0.405084178 | SLC26A11 |
| ENSG00000143554 | 0.07419357 | 0.007455784 | -3.314862173 | 0.012505022 | 0.335746964 | SLC27A3 |
| ENSG00000205060 | 0.03301917 | 0.056100596 | 0.764712259 | 0.019619122 | 0.385178947 | SLC35B4 |
| ENSG00000130958 | 0.00139802 | 0.019676783 | 3.815037896 | 0.000803064 | 0.113280615 | SLC35D2 |
| ENSG00000175782 | 0.07219417 | 0.029682691 | -1.282260388 | 0.030895299 | 0.442917637 | SLC35E3 |
| ENSG00000142494 | 0.01578128 | 0.135063505 | 3.097351382 | 0.000155606 | 0.050959942 | SLC47A1 |
| ENSG00000080493 | 0.04707444 | 0.284206616 | 2.593924199 | 0.019208422 | 0.384073661 | SLC4A4 |
| ENSG00000103061 | 0.13564064 | 0.264150904 | 0.961572834 | 0.01298013 | 0.339353641 | SLC7A6OS |
| ENSG00000133302 | 0.04204024 | 0.120876887 | 1.523695497 | 0.005003192 | 0.233222988 | SLF1 |
| ENSG00000137776 | 0.08830883 | 0.148878905 | 0.753509737 | 0.012158333 | 0.332902829 | SLTM |
| ENSG00000099956 | 0.08124542 | 0.123916397 | 0.609008628 | 0.013870335 | 0.344966687 | SMARCB1 |
| ENSG00000072501 | 0.02601595 | 0.042530134 | 0.709089182 | 0.026915476 | 0.418428952 | SMC1A |
| ENSG00000113810 | 0.05638318 | 0.108343409 | 0.942274597 | 0.024018733 | 0.405084178 | SMC4 |
| ENSG00000163683 | 0.15614567 | 0.101187503 | -0.62586142 | 0.043853506 | 0.502426364 | SMIM14 |
| ENSG00000172594 | 0.20408907 | 0.1055452 | -0.951337984 | 0.033365421 | 0.461947096 | SMPDL3A |
| ENSG00000019549 | 0.13802552 | 0.034212879 | -2.012323603 | 0.009303799 | 0.29843347 | SNAI2 |
| ENSG00000224078 | 4.36928955 | 0.394936142 | -3.467707411 | 0.00025554 | 0.072405882 | SNHG14 |
| ENSG00000250988 | 0.89687492 | 0.243863574 | -1.878832521 | 0.013922007 | 0.344966687 | SNHG21 |
| ENSG00000242125 | 0.01743445 | 0.029285943 | 0.748267788 | 0.008107704 | 0.282940761 | SNHG3 |
| ENSG00000184602 | 0.00281548 | 0.017266075 | 2.616485996 | 8.37E-05 | 0.040908317 | SNN |
| ENSG00000212443 | 125.754693 | 4.87680579 | -4.688531904 | 9.85E-07 | 0.002323986 | SNORA53 |
| ENSG00000206838 | 20.1638729 | 5.984632335 | -1.752438242 | 0.013676101 | 0.344938512 | SNORA5A |
| ENSG00000207523 | 534.473962 | 173.490143 | -1.623265968 | 0.038174288 | 0.481415782 | SNORA66 |
| ENSG00000199753 | 35.7918622 | 202.6511596 | 2.501294919 | 0.022482755 | 0.40226007 | SNORD104 |
| ENSG00000202314 | 15.0787061 | 59.28487044 | 1.975151342 | 0.024143981 | 0.405084178 | SNORD6 |
| ENSG00000231587 | 1.9587006 | 0.157623119 | -3.635345825 | 0.04440754 | 0.502426364 | SNORD62B |
| ENSG00000184209 | 0.13484583 | 0.373268848 | 1.468904246 | 0.000818393 | 0.113280615 | SNRNP35 |
| ENSG00000125743 | 0.09115916 | 0.132210915 | 0.536381744 | 0.044172025 | 0.502426364 | SNRPD2 |
| ENSG00000120451 | 0.0507747 | 0.029775801 | -0.769969619 | 0.004310079 | 0.223921501 | SNX19 |
| ENSG00000185338 | 0.04231877 | 0.234901823 | 2.472688369 | 0.018087763 | 0.375712616 | SOCS1 |
| ENSG00000142168 | 0.37237245 | 0.548837124 | 0.559631728 | 0.022533464 | 0.40226007 | SOD1 |
| ENSG00000185404 | 0.02694003 | 0.091922963 | 1.770673671 | 0.015903871 | 0.360895332 | SP140L |
| ENSG00000144451 | 0.06402394 | 0.11807671 | 0.883041063 | 0.048619431 | 0.513078495 | SPAG16 |
| ENSG00000122432 | 0.57066006 | 0.065577602 | -3.121356527 | 0.012337488 | 0.335541044 | SPATA1 |
| ENSG00000189419 | 0.29633779 | 0.011599225 | -4.675142276 | 0.001153671 | 0.128671335 | SPATA41 |
| ENSG00000145375 | 0.06540212 | 0.171635246 | 1.391936579 | 0.007573066 | 0.274823161 | SPATA5 |
| ENSG00000123352 | 0.10127428 | 0.047383487 | -1.09581153 | 0.020228679 | 0.391697398 | SPATS2 |
| ENSG00000152582 | 0.05360894 | 0.192772173 | 1.846351302 | 0.00646199 | 0.255890575 | SPEF2 |
| ENSG00000188766 | 0.01363291 | 0.042486134 | 1.639898222 | 0.009566474 | 0.30080542 | SPRED3 |
| ENSG00000010072 | 0.05690937 | 0.095735746 | 0.750391394 | 0.045636971 | 0.504879453 | SPRTN |
| ENSG00000171621 | 0.0491586 | 0.099318863 | 1.014624053 | 0.02044572 | 0.392565178 | SPSB1 |
| ENSG00000197694 | 0.07891074 | 0.119964591 | 0.604315046 | 0.023700878 | 0.405084178 | SPTAN1 |
| ENSG00000171943 | 0.00469097 | 0.017247067 | 1.878392764 | 0.000670136 | 0.107195358 | SRGAP2C |
| ENSG00000153037 | 0.07567269 | 0.034495489 | -1.133364981 | 0.037948432 | 0.480908927 | SRP19 |
| ENSG00000144867 | 0.13069249 | 0.226904776 | 0.795910776 | 0.013242818 | 0.340494851 | SRPRB |
| ENSG00000102359 | 0.18785797 | 0.044421964 | -2.08029726 | 0.000180634 | 0.056306887 | SRPX2 |
| ENSG00000167978 | 0.04361082 | 0.060859121 | 0.480787533 | 0.037405643 | 0.479242828 | SRRM2 |
| ENSG00000087087 | 0.04400492 | 0.102522292 | 1.220201037 | 0.001893256 | 0.157805871 | SRRT |
| ENSG00000100650 | 0.17212451 | 0.267044078 | 0.633625309 | 0.010185527 | 0.306498642 | SRSF5 |
| ENSG00000106028 | 0.13579997 | 0.276633965 | 1.026495149 | 0.000644695 | 0.107195358 | SSBP1 |
| ENSG00000176101 | 0.01482604 | 0.02675434 | 0.851639977 | 0.028715242 | 0.429477935 | SSNA1 |
| ENSG00000100380 | 0.02876395 | 0.049514621 | 0.78359281 | 0.016581484 | 0.36320919 | ST13 |
| ENSG00000115525 | 0.06311876 | 0.025689646 | -1.296881783 | 0.003166685 | 0.199184608 | ST3GAL5 |
| ENSG00000214530 | 0.01296817 | 0.006129545 | -1.08112249 | 0.039272253 | 0.488356916 | STARD10 |
| ENSG00000040341 | 0.06058354 | 0.112713103 | 0.89565757 | 0.018290947 | 0.375940647 | STAU2 |
| ENSG00000168439 | 0.04270184 | 0.076306941 | 0.837516031 | 0.009807921 | 0.30080542 | STIP1 |
| ENSG00000117632 | 0.1286716 | 0.192049724 | 0.57778621 | 0.031648248 | 0.448842787 | STMN1 |
| ENSG00000243317 | 0.06398975 | 0.125135334 | 0.967576576 | 0.000829314 | 0.113280615 | STMP1 |
| ENSG00000128578 | 0.05897204 | 0.124228954 | 1.07489841 | 0.033400633 | 0.461947096 | STRIP2 |
| ENSG00000136143 | 0.10105784 | 0.149975716 | 0.569547629 | 0.048488236 | 0.512583902 | SUCLA2 |
| ENSG00000165416 | 0.06614625 | 0.105411481 | 0.67230064 | 0.039070567 | 0.487660049 | SUGT1 |
| ENSG00000109111 | 0.04431242 | 0.067864282 | 0.614941383 | 0.039810203 | 0.488356916 | SUPT6H |
| ENSG00000148290 | 0.06577915 | 0.040019708 | -0.716919684 | 0.018051956 | 0.375712616 | SURF1 |
| ENSG00000135316 | 0.04197482 | 0.071930838 | 0.777086222 | 0.012559923 | 0.335746964 | SYNCRIP |
| ENSG00000197283 | 0.05304096 | 0.01550468 | -1.77440326 | 0.005038388 | 0.233692211 | SYNGAP1 |
| ENSG00000143028 | 0.11825908 | 0.050935044 | -1.215220513 | 0.03455028 | 0.468313295 | SYPL2 |
| ENSG00000184292 | 0.07876735 | 0.046014654 | -0.775504428 | 0.036203406 | 0.474784041 | TACSTD2 |
| ENSG00000152382 | 0.0907064 | 0.174720096 | 0.94576926 | 0.002086323 | 0.166009597 | TADA1 |
| ENSG00000270647 | 0.10319967 | 0.183496847 | 0.830316926 | 0.036815297 | 0.477603236 | TAF15 |
| ENSG00000168394 | 0.08602624 | 0.046374971 | -0.891430354 | 0.007142486 | 0.267871649 | TAP1 |
| ENSG00000157014 | 0.01301843 | 0.004923871 | -1.402690431 | 0.022002567 | 0.401027305 | TATDN2 |
| ENSG00000111490 | 0.10680378 | 0.049870788 | -1.098695757 | 0.030964638 | 0.443089419 | TBC1D30 |
| ENSG00000109436 | 0.07721738 | 0.044902206 | -0.782139351 | 0.038924324 | 0.486834374 | TBC1D9 |
| ENSG00000285053 | 0.00721743 | 0.113284675 | 3.972324454 | 0.004148221 | 0.220217999 | TBCE |
| ENSG00000112592 | 0.1240536 | 0.31256426 | 1.333189183 | 9.04E-07 | 0.002323986 | TBP |
| ENSG00000179152 | 0.02683532 | 0.045371949 | 0.757667396 | 0.031462571 | 0.447881113 | TCAIM |
| ENSG00000113649 | 0.05029148 | 0.086442439 | 0.781425782 | 0.013514718 | 0.343720558 | TCERG1 |
| ENSG00000100207 | 0.01665519 | 0.029153909 | 0.807717815 | 0.025125147 | 0.409445587 | TCF20 |
| ENSG00000145022 | 0.00564076 | 0.001331825 | -2.082485409 | 0.002814181 | 0.189016552 | TCTA |
| ENSG00000139372 | 0.01458184 | 0.033569466 | 1.20297685 | 0.014419169 | 0.34780523 | TDG |
| ENSG00000107140 | 0.03664392 | 0.013721349 | -1.417151514 | 0.009872614 | 0.30080542 | TESK1 |
| ENSG00000187605 | 0.00095694 | 0.008541343 | 3.15795541 | 0.023566961 | 0.405084178 | TET3 |
| ENSG00000144043 | 0.00265161 | 0.000569277 | -2.219664858 | 0.002984795 | 0.195086856 | TEX261 |
| ENSG00000226674 | 30.6605671 | 9.196466592 | -1.73723281 | 0.018040566 | 0.375712616 | TEX41 |
| ENSG00000163235 | 0.03478729 | 0.018692402 | -0.896108177 | 0.035829837 | 0.471698475 | TGFA |
| ENSG00000069702 | 0.2518564 | 0.156800511 | -0.68367111 | 0.049935329 | 0.519301795 | TGFBR3 |
| ENSG00000152291 | 0.0705534 | 0.046775917 | -0.592949764 | 0.001504255 | 0.140670959 | TGOLN2 |
| ENSG00000113272 | 0.06241997 | 0.118205403 | 0.921216512 | 0.020078208 | 0.390545242 | THG1L |
| ENSG00000125676 | 0.0726231 | 0.121659479 | 0.74434826 | 0.022566722 | 0.40226007 | THOC2 |
| ENSG00000126351 | 0.00763547 | 0.024632846 | 1.689794968 | 0.011060593 | 0.319391043 | THRA |
| ENSG00000177370 | 0.03783886 | 0.023501306 | -0.687127524 | 0.023216409 | 0.405084178 | TIMM22 |
| ENSG00000035862 | 0.01615358 | 0.011386472 | -0.504533008 | 0.030665626 | 0.442612992 | TIMP2 |
| ENSG00000142910 | 0.09681842 | 0.058550729 | -0.725594392 | 0.015911812 | 0.360895332 | TINAGL1 |
| ENSG00000075131 | 0.04962968 | 0.136048183 | 1.454842595 | 0.002929053 | 0.192478365 | TIPIN |
| ENSG00000104953 | 0.41817438 | 0.061169555 | -2.773219003 | 0.048963803 | 0.513607879 | TLE6 |
| ENSG00000150403 | 0.14599319 | 0.090335591 | -0.692534654 | 0.014600526 | 0.351479685 | TMCO3 |
| ENSG00000096092 | 0.25049719 | 0.429818679 | 0.778933776 | 0.016848188 | 0.365211851 | TMEM14A |
| ENSG00000170006 | 0.03856395 | 0.085084924 | 1.141650846 | 0.026363979 | 0.416672417 | TMEM154 |
| ENSG00000157600 | 0.02552942 | 0.048576576 | 0.928100016 | 0.028771669 | 0.429477935 | TMEM164 |
| ENSG00000151353 | 0.07934982 | 0.145567512 | 0.875389582 | 0.011305583 | 0.321126116 | TMEM18 |
| ENSG00000184857 | 0.16810743 | 0.082810352 | -1.021500467 | 0.004809796 | 0.232710694 | TMEM186 |
| ENSG00000149932 | 0.03028238 | 0.017575448 | -0.784916942 | 0.010394497 | 0.310481322 | TMEM219 |
| ENSG00000153485 | 0.02423323 | 0.00464043 | -2.384656284 | 0.000284754 | 0.078287599 | TMEM251 |
| ENSG00000205544 | 0.11942386 | 0.236190534 | 0.983860023 | 0.013393495 | 0.342788875 | TMEM256 |
| ENSG00000121900 | 0.01536294 | 0.035253211 | 1.198300136 | 0.02647385 | 0.416672417 | TMEM54 |
| ENSG00000135211 | 0.10821517 | 0.176723604 | 0.707591985 | 0.039679762 | 0.488356916 | TMEM60 |
| ENSG00000167105 | 0.09391889 | 0.054122349 | -0.795190838 | 0.035890523 | 0.471698475 | TMEM92 |
| ENSG00000167920 | 0.01753749 | 0.030734217 | 0.809403453 | 0.037408272 | 0.479242828 | TMEM99 |
| ENSG00000161955 | 0.06722408 | 0.00470585 | -3.8364507 | 0.005454996 | 0.237693156 | TNFSF13 |
| ENSG00000079308 | 0.02203263 | 0.050925467 | 1.208745285 | 0.030042525 | 0.438447748 | TNS1 |
| ENSG00000141232 | 0.09799 | 0.194673946 | 0.990353427 | 0.018489224 | 0.376403414 | TOB1 |
| ENSG00000078902 | 0.02392094 | 0.015325695 | -0.642321795 | 0.044566279 | 0.502426364 | TOLLIP |
| ENSG00000175768 | 6.12929088 | 13.42764405 | 1.13141412 | 0.02444022 | 0.40599313 | TOMM5 |
| ENSG00000198900 | 0.04628712 | 0.081258657 | 0.811910828 | 0.015295429 | 0.356246208 | TOP1 |
| ENSG00000143514 | 0.03080056 | 0.051254016 | 0.734708575 | 0.032534026 | 0.454616267 | TP53BP2 |
| ENSG00000078900 | 0.00557704 | 0.241922877 | 5.438904446 | 1.23E-07 | 0.000745983 | TP73 |
| ENSG00000141933 | 0.02146399 | 0.043161599 | 1.007830199 | 0.025883601 | 0.415479489 | TPGS1 |
| ENSG00000134900 | 0.07896483 | 0.139120412 | 0.817051881 | 0.030822071 | 0.442917637 | TPP2 |
| ENSG00000136527 | 0.10031432 | 0.152945254 | 0.60848775 | 0.025021184 | 0.409078177 | TRA2B |
| ENSG00000056558 | 0.02410361 | 0.065673547 | 1.446063364 | 0.008997675 | 0.296533481 | TRAF1 |
| ENSG00000131323 | 0.01024448 | 0.00517023 | -0.986545926 | 0.045177966 | 0.504879453 | TRAF3 |
| ENSG00000183763 | 0.02008212 | 0.043265536 | 1.107306414 | 0.012056563 | 0.331529111 | TRAIP |
| ENSG00000225791 | 0.00558712 | 0.019426504 | 1.797850738 | 0.000958751 | 0.119062573 | TRAM2-AS1 |
| ENSG00000168016 | 0.06506273 | 0.022665745 | -1.521317792 | 0.042517719 | 0.49982388 | TRANK1 |
| ENSG00000007255 | 0.17856691 | 0.047309466 | -1.916263973 | 0.002611967 | 0.183547294 | TRAPPC6A |
| ENSG00000183665 | 0.1741535 | 0.029290945 | -2.57183278 | 0.000765929 | 0.111447705 | TRMT12 |
| ENSG00000122435 | 0.04292427 | 0.10515352 | 1.292631669 | 0.029775317 | 0.438168687 | TRMT13 |
| ENSG00000089195 | 0.08520785 | 0.138618764 | 0.702064274 | 0.048554909 | 0.512842768 | TRMT6 |
| ENSG00000253368 | 0.01101558 | 0.00405061 | -1.443333634 | 0.024579254 | 0.40765346 | TRNP1 |
| ENSG00000072756 | 0.04785205 | 0.084667539 | 0.823228221 | 0.02708893 | 0.420051176 | TRNT1 |
| ENSG00000165699 | 0.06347242 | 0.096272385 | 0.600992242 | 0.03957097 | 0.488356916 | TSC1 |
| ENSG00000011295 | 0.27502329 | 0.149196339 | -0.882341679 | 0.033987144 | 0.463208189 | TTC19 |
| ENSG00000165914 | 0.04941048 | 0.029966089 | -0.721486168 | 0.02964085 | 0.43731045 | TTC7B |
| ENSG00000116830 | 0.01525537 | 0.034328593 | 1.170093376 | 0.019923624 | 0.388159448 | TTF2 |
| ENSG00000155657 | 29.0095077 | 6.318010412 | -2.198983594 | 1.15E-06 | 0.002323986 | TTN |
| ENSG00000037042 | 0.01603887 | 0.038835315 | 1.275796503 | 0.026082924 | 0.415970645 | TUBG2 |
| ENSG00000126216 | 0.09559567 | 0.063591619 | -0.588108672 | 0.012797197 | 0.337649696 | TUBGCP3 |
| ENSG00000149016 | 0.04170133 | 0.007577722 | -2.46025706 | 0.004504419 | 0.229331663 | TUT1 |
| ENSG00000083223 | 0.08395522 | 0.136460227 | 0.700788586 | 0.02027438 | 0.391853157 | TUT7 |
| ENSG00000117862 | 0.48744739 | 0.946737055 | 0.957717286 | 0.010801192 | 0.316409856 | TXNDC12 |
| ENSG00000091164 | 0.07987486 | 0.116870914 | 0.549102616 | 0.025074349 | 0.409166249 | TXNL1 |
| ENSG00000162971 | 0.07602235 | 0.037682759 | -1.012519087 | 0.02802436 | 0.425333513 | TYW5 |
| ENSG00000197355 | 0.01266842 | 0.026659623 | 1.073420211 | 0.031439713 | 0.447881113 | UAP1L1 |
| ENSG00000134882 | 0.04421381 | 0.025296624 | -0.80555222 | 0.01047899 | 0.31147454 | UBAC2 |
| ENSG00000150991 | 0.01903245 | 0.032913784 | 0.790230563 | 0.007430452 | 0.272832425 | UBC |
| ENSG00000119048 | 0.0698141 | 0.044429967 | -0.651985356 | 0.022137517 | 0.401177406 | UBE2B |
| ENSG00000184787 | 0.03598359 | 0.022585182 | -0.671962671 | 0.013653125 | 0.344938512 | UBE2G2 |
| ENSG00000177889 | 0.01857651 | 0.011509434 | -0.690662523 | 0.035713801 | 0.471698475 | UBE2N |
| ENSG00000108312 | 0.02063849 | 0.037738281 | 0.870691001 | 0.009855078 | 0.30080542 | UBTF |
| ENSG00000143222 | 0.08261737 | 0.139443391 | 0.755162479 | 0.046014861 | 0.505789031 | UFC1 |
| ENSG00000169062 | 0.00942036 | 0.02539582 | 1.430737125 | 0.027417554 | 0.421530402 | UPF3A |
| ENSG00000156467 | 0.09208518 | 0.189950035 | 1.044579106 | 3.99E-05 | 0.03390847 | UQCRB |
| ENSG00000245248 | 0.0140504 | 0.095698632 | 2.767887047 | 0.041139843 | 0.493381361 | USP2-AS1 |
| ENSG00000165280 | 0.0227451 | 0.039061229 | 0.78018149 | 0.035653801 | 0.471646631 | VCP |
| ENSG00000111424 | 0.0088954 | 0.096091568 | 3.433278228 | 0.001221508 | 0.130680255 | VDR |
| ENSG00000150630 | 0.11707944 | 0.07118093 | -0.717925013 | 0.012566001 | 0.335746964 | VEGFC |
| ENSG00000128564 | 0.09299031 | 0.168571756 | 0.858210571 | 0.038214375 | 0.481421929 | VGF |
| ENSG00000026025 | 0.04889763 | 0.075136256 | 0.619744801 | 0.016853159 | 0.365211851 | VIM |
| ENSG00000139719 | 0.01817203 | 0.034931488 | 0.942808708 | 0.035160223 | 0.469376604 | VPS33A |
| ENSG00000261373 | 0.00152135 | 0.020419669 | 3.746532454 | 0.003168138 | 0.199184608 | VPS9D1-AS1 |
| ENSG00000009844 | 0.09331168 | 0.149464331 | 0.679671641 | 0.020338954 | 0.392477245 | VTA1 |
| ENSG00000132970 | 0.07435776 | 0.014501361 | -2.358295072 | 0.003475386 | 0.206098874 | WASF3 |
| ENSG00000226210 | 0.00805329 | 0.002209112 | -1.866111704 | 0.042081699 | 0.49919058 | WASH8P |
| ENSG00000227057 | 0.0358592 | 0.055749544 | 0.636616815 | 0.035029298 | 0.469376604 | WDR46 |
| ENSG00000095397 | 0.01784977 | 0.099125287 | 2.473347758 | 0.000101531 | 0.044719976 | WHRN |
| ENSG00000141499 | 0.07010397 | 0.110788528 | 0.660240468 | 0.017852511 | 0.374194788 | WRAP53 |
| ENSG00000047644 | 0.03377337 | 0.01979258 | -0.770926456 | 0.00937899 | 0.300053646 | WWC3 |
| ENSG00000196584 | 0.05704903 | 0.096464858 | 0.757801166 | 0.046135751 | 0.506097126 | XRCC2 |
| ENSG00000088930 | 0.04133117 | 0.065426362 | 0.662641824 | 0.03862426 | 0.484377942 | XRN2 |
| ENSG00000137693 | 0.04513008 | 0.06479364 | 0.521762839 | 0.036445652 | 0.477185192 | YAP1 |
| ENSG00000182362 | 0.07125641 | 0.156005924 | 1.130509059 | 0.03540498 | 0.469376604 | YBEY |
| ENSG00000060138 | 0.01999797 | 0.034142028 | 0.771695137 | 0.009023963 | 0.296533481 | YBX3 |
| ENSG00000174851 | 0.0489088 | 0.026856976 | -0.864797125 | 0.009265847 | 0.29843347 | YIF1A |
| ENSG00000180667 | 0.03325426 | 0.010033381 | -1.728731092 | 0.006575892 | 0.257881042 | YOD1 |
| ENSG00000047188 | 0.04924068 | 0.084740227 | 0.783196168 | 0.041453934 | 0.495475231 | YTHDC2 |
| ENSG00000213236 | 1.13012333 | 6.275739557 | 2.473305263 | 0.030296299 | 0.440458386 | YWHAZP2 |
| ENSG00000236829 | 0.06332109 | 0.225811816 | 1.83436297 | 0.021401604 | 0.397220313 | Z97634.1 |
| ENSG00000066422 | 0.01587795 | 0.053277597 | 1.746504721 | 0.005096925 | 0.233692211 | ZBTB11 |
| ENSG00000173276 | 0.03272459 | 0.06658569 | 1.024837146 | 0.032643975 | 0.455628934 | ZBTB21 |
| ENSG00000171448 | 0.004739 | 0.051107176 | 3.430872454 | 0.000140491 | 0.050959942 | ZBTB26 |
| ENSG00000168795 | 0.03845449 | 0.020495733 | -0.907828685 | 0.036755893 | 0.477603236 | ZBTB5 |
| ENSG00000178951 | 0.04288878 | 0.065587991 | 0.612831387 | 0.035345942 | 0.469376604 | ZBTB7A |
| ENSG00000158545 | 0.03533729 | 0.059732202 | 0.757317696 | 0.020627729 | 0.393707086 | ZC3H18 |
| ENSG00000188177 | 0.14451057 | 0.035504804 | -2.025088836 | 0.0071832 | 0.267871649 | ZC3H6 |
| ENSG00000168228 | 0.09099359 | 0.050721712 | -0.843161467 | 0.034673284 | 0.468879991 | ZCCHC4 |
| ENSG00000078487 | 0.00720437 | 0.030763164 | 2.094260554 | 0.043760005 | 0.502426364 | ZCWPW1 |
| ENSG00000104231 | 0.07119931 | 0.110641538 | 0.635957957 | 0.044458693 | 0.502426364 | ZFAND1 |
| ENSG00000178381 | 0.04504369 | 0.018967599 | -1.247788008 | 0.024193202 | 0.405119503 | ZFAND2A |
| ENSG00000091656 | 0.13550251 | 0.065471834 | -1.049373248 | 0.022541968 | 0.40226007 | ZFHX4 |
| ENSG00000196867 | 0.08167604 | 0.039590067 | -1.044774412 | 0.016661448 | 0.36351266 | ZFP28 |
| ENSG00000179588 | 0.00722168 | 0.029160745 | 2.013620279 | 0.026371886 | 0.416672417 | ZFPM1 |
| ENSG00000165156 | 0.07435069 | 0.043610361 | -0.769675256 | 0.044260022 | 0.502426364 | ZHX1 |
| ENSG00000196247 | 0.15072307 | 0.048607794 | -1.632640745 | 0.030858316 | 0.442917637 | ZNF107 |
| ENSG00000062370 | 0.11581741 | 0.020895513 | -2.470586989 | 0.017849245 | 0.374194788 | ZNF112 |
| ENSG00000178150 | 0.06842589 | 0.129341131 | 0.918566864 | 0.037047821 | 0.478418048 | ZNF114 |
| ENSG00000167635 | 0.05605222 | 0.03132015 | -0.839680436 | 0.011146572 | 0.319596419 | ZNF146 |
| ENSG00000131115 | 0.14181772 | 0.059837315 | -1.244920459 | 0.018142089 | 0.375712616 | ZNF227 |
| ENSG00000167840 | 0.09761777 | 0.028855229 | -1.758310982 | 0.047713349 | 0.510030853 | ZNF232 |
| ENSG00000264278 | 0.0176003 | 0.080438415 | 2.192284341 | 0.0411929 | 0.493381361 | ZNF236-DT |
| ENSG00000213096 | 0.00988113 | 0.033169244 | 1.747097611 | 0.020970552 | 0.395798212 | ZNF254 |
| ENSG00000063587 | 0.05559856 | 0.018167367 | -1.613698227 | 0.012614137 | 0.336293992 | ZNF275 |
| ENSG00000158805 | 0.00134587 | 0.028250334 | 4.391654618 | 8.41E-05 | 0.040908317 | ZNF276 |
| ENSG00000168661 | 0.11358718 | 0.028096875 | -2.015318464 | 0.011245 | 0.321009949 | ZNF30 |
| ENSG00000089335 | 0.05194646 | 0.022978168 | -1.176761649 | 0.046443077 | 0.507102816 | ZNF302 |
| ENSG00000169740 | 0.00149519 | 0.015896783 | 3.410330244 | 0.00259176 | 0.183186179 | ZNF32 |
| ENSG00000249471 | 0.0540839 | 0.009702598 | -2.47875623 | 3.39E-05 | 0.03390847 | ZNF324B |
| ENSG00000131061 | 0.00288084 | 0.032578967 | 3.499379929 | 0.006931781 | 0.266676147 | ZNF341 |
| ENSG00000165244 | 0.03603778 | 0.069170074 | 0.94063794 | 0.045012774 | 0.504879453 | ZNF367 |
| ENSG00000161298 | 0.01862532 | 0.201533295 | 3.435681334 | 0.004377825 | 0.226473293 | ZNF382 |
| ENSG00000126746 | 0.05608537 | 0.101934368 | 0.861944169 | 0.025220418 | 0.410383263 | ZNF384 |
| ENSG00000124613 | 0.17629911 | 0.08024705 | -1.135504961 | 0.023679512 | 0.405084178 | ZNF391 |
| ENSG00000130818 | 0.04054699 | 0.071942463 | 0.827248689 | 0.042585638 | 0.49982388 | ZNF426 |
| ENSG00000197857 | 0.04518993 | 0.121692915 | 1.429171798 | 0.026613054 | 0.416672417 | ZNF44 |
| ENSG00000101493 | 0.04445918 | 0.017002895 | -1.386701058 | 0.045498825 | 0.504879453 | ZNF516 |
| ENSG00000175322 | 0.05738112 | 0.224892724 | 1.97058888 | 0.00483934 | 0.232710694 | ZNF519 |
| ENSG00000152433 | 0.18407468 | 0.014822378 | -3.634442356 | 0.001310867 | 0.134907466 | ZNF547 |
| ENSG00000251369 | 0.0813171 | 0.022125479 | -1.877850035 | 0.038120214 | 0.481369204 | ZNF550 |
| ENSG00000171827 | 0.04801088 | 0.216083542 | 2.170156041 | 0.024965409 | 0.409035682 | ZNF570 |
| ENSG00000171574 | 0.02816346 | 0.050382006 | 0.839083753 | 0.022475958 | 0.40226007 | ZNF584 |
| ENSG00000180626 | 0.01130774 | 0.05709225 | 2.335984661 | 0.030718426 | 0.442612992 | ZNF594 |
| ENSG00000173545 | 0.025394 | 0.053023803 | 1.062152685 | 0.000575514 | 0.098542631 | ZNF622 |
| ENSG00000198093 | 0.10514701 | 0.020959668 | -2.326720023 | 0.004638706 | 0.230174477 | ZNF649 |
| ENSG00000197372 | 0.06478113 | 0.16457316 | 1.345083434 | 0.028284668 | 0.426092576 | ZNF675 |
| ENSG00000185730 | 0.02880445 | 0.011278736 | -1.35268649 | 0.03711016 | 0.478418048 | ZNF696 |
| ENSG00000196110 | 0.04233387 | 0.229494668 | 2.438576347 | 0.001020134 | 0.119356439 | ZNF699 |
| ENSG00000160336 | 0.00167791 | 0.018349356 | 3.4509881 | 0.009767485 | 0.30080542 | ZNF761 |
| ENSG00000133624 | 0.05727134 | 0.01182871 | -2.27552062 | 0.022283386 | 0.401927489 | ZNF767P |
| ENSG00000188227 | 2.98101197 | 0.119703379 | -4.638266393 | 0.002146624 | 0.167285336 | ZNF793 |
| ENSG00000235944 | 0.10547721 | 0.021381635 | -2.302487198 | 0.047995007 | 0.510030853 | ZNF815P |
| ENSG00000198783 | 0.05085026 | 0.119377254 | 1.231200835 | 0.002756556 | 0.188266548 | ZNF830 |
| ENSG00000223547 | 0.00782185 | 0.038652229 | 2.304969311 | 0.023914505 | 0.405084178 | ZNF844 |
| ENSG00000267041 | 0.17371277 | 0.066956939 | -1.375398323 | 0.009716768 | 0.30080542 | ZNF850 |
| ENSG00000178917 | 0.06158264 | 0.19636191 | 1.672919474 | 0.049083709 | 0.513962667 | ZNF852 |
| ENSG00000132485 | 0.0610846 | 0.135780331 | 1.152393991 | 0.007175467 | 0.267871649 | ZRANB2 |
| ENSG00000169249 | 0.03430303 | 0.139228943 | 2.02105108 | 0.00085278 | 0.11519159 | ZRSR2 |
| ENSG00000196812 | 0.0148916 | 0.067333769 | 2.176831852 | 0.014883024 | 0.35232888 | ZSCAN16 |
| ENSG00000070476 | 0.04965353 | 0.083548122 | 0.750711077 | 0.012370535 | 0.335688827 | ZXDC |

**Table S8**. Primers, antibodies, key resources, and softwares used in this study

| REAGENT or RESOURCE | SOURCE | IDENTIFIER |
| --- | --- | --- |
| **Antibodies** |  |  |
| FIBCD1 | Novus | NBP2-87441 |
| H3K27ac | Abcam | Ab4729 |
| Cyclin A1 | Abcam | Ab270940 |
| Cyclin D1 | Abcam | Ab134175 |
| Cyclin E1 | Proteintech | 11554-1-ap |
| Ki-67 | Proteintech | 28074 |
| MCM5 | Proteintech | 11703 |
| β-Actin | Cell Signaling Technology | 4970S |
| GAPDH | Aksomics | KC-5G5 |
| **Biological samples** |  |  |
| Human breast cancer tumor tissue | Cancer Hospital of Harbin Medical University |  |
| **Primers** | | |
| Name | Sequences (5′ to 3′) | |
| qPCR-human-FIBCD1-F | TGTCATCCCCACTCCTTGTG | |
| qPCR-human-FIBCD1-R | TGCCATCGGGTGTCCTTT | |
| qPCR-human-MCM5-F | GATCCTGGCATTTTCTACAG | |
| qPCR-human-MCM5-R | CCCTGTATTTGAAGGTGAAG | |
| qPCR-human-PRIM2-F | CGGCTTGCTTATTGCCAGTCT | |
| qPCR-human-PRIM2-R | CAATCTCCTGTTCTCGAAGAGTC | |
| qPCR-human-LIG1-F | CAGAGGGCGAGTTTGTCTTC | |
| qPCR-human-LIG1-R | AGCCAGTTGTGCGATCTCTT | |
| qPCR-human-CXCL2-F | TCTCAACCCCGCATCGC | |
| qPCR-human-CXCL2-R | ACAGCCACCAATAAGCTTCC | |
| qPCR-human-ACTB-F | GTGGCCGAGGACTTTGATTG | |
| qPCR-human-ACTB-R | CCTGTAACAACGCATCTCATATT | |
| qPCR-human-18s-F | CCTGGATACCGCAGCTAGGA | |
| qPCR-human-18s-R | GCGGCGCAATACGAATGCCCC | |
| qPCR-mouse-FIBCD1-F | CAAGGCTGACCTTCAGAGGG | |
| qPCR-mouse-FIBCD1-R | GGGGAAGATAGAGTAGACACCAT | |
| qPCR-mouse-ACTB-F | GGCTGTATTCCCCTCCATCG | |
| qPCR-mouse-ACTB-R | CCAGTTGGTAACAATGCCATGT | |
| MCM5 ChIP 1-F | CCACCATGCCCAGCTAATTA | |
| MCM5 ChIP 1-R | GCCTTGAACTCCTGACCTCAG | |
| MCM5 ChIP 2-F | AGTGCCTATAGATCAAGGTTG | |
| MCM5 ChIP 2-R | TCATTCTGTTTAACAGCTGC | |
| MCM5 ChIP 3-F | TGCAAAATGGGCACCTGGAC | |
| MCM5 ChIP 3-R | AAGCACTGGGATTACTGGCG | |
| MCM5 ChIP 4-F | GCACACAAAATGGAGCCCGG | |
| MCM5 ChIP 4-R | AGCGATTGGACCGTTCTGAG | |
| **siRNA** | **Target sequence (5′ to 3′)** | |
| si-FIBCD1 #1 | GCATGAGGTTCACCACCAA | |
| si-FIBCD1 #2 | GACCATTCAGAGAACAACT | |
| si-FIBCD1 #3 | GGCAGTACTCACTCAAGTT | |
| si-MCM5 #1 | CCCGGAATTTCATCATGGA | |
| si-MCM5 #2 | GCATCTACTCCATCAAGAA | |
| si-MCM5 #3 | GCTCCCTGATGGACTTACT |  |
| **shRNA** | **Target sequence (5′ to 3′)** |  |
| sh-FIBCD1 #1 | GCGAGCAAGCTGCAGTTATGT | |
| sh-FIBCD1 #2 | GCACAGTGGCATGAGGTTTAC | |
| sh-FIBCD1 #3 | GCAACTGTCACACATCCAACC | |
| Experimental models: Organisms/strains | | |
| BALB/C mice | Slac Animals (Shanghai, China) |  |
| MCF7 | Cell Bank of the Chinese Academy of Sciences (Shanghai, China) | TCHu 74 |
| MDA-MB-231 | Cell Bank of the Chinese Academy of Sciences (Shanghai, China) | TCHu227 |
| T47D | Cell Bank of the Chinese Academy of Sciences (Shanghai, China) | TCHu 87 |
| 4T1 | Cell Bank of the Chinese Academy of Sciences (Shanghai, China) | SCSP-5056 |
| **Chemicals** |  |  |
| Dulbecco's Modified Eagle Medium (DMEM), high glucose | Gibco | 10566016 |
| Dulbecco's Modified Eagle Medium (DMEM), low glucose | Gibco | 11885084 |
| Dulbecco's Modified Eagle Medium (DMEM), no glucose | Gibco | 11966025 |
| Leibovitz's L-15 culture medium | Gibco | 11415064 |
| RPMI 1640 culture medium | Gibco | 11875119 |
| D -(+)-Glucose solution | Sigma | G8644 |
| Fetal bovine serum (FBS) | Gibco | 10270106 |
| Phosphate buffered saline (PBS) | Gibco | 10010049 |
| Trypsin-EDTA (0.25%) | Gibco | 25200072 |
| Penicillin-Streptomycin | Gibco | 15070063 |
| DMSO | Sigma | D2650 |
| Bovine serum albumin (BSA) | Sigma | V900933 |
| Agarose | Sigma | V900510 |
| Crystal violet | Sigma | C0775 |
| SuperSignal West Pico PLUS | Thermo Scientific | 34577 |
| PageRuler | Thermo Scientific | 26616 |
| Skimmed milk powder | Thermo Scientific | LP0033B |
| SDS-PAGE protein loading buffer(5X) | Beyotime | P0015L |
| Protease and phosphatase inhibitor cocktail for mammalian cell and tissue extracts (50X) | Beyotime | P1050 |
| Cell lysis buffer for Western and IP without inhibitors | Beyotime | P0013J |
| Matrigel™ Matrix | Corning | 356234 |
| Transwell™ Multiwell Plate | Corning | 3464 |
| Cell culture inserts | Falcon | 353097 |
| Puromycin | Aladdin | P113126 |
| PrimeScriptTM RT master mix | TaKaRa | RR036A |
| TB Green TM Premix Ex Taq TM II | TaKaRa | RR420A |
| Lenti-X Concentrator | TaKaRa | 631232 |
| TBS (20x) | Solarbio | T1080 |
| Propidium iodide (PI) | Solarbio | C0080 |
| RNase A solution | Solarbio | R1030 |
| Streptozocin (STZ) | Sangon Biotech | A427651 |
| OPTI-MEM | Invitrogen | 31985-070 |
| 1kbp DNA Ladder Marker | TaKaRa | D517A |
| Trypsin | Hyclone | SH30042.02 |
| Countess chamber slide | Invitrogen | C10283 |
| **Critical commercial assays** |  |  |
| SteadyPure Universal RNA Extraction Kit | Accurate Biology | AG21017 |
| PAGE Gel rapid preparation kit | Epizyme Biotech | PG112 |
| Cell Counting Kit-8 (CCK-8) | Meilunbio | MA0218 |
| Lipofectamine 3000 Transfection Kit | Invitrogen | 2309838 |
| EndoFree Plasmid Maxi Kit | QIAGEN | 12362 |
| The Pierce BCA protein assay kit | Thermo Scientific | 23225 |
| **Software and algorithms** |  |  |
| ImageJ 1.53a | USA |  |
| GraphPad Prism | GraphStats |  |
| R Studio | R v4.1.3 |  |
| Gene Ontology | http://release.geneontology.org |  |
| Gene Set Enrichment Analysis (GSEA) | https://www.gsea-msigdb.org/gsea/index.jsp |  |

| . **Table S9. Treatment-associated effect sample size (ESS)** | | | | |
| --- | --- | --- | --- | --- |
|  | **IPTW** | | **TMLE+SL** | |
| **Treatment** | **ESS** | **ESS_j_/n_j_** | **ESS** | **ESS_j_/n_j_** |
| DM | 157.51 | 0.53 | 204.28 | 0.68 |
| Non-DM | 3051.23 | 0.99 | 3057.12 | 0.99 |

| **Table S10. Clinical characteristics of Chinese breast cancer patients** | | | |
| --- | --- | --- | --- |
| **Variable** | **Our cohort**  **N = 3386** | **Other Cohort 1^1^**  **N=1214** | **Other Cohort 2^2^**  **N=311** |
| Age | 50.50 (44.00, 58.00) | 47.00 (42.00, 55.00) | 47.32 (23.00-76.00) |
| BMI | 23.83 (21.64, 26.02) | 23.62 (21.48, 25.78) |  |
| Stage I | 1535 (45.33%) | 372 (31.0%) | 133 (42.77%) |
| Stage II | 1376 (40.64%) | 592 (49.3%) | 133 (42.77%) |
| Stage III | 475 (14.03%) | 236 (19.7%) | 45 (14.47%) |
| ER | 2459 (72.62%) | 1016 (84.0%) | 244 (78.46%) |
| PR | 2156 (63.67%) | 859 (71.1%) | 218 (70.10%） |


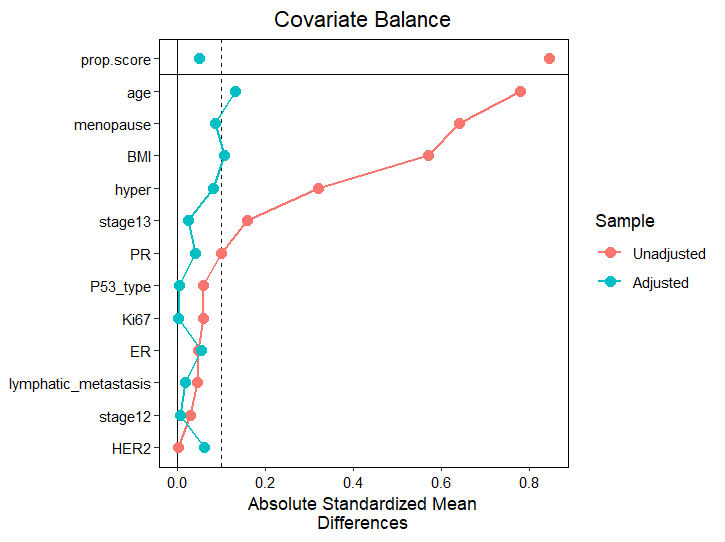


**Figure S1**


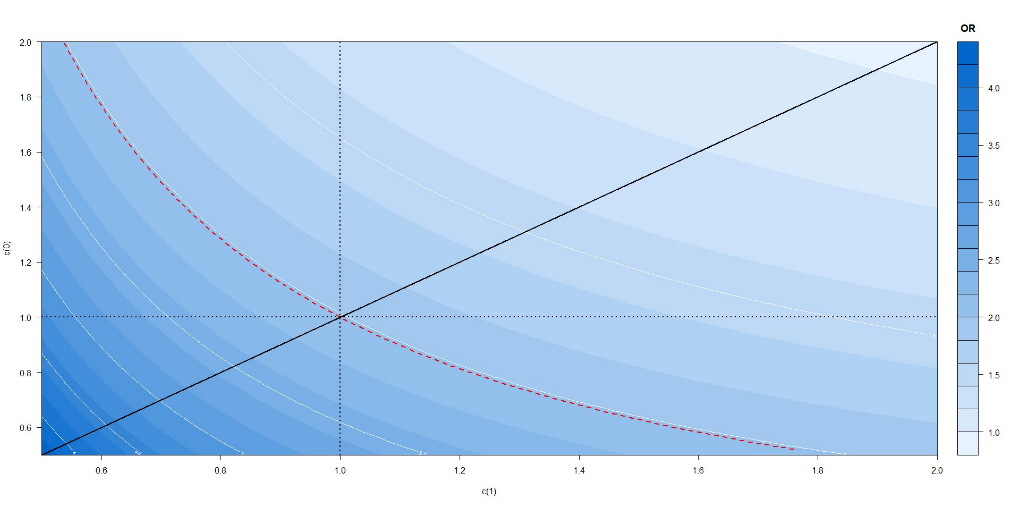


**Figure S2**
