# Supplementary figures and images for: Diabetes is causally associated with increased breast cancer mortality by inducing FIBCD1 to activate MCM5-mediated cell cycle arrest via modulating H3K27ac
Source: Cell Death Dis. 2025 Jul 22;16(1):546. doi: 10.1038/s41419-025-07849-w (PMC12283923; doi:10.1038/s41419-025-07849-w)

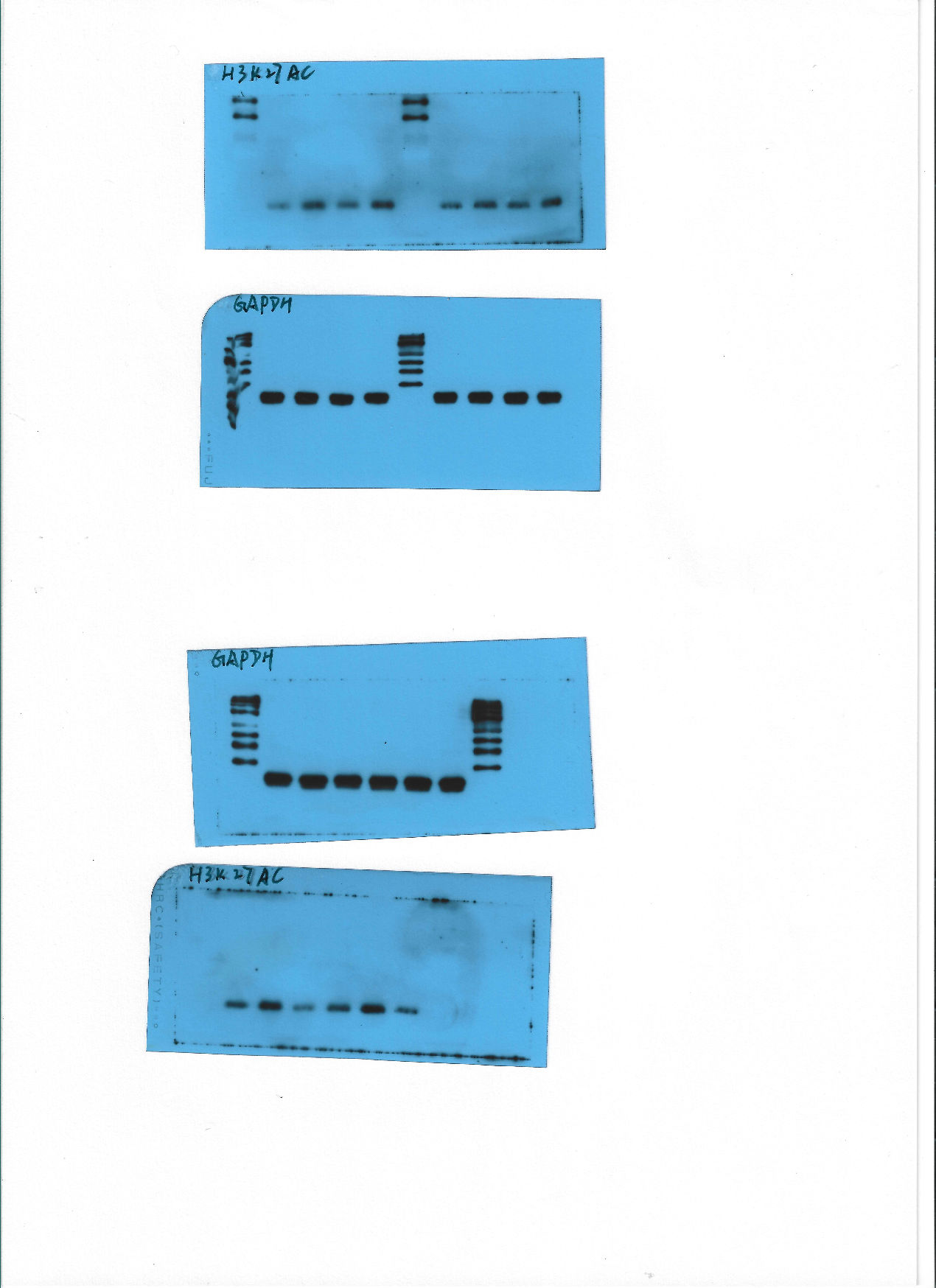


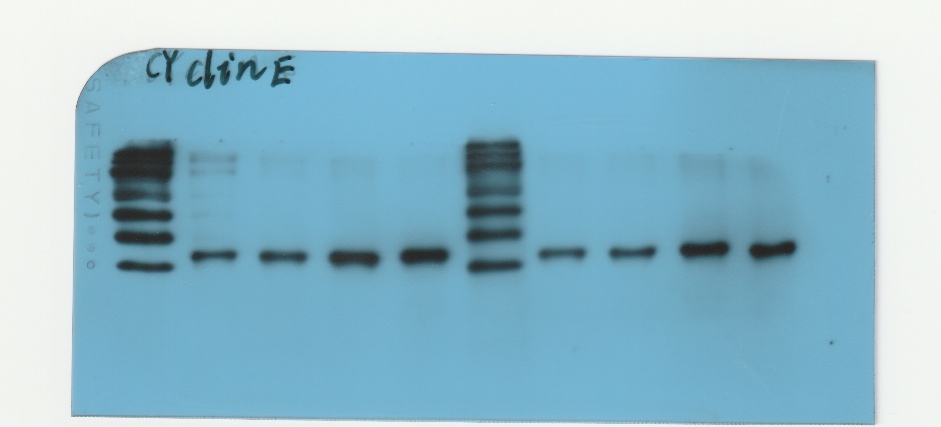


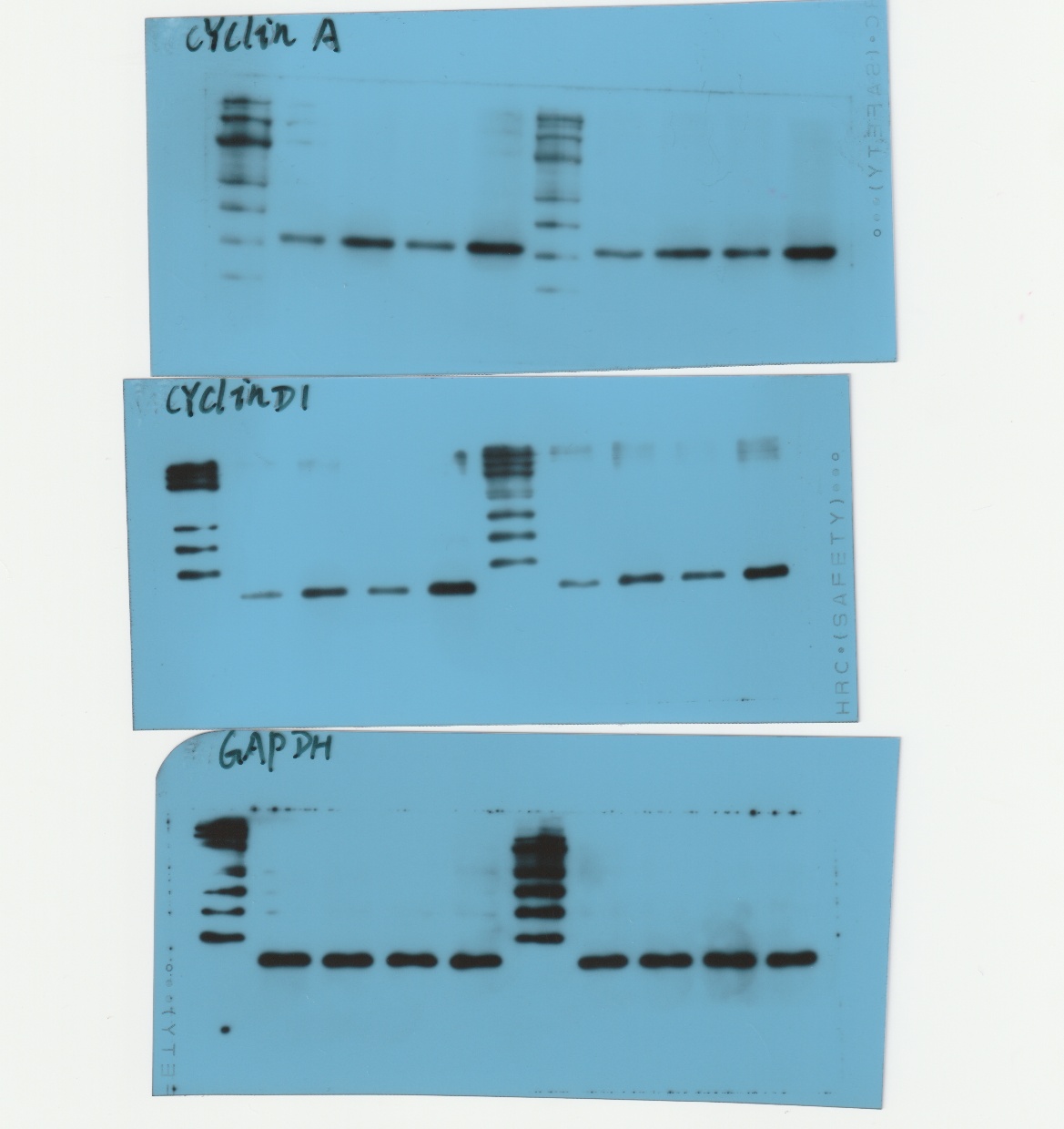


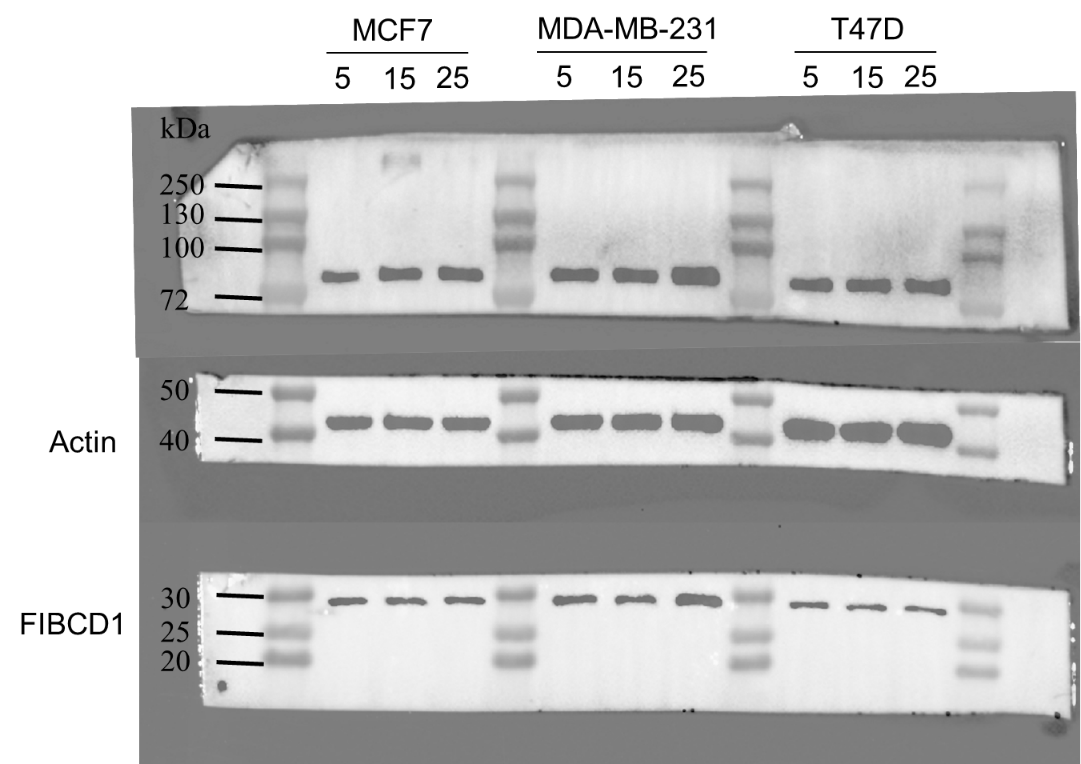


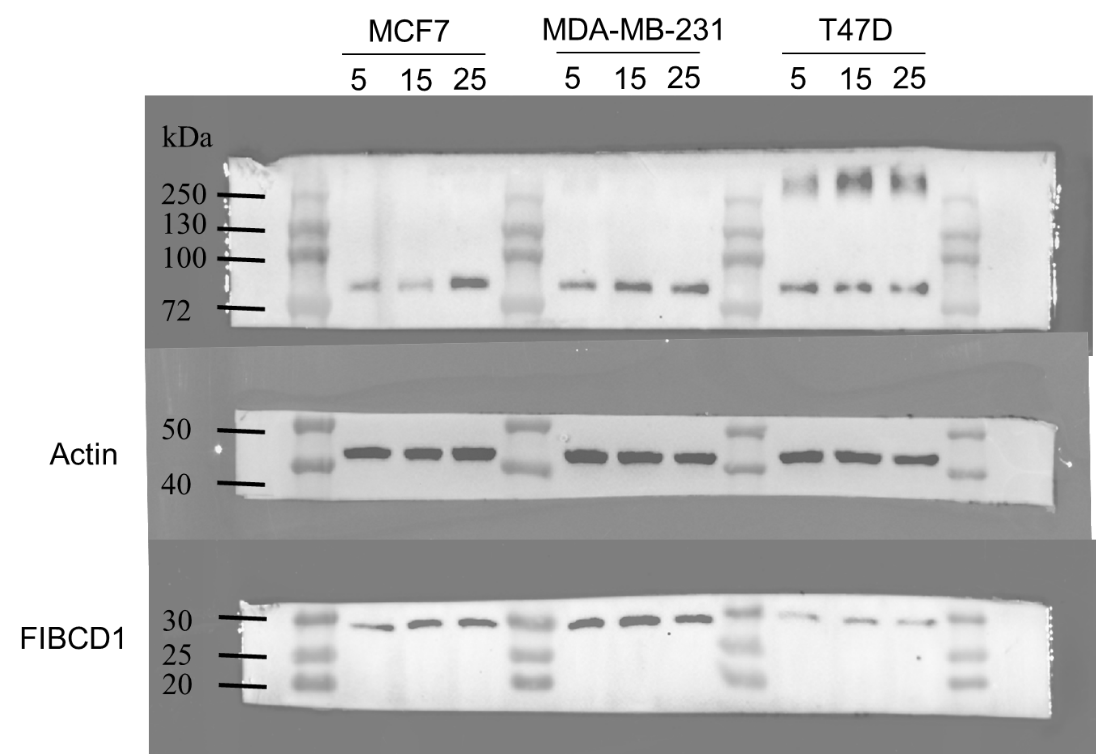

Supplement: Supplementary file 2 — Supplementary materials [file 41419_2025_7849_MOESM2_ESM.docx]
