# Supplementary material for: Diabetes is causally associated with increased breast cancer mortality by inducing FIBCD1 to activate MCM5-mediated cell cycle arrest via modulating H3K27ac
Source: Cell Death Dis. 2025 Jul 22;16(1):546. doi: 10.1038/s41419-025-07849-w (PMC12283923; doi:10.1038/s41419-025-07849-w)
Supplement: Supplementary file 3 — Supplementary materials [file 41419_2025_7849_MOESM3_ESM.docx]

| **Table S11. Characteristics of epidemiological cohort vs. tissue sequencing patients** | | | |
| --- | --- | --- | --- |
| **Variable*** | **Epidemiological cohort**  **N = 3386** | **Tissue sequencing subjects**  **N = 13** | **Adjusted**  ***P*-value^#^** |
| Age | 50.50 (44.00, 58.00) | 57.00 (50.00, 60.00) | 0.120 |
| BMI | 23.83 (21.64, 26.02) | 26.95 (25.30, 27.88) | 0.053 |
| Stage I | 1535 (45.33%) | 11 (84.62%) | 0.052 |
| Stage II | 1376 (40.64%) | 2 (15.38%) |  |
| Stage III | 475 (14.03%) | 0 (0%) |  |
| Post-Menopause | 1568 (46.31%) | 10 (76.92%) | 0.068 |

*Continuous variables are expressed as mean ± SD (normal distribution) or median (interquartile range; not normal distribution) and compared using *t* tests (normal distribution) or Wilcoxon rank-sum tests (not normal distribution) as appropriate. Categorical variables are expressed as n (%) and compared using chi-squared tests.

# *P*-values adjusted by the Benjamini and Hochberg method to control the false discovery rate at 5%
